# National, regional, and global estimates of anaemia by severity in women and children for 2000–19: a pooled analysis of population-representative data

**DOI:** 10.1016/S2214-109X(22)00084-5

**Published:** 2022-04-12

**Authors:** Gretchen A Stevens, Christopher J Paciorek, Monica C Flores-Urrutia, Elaine Borghi, Sorrel Namaste, James P Wirth, Parminder S Suchdev, Majid Ezzati, Fabian Rohner, Seth R Flaxman, Lisa M Rogers

**Affiliations:** a Independent researcher, Los Angeles, CA, USA; b School of Public Health, Imperial College London, London, UK; c Department of Statistics, University of California, Berkeley, CA, USA; d Department of Nutrition and Food Safety, World Health Organization, Geneva, Switzerland; e The DHS Program, ICF, Rockville, MD, USA; f GroundWork, Fläsch, Switzerland; g Department of Pediatrics and Hubert Department of Global Health, Emory University, Atlanta, GA, USA; h Regional Institute for Population Studies, University of Ghana, Legon, Ghana; i Department of Computer Science, University of Oxford, Oxford, UK

## Abstract

**Background:**

Anaemia causes health and economic harms. The prevalence of anaemia in women aged 15–49 years, by pregnancy status, is indicator 2.2.3 of the UN Sustainable Development Goals, and the aim of halving the anaemia prevalence in women of reproductive age by 2030 is an extension of the 2025 global nutrition targets endorsed by the World Health Assembly (WHA). We aimed to estimate the prevalence of anaemia by severity for children aged 6–59 months, non-pregnant women aged 15–49 years, and pregnant women aged 15–49 years in 197 countries and territories and globally for the period 2000–19.

**Methods:**

For this pooled analysis of population-representative data, we collated 489 data sources on haemoglobin distribution in children and women from 133 countries, including 4·5 million haemoglobin measurements. Our data sources comprised health examination, nutrition, and household surveys, accessed as anonymised individual records or as summary statistics such as mean haemoglobin and anaemia prevalence. We used a Bayesian hierarchical mixture model to estimate haemoglobin distributions in each population and country-year. This model allowed for coherent estimation of mean haemoglobin and prevalence of anaemia by severity.

**Findings:**

Globally, in 2019, 40% (95% uncertainty interval [UI] 36–44) of children aged 6–59 months were anaemic, compared to 48% (45–51) in 2000. Globally, the prevalence of anaemia in non-pregnant women aged 15–49 years changed little between 2000 and 2019, from 31% (95% UI 28–34) to 30% (27–33), while in pregnant women aged 15–49 years it decreased from 41% (39–43) to 36% (34–39). In 2019, the prevalence of anaemia in children aged 6–59 months exceeded 70% in 11 countries and exceeded 50% in all women aged 15–49 years in ten countries. Globally in all populations and in most countries and regions, the prevalence of mild anaemia changed little, while moderate and severe anaemia declined in most populations and geographical locations, indicating a shift towards mild anaemia.

**Interpretation:**

Globally, regionally, and in nearly all countries, progress on anaemia in women aged 15–49 years is insufficient to meet the WHA global nutrition target to halve anaemia prevalence by 2030, and the prevalence of anaemia in children also remains high. A better understanding of the context-specific causes of anaemia and quality implementation of effective multisectoral actions to address these causes are needed.

**Funding:**

USAID, US Centers for Disease Control and Prevention, and Bill & Melinda Gates Foundation.

## Introduction

Anaemia can lead to reduced physical work capacity, poor maternal and perinatal health outcomes for pregnant women, and delayed growth, cognition, and motor development in children.^1^ Common causes of anaemia are nutritional deficiencies, infectious and inflammatory diseases, and genetic haemoglobin disorders.^2^ Iron-deficiency anaemia was estimated to cause 22% (95% uncertainty interval [UI] 8–35) of maternal deaths in 2019.^3^

Awareness about anaemia and its consequences for women's and children's health and development has increased in the past decade. In 2012, the 65th World Health Assembly (WHA) approved global targets for maternal, infant, and young child nutrition, with a commitment to halve anaemia prevalence in women of reproductive age (15–49 years) by 2025.^4^ WHO and UNICEF proposed extending this target to 2030 to align with the UN Sustainable Development Goals (SDGs).^5^ In 2020, the prevalence of anaemia in women aged 15–49 years was added as indicator 2.2.3 of the SDGs.^6^

More than 5 years into the SDG era, little is known about global progress towards the anaemia target. We aimed to estimate anaemia prevalence in children aged 6–59 months, non-pregnant women aged 15–49 years, and pregnant women aged 15–49 years during 2000–19. We assessed progress towards the WHA target and investigated whether trends in mild anaemia differed from those of moderate and severe anaemia, which have more severe health sequelae and are more likely to be treated in a clinical setting. We also assessed the influence of factors associated with the measurement of haemoglobin in household surveys.^7^, ^8^

**Research in context**

**Evidence before this study**

We searched the following databases from Jan 1, 2000, to Feb 13, 2021, for relevant studies: AGRICOLA, AIM (AFRO), CINAHL, EMBASE, IBECS, IMEMR (EMRO), IMSEAR, IndMED, LILACS, MEDLINE, PAHO, SCIELO, Web of Science, WHOLIS, and WPRIM. No language restrictions were applied and the following search terms were used: “(((national) AND (survey)) OR ((population) AND (prevalence))) AND ((iron status) OR (iron deficiency) OR (anaemia) OR (anaemia) OR (haemoglobin) OR (haemoglobin) OR (low iron level) OR (transferrin receptor) OR (ferritin) OR (insufficient iron))”. We found two articles that estimated the national, regional, and global prevalence of anaemia and its trends, one presenting WHO estimates and one presenting estimates from the Global Burden of Disease Study 2010 (GBD 2010). Both of these studies were subsequently updated with estimates made available through respective online portals. Since the global studies were published, new population-based data have been collected, and anaemia in women aged 15–49 years has been adopted as an indicator under the UN Sustainable Development Goal (SDG) 2 to end hunger, achieve food security and improved nutrition, and promote sustainable agriculture, with an associated World Health Assembly (WHA) target of reducing the prevalence of anaemia in women by 50% by 2030. We also found several studies considering specific regions or countries, including one that assessed progress towards the WHA anaemia target in low-income and middle-income countries.

**Added value of this study**

In this pooled analysis, we estimated trends in haemoglobin distributions and anaemia prevalence by severity for three target population groups—pregnant women aged 15–49 years, non-pregnant women aged 15–49 years, and children aged 6–59 months—for the years 2000–19 using a Bayesian hierarchical mixture model with new data sources, revised covariates, and a focus on factors associated with the measurement of haemoglobin, which might introduce bias or error in estimates. We estimated the national, regional, and global progress towards the WHA anaemia target and compared progress on reducing anaemia in women and children by severity.

**Implications of all the available evidence**

These estimates indicate that there might have been slower declines in anaemia prevalence in all groups in 2010–19 compared to 2000–09, and that in all but two countries—Guatemala and the Philippines—there has been insufficient progress to meet the WHA target. Reductions in moderate and severe anaemia in pregnant women and children aged 6–59 months and severe anaemia in non-pregnant women were most marked, but globally and in most regions the prevalence of mild anaemia, which has less severe health consequences, barely changed in all population groups. Renewed efforts are needed to better understand the causes of anaemia in each country context, with particular attention given to anaemia severity, and to ensure quality implementation of effective multisectoral strategies to address those causes.

## Methods

### Overview

For this pooled analysis of population-representative data, we estimated trends between 2000 and 2019 in the population distributions of haemoglobin for children aged 6–59 months, non-pregnant women aged 15–49 years, and pregnant women aged 15–49 years in 197 countries and territories organised into 11 epidemiologically and climatically relevant regions (appendix p 17). Our analysis included four steps. The first step involved identifying, assessing, and accessing data sources on haemoglobin and anaemia; the second step involved accounting for complex sample design of population-based surveys; the third step involved adjusting haemoglobin for elevation and smoking; and the fourth involved applying a statistical model to estimate trends in blood haemoglobin distributions and their uncertainties for children aged 6–59 months and for women aged 15–49 years by pregnancy status.

The distributions estimated in the fourth step allowed coherent and consistent estimation of mean haemoglobin concentrations and of the prevalence of anaemia by severity. We used WHO definitions of mild, moderate, severe, and total anaemia (table 1).^9^ The locations of Guidelines for Accurate and Transparent Health Estimates Reporting (GATHER) items are given in the appendix (pp 21, 22).^10^

**Table 1:** Classification of anaemia by blood haemoglobin concentration at sea level and population

	**Mild anaemia**	**Moderate anaemia**	**Severe anaemia**
Children aged 6–59 months	100–109 g/L	70–99 g/L	<70 g/L
Non-pregnant women aged ≥15 years	110–119 g/L	80–109 g/L	<80 g/L
Pregnant women	100–109 g/L	70–99 g/L	<70 g/L

Source: WHO, 2011.^9^

### Data sources

Our data identification, access, and inclusion strategy was designed to obtain as many data sources as possible while ensuring that the sources were representative of at least three first administrative units within each country (appendix pp 2–6). In brief, our data sources comprised health examination, nutrition, and household surveys that included haemoglobin measurement of children aged 6–59 months or women aged 15–49 years, or both. We accessed data as anonymised individual records or as summary statistics, including mean haemoglobin and anaemia prevalence, from the Micronutrients Database of the WHO Vitamin and Mineral Nutrition Information System. Data were identified for the Micronutrients Database via a systematic search of the peer-reviewed literature, an international network of collaborators, and a country consultation. Our data search closed on Feb 13, 2021.

### Statistical methods

Most data on population haemoglobin distribution come from household-based surveys that used complex sample designs. To reflect this, we computed an effective sample size for each data source accessed as individual-level data and estimated the effective sample size for data sources accessed as summary statistics, as described in the appendix (pp 6, 7).

Physiological haemoglobin needs are greater at high elevation (due to the lower concentration of oxygen in the atmosphere) and in smokers (due to their reduced oxygen-carrying capacity).^9^ We used data adjusted for altitude and smoking when available, and adjusted other data as described in the appendix (pp 8, 9).

The statistical methods used to estimate trends in haemoglobin distribution are described in detail in a previous publication^11^ and in the appendix (pp 9–14). In brief, we used a Bayesian hierarchical mixture model to make estimates for each country-year. In the hierarchical model, estimates for each country-year were informed by data from that country-year itself, if available, and by data from other years in the same country and in other countries, especially those in the same region with data in similar time periods. We modelled trends over time as a linear trend plus a smooth non-linear trend, at the country, regional, and global levels. We selected the following time-varying covariates to help predict haemoglobin concentrations when data were sparse or inconsistent (appendix pp 10, 11): Socio-demographic Index,^12^ meat supply (kcal per capita),^13^, ^14^ mean body-mass index (BMI;^15^ for women aged 15–49 years only), and log of shock-free under-5 mortality (for children aged 6–59 months only).^16^ We also assessed whether the blood collection method (ie, venous or capillary blood) should be explicitly included in the model but found that its inclusion had little effect on the estimated haemoglobin distribution (appendix p 12). A variance term was included that accounted for design factors (eg, blood collection method, as well as sample design, season, and haemoglobin measurement method) that lead to additional variability in the data beyond that expected due to sample size. Finally, the model accounted for the fact that subnational data and data that do not exactly cover the age ranges of interest might have larger variation than national data and data that match age ranges of interest. We fitted the model to data collected from 1995 to 2020 to limit boundary effects but reported results between 2000 and 2019, because there were few data sources from 1995 to 1999, and 2020 might not have followed past trends due to the COVID-19 pandemic.

All reported uncertainty intervals are 95% Bayesian credible intervals. Average changes in mean haemoglobin or anaemia prevalence were calculated over the 20 reporting years (2000–19) and for each decade (2000–09 and 2010–19; absolute for mean and proportional for prevalence) and reported as change per decade. We report the posterior probability that an estimated increase or decrease represents a truly increasing or decreasing trend. The posterior probability would be 0·50 if an increase is statistically indistinguishable from a decrease, and a posterior probability greater than 0·50 indicates greater certainty. We also computed the average annual rate of reduction from 2012 (the baseline year for the WHA anaemia target) to 2019 and the posterior probability of meeting the WHA anaemia target, assuming the trends observed in 2012–19 continue (appendix p 14).

### Role of the funding source

The funding sources had no role in study design, data collection, data analysis, data interpretation, writing of the report, or the decision to submit the paper for publication.

## Results

We identified, accessed, and included 489 population-representative data sources from 133 of 197 countries and territories, with 4·5 million haemoglobin measurements. Of these data sources, 458 were nationally representative and 304 sampled capillary blood (appendix p 18). Data availability was similar for children aged 6–59 months and women aged 15–49 years, with 393 data sources from 122 countries covering 91% of children aged 6–59 months worldwide, and 408 data sources from 124 countries covering 90% of women aged 15–49 years. Country estimates of trends in haemoglobin distribution were more reliable if several data sources collected at different timepoints were available. For women aged 15–49 years, at least three data sources were included in 64 countries (covering 72% of women), and for children aged 6–59 months at least three data sources were included in 63 countries (covering 60% of children). Data availability varied by region (appendix p 24). An average of more than three data sources per country were available for both populations in south, east, and southeast Asia, and for children aged 6–59 months in east, west, and central Africa. Data were sparse for children aged 6–59 months in high-income countries, and for both populations in Oceania and central and eastern Europe (<1 data source per country).

**Figure 1:**
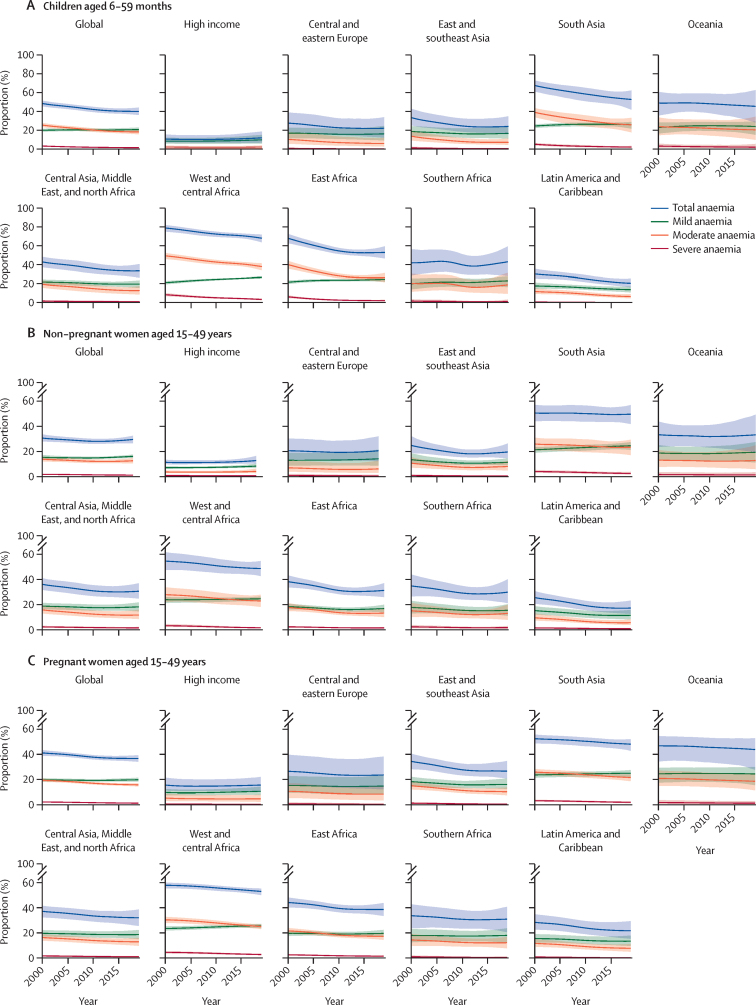
Prevalence of anaemia globally and by region, year, and severity (A) Children aged 6–59 months. (B) Non-pregnant women aged 15–49 years. (C) Pregnant women aged 15–49 years. The prevalence of mild, moderate, and severe anaemia sums to total anaemia. Shaded areas show the 95% uncertainty intervals.

Globally in 2019, 21% (95% UI 19–23) of children aged 6–59 months had mild anaemia, 18% (16–20) had moderate anaemia, and 1% (1–2) had severe anaemia (table 2, figure 1; WHO global anaemia estimates available online). The prevalence of anaemia in this population varied by region, with west and central Africa having the highest prevalence and the high-income countries having the lowest prevalence throughout the analysis period. Regions with a higher overall prevalence of anaemia in children aged 6–59 months also had a larger proportion of those with anaemia who had moderate or severe anaemia.

**Table 2:** Prevalence of total, severe, moderate, and mild anaemia, by world region in 2000 and 2019, for children aged 6–59 months, non-pregnant women aged 15–49 years, pregnant women aged 15–49 years, and all women aged 15–49 years

	**2000**				**2019**			
	Totalanaemia	Mildanaemia	Moderate anaemia	Severe anaemia	Total anaemia	Mildanaemia	Moderate anaemia	Severe anaemia
**Children aged 6–59 months**								
High-income countries	10% (7–15)	8% (6–12)	2% (1–4)	0% (0–0)	12% (7–18)	10% (6–15)	2% (1–4)	0% (0–0)
Central and eastern Europe	27% (18–39)	17% (11–22)	10% (6–18)	1% (0–1)	22% (12–34)	16% (9–24)	6% (3–12)	0% (0–1)
East and southeast Asia	33% (25–42)	18% (14–24)	13% (9–19)	1% (1–2)	24% (16–35)	16% (11–25)	7% (4–11)	0% (0–1)
South Asia	67% (60–72)	24% (22–26)	38% (33–43)	5% (3–6)	52% (41–61)	25% (21–28)	25% (17–33)	2% (1–3)
Oceania	48% (35–60)	22% (16–28)	23% (14–33)	3% (1–5)	45% (28–62)	23% (15–29)	20% (9–34)	2% (0–5)
Central Asia, Middle East, and North Africa	43% (37–48)	22% (19–24)	19% (16–23)	2% (1–2)	33% (26–41)	20% (16–23)	13% (9–17)	1% (0–2)
West and central Africa	79% (75–82)	21% (19–23)	49% (46–52)	8% (7–10)	68% (64–72)	27% (25–28)	38% (34–41)	3% (3–4)
East Africa	68% (63–73)	22% (20–23)	40% (36–44)	6% (5–8)	53% (47–59)	24% (22–26)	26% (22–31)	2% (1–3)
Southern Africa	42% (29–56)	20% (14–25)	20% (12–31)	2% (1–4)	43% (28–59)	23% (17–28)	19% (9–31)	1% (0–3)
Latin America and Caribbean	30% (25–36)	18% (15–21)	12% (9–15)	1% (0–1)	20% (16–25)	14% (11–17)	6% (5–9)	0% (0–1)
Global	48% (45–51)	20% (18–21)	25% (23–27)	3% (3–4)	40% (36–44)	21% (19–23)	18% (16–20)	1% (1–2)
**Non-pregnant women aged 15–49 years**								
High-income countries	11% (10–13)	7% (6–8)	4% (3–5)	1% (0–1)	13% (10–16)	8% (7–10)	4% (3–6)	0% (0–1)
Central and eastern Europe	21% (13–30)	13% (8–19)	7% (4–13)	1% (0–2)	21% (12–32)	14% (9–21)	6% (3–13)	0% (0–1)
East and southeast Asia	25% (19–32)	13% (10–18)	11% (7–15)	1% (0–1)	20% (15–26)	11% (9–14)	8% (5–13)	0% (0–1)
South Asia	51% (44–57)	21% (19–23)	26% (21–31)	4% (3–5)	50% (42–57)	24% (21–27)	23% (17–29)	2% (1–3)
Oceania	33% (24–44)	19% (13–25)	13% (8–21)	2% (0–3)	33% (19–49)	19% (11–27)	13% (6–25)	1% (0–3)
Central Asia, Middle East, and North Africa	36% (32–41)	19% (16–21)	15% (13–19)	2% (1–3)	30% (25–37)	18% (14–22)	11% (8–16)	1% (1–2)
West and central Africa	55% (48–62)	24% (21–26)	28% (22–34)	3% (2–4)	49% (43–55)	25% (22–26)	23% (18–28)	1% (1–2)
East Africa	38% (34–43)	18% (16–21)	18% (15–21)	2% (2–3)	31% (26–37)	17% (14–20)	13% (10–17)	1% (1–2)
Southern Africa	35% (27–44)	18% (13–23)	15% (10–21)	2% (1–4)	30% (21–40)	15% (11–20)	13% (8–20)	2% (1–3)
Latin America and Caribbean	25% (21–31)	15% (12–19)	9% (7–13)	1% (1–2)	17% (12–23)	11% (8–16)	5% (3–8)	1% (0–1)
Global	31% (28–34)	15% (14–17)	14% (12–16)	2% (1–2)	30% (27–33)	16% (15–17)	13% (10–15)	1% (1–1)
**Pregnant women aged 15–49 years**								
High-income countries	15% (11–21)	10% (8–12)	5% (3–9)	1% (0–1)	15% (10–22)	10% (8–14)	5% (2–8)	0% (0–1)
Central and eastern Europe	26% (15–39)	15% (9–23)	10% (5–17)	1% (0–2)	23% (12–38)	15% (8–23)	8% (3–15)	0% (0–1)
East and southeast Asia	34% (28–40)	18% (14–22)	15% (12–18)	1% (1–2)	27% (21–35)	16% (12–21)	10% (8–14)	0% (0–1)
South Asia	52% (48–55)	23% (21–25)	26% (23–28)	3% (2–4)	48% (43–52)	25% (21–27)	21% (18–24)	2% (1–3)
Oceania	46% (34–54)	24% (17–29)	21% (15–27)	2% (0–4)	44% (28–52)	24% (15–30)	18% (11–24)	1% (0–3)
Central Asia, Middle East, and North Africa	37% (32–41)	19% (17–22)	16% (14–18)	1% (1–2)	32% (25–38)	18% (15–22)	13% (9–16)	1% (0–1)
West and central Africa	57% (55–59)	23% (21–25)	30% (27–32)	4% (3–5)	52% (50–55)	25% (23–27)	25% (23–26)	3% (2–3)
East Africa	44% (40–48)	20% (18–22)	22% (19–24)	3% (2–3)	39% (34–44)	20% (17–22)	17% (15–20)	2% (1–2)
Southern Africa	34% (24–43)	18% (13–23)	15% (10–19)	1% (1–2)	31% (21–41)	18% (12–24)	12% (8–17)	1% (0–1)
Latin America and Caribbean	29% (23–35)	16% (13–19)	12% (9–15)	1% (1–2)	22% (16–29)	14% (10–18)	8% (5–11)	0% (0–1)
Global	41% (39–43)	20% (18–21)	19% (18–20)	2% (2–2)	36% (34–39)	20% (18–21)	16% (14–17)	1% (1–2)
**All women aged 15–49 years**								
High-income countries	11% (10–13)	7% (6–8)	4% (3–5)	1% (0–1)	13% (10–16)	8% (7–10)	4% (3–6)	0% (0–1)
Central and eastern Europe	21% (14–30)	13% (9–19)	7% (4–13)	1% (0–2)	21% (13–32)	14% (9–21)	6% (3–13)	0% (0–1)
East and southeast Asia	25% (19–32)	14% (10–18)	11% (7–15)	1% (0–1)	20% (15–26)	11% (9–15)	8% (5–13)	0% (0–1)
South Asia	51% (45–57)	21% (19–23)	26% (21–30)	4% (3–5)	50% (42–57)	24% (21–27)	23% (17–29)	2% (1–3)
Oceania	34% (25–44)	19% (13–25)	14% (8–21)	2% (1–3)	34% (21–49)	20% (12–27)	13% (6–24)	1% (0–3)
Central Asia, Middle East, and North Africa	36% (32–41)	19% (16–21)	16% (13–19)	2% (1–3)	31% (25–37)	18% (15–21)	11% (8–16)	1% (1–2)
West and central Africa	55% (49–61)	24% (22–26)	28% (23–33)	3% (2–4)	49% (44–55)	25% (23–26)	23% (19–27)	2% (1–2)
East Africa	39% (35–44)	19% (17–21)	18% (16–21)	2% (2–3)	32% (27–37)	17% (14–20)	14% (11–17)	1% (1–2)
Southern Africa	35% (27–43)	18% (13–23)	15% (10–21)	2% (1–4)	30% (22–40)	16% (11–20)	13% (8–20)	2% (1–3)
Latin America and Caribbean	26% (21–31)	15% (12–18)	9% (7–13)	1% (1–2)	17% (13–23)	11% (8–16)	5% (4–8)	1% (0–1)
Global	31% (29–34)	15% (14–17)	14% (12–16)	2% (1–2)	30% (27–33)	16% (15–18)	13% (11–15)	1% (1–1)

Numbers in parentheses represent 95% uncertainty intervals. See the appendix (pp 19, 20) for mean haemoglobin concentrations by world region in 2000 and 2019.

In 2000, the prevalence of anaemia in children aged 6–59 months exceeded 80% in nine countries in west and central Africa and in Yemen, and anaemia prevalence exceeded 70% in an additional 19 countries in east, west, and central Africa (see WHO global anaemia estimates available online). By 2019, no country had an anaemia prevalence higher than 80% in children aged 6–59 months, and the prevalence of anaemia exceeded 70% in children aged 6–59 months in 11 countries (Yemen and ten countries in west and central Africa). At the other extreme, anaemia prevalence in the USA was around 6% during 2000–19. Other high-income countries also had low anaemia prevalence, but data availability was low in these countries (appendix p 24).

Globally and in most regions, the prevalence of anaemia in children aged 6–59 months declined between 2000 and 2019 (from 48% [95% UI 45–51] to 40% [36–44], posterior probability of a true decline >0·99; figure 1). Declines in anaemia were largest (relative reductions of 12–20% per decade and a posterior probability of a true decline >0·92) in Latin America and the Caribbean, east and southeast Asia, central Asia, the Middle East and North Africa, east Africa, and south Asia. Anaemia prevalence in children aged 6–59 months in 2000 in these regions ranged from less than 35% in Latin America and the Caribbean and in east and southeast Asia to more than 65% in south Asia and east Africa, meaning that reductions in anaemia occurred despite a wide range of starting points (appendix p 25). Countries within these regions with large declines in anaemia prevalence in children aged 6–59 months include Guatemala, the Philippines, Uzbekistan, Brazil, Kazakhstan, Panama, and Azerbaijan (estimated relative decline >25% per decade; appendix pp 30–161). In total, anaemia prevalence declined in 141 countries (posterior probability of a true decline >0·95 in 36 of these countries), and possible increases in prevalence (posterior probability of a true increase <0·70) were estimated for the remaining 56 countries. Globally, moderate and severe anaemia declined more rapidly than total anaemia, with relative declines of 17% per decade for moderate anaemia and 37% per decade for severe anaemia (posterior probability of a true decline >0·99). The global prevalence of mild anaemia in children aged 6–59 months was stable (posterior probability of an increase: 0·62), with stable, slightly increasing, or decreasing regional and country trends. In east, west, and central Africa, and south Asia, mild anaemia might have increased (posterior probability of an increase >0·75), while moderate and severe anaemia decreased (relative declines of 12–44% per decade; posterior probability of a true decline >0·99).

Increases in mean haemoglobin in children aged 6–59 months might have slowed globally in 2010–19 (0·9 [95% UI –0·9 to 2·7] g/L per decade) compared to 2000–09 (2·6 [1·1 to 4·1] g/L per decade; appendix p 26), with a similar pattern in most regions. This pattern was most notable in east Africa, where mean haemoglobin was estimated to increase to 6·5 g/L per decade (95% UI 3·6 to 9·3) during 2000–09 versus 0·8 g/L per decade (–2·3 to 3·7) during 2010–19. Globally and in most regions, large, overlapping uncertainty intervals for the trends mean that this pattern is uncertain.

**Figure 2:**
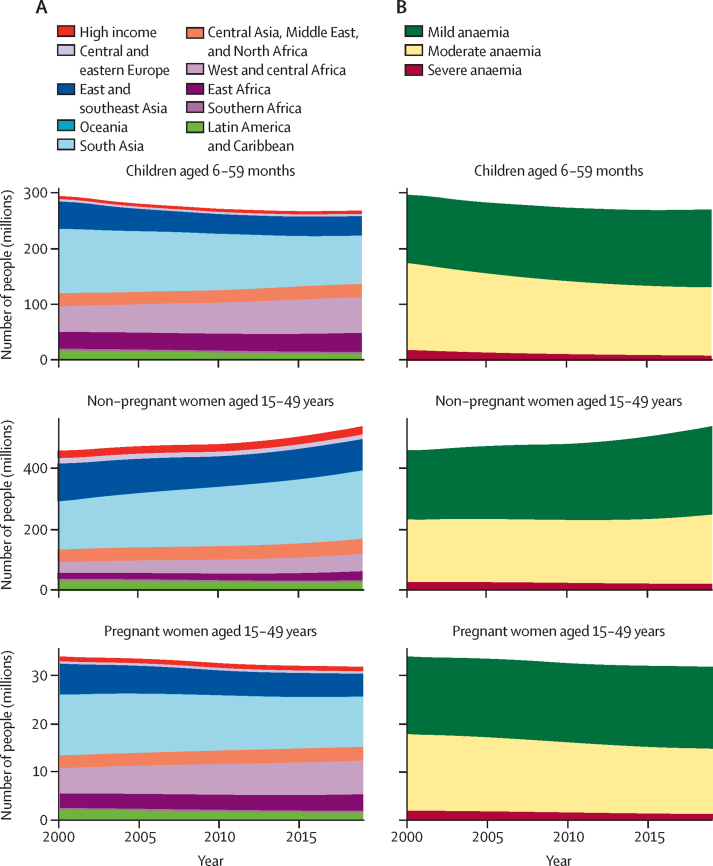
Number of children aged 6–59 months and pregnant and non-pregnant women aged 15–49 years with anaemia (A) By region. (B) By anaemia severity.

The global number of children aged 6–59 months with anaemia declined slightly from 296 million (95% UI 277–314) in 2000 to 269 million (244–297) in 2019 (figure 2). The number of children aged 6–59 months with anaemia increased in some regions despite declines in prevalence due to the increasing population, most notably in west and central Africa (increasing from 46 million to 64 million children aged 6–59 months between 2000 and 2019; table 3). This increase was offset by reductions in the number of children aged 6–59 months with anaemia in south Asia and in east and southeast Asia, together accounting for 43 million fewer children aged 6–59 months with anaemia in 2019. In terms of anaemia severity, globally the number of children aged 6–59 months with mild anaemia increased between 2000 and 2019, from 122 million (95% UI 113–131) to 139 million (126–153); this increase was offset by decreases in the number of children aged 6–59 months with moderate and severe anaemia (table 3, figure 2).

**Table 3:** Number of children aged 6–59 months, non-pregnant women aged 15–49 years, pregnant women aged 15–49 years, and all women aged 15–49 years with anaemia, by severity and world region in 2000 and 2019

	**2000**				**2019**			
	Total anaemia (millions)	Mild anaemia (millions)	Moderate anaemia (millions)	Severe anaemia (millions)	Total anaemia (millions)	Mild anaemia (millions)	Moderate anaemia (millions)	Severe anaemia (millions)
**Children aged 6–59 months**								
High-income countries	5·62 (3·72–8·20)	4·43 (3·00–6·44)	1·12 (0·570–1·94)	0·063 (0·011–0·211)	6·18 (3·49–9·69)	5·07 (2·97–7·79)	1·08 (0·454–1·96)	0·024 (0·003–0·100)
Central and eastern Europe	4·36 (2·89–6·17)	2·66 (1·80–3·57)	1·62 (0·882–2·83)	0·086 (0·025–0·231)	4·00 (2·20–6·14)	2·92 (1·65–4·43)	1·06 (0·463–2·16)	0·025 (0·004–0·093)
East and southeast Asia	49·0 (36·9–62·7)	27·5 (20·7–35·8)	19·8 (13·3–28·6)	1·66 (0·762–3·41)	34·8 (23·3–50·4)	24·0 (15·9–35·8)	10·3 (6·34–16·3)	0·456 (0·211–1·03)
South Asia	116 (105–125)	41·6 (37·9–44·6)	66·5 (57·7–74·3)	7·86 (5·65–10·3)	87·0 (69·0–103)	42·4 (35·4–47·1)	42·0 (28·7–54·5)	2·60 (1·23–4·68)
Oceania	0·558 (0·406–0·694)	0·259 (0·184–0·319)	0·269 (0·163–0·382)	0·030 (0·010–0·060)	0·630 (0·389–0·871)	0·329 (0·205–0·403)	0·279 (0·130–0·476)	0·022 (0·004–0·069)
Central Asia, Middle East, and North Africa	23·7 (20·7–26·7)	12·0 (10·6–13·4)	10·7 (8·69–12·9)	0·925 (0·439–1·38)	23·9 (18·9–29·0)	14·0 (11·1–16·7)	9·13 (6·14–12·4)	0·764 (0·160–1·28)
West and central Africa	46·1 (43·8–47·9)	12·3 (11·3–13·3)	28·9 (26·9–30·6)	4·90 (4·00–5·84)	64·2 (60·1–67·8)	25·1 (23·9–26·2)	35·8 (32·2–39·1)	3·20 (2·38–4·15)
East Africa	30·1 (27·8–32·1)	9·57 (8·77–10·3)	17·8 (15·9–19·5)	2·75 (2·19–3·39)	34·3 (30·2–38·4)	15·9 (14·4–17·1)	17·0 (13·9–20·2)	1·38 (0·891–1·99)
Southern Africa	3·12 (2·13–4·19)	1·49 (1·05–1·89)	1·51 (0·861–2·27)	0·128 (0·040–0·272)	3·84 (2·51–5·29)	2·05 (1·47–2·47)	1·68 (0·839–2·80)	0·108 (0·024–0·290)
Latin America and Caribbean	17·1 (14·4–20·2)	10·0 (8·33–11·9)	6·70 (5·28–8·56)	0·404 (0·243–0·656)	10·6 (8·42–13·2)	7·14 (5·65–8·83)	3·36 (2·37–4·73)	0·123 (0·052–0·265)
Global	296 (277–314)	122 (113–131)	155 (143–168)	18·8 (15·8–22·1)	269 (244–297)	139 (126–153)	122 (105–138)	8·70 (6·67–11·2)
**Non-pregnant women aged 15–49 years**								
High-income countries	24·7 (21·4–29·2)	15·4 (13·8–17·7)	7·76 (5·94–10·3)	1·54 (1·08–2·24)	27·3 (21·8–35·2)	17·7 (14·8–22·0)	8·60 (5·62–13·2)	0·979 (0·518–1·69)
Central and eastern Europe	17·8 (11·6–26·2)	11·2 (7·35–16·4)	5·94 (3·20–11·0)	0·677 (0·216–1·70)	14·9 (8·86–23·3)	10·2 (6·19–15·6)	4·37 (2·12–9·24)	0·312 (0·075–0·927)
East and southeast Asia	124 (94·3–160)	67·1 (51·3–92·2)	52·8 (34·9–76·7)	3·85 (2·07–7·25)	102 (76·0–138)	59·0 (46·6–75·6)	41·6 (25·0–65·5)	1·73 (0·935–3·24)
South Asia	157 (137–177)	65·8 (58·5–72·7)	80·0 (64·5–95·5)	11·5 (8·48–15·3)	223 (188–256)	109 (94·7–122)	104 (75·7–131)	9·79 (5·54–15·5)
Oceania	0·585 (0·414–0·768)	0·329 (0·221–0·439)	0·230 (0·134–0·370)	0·027 (0·008–0·061)	0·876 (0·508–1·30)	0·509 (0·294–0·722)	0·335 (0·156–0·655)	0·032 (0·008–0·090)
Central Asia, Middle East, and North Africa	40·7 (35·7–46·2)	21·0 (18·3–23·9)	17·5 (14·4–21·4)	2·12 (1·46–3·05)	49·2 (39·7–59·5)	29·0 (23·2–34·7)	18·4 (13·4–25·2)	1·79 (0·940–2·95)
West and central Africa	36·2 (31·5–40·9)	15·8 (14·1–17·2)	18·4 (14·7–22·3)	2·01 (1·38–2·84)	57·5 (50·5–64·5)	28·9 (26·5–31·0)	26·9 (21·3–32·7)	1·64 (1·16–2·24)
East Africa	18·8 (16·6–21·3)	9·07 (8·01–10·2)	8·70 (7·26–10·4)	1·08 (0·806–1·40)	29·2 (24·3–34·6)	15·7 (13·0–18·5)	12·3 (9·34–15·9)	1·23 (0·814–1·81)
Southern Africa	5·48 (4·18–6·90)	2·78 (2·03–3·60)	2·35 (1·60–3·34)	0·342 (0·167–0·571)	6·21 (4·39–8·34)	3·20 (2·30–4·20)	2·69 (1·63–4·09)	0·324 (0·141–0·600)
Latin America and Caribbean	33·4 (27·1–40·3)	19·8 (15·7–24·3)	12·2 (9·34–16·8)	1·47 (0·936–2·46)	28·2 (20·5–38·1)	18·5 (13·2–25·8)	8·77 (5·79–13·3)	0·902 (0·441–1·63)
Global	459 (420–503)	228 (208–256)	206 (180–237)	24·6 (20·4–29·9)	539 (485–592)	292 (268–318)	228 (190–268)	18·7 (13·7–25·1)
**Pregnant women aged 15–49 years**								
High-income countries	1·02 (0·732–1·40)	0·634 (0·497–0·810)	0·347 (0·199–0·568)	0·034 (0·011–0·076)	0·984 (0·658–1·39)	0·667 (0·492–0·901)	0·300 (0·148–0·507)	0·018 (0·004–0·050)
Central and eastern Europe	0·513 (0·301–0·768)	0·294 (0·180–0·440)	0·204 (0·103–0·330)	0·015 (0·004–0·035)	0·510 (0·263–0·827)	0·318 (0·176–0·500)	0·184 (0·075–0·324)	0·008 (0·001–0·024)
East and southeast Asia	6·45 (5·27–7·60)	3·42 (2·72–4·13)	2·80 (2·25–3·41)	0·229 (0·129–0·378)	4·74 (3·68–6·17)	2·84 (2·20–3·70)	1·81 (1·35–2·42)	0·085 (0·040–0·163)
South Asia	12·7 (11·8–13·5)	5·70 (5·17–6·17)	6·24 (5·65–6·83)	0·758 (0·593–0·943)	10·5 (9·35–11·4)	5·42 (4·71–5·97)	4·67 (4·04–5·24)	0·395 (0·240–0·577)
Oceania	0·075 (0·055–0·087)	0·039 (0·028–0·047)	0·033 (0·024–0·043)	0·003 (0·001–0·006)	0·082 (0·052–0·098)	0·045 (0·029–0·055)	0·034 (0·021–0·046)	0·002 (0·000–0·005)
Central Asia, Middle East, and North Africa	2·67 (2·33–2·99)	1·41 (1·23–1·59)	1·16 (0·983–1·34)	0·103 (0·065–0·145)	2·88 (2·29–3·47)	1·66 (1·34–2·00)	1·14 (0·857–1·42)	0·071 (0·028–0·119)
West and central Africa	5·33 (5·09–5·53)	2·15 (2·00–2·31)	2·79 (2·55–3·00)	0·395 (0·316–0·471)	7·07 (6·68–7·37)	3·40 (3·17–3·60)	3·33 (3·05–3·57)	0·341 (0·260–0·423)
East Africa	2·97 (2·70–3·24)	1·33 (1·19–1·48)	1·46 (1·31–1·61)	0·184 (0·147–0·221)	3·45 (2·99–3·90)	1·75 (1·51–2·00)	1·55 (1·31–1·79)	0·147 (0·099–0·203)
Southern Africa	0·340 (0·240–0·432)	0·182 (0·128–0·234)	0·146 (0·099–0·194)	0·012 (0·005–0·021)	0·355 (0·237–0·467)	0·207 (0·139–0·272)	0·141 (0·090–0·192)	0·007 (0·002–0·016)
Latin America and Caribbean	2·05 (1·65–2·52)	1·13 (0·901–1·39)	0·855 (0·661–1·09)	0·070 (0·043–0·111)	1·41 (1·00–1·90)	0·876 (0·633–1·17)	0·513 (0·338–0·729)	0·025 (0·012–0·046)
Global	34·1 (32·3–35·9)	16·3 (15·2–17·4)	16·0 (15·0–17·1)	1·80 (1·55–2·07)	32·0 (29·8–34·2)	17·2 (15·9–18·6)	13·7 (12·6–14·8)	1·10 (0·874–1·35)
**All women aged 15–49 years**								
High-income countries	25·8 (22·4–30·3)	16·1 (14·4–18·3)	8·11 (6·28–10·7)	1·57 (1·11–2·27)	28·3 (22·7–36·2)	18·4 (15·4–22·6)	8·90 (5·91–13·6)	0·996 (0·530–1·71)
Central and eastern Europe	18·3 (12·1–26·9)	11·5 (7·63–16·7)	6·15 (3·36–11·3)	0·691 (0·222–1·73)	15·4 (9·35–23·9)	10·6 (6·50–15·9)	4·55 (2·26–9·56)	0·320 (0·077–0·949)
East and southeast Asia	130 (100–167)	70·5 (54·4–95·8)	55·6 (37·4–79·5)	4·08 (2·24–7·60)	107 (80·3–143)	61·8 (49·2–78·7)	43·5 (26·5–67·6)	1·81 (0·992–3·36)
South Asia	170 (149–190)	71·5 (64·1–78·7)	86·3 (70·4–102)	12·3 (9·14–16·2)	234 (198–267)	115 (99·9–128)	109 (80·2–136)	10·2 (5·81–16·0)
Oceania	0·660 (0·484–0·845)	0·368 (0·256–0·479)	0·263 (0·161–0·405)	0·029 (0·010–0·066)	0·958 (0·583–1·39)	0·554 (0·338–0·769)	0·370 (0·182–0·692)	0·034 (0·008–0·095)
Central Asia, Middle East, and North Africa	43·3 (38·2–48·8)	22·4 (19·7–25·4)	18·7 (15·4–22·7)	2·22 (1·54–3·17)	52·1 (42·3–62·7)	30·6 (24·8–36·5)	19·6 (14·3–26·4)	1·86 (0·980–3·04)
West and central Africa	41·6 (36·8–46·3)	17·9 (16·2–19·4)	21·2 (17·4–25·2)	2·41 (1·73–3·28)	64·6 (57·3–71·7)	32·3 (29·8–34·5)	30·3 (24·6–36·1)	1·98 (1·43–2·64)
East Africa	21·8 (19·4–24·4)	10·4 (9·29–11·5)	10·2 (8·68–11·9)	1·26 (0·964–1·61)	32·7 (27·5–38·1)	17·4 (14·7–20·3)	13·9 (10·7–17·5)	1·38 (0·918–2·00)
Southern Africa	5·82 (4·48–7·25)	2·97 (2·19–3·79)	2·50 (1·72–3·51)	0·354 (0·175–0·587)	6·57 (4·70–8·76)	3·40 (2·47–4·44)	2·83 (1·74–4·28)	0·331 (0·144–0·611)
Latin America and Caribbean	35·5 (29·0–42·4)	20·9 (16·7–25·4)	13·1 (10·1–17·8)	1·54 (0·990–2·57)	29·6 (21·9–39·7)	19·4 (14·1–26·7)	9·28 (6·19–13·9)	0·927 (0·455–1·67)
Global	493 (453–538)	244 (225–272)	222 (196–254)	26·4 (22·0–32·0)	571 (515–625)	309 (285–336)	242 (203–282)	19·8 (14·6–26·5)

Numbers in parentheses represent 95% uncertainty intervals. Numbers 1 million or larger are reported to 3 significant figures and numbers less than 1 million are reported to three decimal places.

Globally in 2019, 30% (95% UI 27–33) of non-pregnant women aged 15–49 years and 36% (34–39) of pregnant women aged 15–49 years were anaemic; this represents a modest decrease from 2000 for pregnant women aged 15–49 years (a relative decline of 7% per decade; posterior probability of a true decline >0·99) and little change for non-pregnant women aged 15–49 years (a relative decline of 3% per decade; posterior probability of a true decline: 0·79). Because of the increasing population, the number of women aged 15–49 years with anaemia increased from 493 million (95% UI 453–538) in 2000 to 571 million (515–625) in 2019 (table 3). Patterns and trends in anaemia by severity in all women aged 15–49 years followed the same pattern as non-pregnant women aged 15–49 years (table 2). The largest relative declines were in the prevalence of moderate and severe anaemia in pregnant women aged 15–49 years, and in the prevalence of severe anaemia in non-pregnant women aged 15–49 years (relative declines of 12–27% per decade; posterior probability of a true decline >0·99). Trends in mild anaemia were stable in some regions, slightly increased in other regions, and declined in other regions, with little change in the prevalence of mild anaemia globally (posterior probability of an increase in all women aged 15–49 years: 0·76).

In 2000, Yemen, India, Cambodia, Haiti, and 20 countries in west and central Africa had a prevalence of anaemia of more than 50% in women aged 15–49 years (see WHO global anaemia estimates available online). By 2019, prevalence was higher than 50% in ten countries: Yemen, India, Maldives, and seven countries in west and central Africa (from high to low: Mali, Benin, Nigeria, Senegal, Burkina Faso, Gabon, and Côte d’Ivoire). Although anaemia prevalence was higher in pregnant women aged 15–49 years than in non-pregnant women aged 15–49 years in most countries and in most years (171 of 197 countries in 2019), regional patterns in anaemia prevalence were similar (appendix p 28). In a few countries, anaemia prevalence in non-pregnant women aged 15–49 years was higher than that of pregnant women aged 15–49 years; these countries included those with the highest overall anaemia prevalence. In 2019, the absolute difference in total anaemia prevalence between non-pregnant and pregnant women aged 15–49 years was 3% or higher in Yemen, India, the Maldives, Jordan, and Afghanistan (see WHO global anaemia estimates available online).

Regional trends in anaemia prevalence among women aged 15–49 years were similar or somewhat smaller than trends for children aged 6–59 months, with the exception of south Asia: anaemia in children aged 6–59 months declined by 12% per decade (a relative decline; posterior probability of a true decline >0·99), while anaemia in women aged 15–49 years declined by 2% per decade (posterior probability of a true decline: 0·64). In both 2000 and 2019, the prevalence of anaemia was highest in west and central Africa and south Asia, and lowest in high-income countries, with modest changes in prevalence in all three regions (table 2). By contrast, the prevalence of anaemia declined by 21% per decade in Latin America and the Caribbean (posterior probability of true decline >0·99), by 12% per decade in east and southeast Asia (posterior probability of true decline 0·91), and by 12% per decade in east Africa (posterior probability of true decline >0·99). In some regions and globally, progress might have slowed in 2010–19 compared to 2000–09 (appendix p 26).

**Figure 3:**
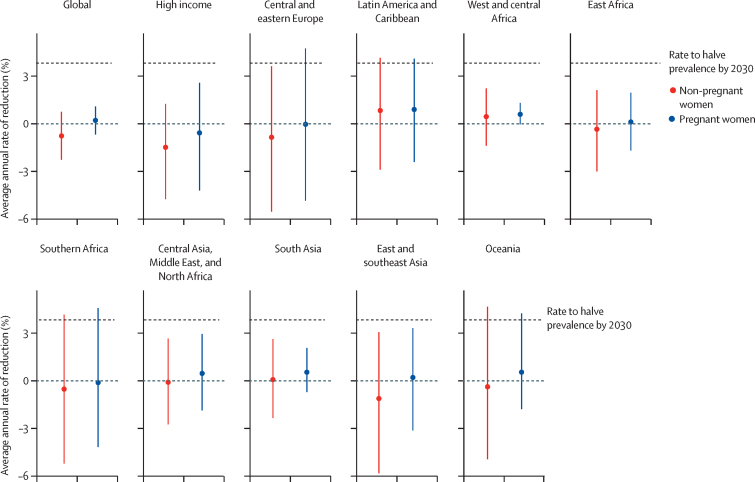
Average annual rate of reduction in anaemia prevalence between 2012 and 2019, globally and by world region and physiological status, for women aged 15–49 years Vertical bars show 95% uncertainty intervals. The horizontal black dashed line shows the required average annual rate of reduction to meet the World Health Assembly anaemia target to reduce anaemia prevalence by 50% by 2030.

Globally and in every region, the average annual rate of reduction in anaemia prevalence in women aged 15–49 years during 2012–19 was well below that needed to meet the WHA target on anaemia (figure 3). If 2012–19 trends continue until 2030, the posterior probability of meeting the WHA target is 0·03 or lower globally and in all regions (appendix pp 19, 20). However, two countries are on track to meet the target: Guatemala (posterior probability of meeting the target if recent trends continue: 0·80) and the Philippines (posterior probability 0·64; appendix p 29). Both countries already had a low prevalence of anaemia in 2012 (11% [95% UI 9–14] in Guatemala and 17% [13–21] in the Philippines). Country-specific data are presented in the online dataset of WHO global anaemia estimates.

## Discussion

Globally, regionally, and in all countries other than Guatemala and the Philippines, the rate of reduction in anaemia in women is insufficient to meet the WHA target to halve anaemia prevalence in women of reproductive age by 2030. Relative reductions in total anaemia prevalence in women aged 15–49 years and children aged 6–59 months have been slow compared to other child, maternal, and nutritional health indicators, which have achieved worldwide average annual reductions of around 2–4% per year since 2000,^16^, ^17^, ^18^ compared to global reductions in total anaemia prevalence of 0–1% per year during the same period. Beneath these global patterns, declines in anaemia prevalence were greater in some populations and time periods, and for more severe anaemia. The Latin America and Caribbean region had the largest relative declines in total anaemia in both women aged 15–49 years and children aged 6–59 months. Declines in anaemia prevalence might have been larger in 2000–09 than in 2010–19. Finally, reductions in moderate and severe anaemia in children aged 6–59 months and pregnant women aged 15–49 years and in severe anaemia in non-pregnant women aged 15–49 years were most marked, while national and regional trends in mild anaemia were small, of mixed direction, and uncertain in all population groups. The relative health consequences of different severity levels of anaemia are an area of active research.^19^

Anaemia can be caused by nutrition-specific factors (eg, due to insufficient intake or poor absorption of micronutrients), non-nutritional factors, or a combination of these; each of these factors has social determinants.^1^, ^2^, ^20^ Non-nutritional causes of anaemia include zoonotic diseases, such as malaria, helminth infection, or schistosomiasis; chronic inflammatory disorders; or genetic haemoglobin disorders. Of nutritional factors, iron deficiency is the most common cause of anaemia. Iron deficiency is present in approximately a quarter to half of children aged 6–59 months and women aged 15–49 years with anaemia, with a possibly lower proportion of anaemia cases associated with iron deficiency in populations with a high burden of anaemia and infections.^21^, ^22^

Isolating the contributions of changes in nutritional and non-nutritional causes of anaemia to regional trends requires detailed trend data on each of these determinants. Population-based data on micronutrient deficiencies are sparse and insufficient to generate reliable national and regional trends in the prevalence of such deficiencies.^23^ The dominant public health response to anaemia has been to provide nutrients, primarily iron, to populations at risk through supplementation, fortification of staple foods, and multiple micronutrient powders. Although these strategies can be beneficial in contexts where micronutrient intakes are inadequate, particularly when supplementation is provided as part of antenatal care,^24^, ^25^ coverage of many of these intervention programmes remains low^26^, ^27^, ^28^, ^29^ and complementary interventions are generally needed to address other causes of anaemia.^30^ The availability of animal-source foods has increased in most low-income and middle-income countries, especially in east Asia.^14^ Reductions in malaria incidence might have contributed to the observed improvements in anaemia prevalence in Africa, especially during 2005–15 when increases in the use of insecticide-treated nets and reductions in malaria incidence were most rapid.^31^ Malaria incidence remains higher in west and central Africa than in other regions,^31^ which might contribute to the high prevalence of anaemia in these regions. Because there are interactions between various causes of anaemia, especially iron status and infections, they should be addressed together.^2^, ^32^

Our estimates extend through 2019, based on data collected through 2020. Of the data collected in 2020, only one source (Rwanda 2019–20 Demographic and Health Survey) completed fieldwork after March, 2020; thus, the estimates do not take into account the COVID-19 pandemic. Pandemic-related disruptions are likely to have reduced coverage of essential interventions and access to nutritious foods.^14^, ^33^, ^34^ Data collection must resume for a full understanding of the effect of the COVID-19 pandemic on anaemia prevalence globally and across different regions and countries.

Recent evidence shows that close-in-time household surveys estimate haemoglobin distributions that differ by more than expected, given sampling and non-sampling errors.^7^, ^35^ Influential factors might include pre-analytical factors such as different types of blood sample (eg, venous or capillary blood) and the quality of the blood sample, as well as analytical methods for measuring blood haemoglobin, or other factors associated with survey design and implementation.^8^ These factors have not been taken into account in this study or in previous global, regional, and country estimates of anaemia prevalence.^3^, ^36^ We tested the influence of accounting for the type of blood sample in our statistical model but excluded the term from our final model because it had little effect on our results (appendix p 12). Further research is needed to determine which factors associated with survey design, implementation, or haemoglobin measurement are responsible for any systematic differences among data sources, so that global models can account for the relevant factors.^37^

These estimates supersede previous global estimates of anaemia prevalence. At the global level, the estimates presented here are similar to our previous estimates,^36^, ^38^ with new estimates for all population groups falling within the uncertainty interval of the previous estimate set. Some regional and country estimates differ because new data were included. The current estimates included 489 data sources spanning 1995–2020, while the estimates for 1995–2016 analysed 357 data sources spanning 1990–2016. The covariates used in the global model were also updated; however, sensitivity analyses showed that this change had a negligible effect on estimates of haemoglobin distribution globally and in countries with primary data (appendix pp 10, 11). The Global Burden of Diseases, Injuries, and Risk Factors Study (GBD) also regularly updates global, regional, and national estimates of anaemia prevalence, using different inclusion and exclusion criteria, statistical models, and time-varying covariates.^3^, ^39^ Nevertheless, the GBD 2019 collaborators estimated a similar prevalence of anaemia in women aged 15–49 years in 2019 (29% [95% UI 28–29] in GBD 2019 *vs* 30% [27–33] in the present study). Their estimates for anaemia prevalence in children younger than 5 years were slightly higher than ours (46% [95% UI 44–48] in GBD 2019 *vs* 40% [36–44] in the present study). This discrepancy can partly, but not entirely, be explained by our exclusion of infants aged 0–5 months, who typically have lower haemoglobin concentrations than older children and are rarely included in population-based surveys. Finally, our finding of poor progress towards the WHA anaemia target is consistent with the conclusions of a study that assessed progress in specific low-income and middle-income countries.^40^

Key strengths of this study include our extensive data search and rigorous criteria for inclusion of sources; an active consultation with WHO Member States, which resulted in identification and inclusion of additional data sources; estimation of trends by country and region; estimation of the full population distributions of haemoglobin, including prevalence of anaemia by severity; and systematic estimation and reporting of uncertainty. The main limitation of our analysis is that, despite the extensive data searches and access to data, there were considerable gaps in data availability. Data were most sparse in the high-income countries, where anaemia prevalence is low. Finally, we were unable to systematically account for the effect of any factors associated with haemoglobin measurement that might affect population estimates.

Reducing anaemia prevalence is recognised as an important component of women's and children's health. Despite the observed improvements, anaemia prevalence remains high in the poorest regions in the world, presenting an obstacle to reducing maternal and neonatal mortality, as well as promoting healthy early childhood development and improving school performance and work productivity.^2^ Further reductions in anaemia prevalence are likely to require a combination of programmes that address the infectious and nutritional determinants of low haemoglobin concentrations. Effectively addressing anaemia in all its forms requires a firm understanding of the unique determinants of anaemia in a particular setting, including by subnational area.^40^ Steps for developing an anaemia-control strategy, along with key actions and recommendations, have been outlined by WHO and others.^1^, ^41^

## Data sharing

Mean haemoglobin and prevalence of anaemia by severity from all data sources, as available, together with relevant metadata, are available online. Following data use agreements, individual-level data must be obtained from data owners.

## Declaration of interests

GAS and CJP report consulting contracts from WHO supporting this work. ME reports receiving a charitable grant from the AstraZeneca Young Health Programme. GAS, FR, and JPW report contracts with the Global Alliance for Improved Nutrition, WHO, and UNICEF. FR and JPW report contracts with Harvest Plus. GAS reports a contract with the World Bank and travel support from UNICEF. PSS reports contracts with the US Centers for Disease Control and Prevention and the Bill & Melinda Gates Foundation. All other authors declare no competing interests.
