# Supplementary material for: National, regional, and global estimates of anaemia by severity in women and children for 2000–19: a pooled analysis of population-representative data
Source: Lancet Glob Health. 2022 Apr 12;10(5):e627–39. doi: 10.1016/S2214-109X(22)00084-5 (PMC9023869; doi:10.1016/S2214-109X(22)00084-5)
Supplement: Supplementary appendix [file mmc1.pdf]

THE LANCET
Global Health

Supplementary appendix

This appendix formed part of the original submission and has been peer reviewed. We post it as supplied by the authors.

Supplement to: Stevens GA, Paciorek CJ, Flores-Urrutia MC, et al. National, regional, and global estimates of anaemia by severity in women and children for 2000–19: a pooled analysis of population-representative data. *Lancet Glob Health* 2022; **10:** e627–39.

CONTENTS

1. Data identification, access and inclusion 2

1.1 Individual-level data 3

1.2 Data accessed as summary statistics 4

1.3 Prevalence of pregnancy 5

2. Accounting for complex survey design 6

3. Adjusting haemoglobin for elevation and smoking 8

4. Bayesian hierarchical mixture model 9

4.1 Overview 9

4.2 Covariate selection 10

4.3 Technical description 12

5. References 15

Table 1 17

Table 2 18

Table 3 19

Table 4 21

Figure 1 23

Figure 2 24

Figure 3 25

Figure 4 26

Figure 5 28

Figure 6 29

Figure 7 30

1. Data identification, access and inclusion

Our data search and access strategy were designed to obtain as many sources as possible while ensuring that the sources were representative of the population at the national level or at least covered three first administrative units within the country. The distribution of blood haemoglobin concentration in a population is commonly summarized as a percentage below a threshold, or a prevalence of anaemia. Mean haemoglobin and its standard deviation may also be reported. Anaemia thresholds typically vary by age, sex, and pregnancy status. Studies may also use different haemoglobin thresholds to define anaemia, and may report multiple anaemia severities, such as mild, moderate and severe anaemia. We accessed data in two forms: 1) anonymised individual-level haemoglobin data when available, and 2) summary statistics, including mean haemoglobin and anaemia prevalences below specific thresholds, from the Micronutrients Database of the WHO Vitamin and Mineral Nutrition Information System (VMNIS) database (see Figure 1 for a flowchart summarizing the process). We used anaemia prevalences with any definition in our statistical model described in Section 4, which accounts for the specific thresholds used to define anaemia when using the data.

We included data sources if:

- blood haemoglobin was measured;
- the study reported anaemia or mean haemoglobin for preschool-age children (6–59 months of age) or women aged 15–49 years;
- a probabilistic sampling method with a defined sampling frame was used and data were representative of at least three first administrative units within a country;
- data were collected in or after 1995;
- data were from 194 Member States or 3 territories: Puerto Rico; China, Province of Taiwan; West Bank and Gaza Strip; and
- standard, validated data collection techniques and laboratory methodologies were used.

Measurement of haemoglobin for children younger than 6 months of age is not common in surveys because it requires a puncture to obtain blood drops. For this reason, estimates were made for children 6–59 months of age. Some data sources did not report anaemia prevalence for the target age ranges. We included data on preschool-age children up to 71 months of age as long as children 6–59 months were included in the sample. For women, we included data sources reporting on any age range 10 years and over as long as women aged 15 to 49 years were included in the sample. Data sources that did not cover the exact age range for children or included women younger or older than 15 to 49 years were given smaller weights, as described in Section 4.

We performed an additional screening if a facility-based sampling scheme was used in order to exclude data where these would not be representative of the general population. The general threshold for inclusion was 80% affiliation of the target population with the facility. For studies of children sampled from primary care physician rosters or well-child visits, we included the data if national coverage of the third dose of DTP vaccine exceeded 80% [1]. For women sampled from obstetric care providers, data were included if the coverage of at least one ANC visit was greater than 80% [2]. For school-based sampling of adolescents, the completion rate of lower secondary school for girls was required to be greater than 80% [3].

In four countries where migrants comprised more than 40% of the population in the country (Kuwait, Qatar, United Arab Emirates, and Singapore), we excluded any data that covered only national citizens [4]. We also excluded subnational data sources if the subnational area was selected on a variable causally related to anaemia prevalence, e.g., malaria endemicity.

A country consultation was carried out in December 2020 with national focal points. Focal points were consulted on draft estimates of mean haemoglobin, prevalence of anaemia and prevalence of severe anaemia in children 6-59 months of age and women 15 to 49 years of age (estimates by pregnancy status and combined estimates), as well as on the input data (i.e., primary data such as household surveys) that were used to compute the anaemia estimates for each demographic group in each country. Countries were asked to share data sources not included in the draft input data series (e.g., published or unpublished nationally representative surveys or peer-reviewed publications with national level data). We included all data provided by national governments in the WHO Micronutrients Database (see section 1.2) and used them to compute final estimates provided that they fulfilled inclusion criteria. We manually identified and removed duplicated data accessed from more than one source. Our dataset closed in February 2021.

1.1 Individual-level data

We obtained anonymised individual-level data from health-examination surveys and household surveys with haemoglobin measurements. Most of these sources were multi-country surveys including the Demographic and Health Surveys (DHS), Multiple Indicator Cluster Surveys (MICS), Reproductive Health Surveys (RHS) and the Malaria Indicator Surveys (MIS). We also used national health examination survey data when the data were available to us and it was not possible to obtain summary statistics for the source. From each source, we extracted the following variables: age, sex, haemoglobin concentration, pregnancy status, elevation (if available), and survey sample weight, stratum, and primary sampling unit.

We only used data for children aged 6–59 months and women aged 15–49 years. Haemoglobin concentrations recorded in survey datasets that were considered biologically implausible were excluded; i.e., haemoglobin measurements that were less than 25 g/L or greater than 200 g/L. A histogram of haemoglobin concentrations was visually screened for each population-survey combination to identify any data anomalies. Finally, we adjusted all haemoglobin data for elevation, as described in Section 3.

We excluded all individual-record observations for women who reported that they did not know their pregnancy status. Five surveys with individual-level data did not record pregnancy status as a part of their design. In these cases, we accessed the data directly as summary statistics, as described below.

1.2 Data accessed as summary statistics

WHO maintains a Micronutrients Database, part of VMNIS, which contains haemoglobin concentrations and anaemia prevalence [5]. Data are identified via periodic MEDLINE searches and an international network of collaborators, who uncover data sources not reported in bibliographic databases. In order to obtain up-to-date anaemia data, a search on bibliographic databases[1] was performed. The search was limited to humans, and the following search terms were used:

(((national) AND (survey)) OR ((population) AND (prevalence))) AND ((iron status) OR (iron deficiency) OR (anemia) OR (anaemia) OR (hemoglobin) OR (haemoglobin) OR (low iron level) OR (transferrin receptor) OR (ferritin) OR (insufficient iron))

Studies are included in the WHO Micronutrients Database if there is a defined population-based sampling frame, a probabilistic sampling procedure is used and standard, validated data collection techniques and laboratory methodologies were used. For inclusion of these summary data in the analysis, we screened these summary data using our exclusion criteria. Consistent with our inclusion and exclusion criteria, we excluded summarized data sources if:

- data were collected prior to 1995;
- we had access to the same data as individual-level records;
- they were representative of only one or two first administrative level(s) or of only urban or rural areas;

---

[1] Bibliographic databases used to identify anaemia data sources: MEDLINE, EMBASE, Web of Science, CINAHL, AGRICOLA, IBECS, SCIELO, LILACS, AIM (AFRO), IMEMR (EMRO), PAHO, WHOLIS, WPRIM (WPRO), IMSEAR, Native Health Research Database

- the study did not report data on haemoglobin concentration in children aged 6–59 months or women aged 15–49 years;
- only aggregated summary statistics that included subjects beyond an acceptable age interval or sex (i.e., data on preschool children included children over 6 years of age or data on women included males);
- we were unable to determine whether the data were adjusted for elevation and the data were collected in a high-elevation country;
- non-random sampling methods were used, or sampling methods were not adequately described; or
- a facility-based surveillance method was used in a country where facility affiliation was lower than 80% (as described above).

In some cases, the sample size was not reported in the WHO Micronutrients Database. In that case, we conservatively assumed a sample size of 100. For four data sources, we had some information that sample sizes were substantially larger than 100, such as number of households sampled or all-age-sex sample size, which we used to make a conservative estimate of sample size (India 2019-2020 National Family Health Survey – Phase 1, Indonesia RISKESDAS 2013, Pakistan National Nutrition Survey 2018, and China Chronic Diseases and Nutritional Health Surveillance 2015).

We inspected scatterplots of mean haemoglobin vs. prevalence of anaemia, moderate anaemia, and severe anaemia, as well as total anaemia vs. moderate and severe anaemia, and inspected plots of each country's data by year (e.g., appendix Figure 7). We excluded the following data sources that reported anaemia prevalence, mean haemoglobin or haemoglobin distribution which we considered implausible: Enquète nationale sur les indicateurs du paludisme au Tchad 2010 (ENIPT-2010), and Enquète nationale sur les indicateurs du paludisme au Tchad de 2017 (ENIPT-2017), 2005 Rwanda DHS, 2007 Jordan DHS, and 1998-1999 Bahrain National Nutrition Survey. We also excluded preschool children's mean haemoglobin from the Iraq national micronutrient deficiencies: assessment and response 2011-2012 and prevalence of anaemia in pregnant women reported by the Afghanistan National Nutrition Survey 2004 because we considered the values to be implausible. We excluded any population standard deviation below 5 g/L, as these are likely mislabelled standard errors. Finally, we included summary statistics from 4 preliminary DHS reports which were not yet included in the WHO Micronutrients Database.

1.3 Prevalence of pregnancy

As described below, the statistical model used separate data for pregnant and non-pregnant women when data were available by pregnancy status, and it accounted for the proportion of the sample that was pregnant in sources that reported combined summary statistics. We then made estimates by pregnancy

status and combined these to estimate total prevalence of anaemia in women aged 15–49 years. Both of these steps required national estimates of the percentage of women who are pregnant.

In our prior modelling exercise, we found that women are not very likely to report early pregnancies, with reporting of pregnancies becoming consistent after the 10th week of pregnancy. This early pregnancy period is also the period during which the decline in haemoglobin concentrations is steepest [6]. To be consistent with the reporting behaviours and to restrict pregnancy to periods when haemoglobin concentrations are consistently low, our operational definition of pregnancy was restricted to after 8 weeks of gestation. We used an average pregnancy duration of 32 weeks (gestational weeks 8-40) to calculate the proportion of women in each country and year who were pregnant at any time. To calculate this quantity, we used country- and age-specific data on live births from the UN Population Division's 2019 Revision of the World Population Prospects and an estimate of the number of stillbirths, together with estimates of the total number of women aged 15–49 years[7,8].

2. Accounting for complex survey design

As described in Section 4, our statistical model used individual-level data when available and summary statistics when not to estimate the full distributions of blood haemoglobin concentration by country and year.

All individual-level data in the analysis came from surveys that used complex survey designs. Specifically, in designing a representative survey, the target populations were usually divided into strata based on geographical regions within the country, whether place of residence was rural or urban, and/or the socio-economic characteristics of the place of residence; within each stratum, a number of clusters were randomly selected. Clusters may be villages, administrative units, or census units. Households or participants were then randomly sampled within each cluster. Depending on survey design, some individuals or households may have a higher probability of being selected than others. To account for the differences in probability of being sampled, each observation is assigned a sample weight. These weights are calculated to make the survey data representative of the total population.

An implication of the sampling method is that the so-called effective sample size of the survey (ESS) is different from its actual sample size. This occurs primarily because the sampled individuals representative but are not randomly sampled from the entire country, and hence contain less information than they would, had they been a true simple random sample of the population.

To reflect the true availability of information in each survey and in the individual level data that it provided to the statistical model, we estimated ESS using the "*estat effects*" command of the Stata version 16.1 svy suite of commands (StataCorp, 2019). In particular, this command generates the design effect (DEFF), which is the ratio between the (usually smaller) ESS and the real sample size, e.g., a survey with 1000 subjects with a DEFF of 2.0 has an ESS of 500. The DEFF may differ by summary statistic metrics (mean vs. prevalence below 100 g/L vs. prevalence below 120 g/L) depending on how these indicators are distributed across the strata and clusters. For each survey, we calculated the DEFF as the median of those from a range of metrics, specifically, mean haemoglobin concentration and prevalence below 90, 100, 110, 120, 130 g/L. ESS was then calculated as sample size divided by DEFF.

In our statistical model, we accounted for the difference between the real and effective sample sizes and for the difference in weights for each observation by scaling the weights across all observations in a study to sum to the ESS. These scaled weights were then used to weight the likelihood contributions from each individual. In addition, surveys may over- or under-sample pregnant women relative to their fraction of the population. To account for this imbalance in the statistical model for women, we scaled the weights for each individual such that the sum of the weights for pregnant women was equal to the total ESS for the study multiplied by the proportion of pregnant women in the study; we did the same for non-pregnant women. This ensured that the sum of weights across all women was equal to the ESS for the study and that the relative weighting of pregnant and non-pregnant women reflected the number of women in each category in the study.

Data sources providing only summary statistics were also predominantly from surveys that used complex survey designs, but sample sizes recorded for these data sources are actual sample sizes and not the effective sample sizes. To ensure that the sample sizes used for these sources in the statistical modelling also reflect the complex survey design, we estimated ESS for each study as the actual sample size multiplied by an estimate of the DEFF. Calculating the DEFF requires individual-level data, which by definition are not available for these data sources. We used the median DEFF from all surveys with individual-level data. We then used the estimated ESS for each study to compute the statistical uncertainty associated with each study.

3. Adjusting haemoglobin for elevation and smoking

Haemoglobin needs are greater for those living at high elevations due to the lower concentration of oxygen in the atmosphere [9]. To reflect the health and functional consequences of low haemoglobin in a comparable manner, data adjusted for altitude were used if reported. For individual-level data, when haemoglobin data were not adjusted for elevation and elevation measurements corresponding to individual-level observations were available, we adjusted haemoglobin concentrations using a formula recommended by WHO [9] and commonly used in studies worldwide:

$$Hb_{adjusted} = Hb_{unadjusted} + 0.32 *(elevation * 0.0033) - 0.22 *(elevation *. 0.0033)^2 \quad (1)$$

where haemoglobin is measured in g/L and elevation is measured in meters above sea level (m.a.s.l.). The adjustment is only applied to individuals living at elevations over 1000 m.a.s.l.

We were unable to obtain elevation information for individual subjects for some surveys with individual record data. When the proportion of population living at elevations above 1500 m.a.s.l. (an elevation at which there is 3 g/L effect on haemoglobin concentration) was less than 5% of total population (hereafter termed low-elevation countries), we included the source without adjusting haemoglobin for elevation. Data from individual-record data sources without individual-level elevation in a country where more than 5% of total population lived above 1500 m.a.s.l. were converted to summary statistics and adjusted as described below.

For data available as only summary statistics, we determined whether the data were from a low-elevation country. If data were from a low-elevation country, we used the data regardless of adjustment for elevation (328 data sources). If data were not from a low-elevation country, we classified the data as adjusted for altitude, unadjusted or unknown adjustment. We used data adjusted for altitude and excluded data where we could not determine whether they were adjusted for altitude (Figure 1). We developed regression equations to correct unadjusted summary statistics from high-elevation countries. We pooled data from 98 DHS surveys in countries with some population living over 1000 m.a.s.l. We extracted both adjusted and unadjusted haemoglobin data for all survey participants in the target populations (women aged 15–49 years and children aged 6–59 months). We calculated mean haemoglobin and prevalence of anaemia (using each of the cut-offs 70, 100, 110, 120, and 130 g/L) for women and children using both adjusted and unadjusted data from each of the surveys. We then related elevation-adjusted mean haemoglobin concentration and prevalence of anaemia to the corresponding unadjusted values using separate regressions for each of the six metrics and for each population group, for a total of 12 regressions. For each metric, we regressed the

adjusted values against the unadjusted values, including as additional covariates the percent of population living over 1000 m.a.s.l., the percent of population living over 2000 m.a.s.l., and an interaction between the two. This regression specification was intended to mimic the quadratic relationship in the CDC adjustment (Eq. 1). In the regression for mean haemoglobin, we fixed the coefficient of unadjusted haemoglobin at one to reflect the relationship in (Eq. 1).

We used these regression relationships to predict adjusted mean haemoglobin concentration and adjusted prevalence of anaemia based on unadjusted summary statistics from high-elevation countries (22 data sources). We accounted for uncertainty of this step by calculating the standard regression prediction variance, which reflects both uncertainty in estimating the regression relationship and variability of individual values around the regression line. This variability from the effect of predicting adjusted country-level metrics was then included in the statistical modelling as an added variance in the likelihood for each summary statistic from these sources.

We used data adjusted for smoking status if available but did not make any post-adjustment to data that were not adjusted for smoking status, because the effects of smoking (~0.3 g/L) are substantially smaller than those of elevation (~8 g/L for living over 2000 m.a.s.l.).

4. Bayesian hierarchical mixture model

4.1 Overview

Our aim was to estimate the complete distributions of blood haemoglobin for every country and year, which would then allow calculating any relevant summary statistic. This approach allows making coherent inference on mean haemoglobin and on the prevalence of anaemia at all levels of severity. All analyses were done separately for children and women.

The statistical methods are described in detail in a previous publication [10]. In brief, we used a Bayesian hierarchical mixture model, which uses all available data to make estimates for each country-year. In the hierarchical model, countries are organized into regions that have similar epidemiological and climatological profiles (Table 1). Estimates for each country-year were informed by data from that country-year itself, if available, and by data from other years in the same country and in other countries, especially those in the same region with data in similar time periods. The hierarchical model shares information to a greater degree where data are non-existent or weakly informative (i.e., have large uncertainty), and a lesser degree in data-rich countries and regions. We modelled trends over time as a linear trend plus a smooth nonlinear trend, at

the country, regional, and global levels. The estimates are also informed by covariates that help predict haemoglobin levels. The model included a variance term that accounted for unobserved design factors (sample design, season, haemoglobin measurement method, etc.) that lead to additional variability in the data beyond that expected due to sample size. Finally, the model accounted for the fact that subnational data and data that do not exactly cover the age ranges of interest may have larger variation than national data and data that exactly cover the age ranges of interest, respectively. We fitted the model to data from 1995 to 2020 to limit boundary effects but report results between 2000 and 2019, because there were fewer sources between 1995 and 1999 and in 2020.

The mixture model uses a mixture (a weighted average) of multiple normal ("bell-shaped") densities to estimate the full haemoglobin distributions, which may themselves be skewed. We used a mixture of five normal distributions for children. For adult women, we used two five-component mixtures, one for pregnant women and another for non-pregnant women together in one model. This approach uses all data sources – those that separate pregnant and non-pregnant women, those in which pregnant and non-pregnant women are reported together, and those in which only one group was measured – to make separate estimates by pregnancy status. The differences in haemoglobin distributions between pregnant and non-pregnant women were allowed to vary by country and year. In years and countries where separate data by pregnancy status were lacking, the difference was informed based on other sources, especially those in the same region with data in similar time periods.

The uncertainties of our estimates incorporated sampling error in each data source; non-sampling error of national data (e.g., because of issues in sample design and measurement); additional error associated with subnational data; uncertainty due to elevation adjustments; and uncertainty due to making estimates by country and year when data were missing altogether, when only summary statistics (vs. individual-level data) were available, or when data were not available separately by pregnancy status.

4.2 Covariate selection

Within the framework of the model described above, we reviewed and revised the set of time-varying covariates that were used to inform the model. Previous editions of WHO haemoglobin estimates used the following time-varying covariates to inform estimates: mean years of maternal education, proportion of population in urban areas, mean latitude, prevalence of sickle-cell disorders and thalassemias, mean body-mass index for women, and mean weight-for-age Z score for children. Some of these datasets are no longer updated, including prevalence of sickle-cell disorders and thalassemias and mean weight-for-age Z score in children, while new covariate datasets have become available. We sought candidate covariates that were

distal determinants of anaemia (i.e., indicators of socio-economic status such as education levels or wealth), proximal determinants (e.g., measures of iron intake or malaria prevalence, including food balance data, climate data, and supplementation/fortification data), or health outcomes that are correlated with haemoglobin concentrations (e.g., body mass index or weight for age Z-score). We considered covariates prepared for the GBD2019 study [11] or by the UN system. Because the goal of including country covariates is to improve model estimates when haemoglobin data are sparse, inconsistent or missing altogether, we excluded country covariates for which primary data are scarce. We reviewed plots of covariate values against year for each country to assess plausibility.

We tested three covariate sets. Set 0 excluded time-varying covariates. Set 1 approximated covariates used in prior estimation rounds while accounting for current data availability, excluding prevalence of haemoglobinopathies and replacing mean weight-for-age Z score with log of shock-free child mortality [12] for children, since the former two datasets are no longer updated. Shock-free child mortality was selected because (a) child mortality has similar infectious and nutritional risk factors as low haemoglobin, and (b) anaemia would not be expected to fluctuate in response to natural disasters or wars (that cause mortality shocks) to the same extent as child mortality. Set 2 included socio-demographic index [11], meat supply (kcal/capita) [13,14], mean BMI (only for women) [15], and log of shock-free under-five mortality (only for children). For all covariates that did not contain estimates for recent years, we held the most recent covariate value constant through 2020. We aimed to select our final covariate set prior to carrying out our consultation with national ministries of health, and therefore tested the covariate sets on a near-final database of primary haemoglobin data (i.e., the dataset did not include data provided by the ministries of health during the consultation). We compared Watanabe-Akaike information criterion (WAIC) – a measure of model fit – across the three covariate sets [16]. How to implement WAIC in this complicated setting with temporal and spatial correlation is not well-defined, and there is some uncertainty in estimating WAIC, so we consider the WAIC results to be one piece of evidence and not conclusive. We also compared the fitted estimates using each of the three covariate sets and compared the estimates to primary data where the estimates differed. For the women's model, WAIC was inconclusive; for the children's model, WAIC indicated that including covariates was preferred to a model with no time-varying covariates. Comparing fitted estimates using each of the three covariate sets, estimated prevalence of anaemia and severe anaemia and mean haemoglobin were similar at the global and in countries with primary data available. These indicators were also similar at the region level, with the exception of regions with extremely sparse data (specifically, Oceania and Central and Eastern Europe). Therefore, we chose the covariate set based on our assessment of plausibility of estimates in countries with no, sparse or inconsistent data. We considered the estimates generated using covariate set 2 to be most plausible.

There is increasing concern that factors associated with haemoglobin measurement can have a substantial effect on the estimated prevalence of anaemia [17–19]. Factors that may influence haemoglobin measurements include different types of blood sample (e.g., venous or capillary blood) and analytical methods for measuring blood haemoglobin in household surveys. We tested an offset for blood collection by capillary puncture in the global estimation model. This approach carries some risk. Capillary puncture is more likely to be used in some settings (less wealthy countries) and time periods (more recent years). The fitted coefficient for the offset may reflect residual regional or time differences, rather than any systematic bias of capillary blood collection. We ran both the models for children and women with an offset for capillary blood collection. The capillary offset was estimated to be 1 g/L for pre-school children (i.e., the model estimated that capillary blood collection underestimates haemoglobin by 1 g/L), and -1.5 g/L for women (capillary blood collection overestimates haemoglobin by 1.5 g/L). WAIC indicates that including the capillary offset makes the fits worse for children, but improves model fit for women. The offset estimated in the model was smaller than expected for children (expected value of around 5 g/L) based on comparison of near-in-time survey pairs with different blood collection types. We suspect that the offset coefficient in the children's model may reflect residual differences in region/time period that were not explained by the other terms in the regression model. For women, the offset was estimated to be small, as expected. Inclusion of the offset also required exclusion of data sources that did not report type of blood collection. Therefore, we chose not to include the offset in the final global estimation model for children. In the interest of fitting a consistent and parsimonious model for women, we also excluded the offset from the women's model.

4.3 Technical description

The model is specified as follows, with *g* an indicator differentiating pregnant and non-pregnant strata within a study:

$$f_{gi}(z) = \sum_{m=1}^{M+1} w_{mgi}\, \mathcal{N}\left(z \,|\, \theta_m, \sigma_m^2\right) \quad (2)$$

$$w_{mgi} = \begin{cases} \Phi(\alpha_{mgi}) \prod_{u=1}^{m-1}(1-\Phi(\alpha_{ugi})) & \text{if } m \leq M \\ \prod_{u=1}^{M}(1-\Phi(\alpha_{mgi})) & \text{if } m = M+1 \end{cases} \quad (3)$$

$$\alpha_{mgi} = \delta^c_{mj[i]} + (\varphi\delta^c)_{mj[i]}t_i + u_{mj[i]t_i} + \beta_m x_i + a_{mi} + b_{mi} + I_{gi}(\gamma^c_{mj[i]} + (\varphi\gamma^c)_{mj[i]}t_i + c_{mi}) \quad (4)$$

Details on the model specifications and features are provided elsewhere [10]. Briefly, equation 2 describes a finite mixture of *M* + 1 normal (*N*) distributions (or mixture components), where the weights (ω) on the constituent normal distributions vary across studies. We specified a probit stick-breaking model for the ω's

in equation 3. This transformation uses the standard normal cumulative distribution function (Φ) to transform α's that range between - ∞ and ∞ to ω's that range between 0 and 1. Specifically, the α's determine the relative weights assigned to each cluster in the following manner: starting with a 'stick' of length one, Φ (α_{1gi}) is the proportion of the stick that we break off and assign to ω_{1gi}; Φ (α_{2gi}) is the proportion of the remaining stick of length (1— ω_{1gi}) that we break off and allocate to ω_{2i}; and so on. Larger values of α_{mgi} thus correspond to higher weights on the mth mixture component for stratum g in study i. The probit stick-breaking transformation therefore allows placing a flexible model on the α's, while ensuring that the ω's still add to one, in such a way that large mass in one part of the haemoglobin distribution is balanced by smaller mass in other parts, and vice versa, through exchanges among the constituent mixture components.

In equation 4, α_{mgi} is defined to leverage all available information in making estimates for each country-year-stratum. $\delta^c_{mj[i]}$ is a country-by-component interaction term, determining the baseline weight placed on each of the M + 1 normal distributions in country j. $(\varphi\delta^c)_{mj[i]}$ is a country- and component-specific linear time effect, determining the linear parts of country j's time trend. Letting T = 26 be the total number of analysis years (1995, 1996,… , 2020), the T-vector $u_{mj[i]}$ captures smooth nonlinear change over time in country j and mixture component m. b_m is the effect of time-varying country-level covariates x (described above) in mixture component m. The a's are study-specific random effects, and the b's capture the extra variance of studies that included women under age 15 or over age 50 (or those that did not cover exactly 6-59 months of age in the model for children). The difference between the models for women and children is that for the former, the model includes the additional terms that are multiplied by I_{gi}, which is an indicator variable that takes the value one when stratum g contains pregnant women and -1 when stratum g contains non-pregnant women.

This indicator multiplies a country- and component-specific term, $\gamma^c_{mj[i]}$, that quantifies the overall difference between pregnant and non-pregnant women, a linear time effect for the pregnant/non-pregnant difference, $(\varphi\gamma^c)_{mj[i]}$, and study-specific errors, c_{mi}, in the difference. The difference in haemoglobin between pregnant and non-pregnant women was modelled as linear for simplicity and because there are insufficient data to reliably estimate more complex trends in difference.

The hierarchical prior distributions for the country-specific terms and specifications of the study-specific error terms are described in detail elsewhere [10], with the additional terms introduced here, $\gamma^c_{mj[i]}$, $(\varphi\gamma^c)_{mj[i]}$ and c_{mi}, treated analogously to δ^c_{mj}, $(\varphi\delta^c)_{mj}$ and a_{mi}, respectively.

For data accessed as summary statistics for which pregnant and non-pregnant women are not distinguished, we took the mixture densities for pregnant and for non-pregnant women and combined them into a $(2M + 2)$-component mixture, weighting by the proportion of pregnant women estimated for that country-year, as described earlier.

The model was previously evaluated by cross-validation to simulate several missingness situations, as described in reference [10]. The median absolute prediction error was smaller than median difference between two surveys carried out in the same country and year, indicating good model performance.

We fitted the Bayesian model using the Markov chain Monte Carlo (MCMC) algorithm and obtained 3000 samples from the parameters' posterior, in turn used to obtain 3000 posterior samples of the population haemoglobin distributions for each country-year. With each of the 3000 sampled distributions we calculated the population haemoglobin mean, and total anaemia, mild anaemia, moderate anaemia, and severe anaemia prevalences for each country-year. Numbers of persons affected by anaemia were computed for each sample by multiplying county-year-age-sex-group population totals from the UN Population Division's 2019 Revision of the World Population Prospects by the prevalence anaemia [8]. Means and prevalence by severity for regions and the world were calculated as population-weighted averages of those of the constituent countries. Estimates for all Latin America and Caribbean regions were presented together. All reported uncertainty intervals are 95% Bayesian credible intervals, computed as the 2.5th-97.5th percentiles of these 3000 draws.

Average changes in mean or prevalence were calculated over the 20 reporting years (2000–2019) and for each decade (2000–2009 and 2010–2019) (absolute for mean and proportional for prevalence). Changes in prevalence measures were computed by fitting the following regression on time in years, separately for each draw, type of anaemia by severity, and time period:

$$\ln(prev) = \alpha + \beta * year \qquad (5)$$

The relative change in prevalence per decade is computed as 100*exp(10* β), and uncertainty intervals were computed as described above. We report the posterior probability (PP) that an estimated increase/decrease represents a truly increasing/decreasing trend as the percentage of draws for which β is greater than/less than 0. To assess progress toward global goals, we computed the average annual rate of reduction (AARR) during 2012–2019 and the posterior probability of meeting the target on anaemia reduction assuming trends 2012–2019 continue. To compute the AARR, regression equation 5 is fitted on total anaemia prevalence in WRA, PW, and NPW estimates for 2012–2019, separately for each population and draw. AARR is computed as 100*(1-exp(β)).

Statistical code is available from the authors upon request (nutrition@who.int).

5. References

1 World Health Organization. Diptheria tetanus toxoid and pertussis (DTP3) immunization coverage among 1-year-olds (%).2020. https://www.who.int/data/gho/data/indicators/indicator-details/GHO/diphtheria-tetanus-toxoid-and-pertussis-(dtp3)-immunization-coverage-among-1-year-olds-(-).

2 United Nations Children's Emergency Fund. UNICEF maternal and newborn health coverage database. 2020. https://data.unicef.org/topic/maternal-health/antenatal-care/.

3 UNESCO Institute for Statistics. Lower secondary completion rate, female (% of relevant age group). 2020. https://data.worldbank.org/indicator/SE.SEC.CMPT.LO.FE.ZS.

4 United Nations Department of Economic and Social Affairs. International Migrant Stock 2019 (United Nations database, POP/DB/MIG/Stock/Rev.2019). 2019.

5 World Health Organization. Micronutrients database. 2021. https://www.who.int/vmnis/database/en/ (accessed March 17, 2021).

6 Stevens GA, Finucane MM, De-Regil LM, *et al.* Global, regional, and national trends in haemoglobin concentration and prevalence of total and severe anaemia in children and pregnant and non-pregnant women for 1995-2011: A systematic analysis of population-representative data. *Lancet Glob Heal* 2013; **1**: e16-25.

7 Blencowe H, Cousens S, Jassir FB, *et al.* National, regional, and worldwide estimates of stillbirth rates in 2015, with trends from 2000: A systematic analysis. *Lancet Glob Heal* 2016; **4**: e98–108.

8 United Nations Department of Economic and Social Affairs. World Population Prospects 2019. 2019. https://population.un.org/wpp/ (accessed March 17, 2021).

9 World Health Organization. Haemoglobin concentrations for the diagnosis of anaemia and assessment of severity. Geneva, 2011 https://apps.who.int/iris/handle/10665/85839 (accessed Oct 12, 2021).

10 Finucane M, Paciorek C, Stevens G, Ezzati M. Semiparametric Bayesian density estimation with disparate data sources: a meta-analysis of global childhood undernutrition. *J Am Stat Assoc* 2015; **110**: 889–901.

11 Global Burden of Disease Collaborative Network. Global Burden of Disease 2019 (GBD 2019) Covariates 1980-2019. 2020.

12 United Nations Inter-Agency Group for Child Mortality Estimation (UN IGME). Levels & Trends in Child Mortality: Report 2020, Estimates developed by the United Nations Inter-agency Group for Child Mortality Estimation. New York, 2020 https://childmortality.org/wp-content/uploads/2020/09/UNICEF-2020-Child-Mortality-Report.pdf (accessed Oct 12, 2021).

13 FAOSTAT. Food Balance Sheets. 2019. http://www.fao.org/faostat/en/#data/FBS (accessed Sept 3, 2020).

14 Bentham J, Singh GM, Danaei G, *et al.* Multidimensional characterization of global food supply from 1961 to 2013. *Nat Food* 2020; **1**: 70–5.

15 Bixby H, Bentham J, Zhou B, *et al.* Rising rural body-mass index is the main driver of the global obesity epidemic in adults. *Nature* 2019; **569**: 260–4.

16 Vehtari A, Gelman A, Gabry J. Practical Bayesian model evaluation using leave-one-out cross-validation and WAIC. *Stat Comput* 2017; **27**: 1413–32.

17 Rappaport AI, Karakochuk CD, Hess SY, *et al.* Variability in haemoglobin concentration by measurement tool and blood source: An analysis from seven countries. *J Clin Pathol* 2020; published online Oct 6. DOI:10.1136/jclinpath-2020-206717.

18 Hruschka DJ, Williams AM, Mei Z, *et al.* Comparing hemoglobin distributions between population-

based surveys matched by country and time. *BMC Public Health* 2020; **20**: 1–10.
19 SPRING. Anemia Assessment in Micronutrient and Demographic and Health Surveys: Comparisons in Malawi and Guatemala. Arlington, VA, 2018.

Table 1. Countries and territories for which estimates were made, and the regions to which they were assigned.

Region	Countries
High-income countries	Andorra, Australia, Austria, Belgium, Brunei Darussalam, Canada, Cyprus, Denmark, Finland, France, Germany, Greece, Iceland, Ireland, Israel, Italy, Japan, Luxembourg, Malta, Monaco, Netherlands, New Zealand, Norway, Portugal, Republic of Korea, San Marino, Singapore, Spain, Sweden, Switzerland, United Kingdom, United States of America
Central and Eastern Europe	Albania, Belarus, Bosnia and Herzegovina, Bulgaria, Croatia, Czechia, Estonia, Hungary, Latvia, Lithuania, Montenegro, North Macedonia, Poland, Republic of Moldova, Romania, Russian Federation, Serbia, Slovakia, Slovenia, Ukraine
Central and West Africa	Angola, Benin, Burkina Faso, Cabo Verde, Cameroon, Central African Republic, Chad, Congo, Cote d'Ivoire, Democratic Republic of the Congo, Equatorial Guinea, Gabon, Gambia, Ghana, Guinea, Guinea-Bissau, Liberia, Mali, Mauritania, Niger, Nigeria, Sao Tome and Principe, Senegal, Sierra Leone, Togo
Central Asia, Middle East, and North Africa	Algeria, Armenia, Azerbaijan, Bahrain, Egypt, Georgia, Iran (Islamic Republic of), Iraq, Jordan, Kazakhstan, Kuwait, Kyrgyzstan, Lebanon, Libya, Mongolia, Morocco, , Oman, Qatar, Saudi Arabia, Sudan, Syrian Arab Republic, Tajikistan, Tunisia, Turkey, Turkmenistan, United Arab Emirates, Uzbekistan, West Bank and Gaza Strip, Yemen
South Asia	Afghanistan, Bangladesh, Bhutan, India, Nepal, Pakistan
East and Southeast Asia	Cambodia, China, Democratic People's Republic of Korea, Indonesia, Lao People's Democratic Republic, Malaysia, Maldives, Myanmar, Philippines, Sri Lanka, China, Province of Taiwan, Thailand, Timor-Leste, Viet Nam
Southern Latin America	Argentina, Chile, Uruguay
Andean, Central, and Tropical Latin America and Caribbean	Antigua and Barbuda, Bahamas, Barbados, Belize, Bolivia (Plurinational State of), Brazil, Colombia, Costa Rica, Cuba, Dominica, Dominican Republic, Ecuador, El Salvador, Grenada, Guatemala, Guyana, Haiti, Honduras, Jamaica, Mexico, Nicaragua, Panama, Paraguay, Peru, Puerto Rico, Saint Kitts and Nevis, Saint Lucia, Saint Vincent and the Grenadines, Suriname, Trinidad and Tobago, Venezuela (Bolivarian Republic of)
Oceania	Cook Islands, Fiji, Kiribati, Marshall Islands, Micronesia (Federated States of), Nauru, Niue, Palau, Papua New Guinea, Samoa, Solomon Islands, Tonga, Tuvalu, Vanuatu
East Africa	Burundi, Comoros, Djibouti, Eritrea, Ethiopia, Kenya, Madagascar, Malawi, Mauritius, Mozambique, Rwanda, Seychelles, Somalia, South Sudan, Uganda, United Republic of Tanzania, Zambia
Southern Africa	Botswana, Eswatini, Lesotho, Namibia, South Africa, Zimbabwe

Table 2. Included data sources by population, haemoglobin measurement method and type of blood sample.

		Sample collection method							
		Capillary puncture		Venipuncture		Not specified		Total	
		n	%	n	%	n	%	n	%
**Haemoglobin measurement method**	*Children aged 6–59 months*								
	HemoCue®	278	71%	45	11%	8	2%	331	84%
	Clinical analyzer (chemical based)	0	0%	26	7%	1	0%	27	7%
	Cyanmethaemoglobin	8	2%	11	3%	1	0%	20	5%
	Not specified	1	0%	10	3%	4	1%	15	4%
	Total	287	73%	92	23%	14	4%	393	100%
	Women aged 15-49 years								
	HemoCue®	231	57%	43	11%	10	2%	284	70%
	Clinical analyzer (chemical based)	0	0%	62	15%	2	0%	64	16%
	Cyanmethaemoglobin	11	3%	14	3%	1	0%	26	6%
	Not specified	1	0%	22	5%	11	3%	34	8%
	Total	243	60%	141	35%	24	6%	408	100%

Table 3. Mean haemoglobin by world region in 2000 and 2019, and posterior probability of meeting the target of halving anaemia prevalence by 2030 (baseline year 2012) if trends 2012-2019 continue to 2030.

	**Mean haemoglobin (g/L)**		**Posterior probability of meeting anaemia target**
**Region**	**2000**	**2019**	
Children aged 6-59 months			
High Income	123 (121, 124)	122 (120, 124)	
Eastern and Central Europe	117 (113, 120)	119 (114, 122)	
East and Southeast Asia	115 (111, 119)	118 (114, 122)	
South Asia	102 (100, 104)	108 (105, 112)	
Oceania	109 (104, 114)	110 (104, 117)	
Central Asia, Middle East, and North Africa	111 (109, 113)	114 (112, 117)	
West and Central Africa	96 (94, 98)	102 (100, 104)	
East Africa	100 (98, 103)	107 (105, 110)	
Southern Africa	112 (106, 117)	111 (106, 117)	
Latin America and Caribbean	116 (114, 118)	120 (118, 123)	
Globe	*109 (108, 110)*	*112 (111, 114)*	
Non-pregnant women aged 15-49 years			
High Income	132 (131, 133)	131 (130, 132)	
Eastern and Central Europe	130 (126, 133)	129 (125, 133)	
East and Southeast Asia	128 (126, 131)	131 (128, 133)	
South Asia	118 (115, 120)	119 (116, 121)	
Oceania	125 (121, 130)	125 (119, 131)	
Central Asia, Middle East, and North Africa	123 (122, 125)	125 (123, 127)	
West and Central Africa	117 (114, 119)	119 (117, 121)	
East Africa	124 (122, 126)	126 (124, 128)	
Southern Africa	124 (121, 128)	127 (123, 131)	
Latin America and Caribbean	128 (126, 130)	131 (129, 134)	
Globe	*126 (125, 127)*	*126 (125, 127)*	
Pregnant women aged 15-49 years			
High Income	121 (119, 123)	121 (118, 123)	
Eastern and Central Europe	118 (113, 123)	119 (114, 124)	
East and Southeast Asia	116 (114, 119)	118 (115, 122)	
South Asia	108 (107, 109)	110 (108, 112)	
Oceania	111 (107, 117)	112 (108, 118)	
Central Asia, Middle East, and North Africa	114 (112, 116)	115 (113, 118)	
West and Central Africa	106 (105, 107)	108 (107, 109)	
East Africa	112 (110, 113)	114 (112, 116)	
Southern Africa	116 (112, 121)	117 (113, 122)	
Latin America and Caribbean	117 (115, 120)	120 (117, 123)	

	Mean haemoglobin (g/L)		Posterior probability of meeting anaemia target
**Region**	**2000**	**2019**	
Globe	*113 (112, 114)*	*114 (113, 115)*	
Women aged 15-49 years			
High Income	132 (131, 133)	131 (130, 132)	0.000
Eastern and Central Europe	129 (126, 133)	129 (125, 133)	0.012
East and Southeast Asia	128 (125, 130)	130 (128, 133)	0.002
South Asia	117 (115, 119)	118 (116, 121)	0.002
Oceania	124 (120, 128)	124 (119, 130)	0.030
Central Asia, Middle East, and North Africa	123 (121, 124)	125 (123, 127)	0.001
West and Central Africa	115 (113, 118)	118 (116, 120)	0.001
East Africa	122 (120, 124)	125 (123, 127)	0.000
Southern Africa	124 (120, 127)	126 (122, 130)	0.023
Latin America and Caribbean	127 (125, 130)	131 (128, 133)	0.008
Globe	*125 (124, 126)*	*126 (125, 127)*	*0.000*

Table 4. Location of reporting of GATHER checklist items.

Item #	Checklist item	Location reported
**Objectives and funding**		
1	Define the indicator(s), populations (including age, sex, and geographic entities), and time period(s) for which estimates were made.	Methods
2	List the funding sources for the work.	Acknowledgments
**Data Inputs**		
For all data inputs from multiple sources that are synthesized as part of the study:		
3	Describe how the data were identified and how the data were accessed.	Appendix section 1
4	Specify the inclusion and exclusion criteria. Identify all ad-hoc exclusions.	Appendix section 1
5	Provide information on all included data sources and their main characteristics. For each data source used, report reference information or contact name/institution, population represented, data collection method, year(s) of data collection, sex and age range, diagnostic criteria or measurement method, and sample size, as relevant.	https://www.who.int/data/gho/data/themes/topics/anaemia_in_women_and_children
6	Identify and describe any categories of input data that have potentially important biases (e.g., based on characteristics listed in item 5).	Appendix sections 3 and 4
For data inputs that contribute to the analysis but were not synthesized as part of the study:		
7	Describe and give sources for any other data inputs.	Appendix section 1.3
For all data inputs:		
8	Provide all data inputs in a file format from which data can be efficiently extracted (e.g., a spreadsheet rather than a PDF), including all relevant meta-data listed in item 5. For any data inputs that cannot be shared because of ethical or legal reasons, such as third-party ownership, provide a contact name or the name of the institution that retains the right to the data.	https://www.who.int/data/gho/data/themes/topics/anaemia_in_women_and_children
**Data analysis**		
9	Provide a conceptual overview of the data analysis method. A diagram may be helpful.	Appendix section 4.1
10	Provide a detailed description of all steps of the analysis, including mathematical formulae. This description should cover, as relevant, data cleaning, data pre-processing, data adjustments and weighting of data sources, and mathematical or statistical model(s).	Appendix sections 1-4

**11**	Describe how candidate models were evaluated and how the final model(s) were selected.	Appendix section 4.2
**12**	Provide the results of an evaluation of model performance, if done, as well as the results of any relevant sensitivity analysis.	Appendix section 4.2-4.3
**13**	Describe methods for calculating uncertainty of the estimates. State which sources of uncertainty were, and were not, accounted for in the uncertainty analysis.	Appendix section 4.3
**14**	State how analytic or statistical source code used to generate estimates can be accessed.	Appendix section 4.3
**Results and Discussion**		
**15**	Provide published estimates in a file format from which data can be efficiently extracted.	https://www.who.int/data/gho/data/themes/topics/anaemia_in_women_and_children[2]
**16**	Report a quantitative measure of the uncertainty of the estimates (e.g., uncertainty intervals).	https://www.who.int/data/gho/data/themes/topics/anaemia_in_women_and_children
**17**	Interpret results in light of existing evidence. If updating a previous set of estimates, describe the reasons for changes in estimates.	Discussion, appendix Figure 7
**18**	Discuss limitations of the estimates. Include a discussion of any modelling assumptions or data limitations that affect interpretation of the estimates.	Discussion

[2] Estimate for Turkey is not available.

Figure 1. Flowchart of data identification, access, and extraction.

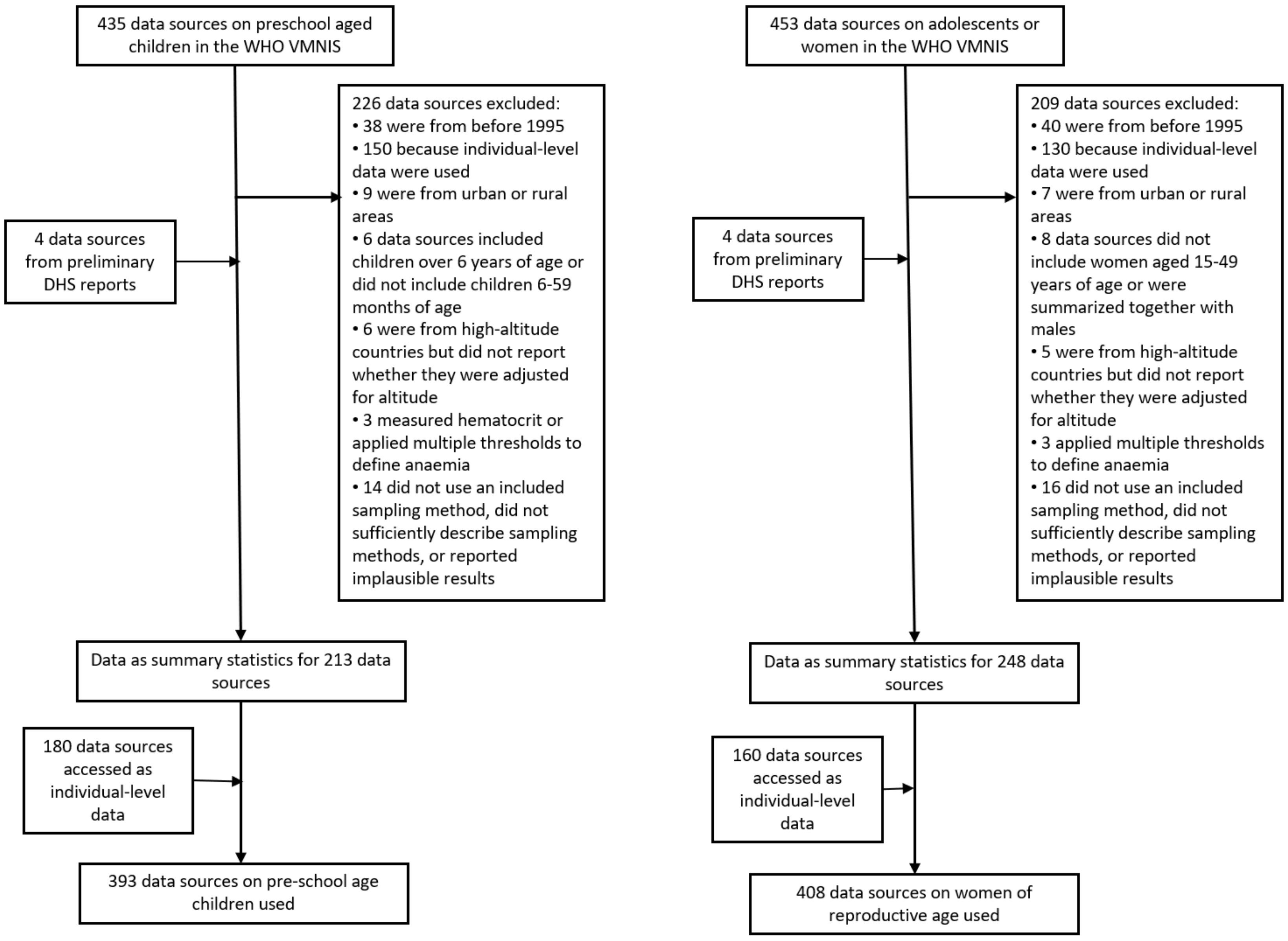

Figure 2. Number of data sources included in each country/territory.[1] Data sources may include data on women aged 15–49 years, children aged 6–59 months, or both populations.

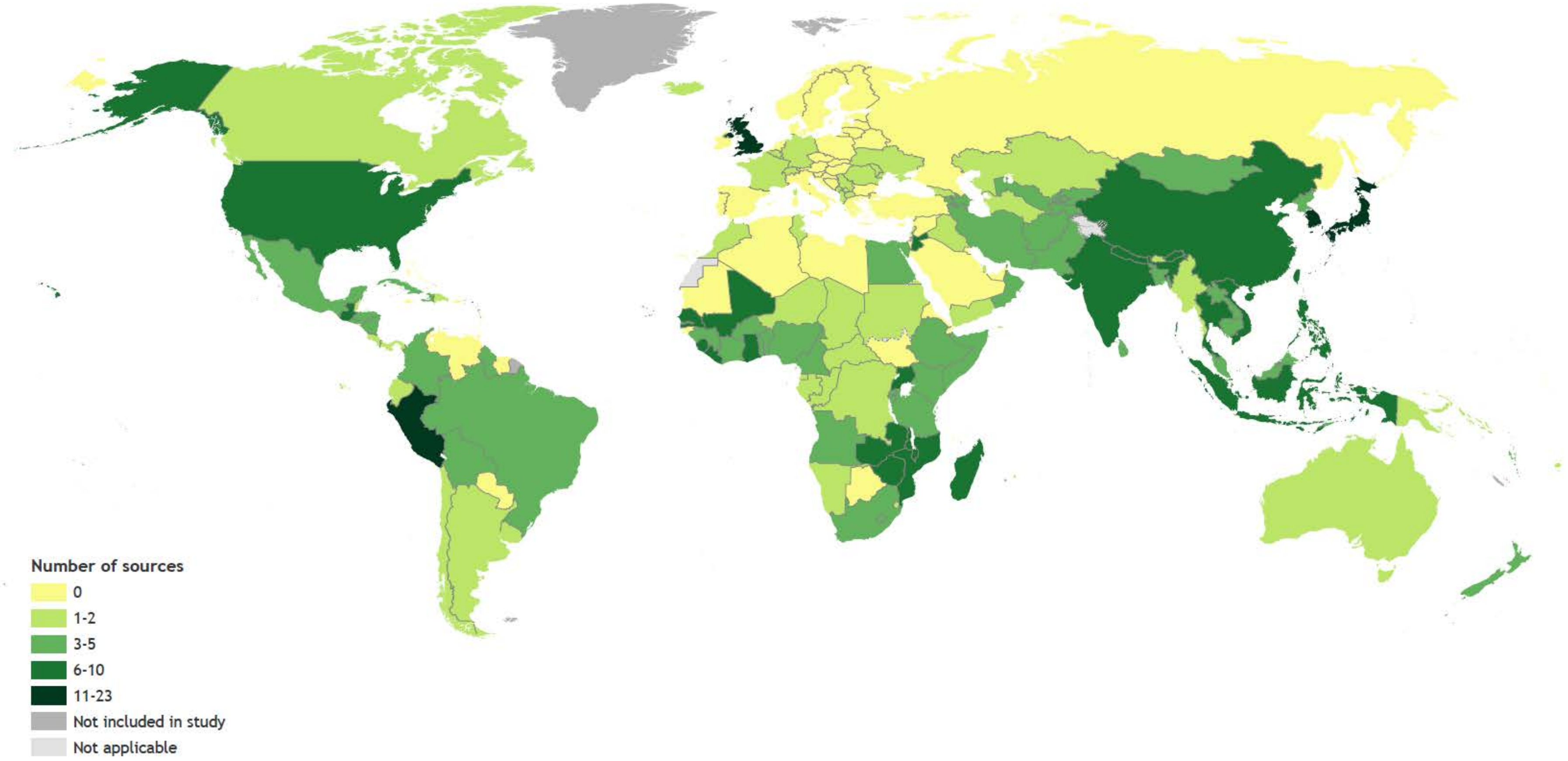

[1] Three data sources were included for China, Province of Taiwan.

Figure 3. Comparison of relative change in anaemia prevalence during 2000–2019 to anaemia prevalence in 2000 by region, children aged 6–59 months and women aged 15–49 years. Error bars show the 95% uncertainty intervals.

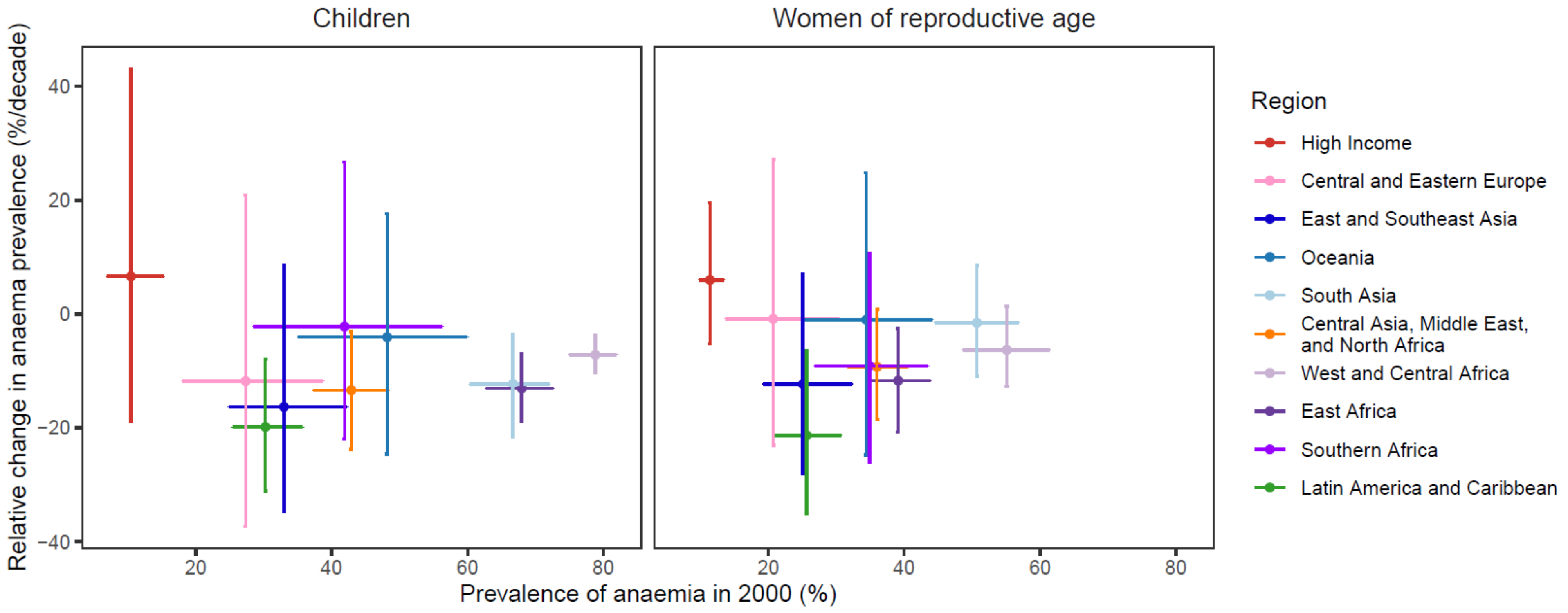

Figure 4. Change in mean haemoglobin (Hb) during 2000–2009 and 2010–2019, globally and by region, (A) children aged 6–59 months and (B) women aged 15–49 years. Error bars show 95% uncertainty intervals.

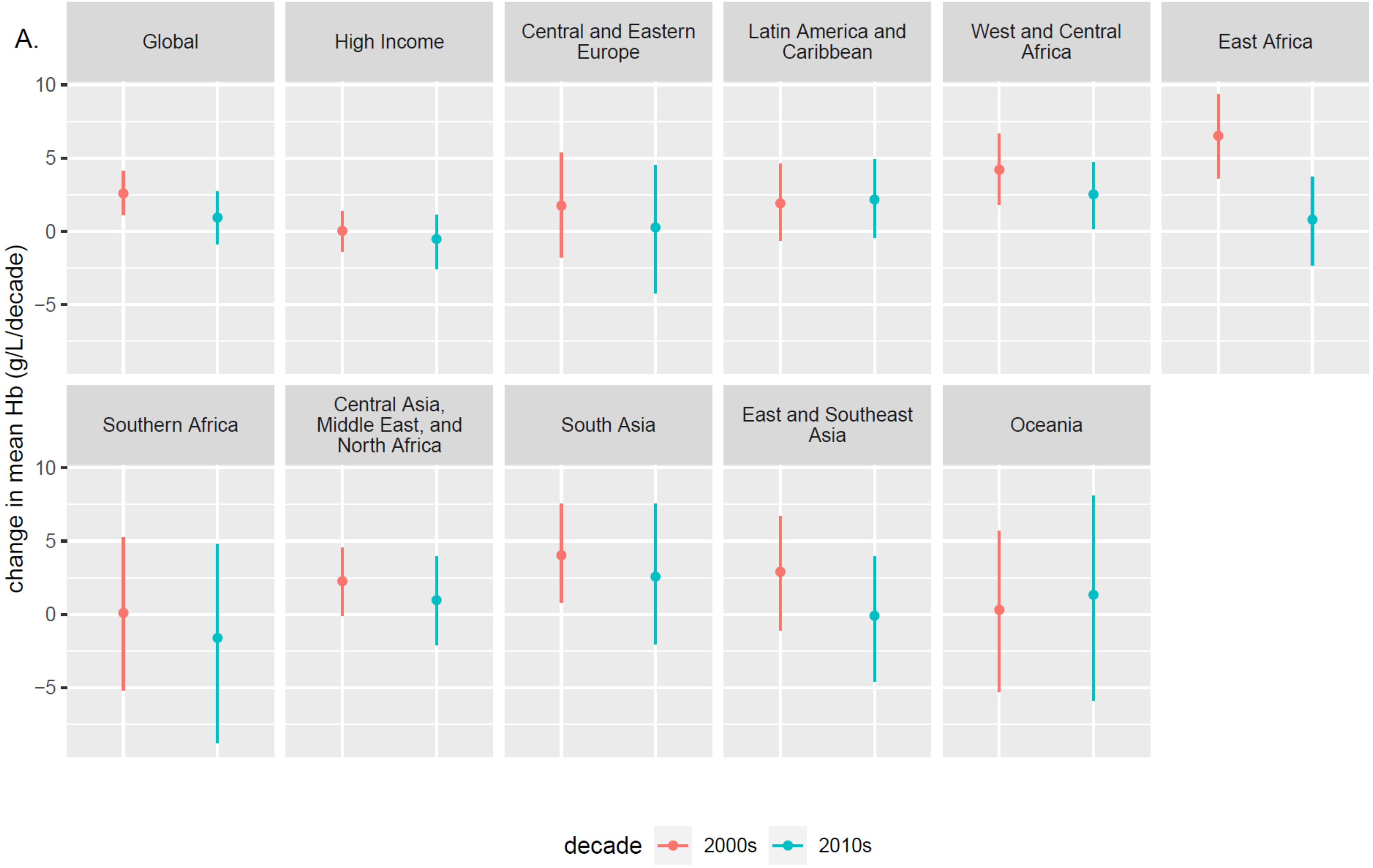

Figure 4 (continued). Change in mean haemoglobin (Hb) during 2000–2009 and 2010–2019, globally and by region, (A) children aged 6–59 months and (B) women aged 15–49 years. Error bars show 95% uncertainty intervals.

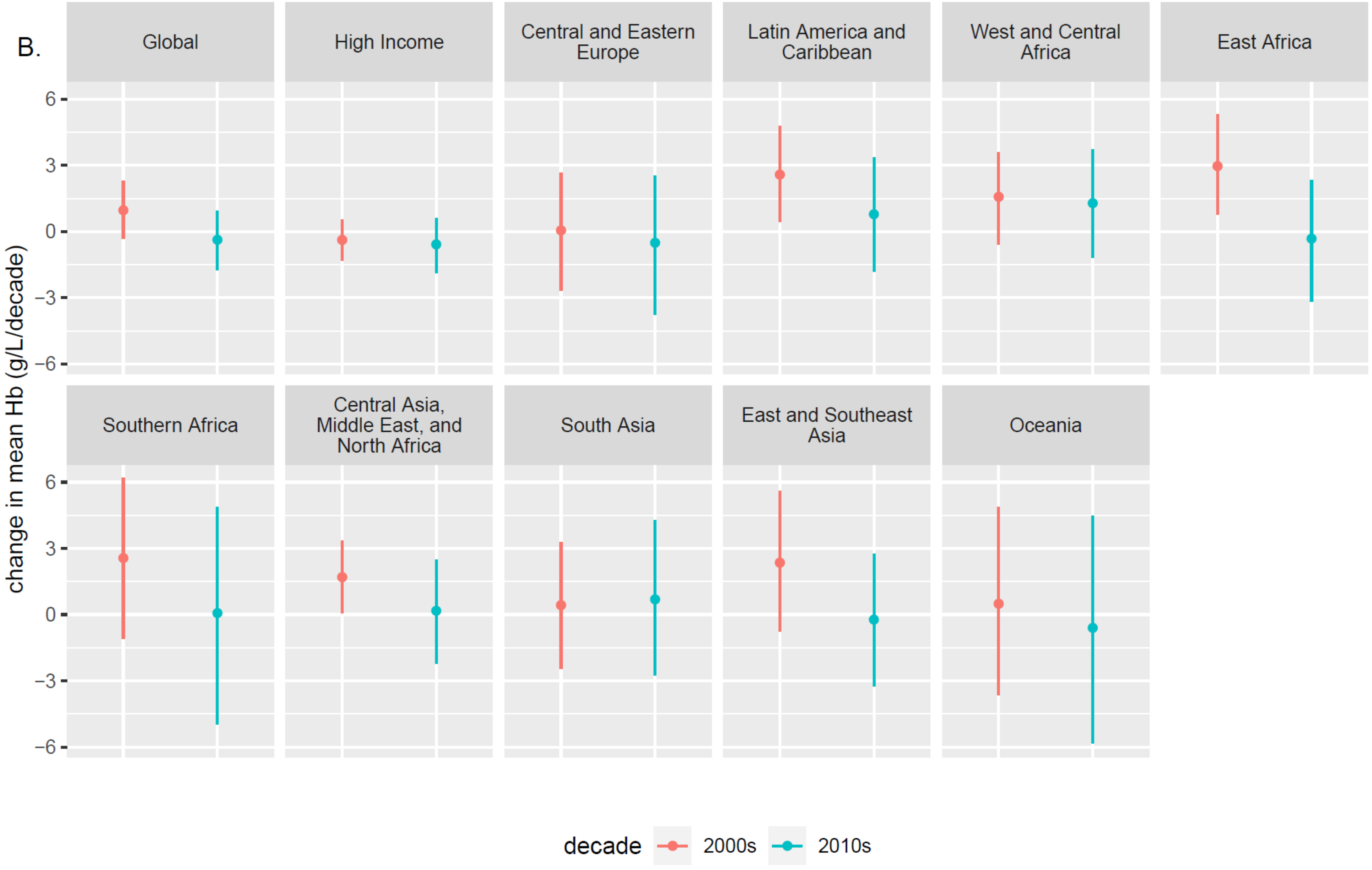

Figure 5. Comparison of mean haemoglobin of non-pregnant women aged 15–49 years, with those of pregnant women aged 15–49 years and children aged 6–59 months in 2000 and 2019. Error bars show the 95% uncertainty intervals.

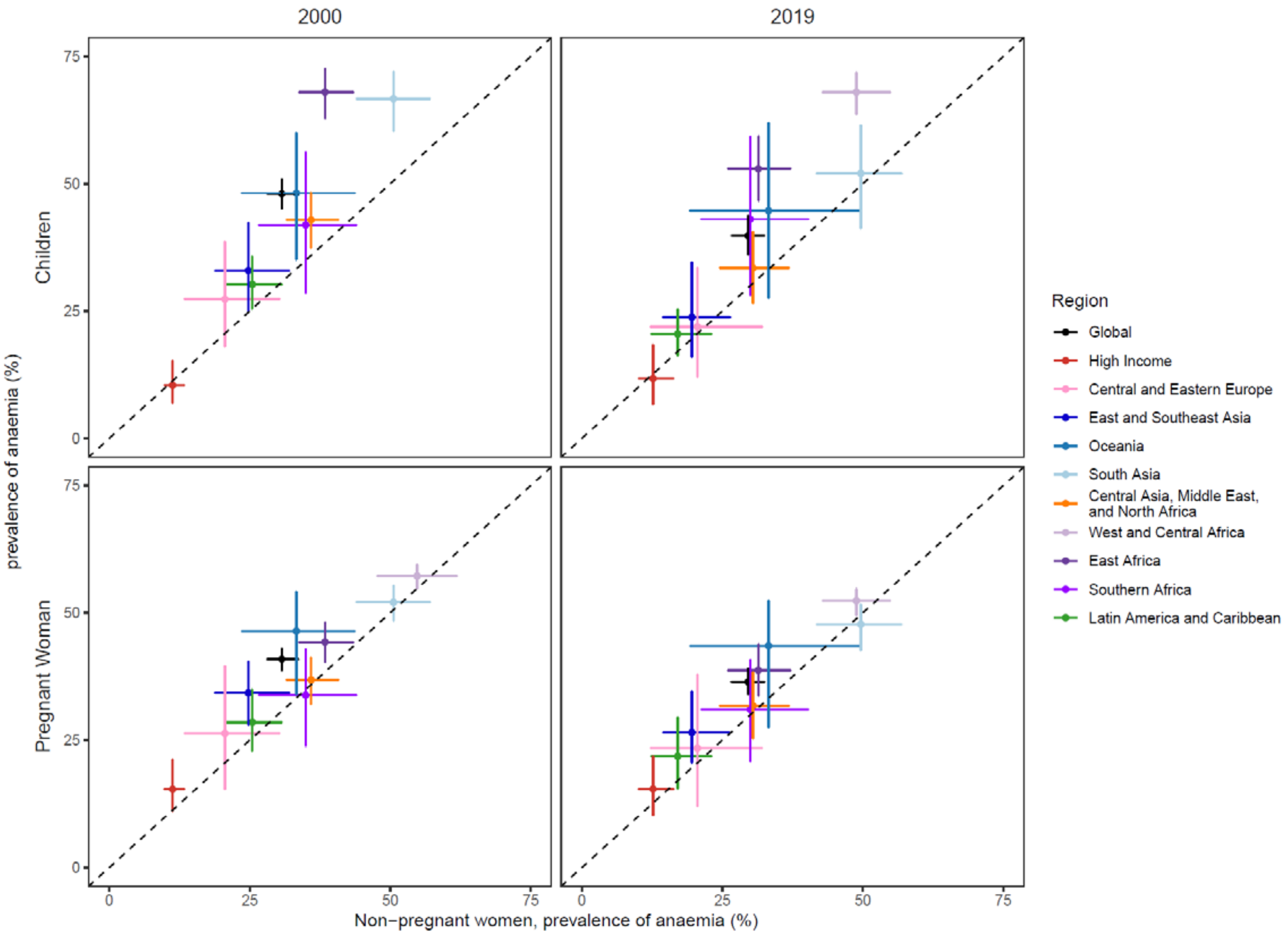

Figure 6. Posterior probability of reducing total anaemia prevalence by 50% by 2030 (with a baseline year of 2012), by country/territory, assuming trends 2012–2019 continue to 2030. Results shown for countries with at least 2 data sources.[1]

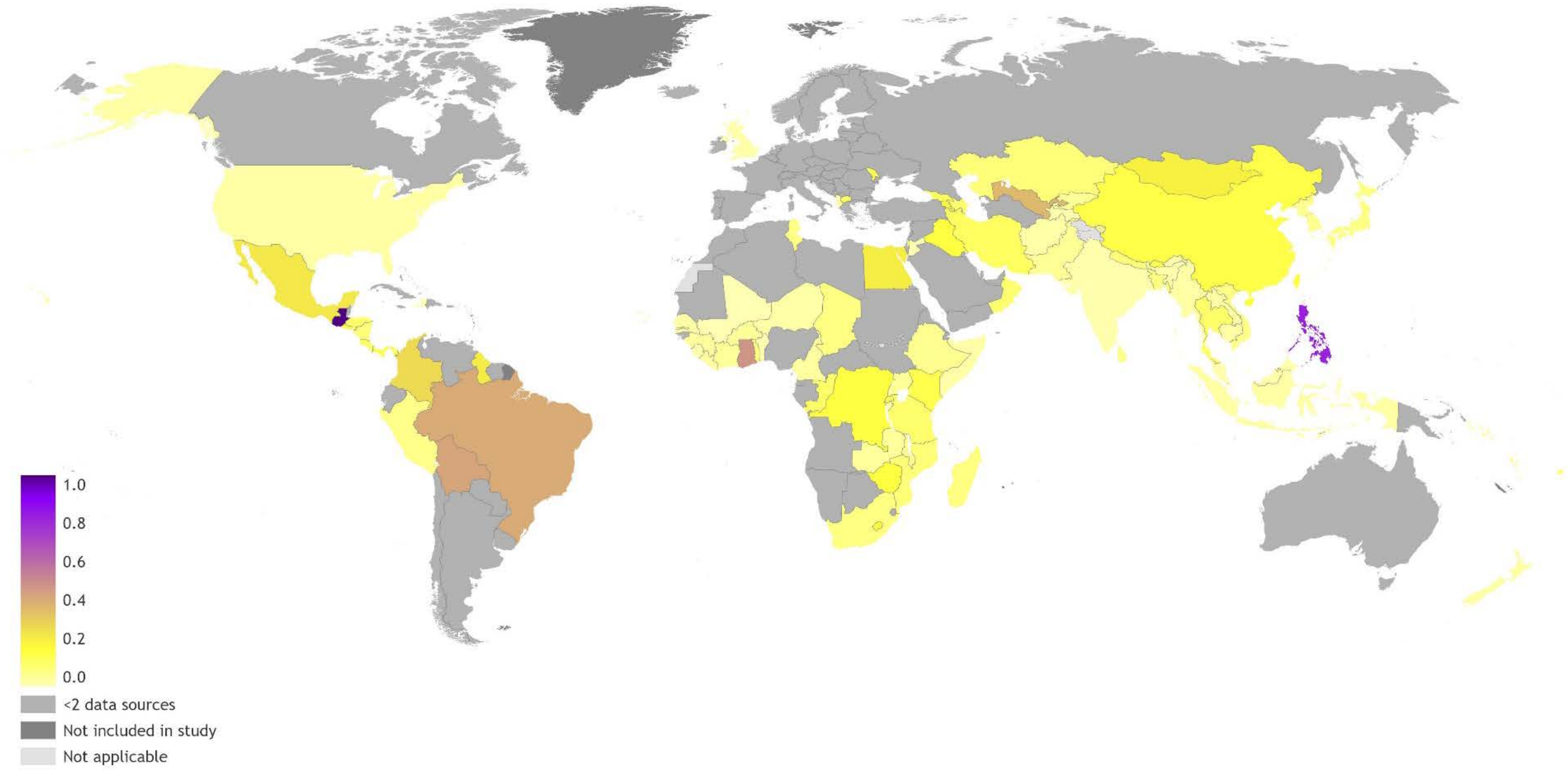

[1] The estimate for China, Province of Taiwan is 0.052.

Figure 7. Trends in mean haemoglobin concentration, total anaemia, and severe anaemia prevalence by country/territory between 2000 and 2019. Trends are shown in relation to observed (survey) data for all countries with primary data. The shaded areas show 95% uncertainty intervals. Data using other definitions of anaemia were used in the analysis, accounting for the specific anaemia definitions, but are not shown here. The number of observations for each country/territory used but not shown is reported in each panel. Observed anaemia prevalences for combined groups of pregnant and non-pregnant women are based on cut-offs for non-pregnant women (120 g/L for total anaemia and 80 g/L for severe anaemia).

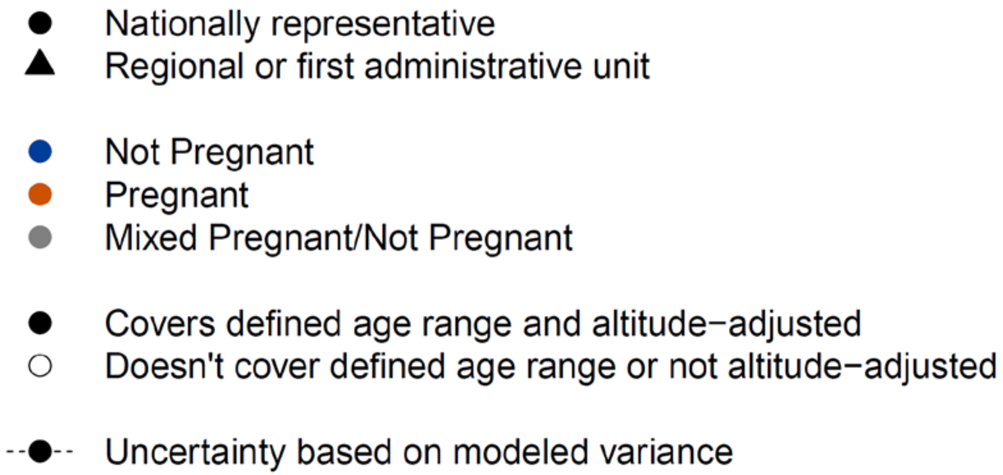

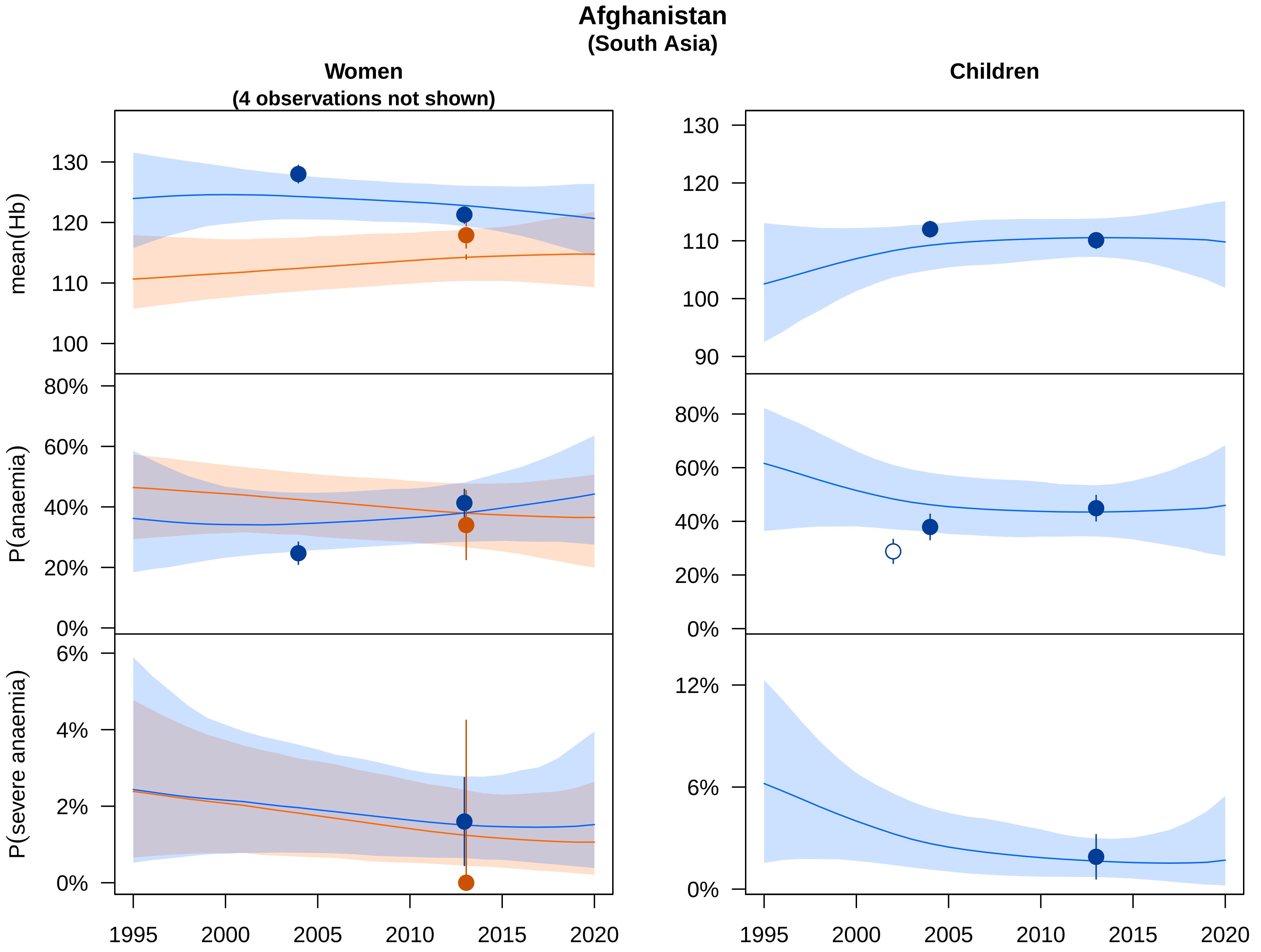

Afghanistan
(South Asia)
Women
(4 observations not shown)
Children
mean(Hb)
P(anaemia)
P(severe anaemia)
130
120
110
100
90
80%
60%
40%
20%
0%
6%
4%
2%
12%
1995
2000
2005
2010
2015
2020

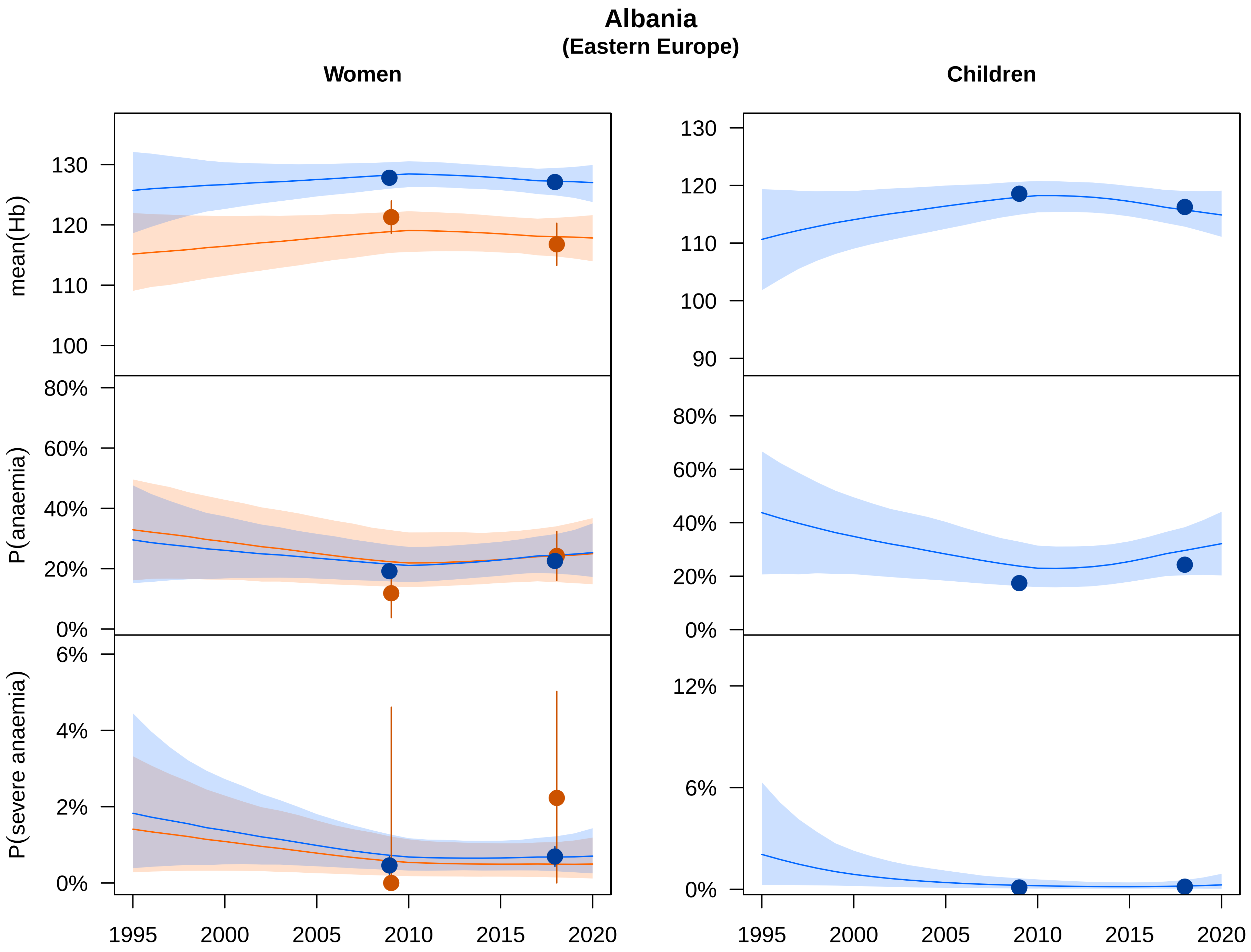

Albania
(Eastern Europe)
Women
Children
mean(Hb)
P(anaemia)
P(severe anaemia)
130
120
110
100
90
80%
60%
40%
20%
0%
6%
4%
2%
12%
1995
2000
2005
2010
2015
2020

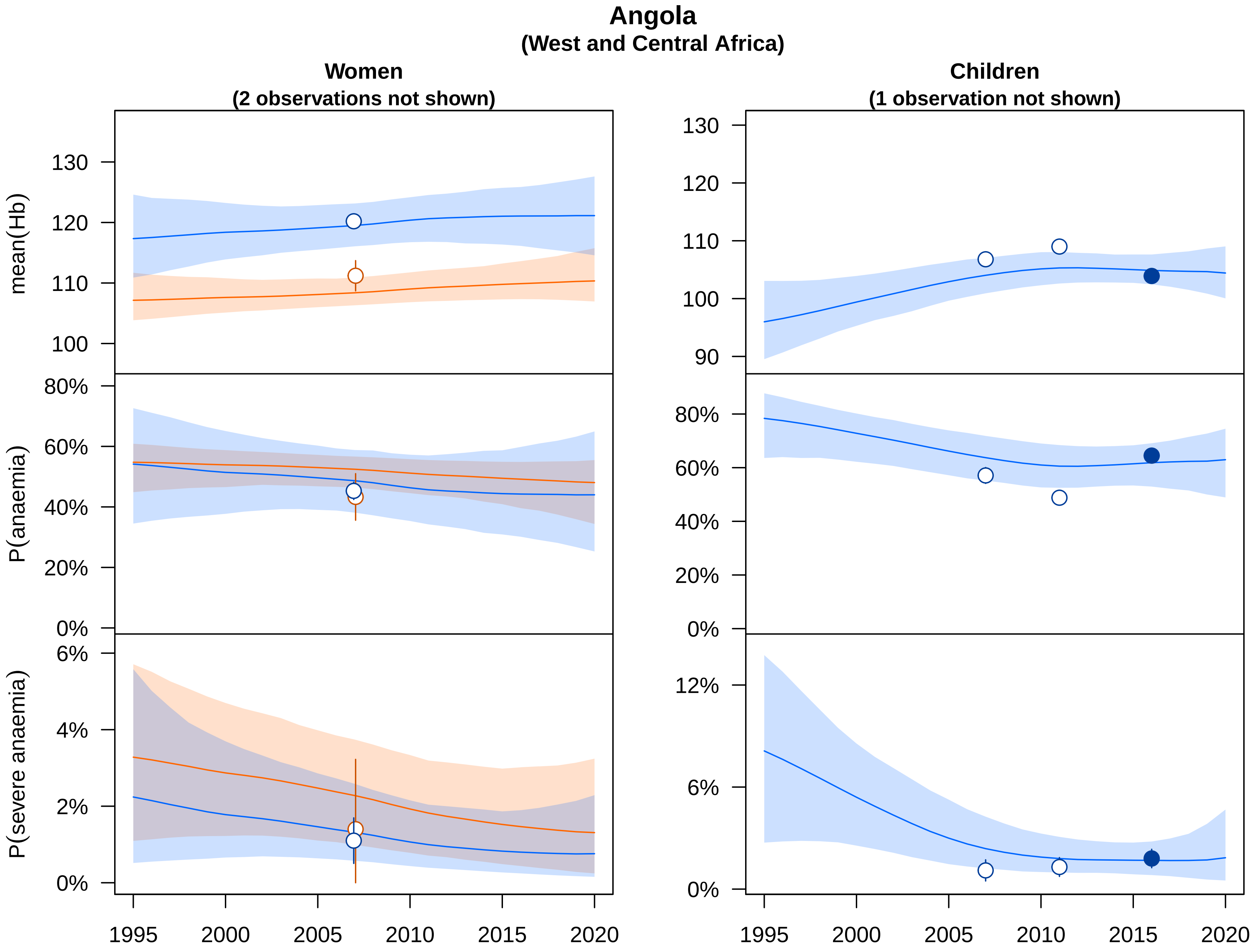

Angola
(West and Central Africa)
Women
(2 observations not shown)
Children
(1 observation not shown)
mean(Hb)
P(anaemia)
P(severe anaemia)
130
120
110
100
90
80%
60%
40%
20%
0%
6%
4%
2%
12%
1995
2000
2005
2010
2015
2020

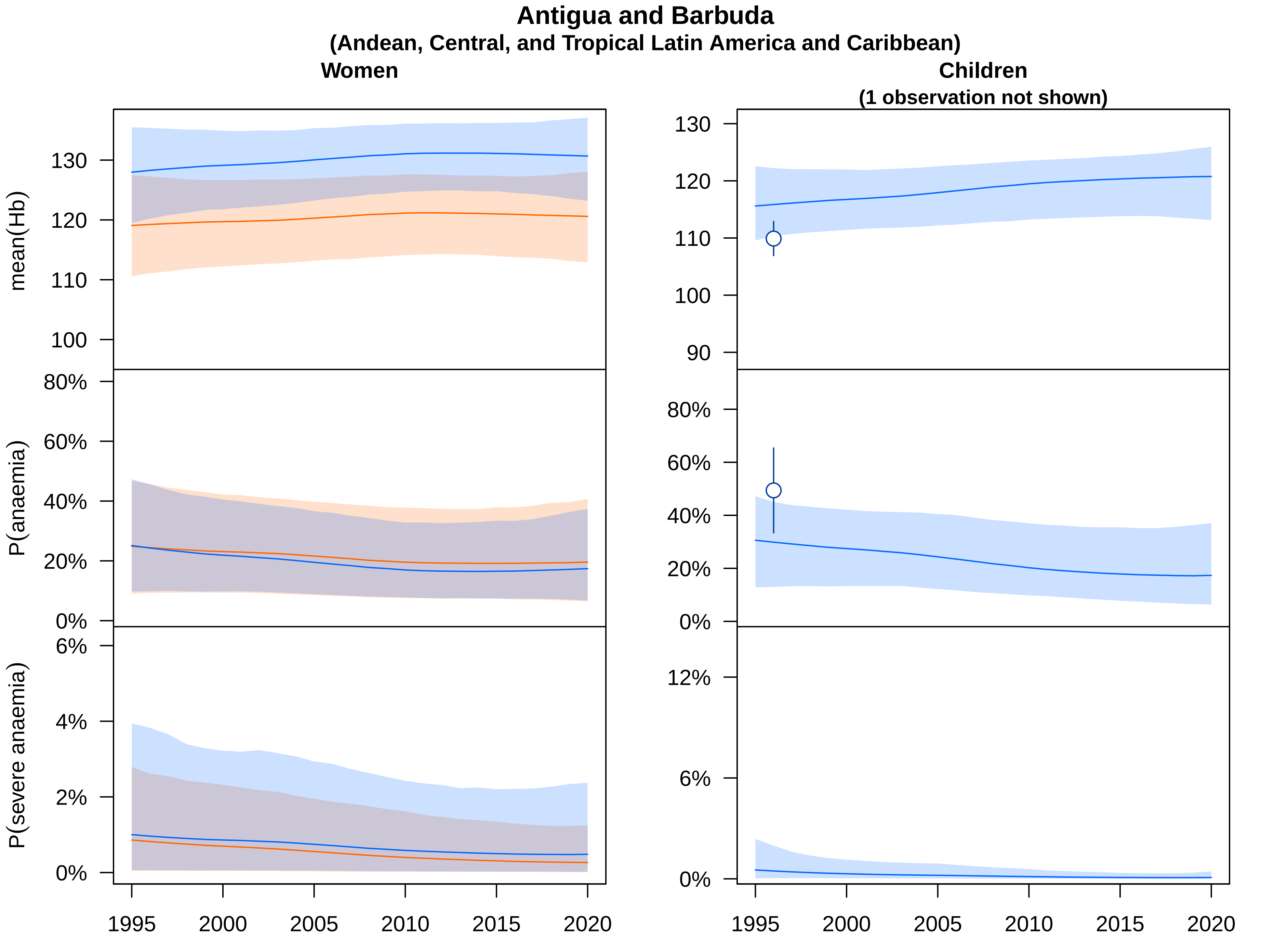

Antigua and Barbuda
(Andean, Central, and Tropical Latin America and Caribbean)
Women
Children
(1 observation not shown)
mean(Hb)
P(anaemia)
P(severe anaemia)
130
120
110
100
90
80%
60%
40%
20%
0%
6%
4%
2%
12%
1995
2000
2005
2010
2015
2020

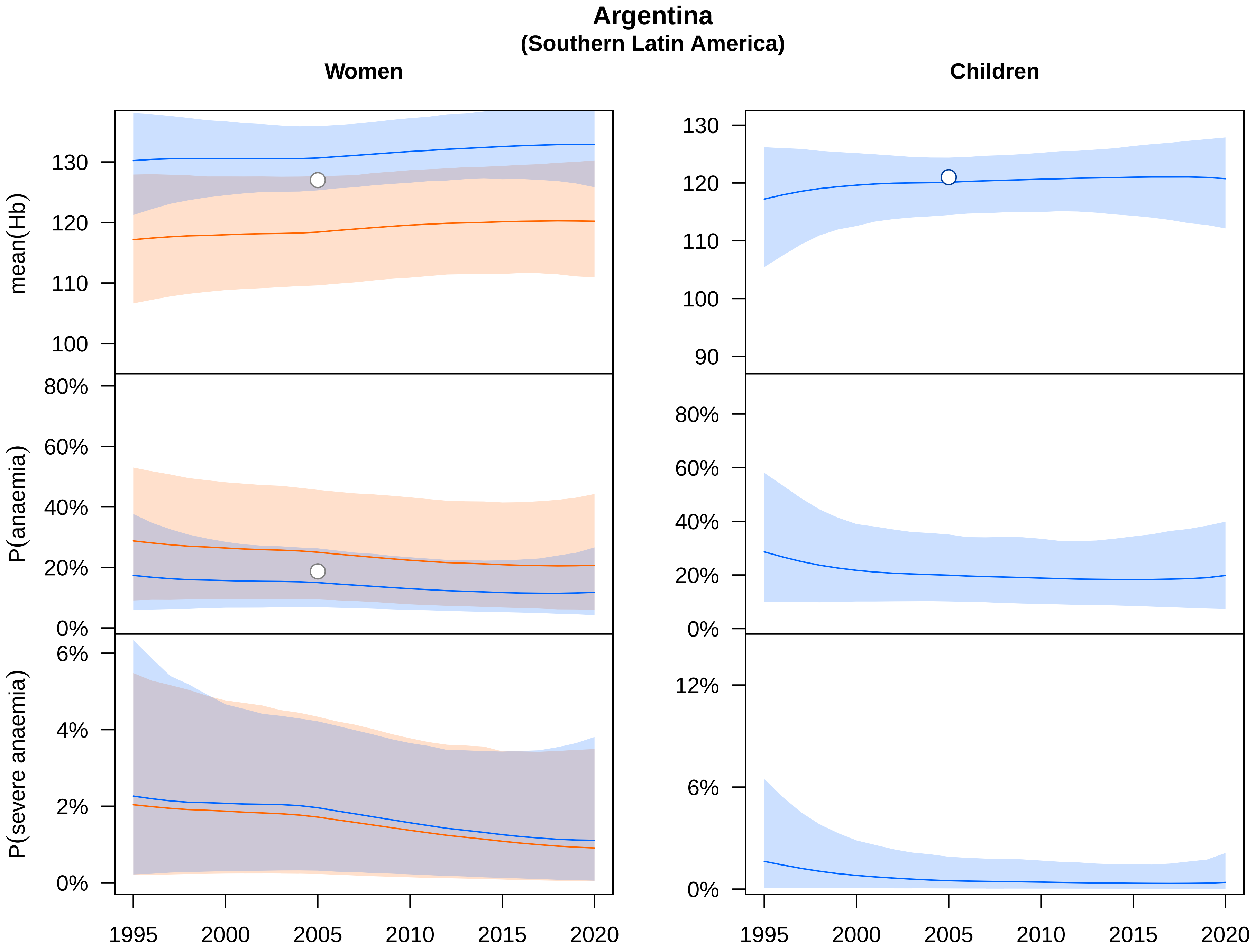

Argentina
(Southern Latin America)
Women
Children
mean(Hb)
P(anaemia)
P(severe anaemia)
130
120
110
100
90
80%
60%
40%
20%
0%
6%
4%
2%
12%
1995
2000
2005
2010
2015
2020

Armenia
(Central Asia, Middle East, and North Africa)

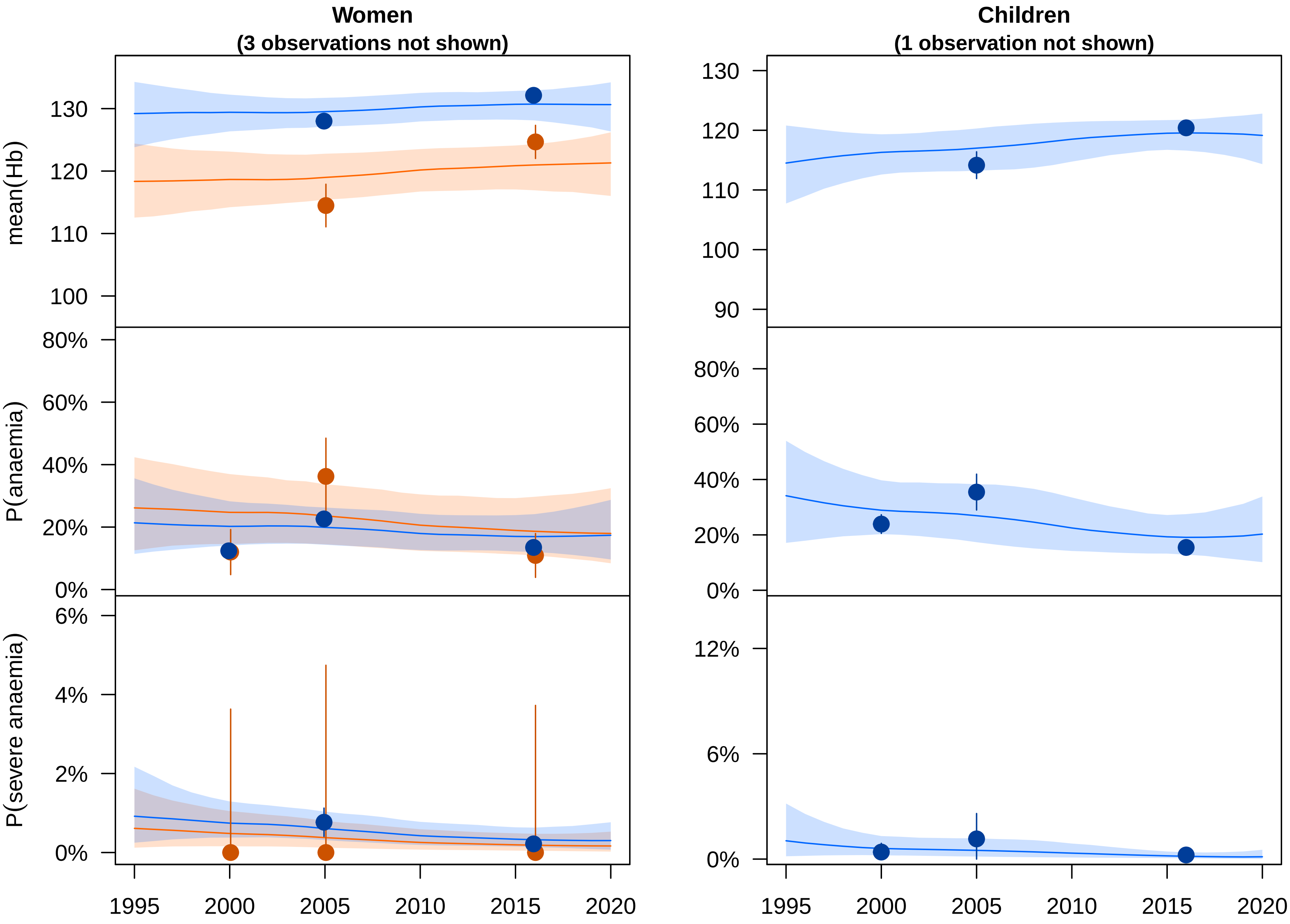

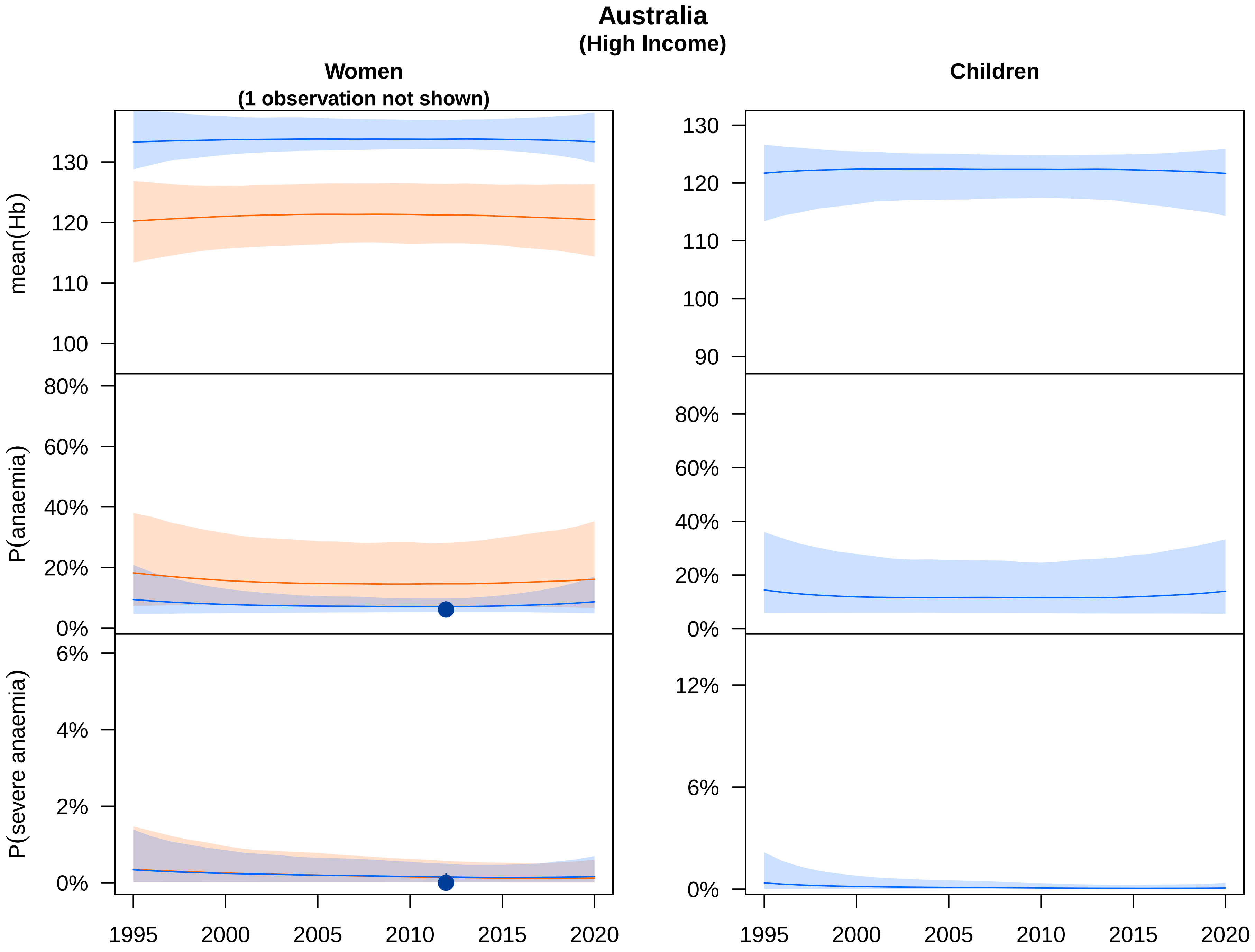

Australia
(High Income)
Women
(1 observation not shown)
Children
mean(Hb)
P(anaemia)
P(severe anaemia)
130
120
110
100
90
80%
60%
40%
20%
0%
6%
4%
2%
12%
1995
2000
2005
2010
2015
2020

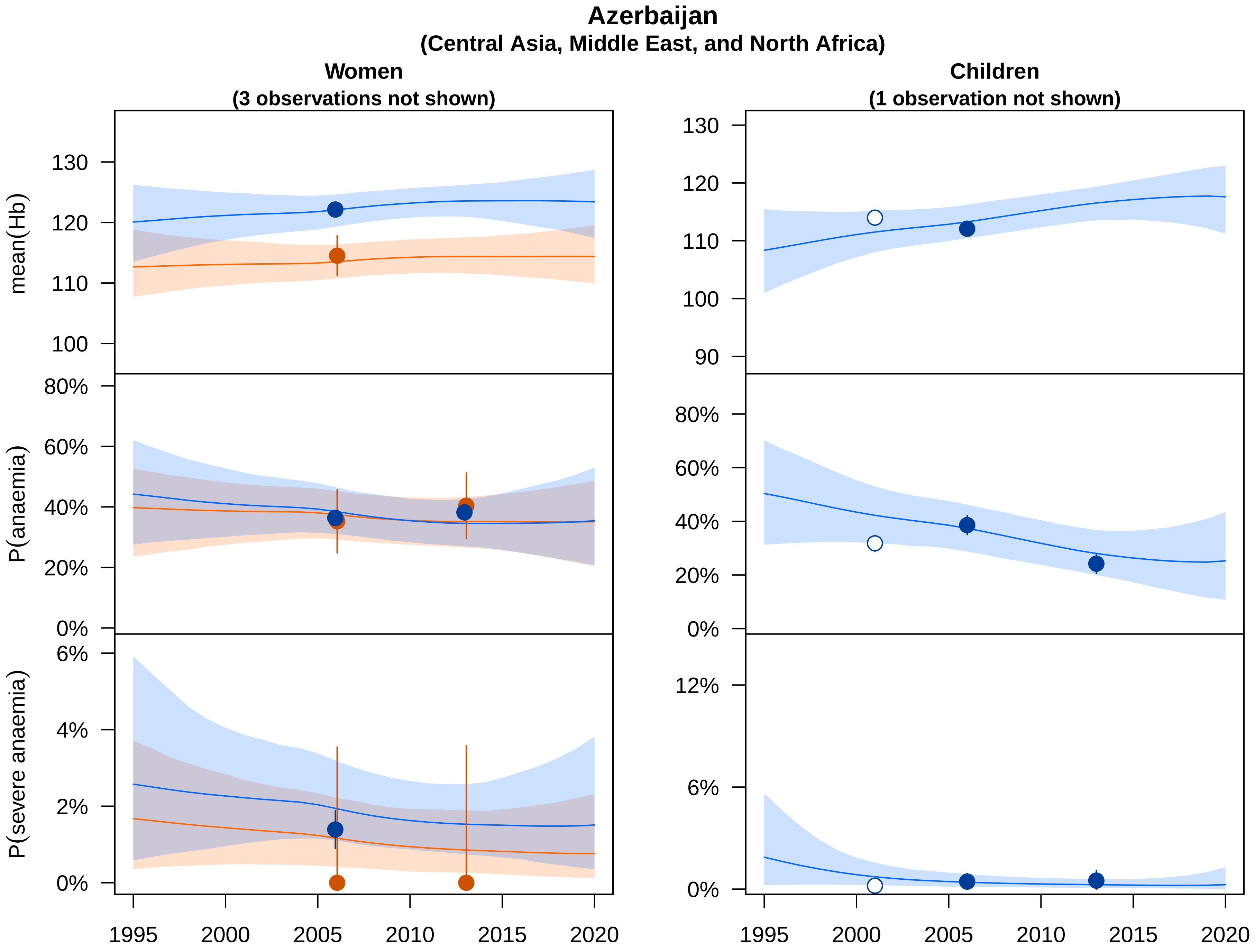
Azerbaijan
(Central Asia, Middle East, and North Africa)
Women
(3 observations not shown)
Children
(1 observation not shown)
mean(Hb)
P(anaemia)
P(severe anaemia)
130
120
110
100
90
80%
60%
40%
20%
0%
6%
4%
2%
12%
1995
2000
2005
2010
2015
2020

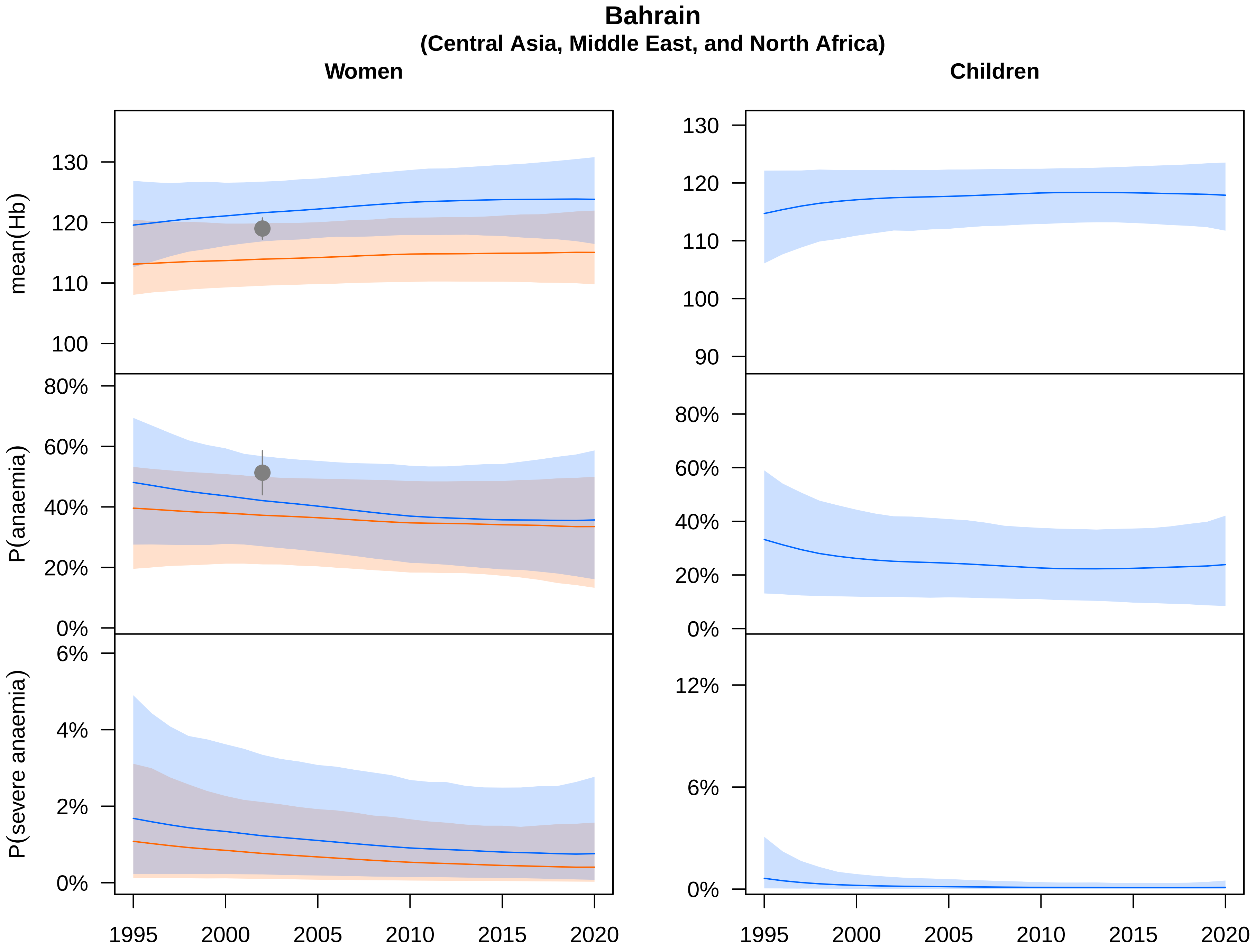

Bahrain
(Central Asia, Middle East, and North Africa)
Women
Children
mean(Hb)
P(anaemia)
P(severe anaemia)
130
120
110
100
90
80%
60%
40%
20%
0%
6%
4%
2%
12%
1995
2000
2005
2010
2015
2020

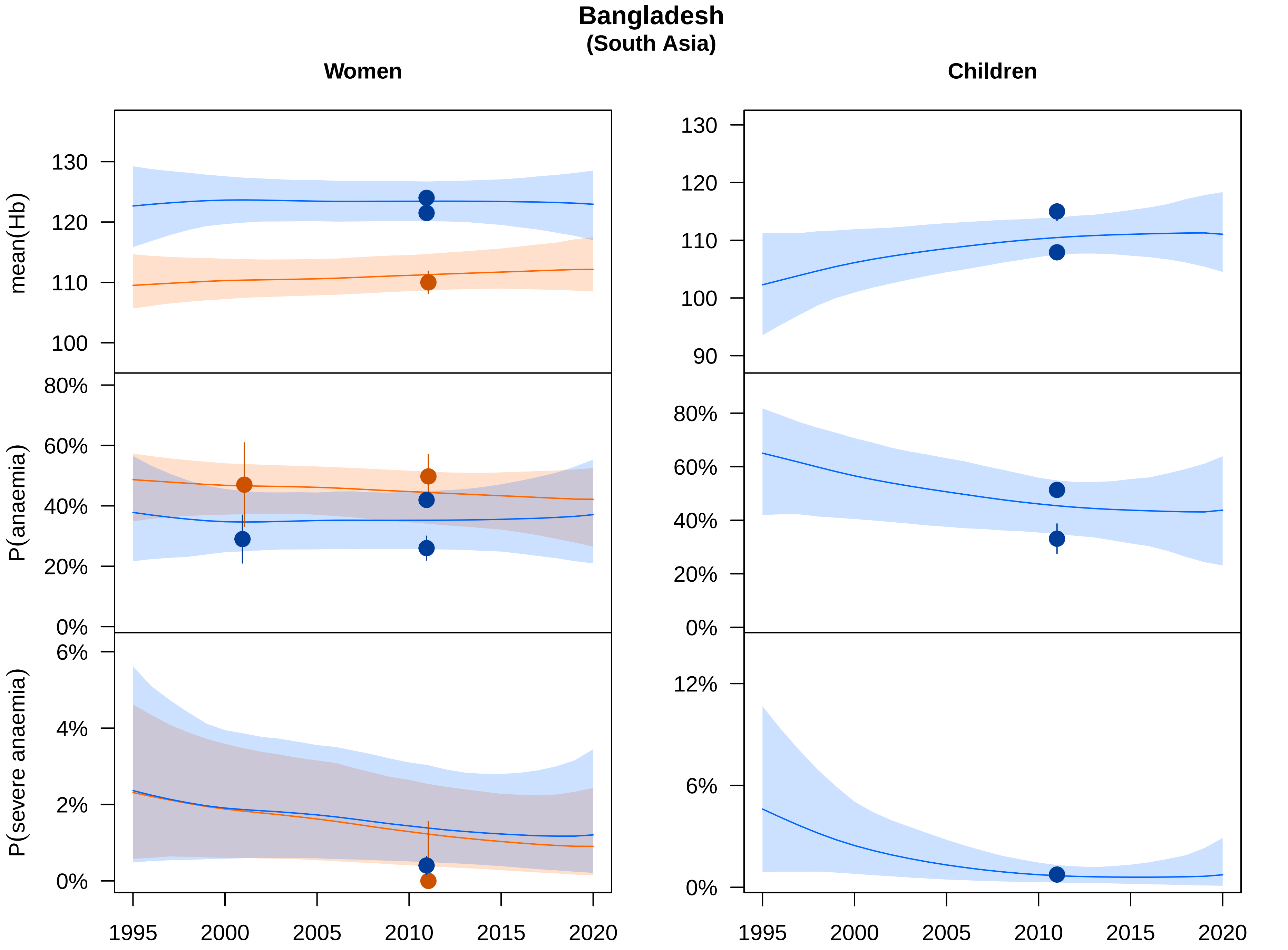

Bangladesh
(South Asia)
Women
Children
mean(Hb)
P(anaemia)
P(severe anaemia)
130
120
110
100
90
80%
60%
40%
20%
0%
6%
4%
2%
12%
1995
2000
2005
2010
2015
2020

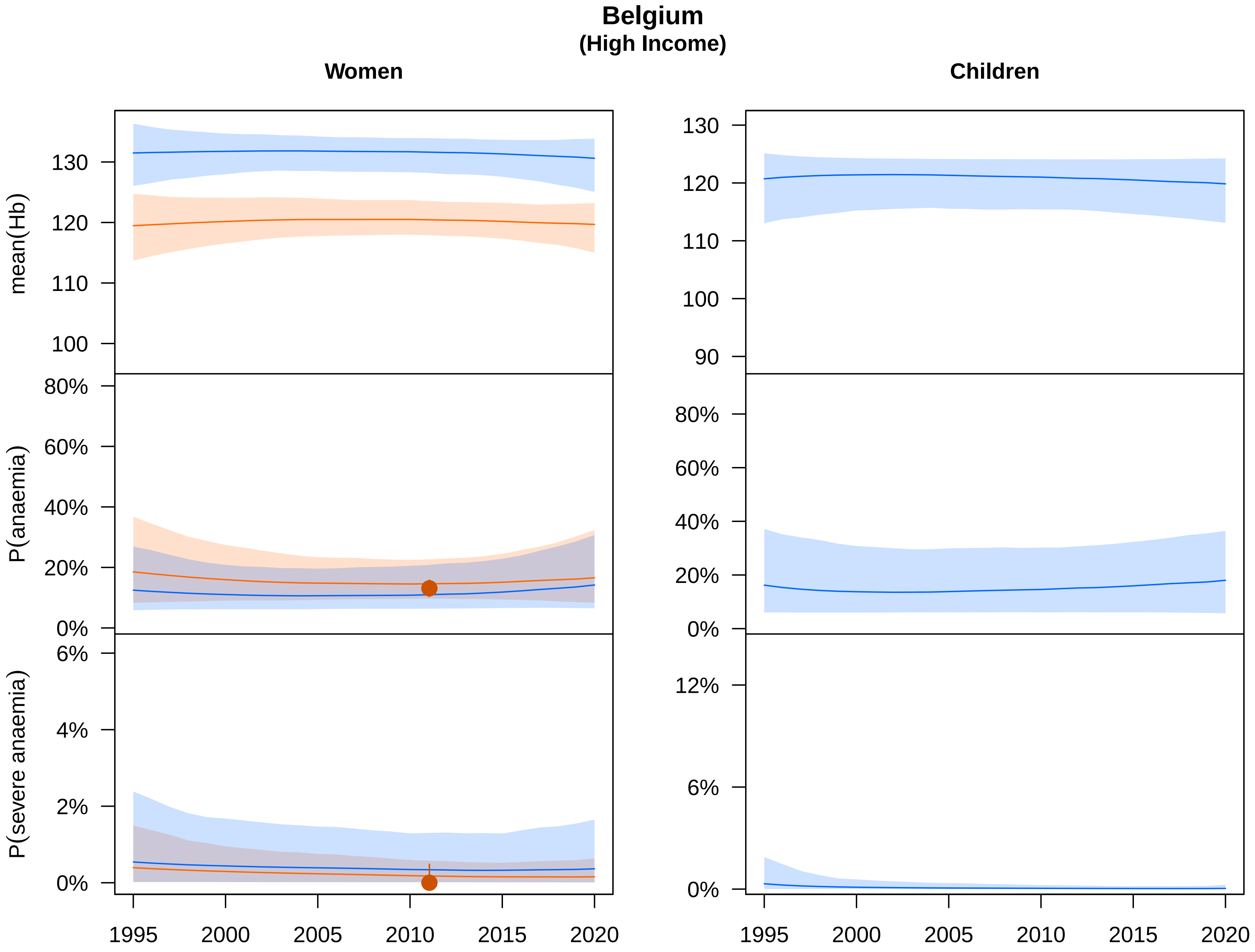

Belgium
(High Income)
Women
Children
mean(Hb)
P(anaemia)
P(severe anaemia)
130
120
110
100
90
80%
60%
40%
20%
0%
6%
4%
2%
12%
1995
2000
2005
2010
2015
2020

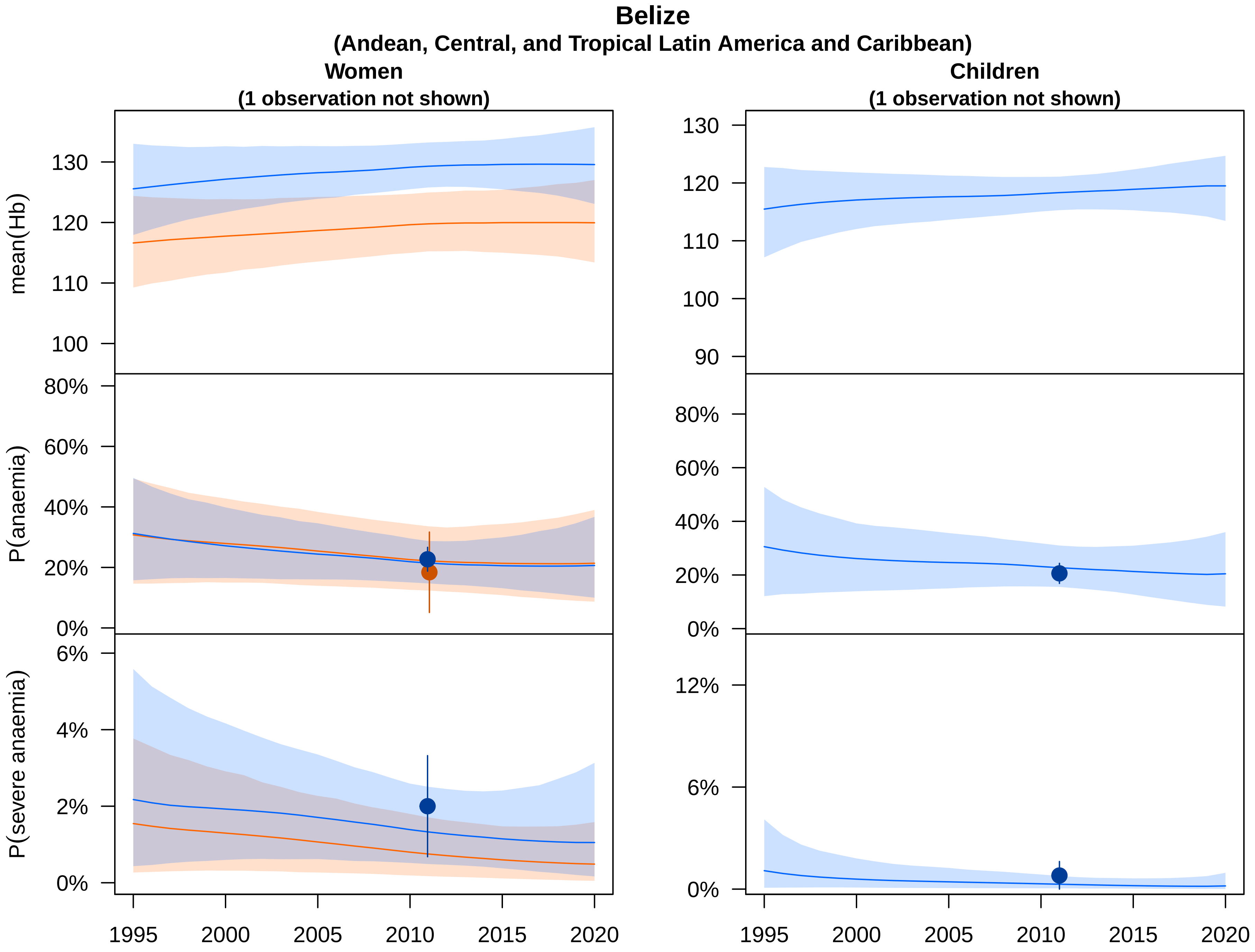

Belize
(Andean, Central, and Tropical Latin America and Caribbean)
Women
(1 observation not shown)
Children
(1 observation not shown)
mean(Hb)
P(anaemia)
P(severe anaemia)
130
120
110
100
90
80%
60%
40%
20%
0%
6%
4%
2%
12%
1995
2000
2005
2010
2015
2020

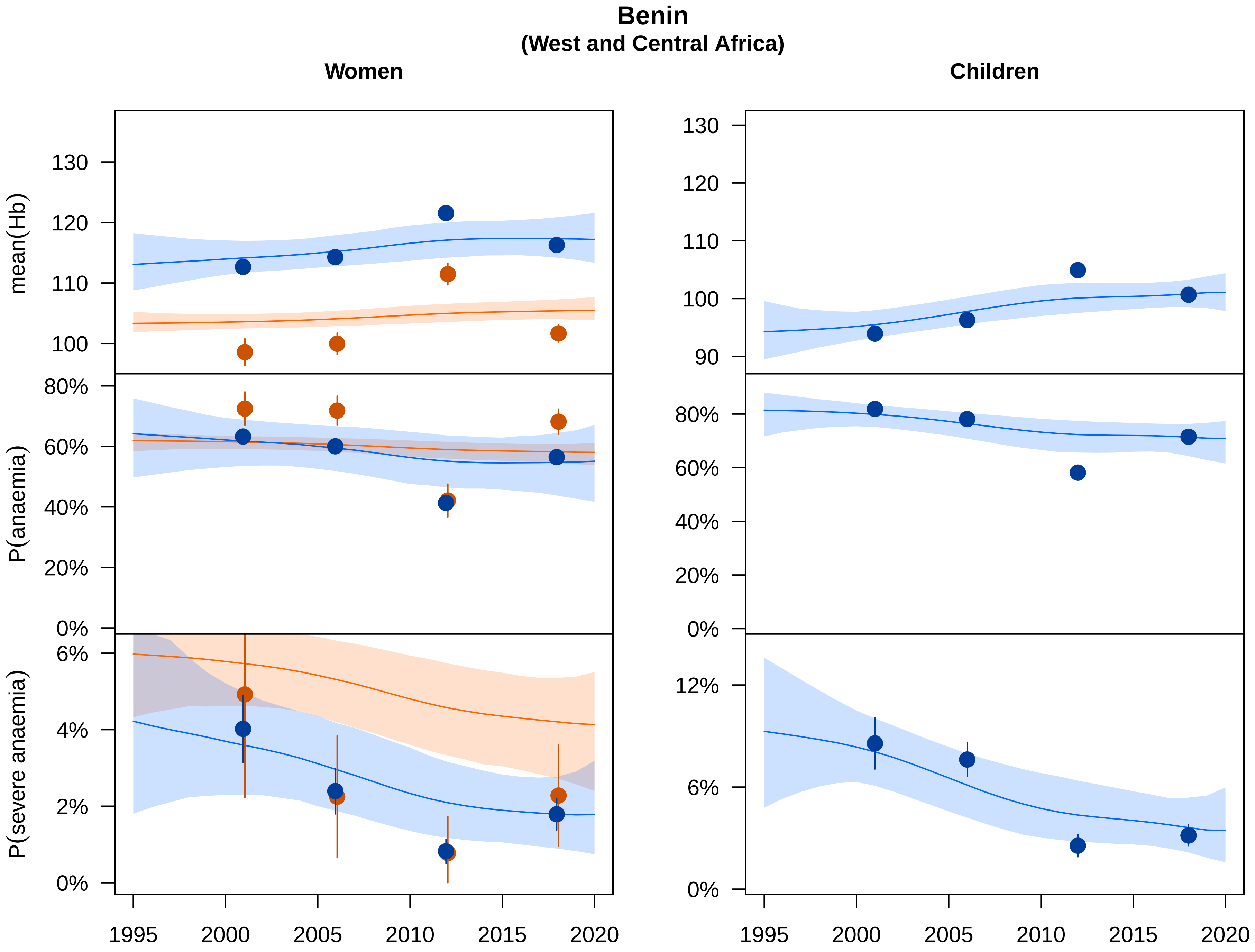
Benin
(West and Central Africa)
Women
Children
mean(Hb)
P(anaemia)
P(severe anaemia)
130
120
110
100
90
80%
60%
40%
20%
0%
6%
4%
2%
12%
1995
2000
2005
2010
2015
2020

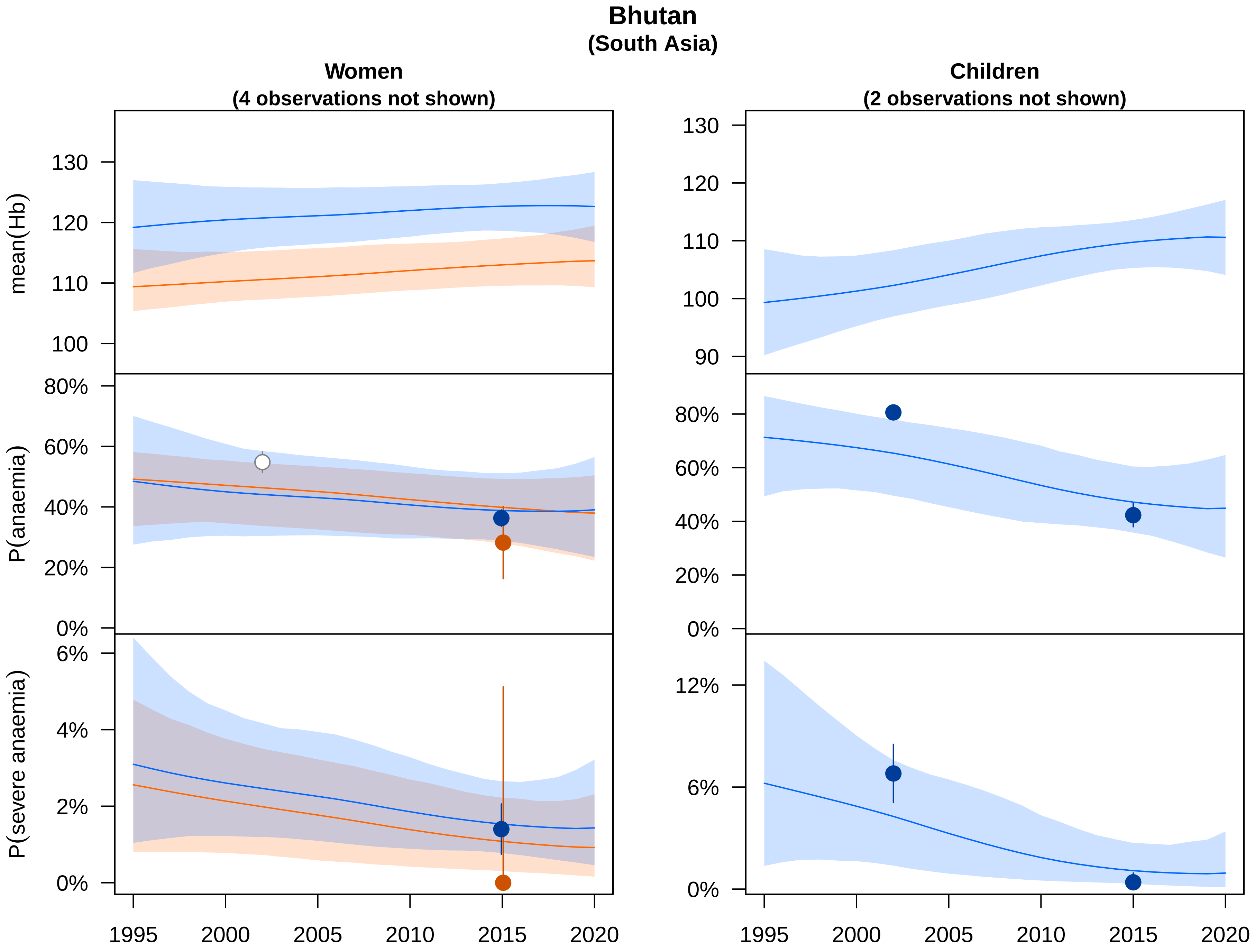
Bhutan
(South Asia)
Women
(4 observations not shown)
Children
(2 observations not shown)
mean(Hb)
P(anaemia)
P(severe anaemia)
130
120
110
100
90
80%
60%
40%
20%
0%
6%
4%
2%
12%
1995
2000
2005
2010
2015
2020

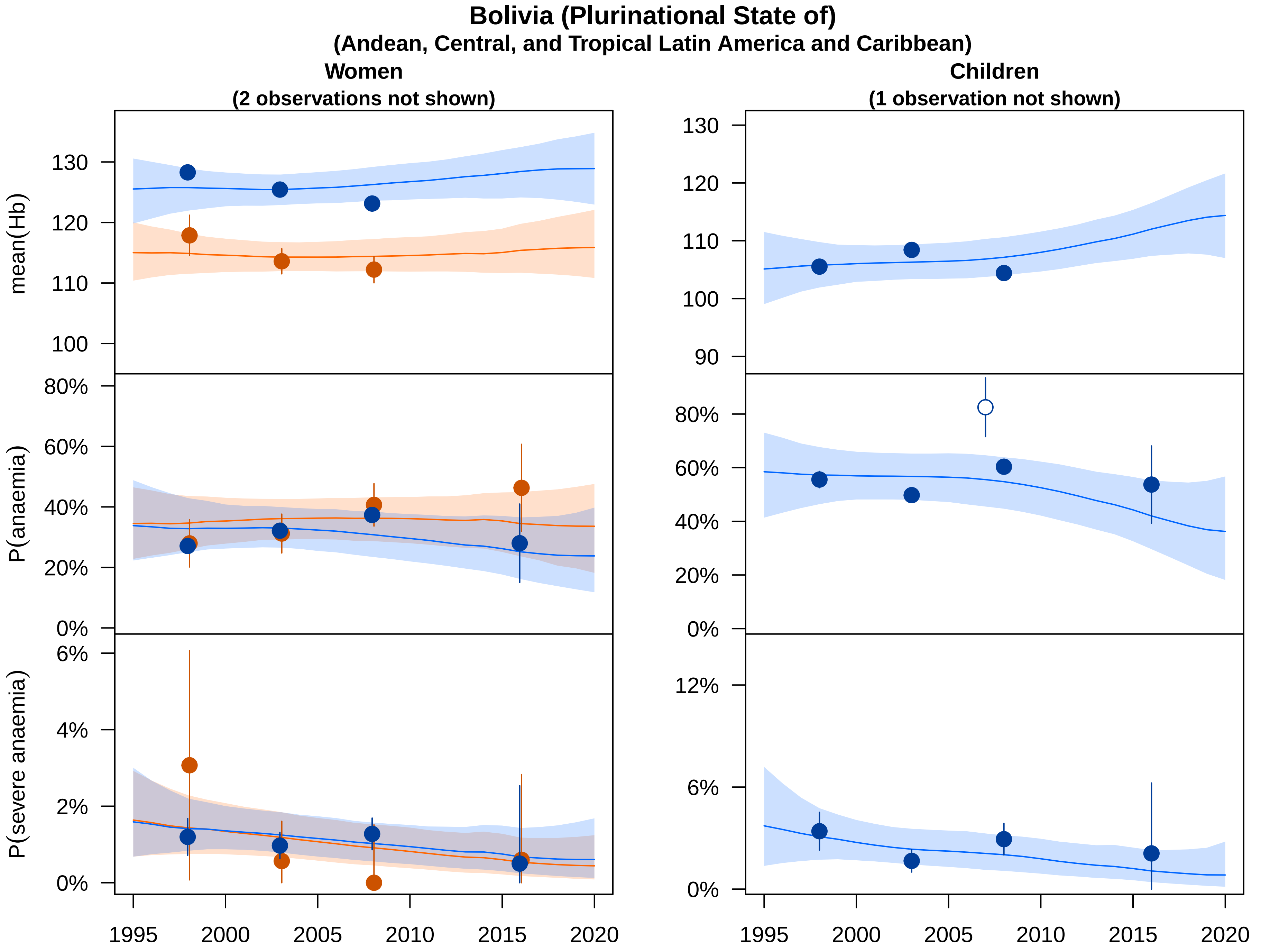

Bolivia (Plurinational State of)
(Andean, Central, and Tropical Latin America and Caribbean)
Women
(2 observations not shown)
Children
(1 observation not shown)
mean(Hb)
P(anaemia)
P(severe anaemia)
130
120
110
100
90
80%
60%
40%
20%
0%
6%
4%
2%
12%
1995
2000
2005
2010
2015
2020

Brazil
(Andean, Central, and Tropical Latin America and Caribbean)

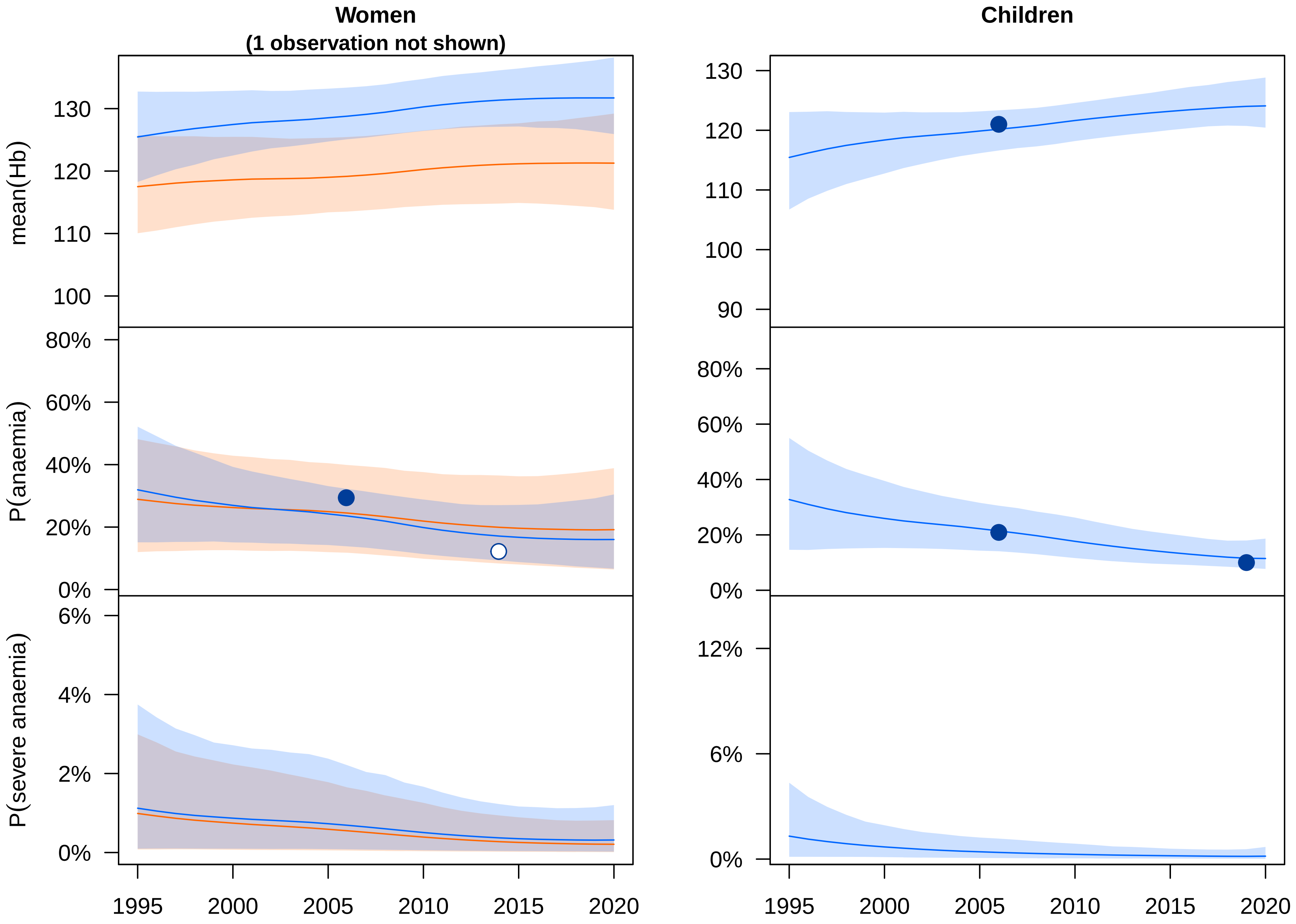

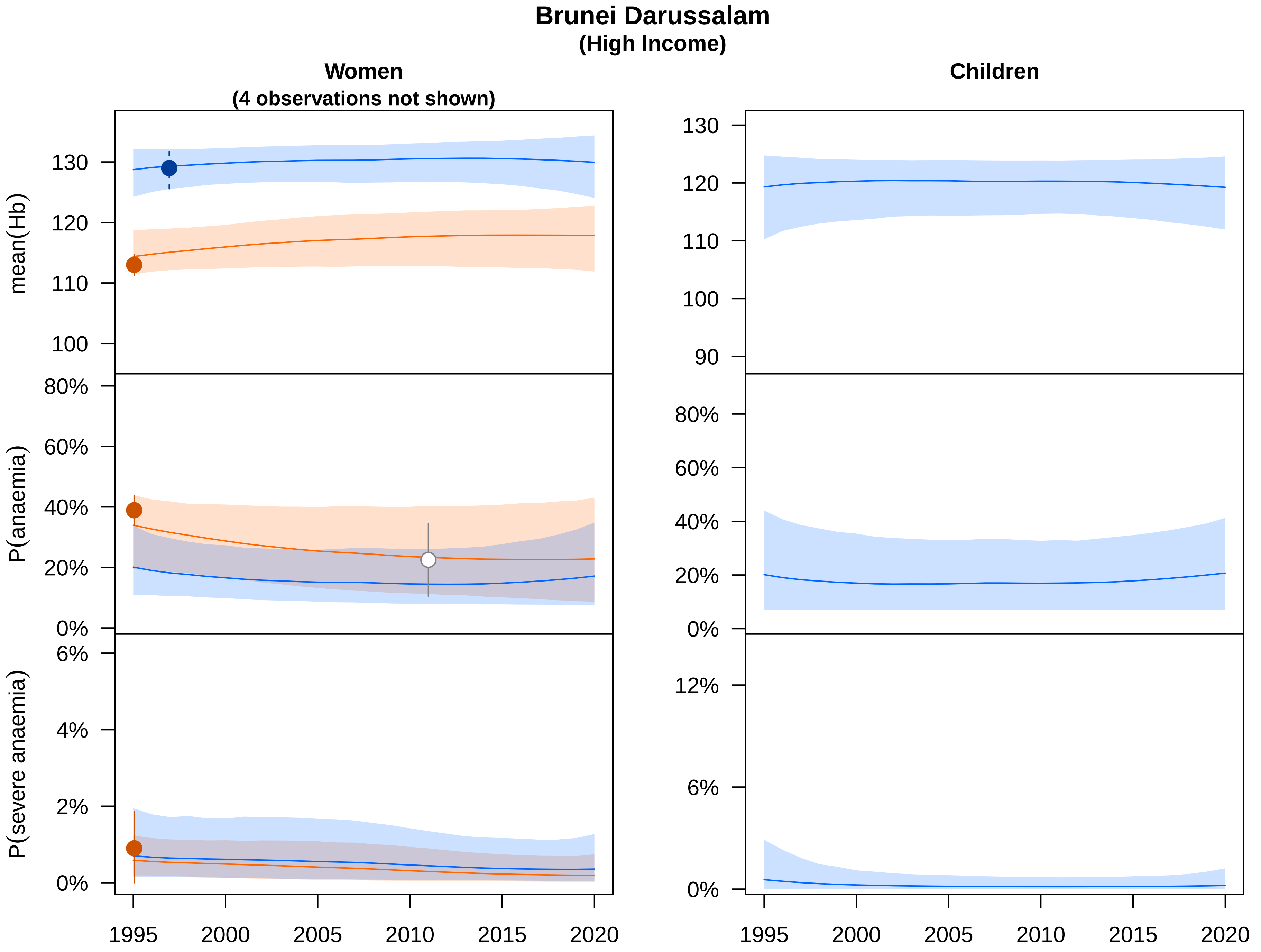

Brunei Darussalam
(High Income)
Women
(4 observations not shown)
Children
mean(Hb)
P(anaemia)
P(severe anaemia)
130
120
110
100
90
80%
60%
40%
20%
0%
6%
4%
2%
12%
1995
2000
2005
2010
2015
2020

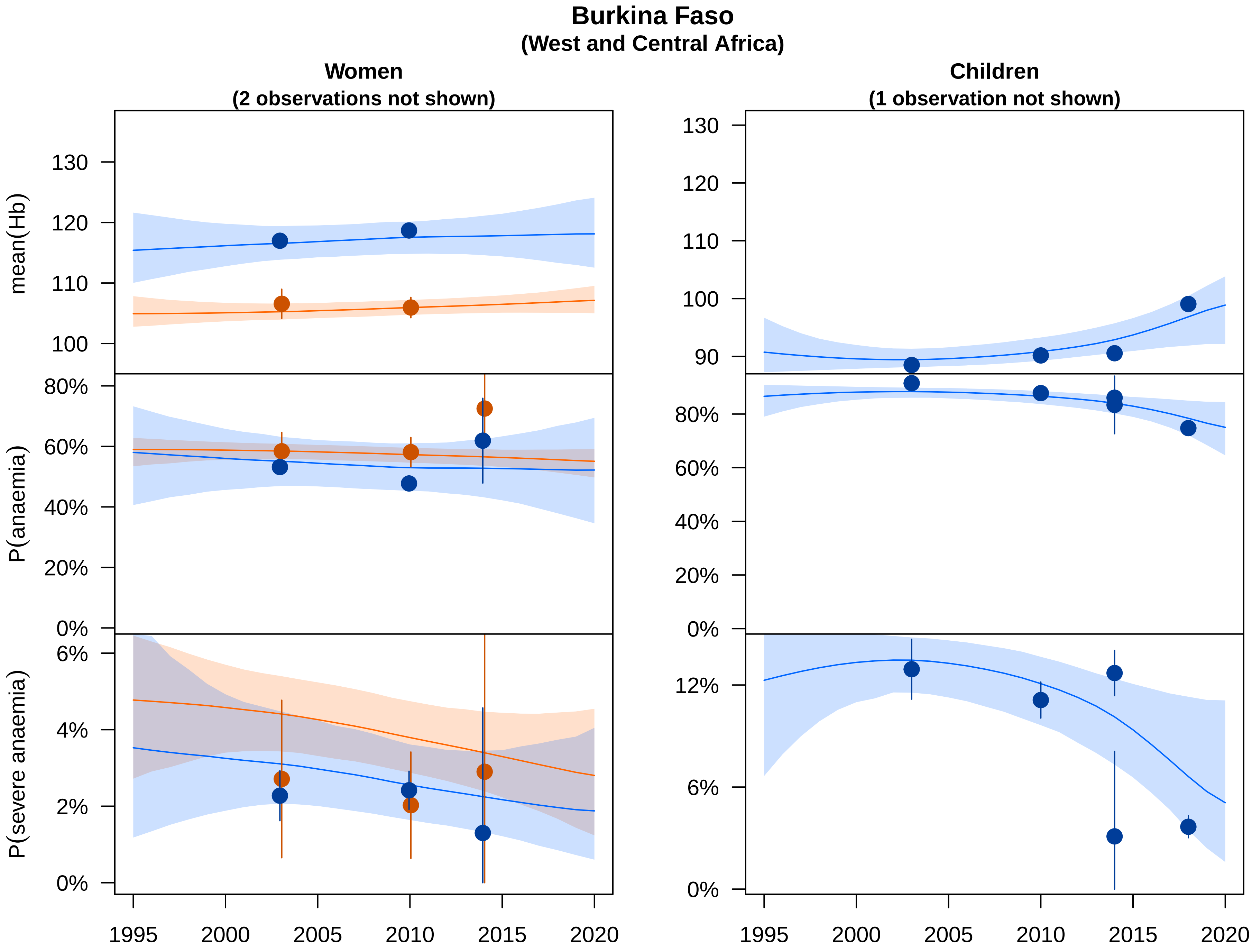

Burkina Faso
(West and Central Africa)
Women
(2 observations not shown)
Children
(1 observation not shown)
mean(Hb)
P(anaemia)
P(severe anaemia)
130
120
110
100
90
80%
60%
40%
20%
0%
6%
4%
2%
12%
1995
2000
2005
2010
2015
2020

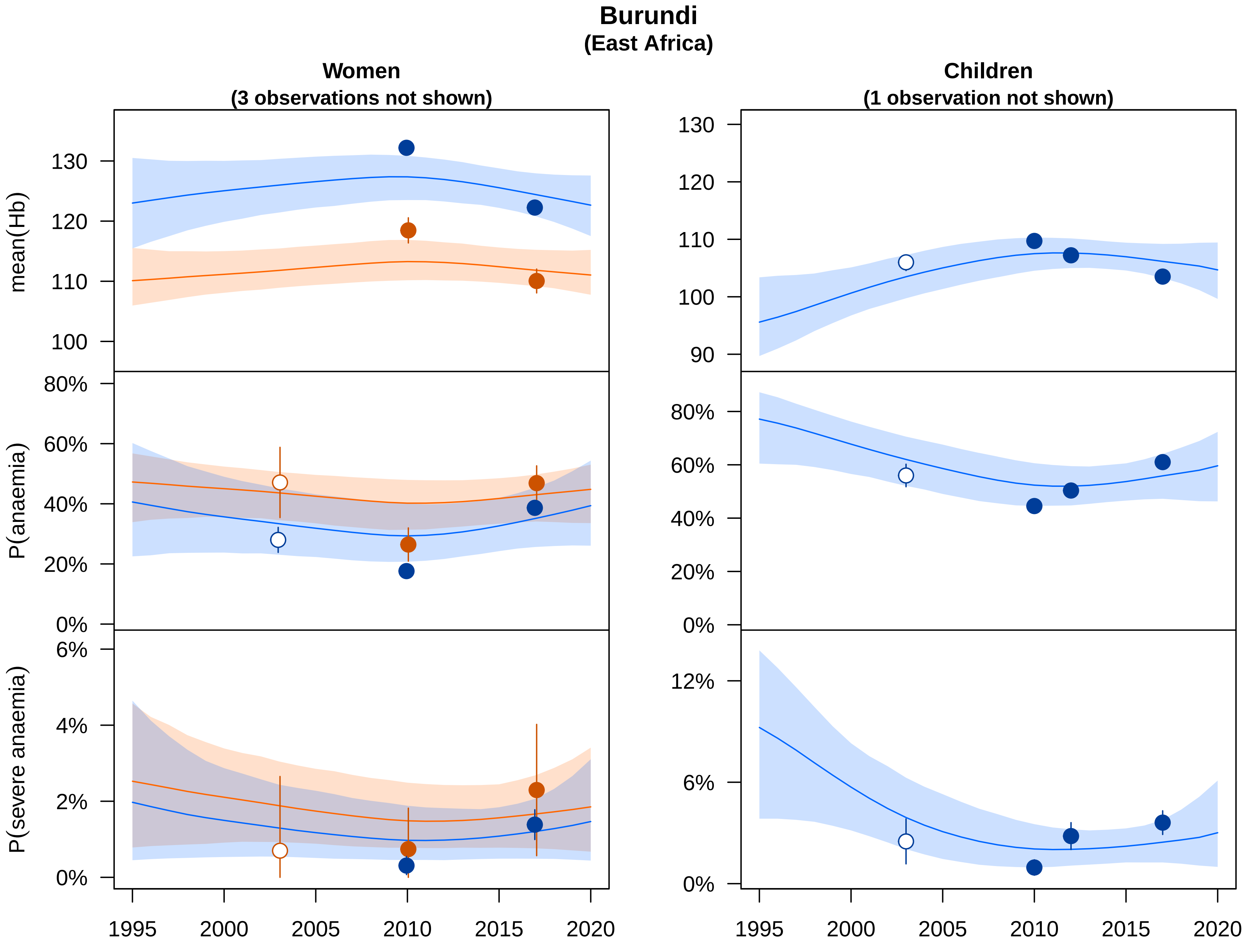

Burundi
(East Africa)
Women
(3 observations not shown)
Children
(1 observation not shown)
mean(Hb)
P(anaemia)
P(severe anaemia)
130
120
110
100
90
80%
60%
40%
20%
0%
6%
4%
2%
12%
1995
2000
2005
2010
2015
2020

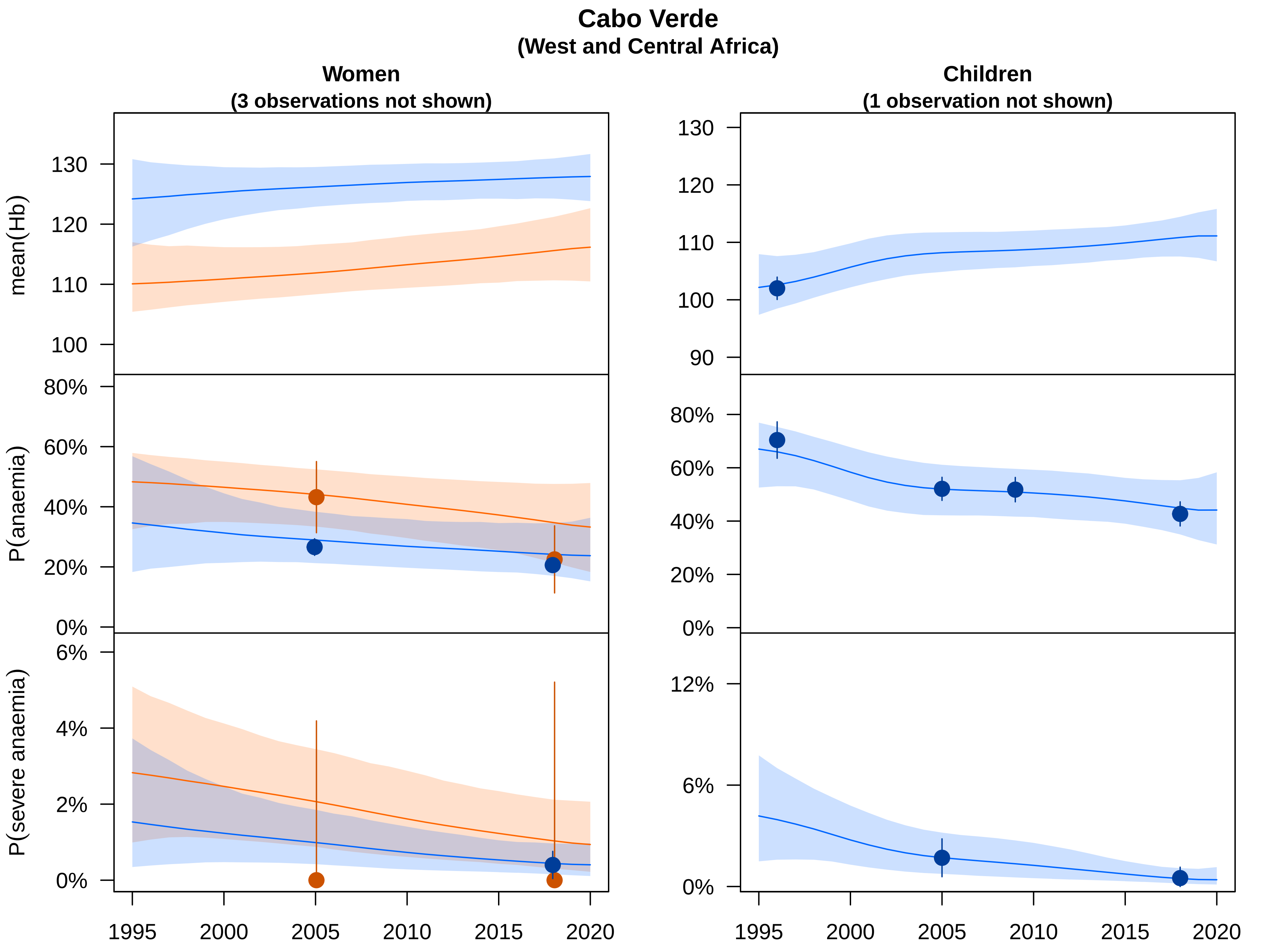

Cabo Verde
(West and Central Africa)
Women
(3 observations not shown)
Children
(1 observation not shown)
mean(Hb)
P(anaemia)
P(severe anaemia)
130
120
110
100
90
80%
60%
40%
20%
0%
6%
4%
2%
12%
1995
2000
2005
2010
2015
2020

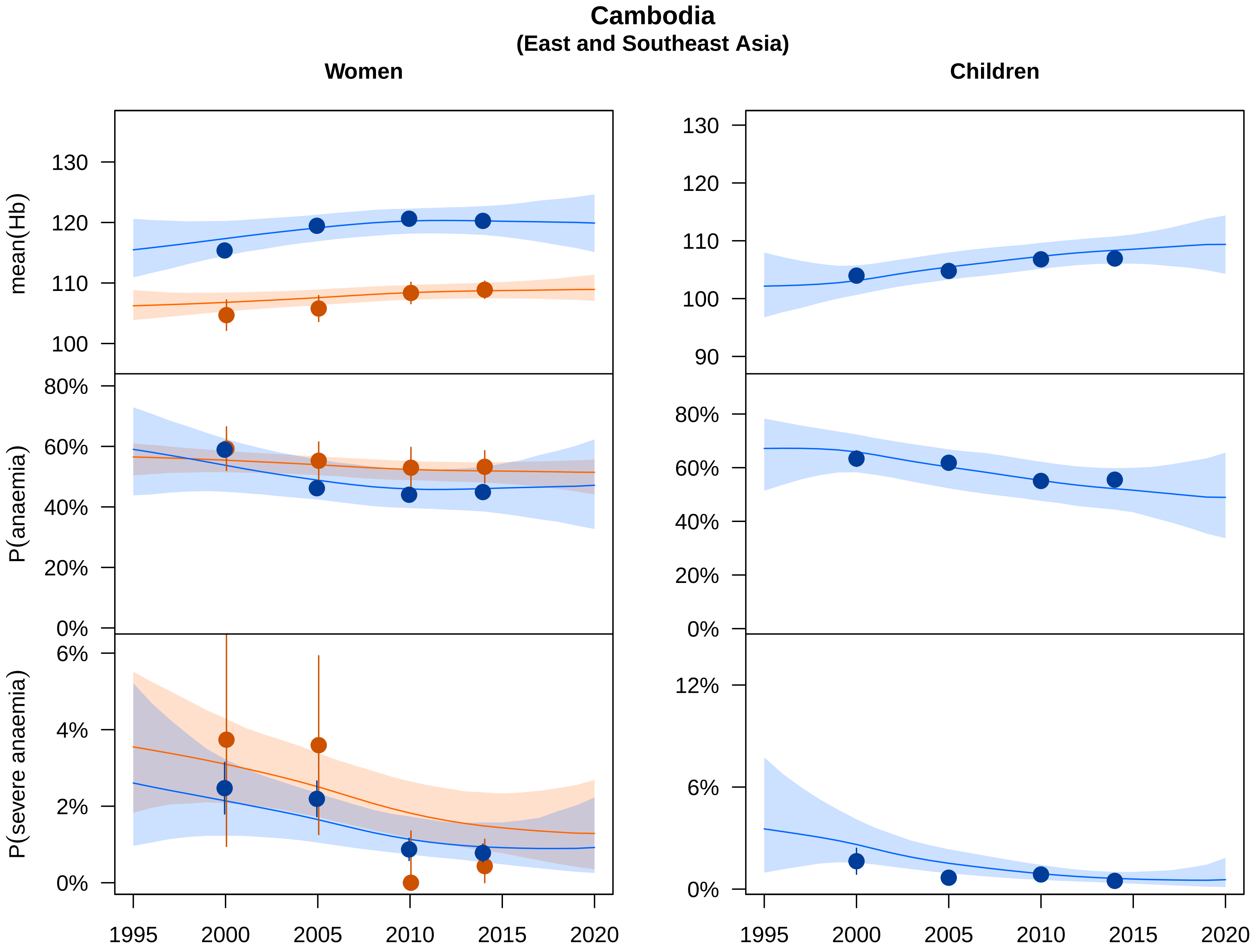

Cambodia
(East and Southeast Asia)
Women
Children
mean(Hb)
P(anaemia)
P(severe anaemia)
130
120
110
100
90
80%
60%
40%
20%
0%
6%
4%
2%
12%
1995
2000
2005
2010
2015
2020

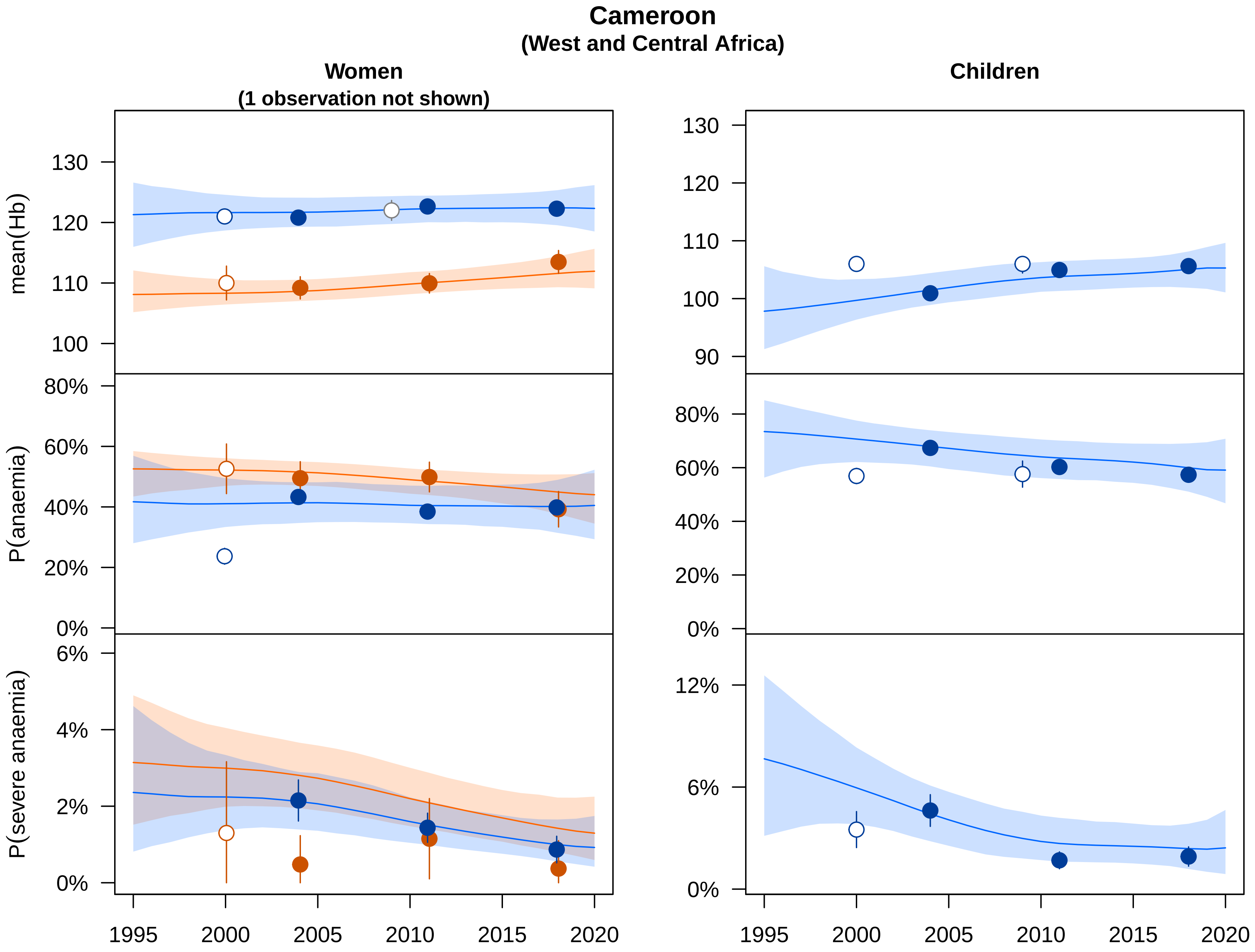
Cameroon
(West and Central Africa)
Women
(1 observation not shown)
Children
mean(Hb)
P(anaemia)
P(severe anaemia)
130
120
110
100
90
80%
60%
40%
20%
0%
6%
4%
2%
12%
1995
2000
2005
2010
2015
2020

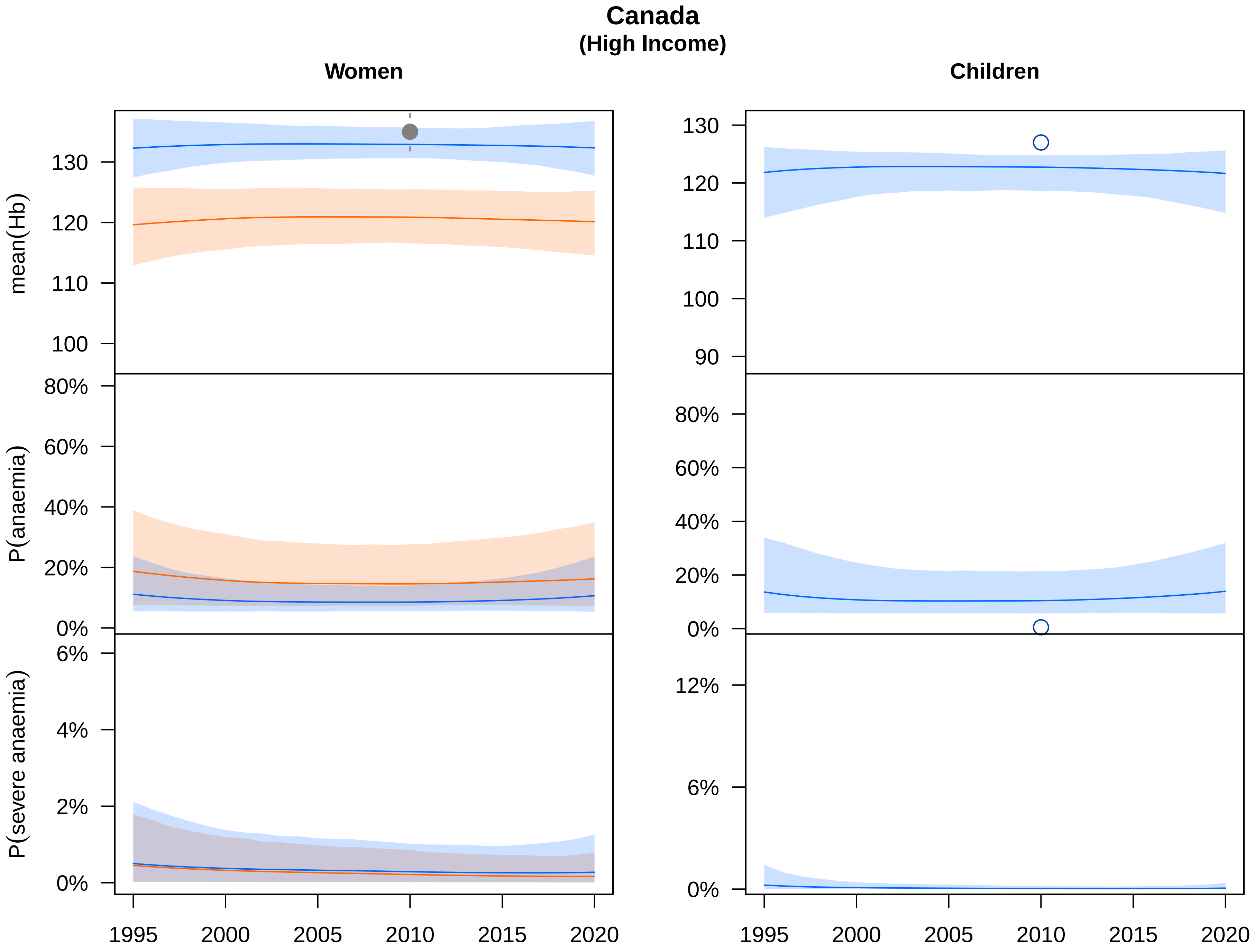

Canada
(High Income)
Women
Children
mean(Hb)
P(anaemia)
P(severe anaemia)
130
120
110
100
90
80%
60%
40%
20%
0%
6%
4%
2%
12%
1995
2000
2005
2010
2015
2020

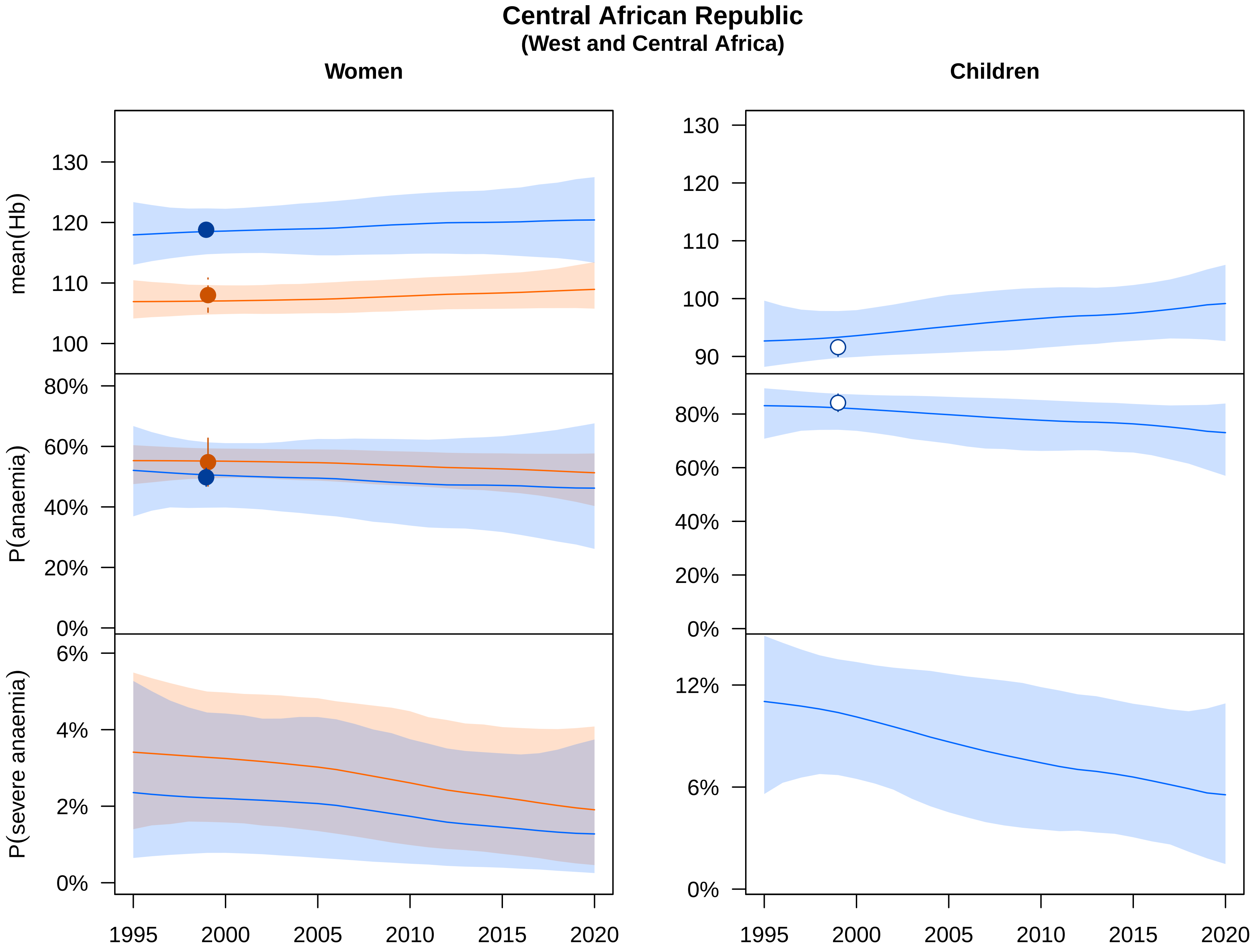
Central African Republic
(West and Central Africa)
Women
Children
mean(Hb)
P(anaemia)
P(severe anaemia)
130
120
110
100
90
80%
60%
40%
20%
0%
6%
4%
2%
12%
1995
2000
2005
2010
2015
2020

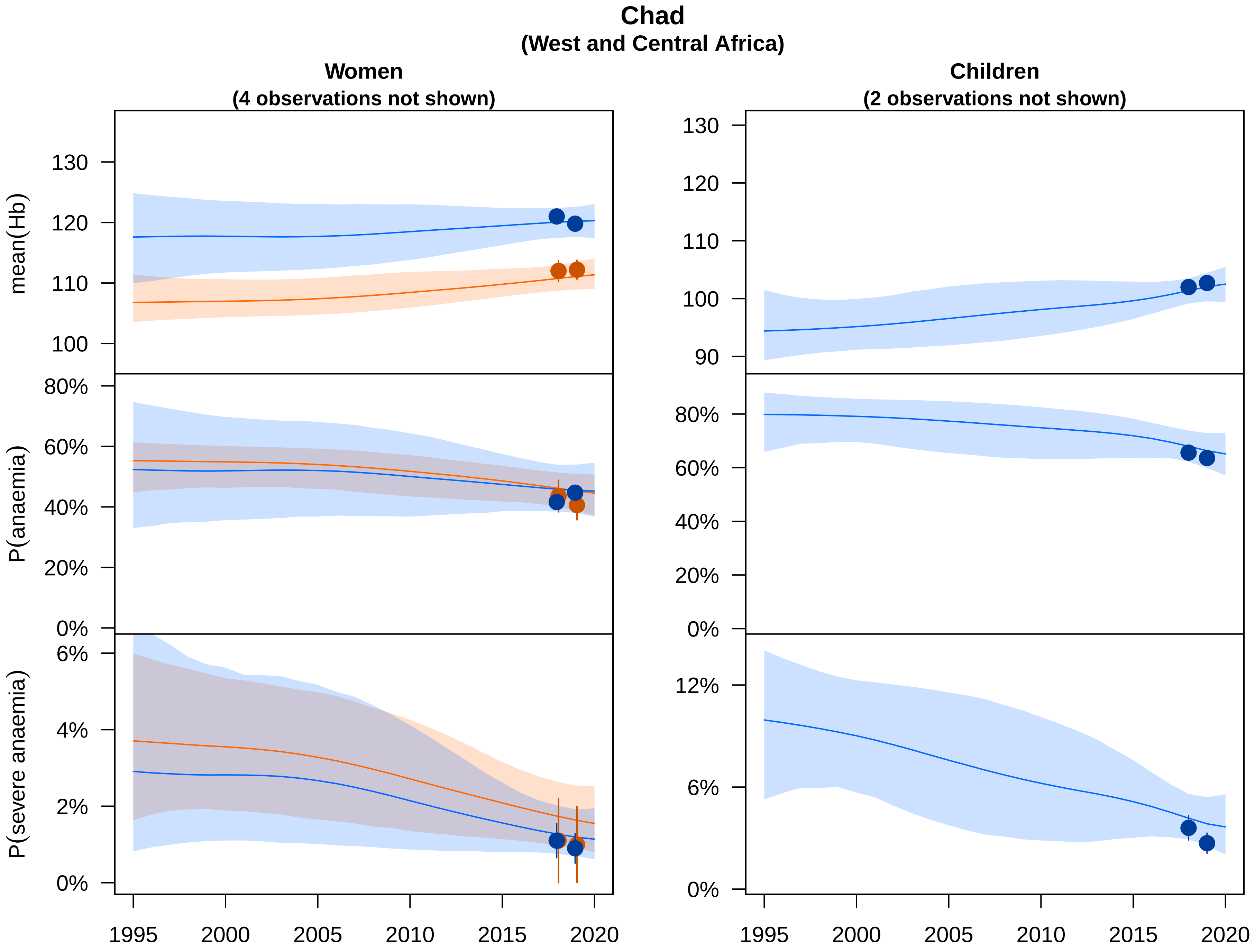

Chad
(West and Central Africa)
Women
(4 observations not shown)
Children
(2 observations not shown)
mean(Hb)
P(anaemia)
P(severe anaemia)
130
120
110
100
90
80%
60%
40%
20%
0%
6%
4%
2%
12%
1995
2000
2005
2010
2015
2020

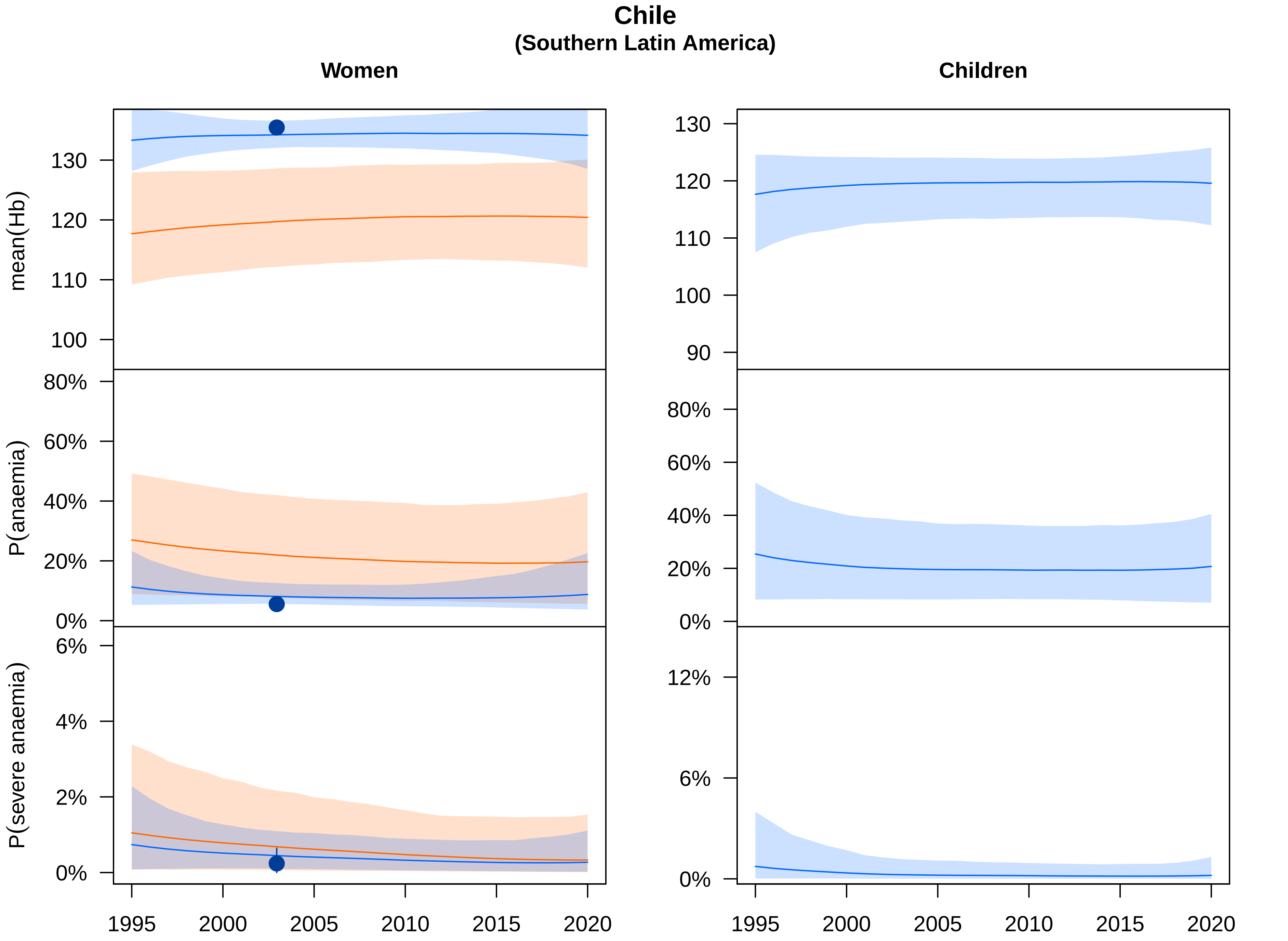

Chile
(Southern Latin America)
Women
Children
mean(Hb)
P(anaemia)
P(severe anaemia)
130
120
110
100
90
80%
60%
40%
20%
0%
6%
4%
2%
12%
1995
2000
2005
2010
2015
2020

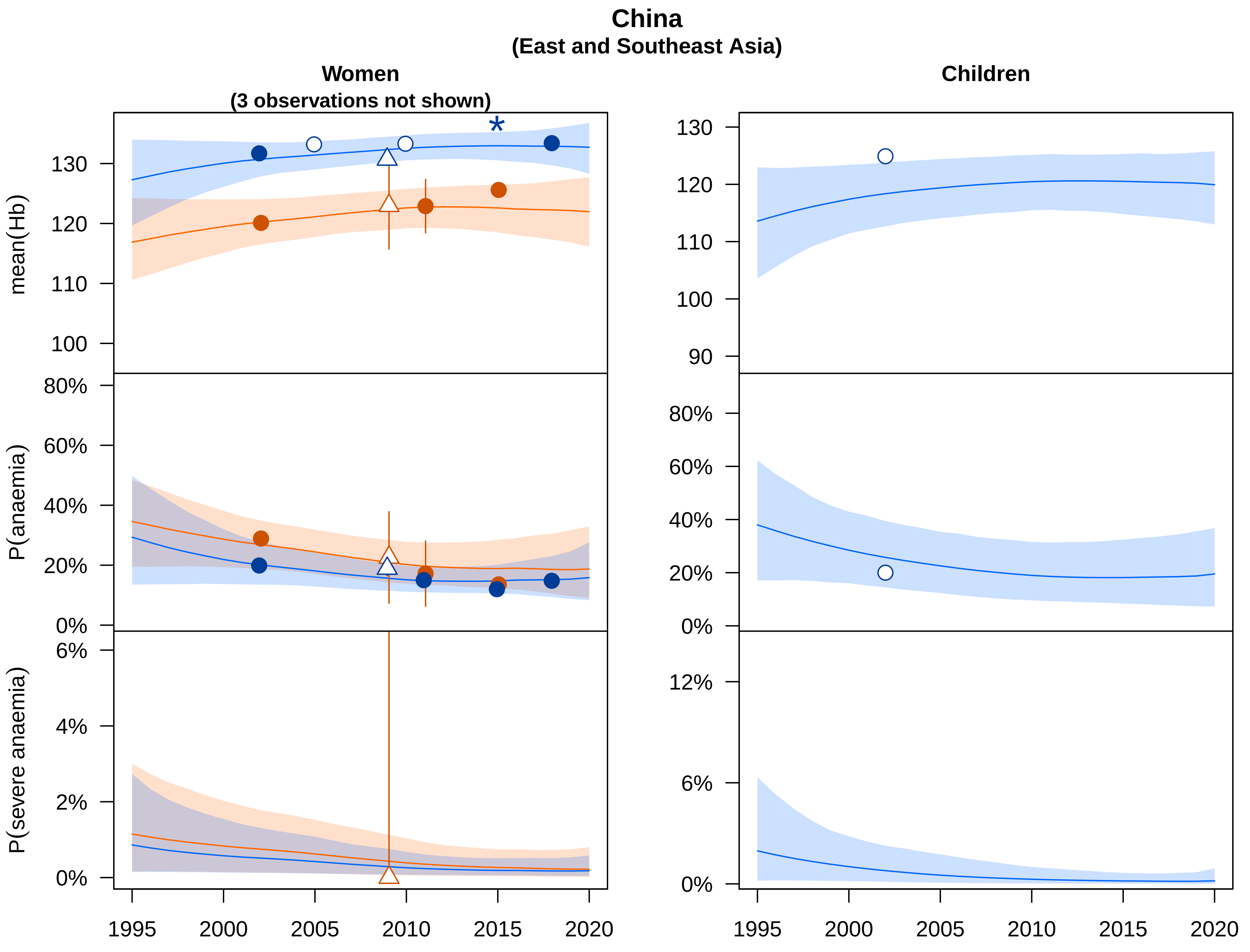
China
(East and Southeast Asia)
Women
(3 observations not shown)
Children
mean(Hb)
P(anaemia)
P(severe anaemia)
130
120
110
100
90
80%
60%
40%
20%
0%
6%
4%
2%
12%
1995
2000
2005
2010
2015
2020

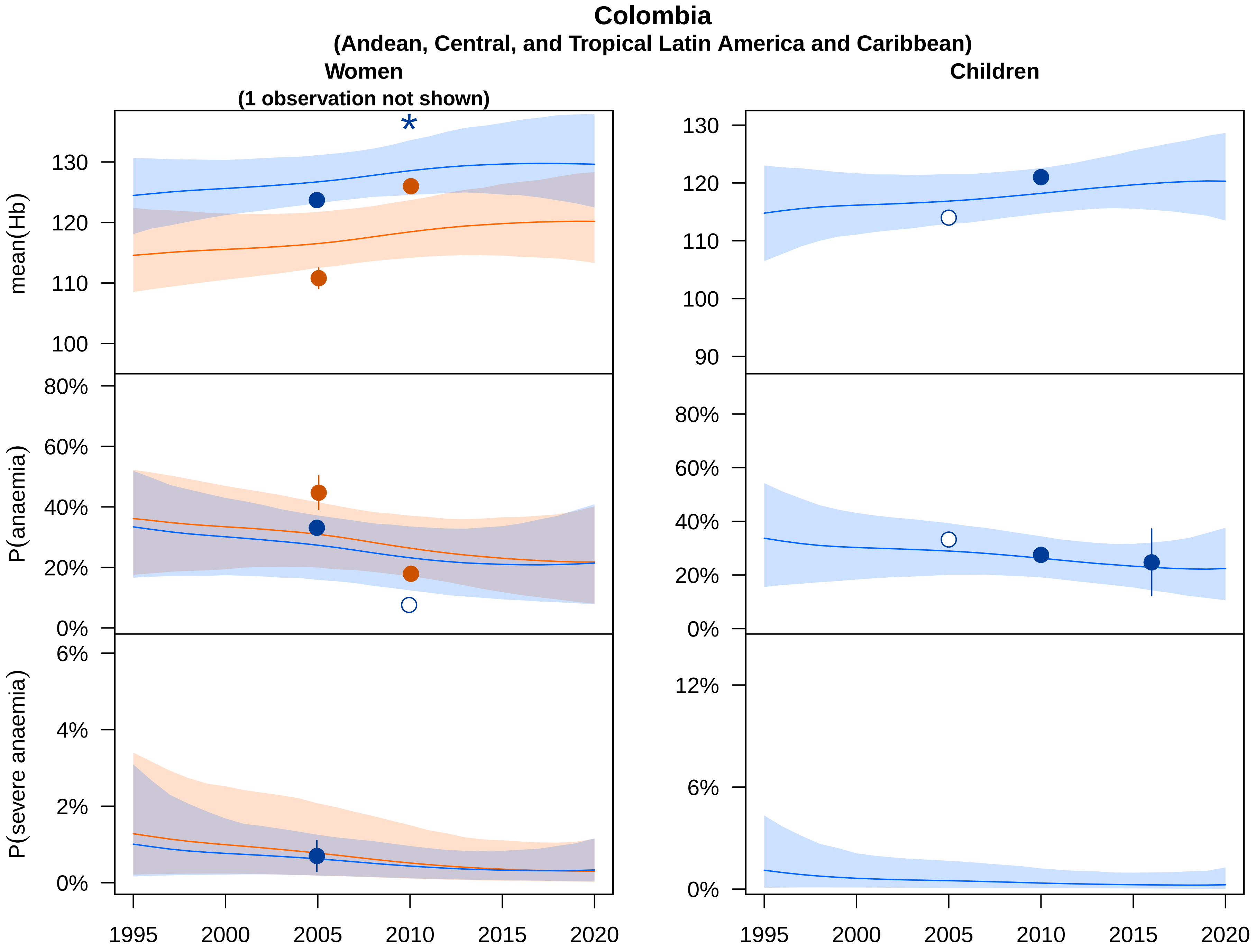
Colombia
(Andean, Central, and Tropical Latin America and Caribbean)
Women
(1 observation not shown)
Children
mean(Hb)
P(anaemia)
P(severe anaemia)
130
120
110
100
90
0%
20%
40%
60%
80%
2%
4%
6%
12%
1995
2000
2005
2010
2015
2020

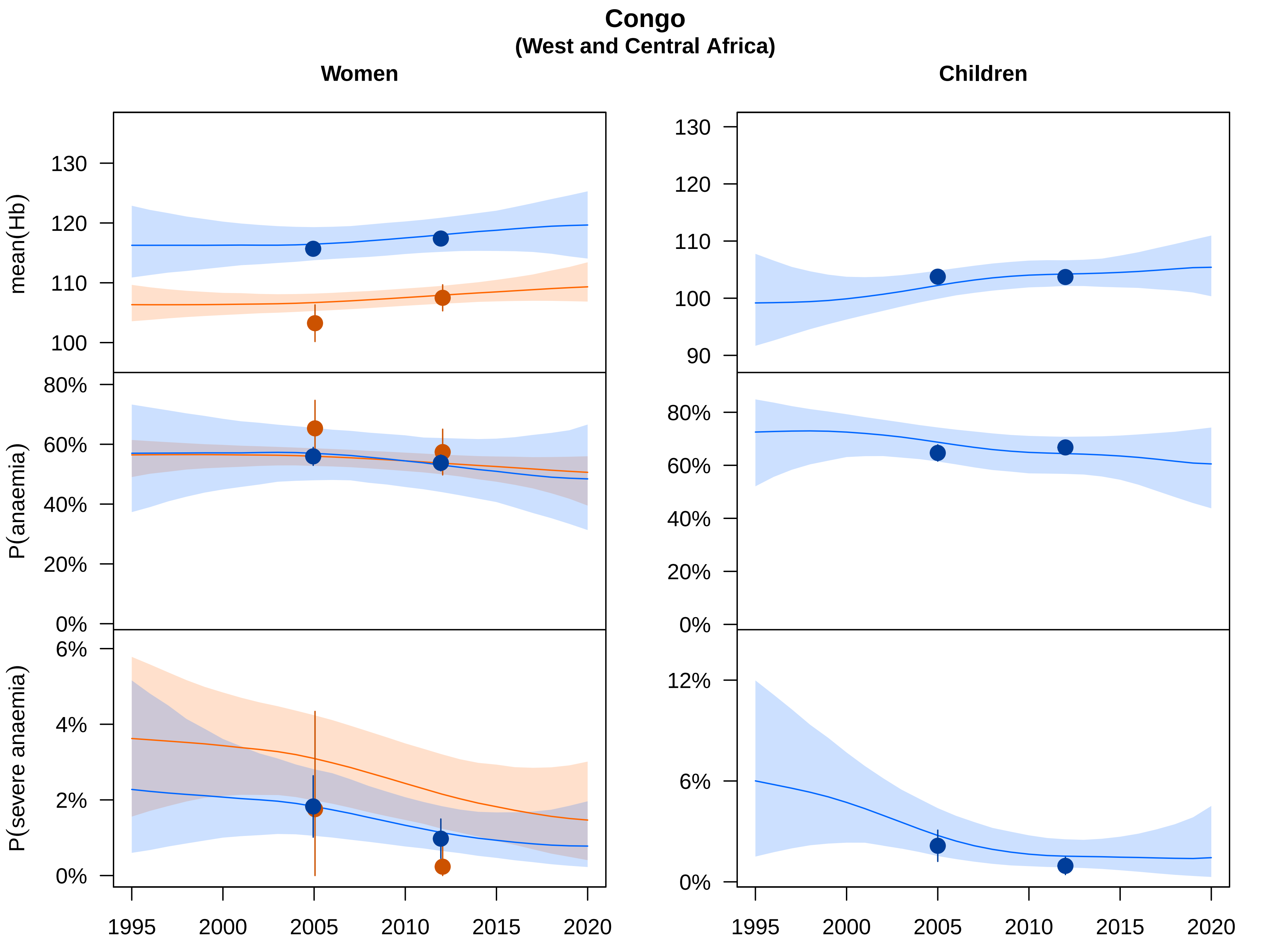

Congo
(West and Central Africa)
Women
Children
mean(Hb)
P(anaemia)
P(severe anaemia)
130
120
110
100
90
80%
60%
40%
20%
0%
6%
4%
2%
12%
1995
2000
2005
2010
2015
2020

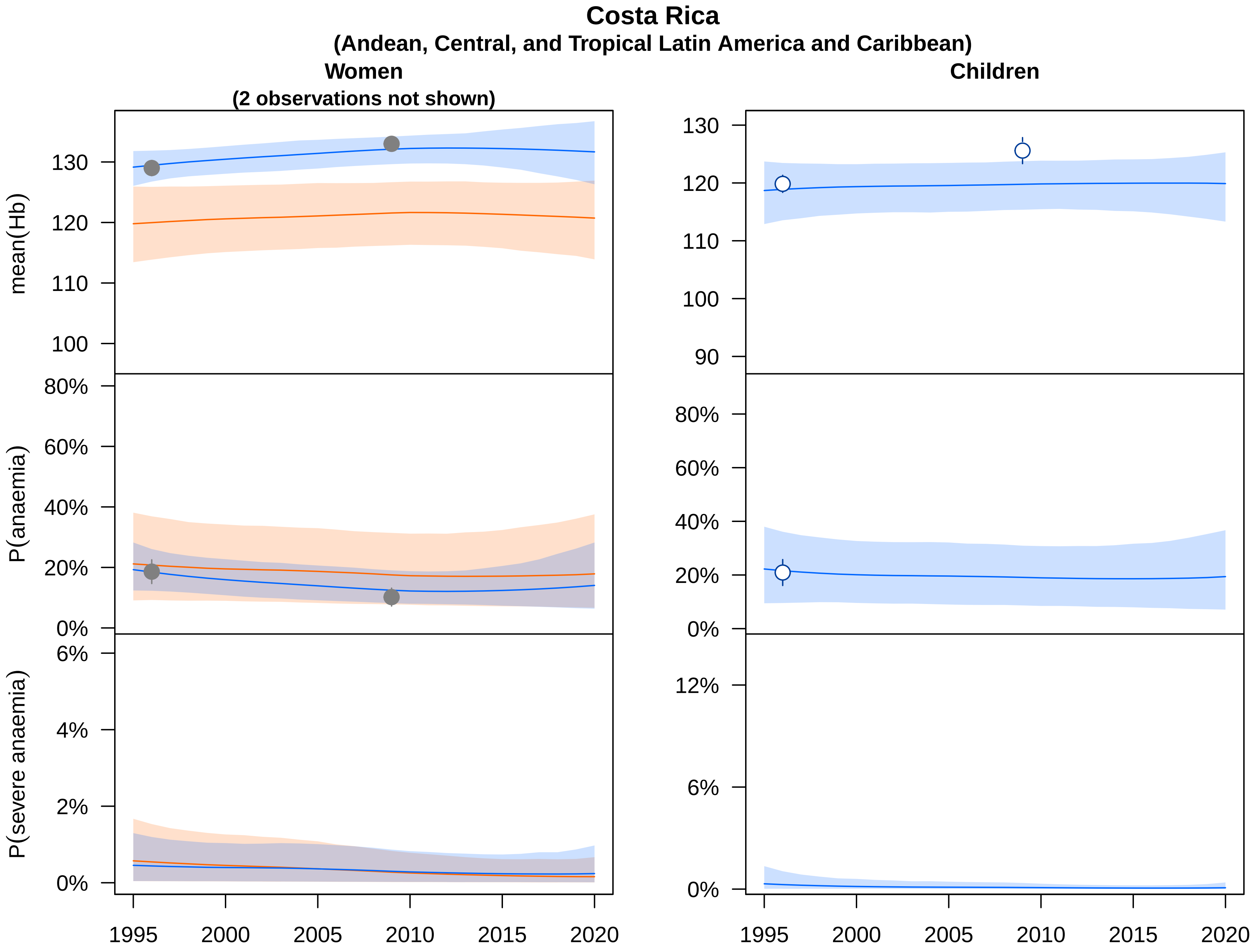

Costa Rica
(Andean, Central, and Tropical Latin America and Caribbean)
Women
(2 observations not shown)
Children
mean(Hb)
P(anaemia)
P(severe anaemia)
130
120
110
100
90
80%
60%
40%
20%
0%
6%
4%
2%
12%
1995
2000
2005
2010
2015
2020

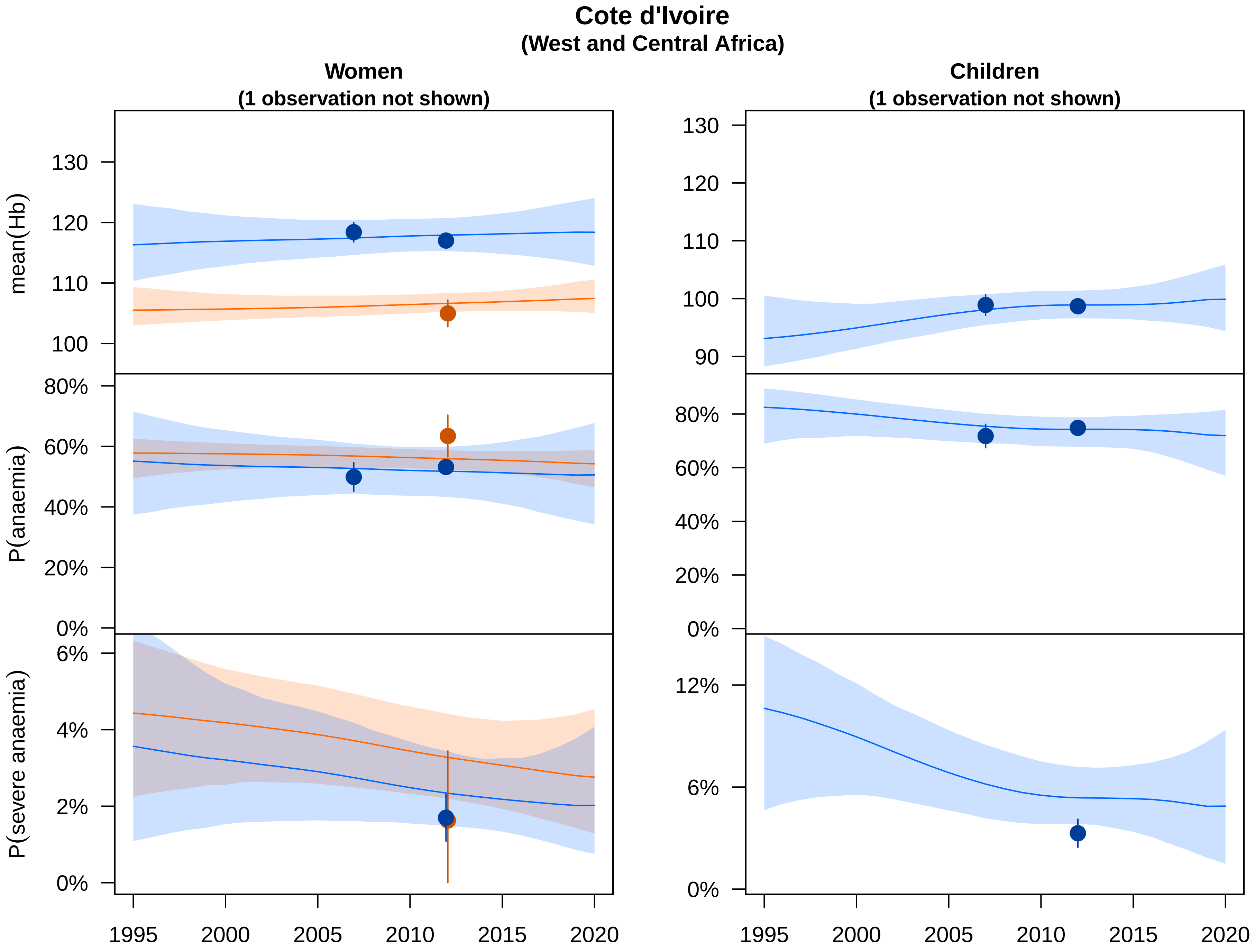

Cote d'Ivoire
(West and Central Africa)
Women
(1 observation not shown)
Children
(1 observation not shown)
mean(Hb)
P(anaemia)
P(severe anaemia)
130
120
110
100
90
80%
60%
40%
20%
0%
6%
4%
2%
12%
1995
2000
2005
2010
2015
2020

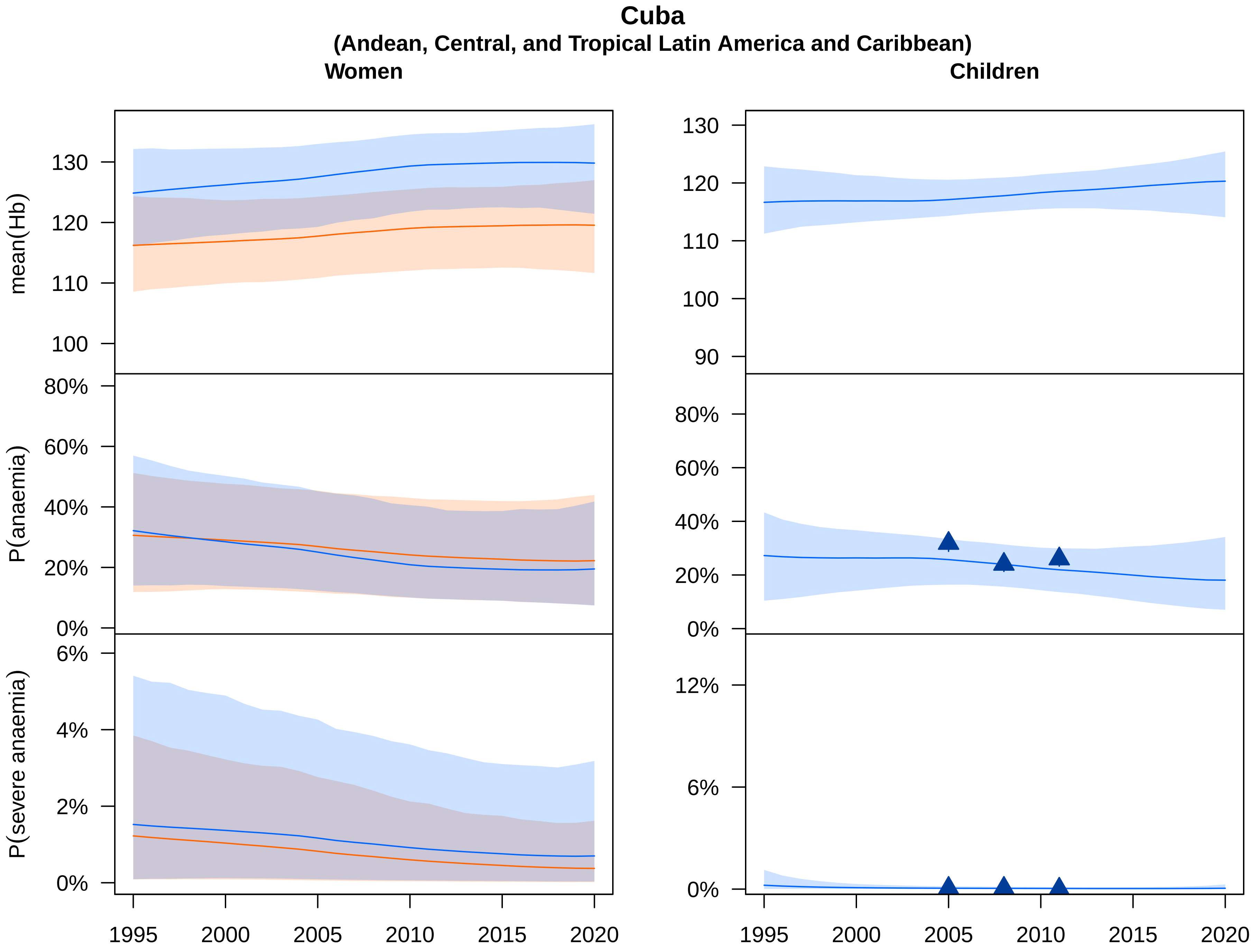

Cuba
(Andean, Central, and Tropical Latin America and Caribbean)
Women
Children
mean(Hb)
P(anaemia)
P(severe anaemia)
130
120
110
100
90
80%
60%
40%
20%
0%
6%
4%
2%
12%
1995
2000
2005
2010
2015
2020

Democratic People's Republic of Korea
(East and Southeast Asia)

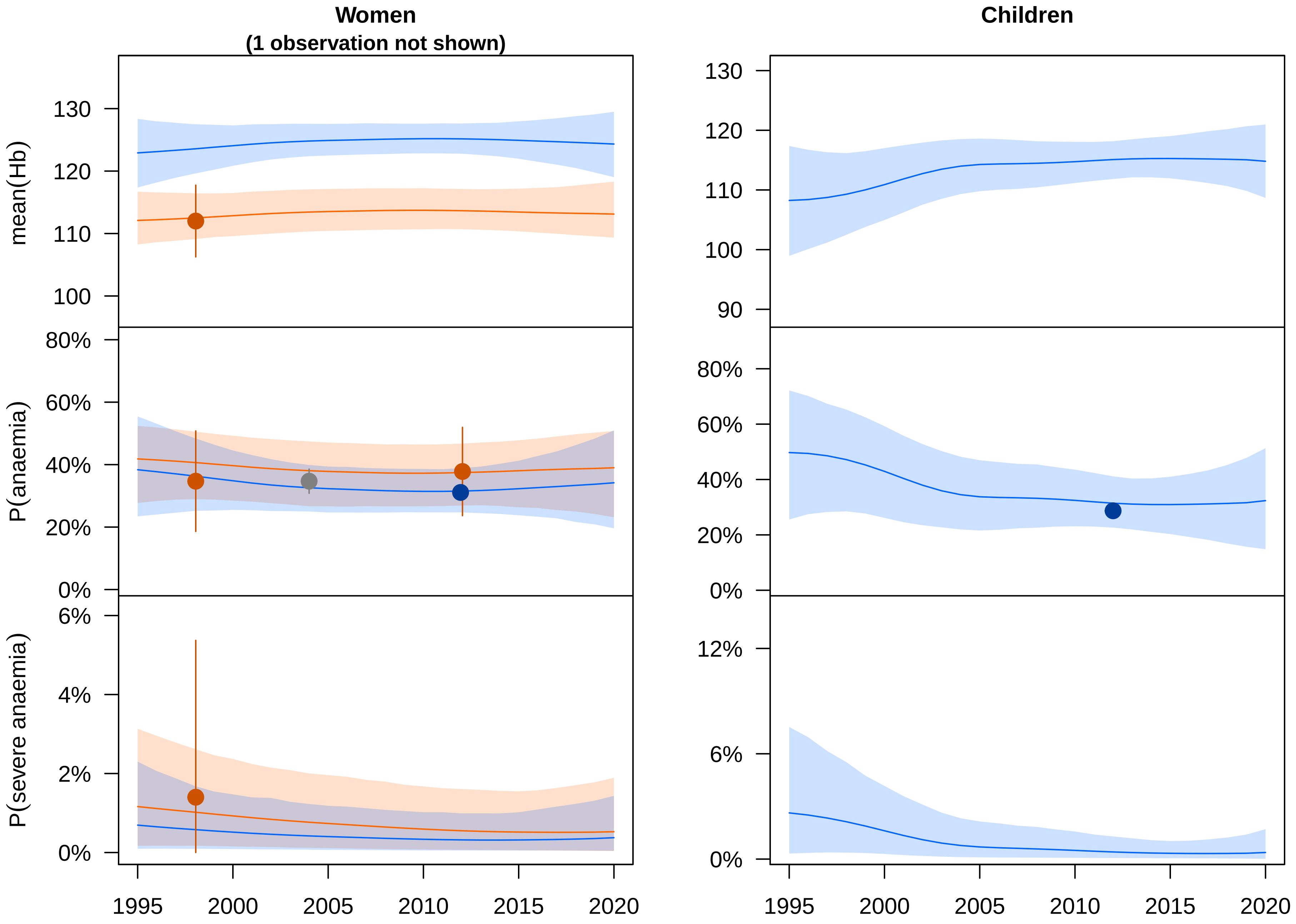

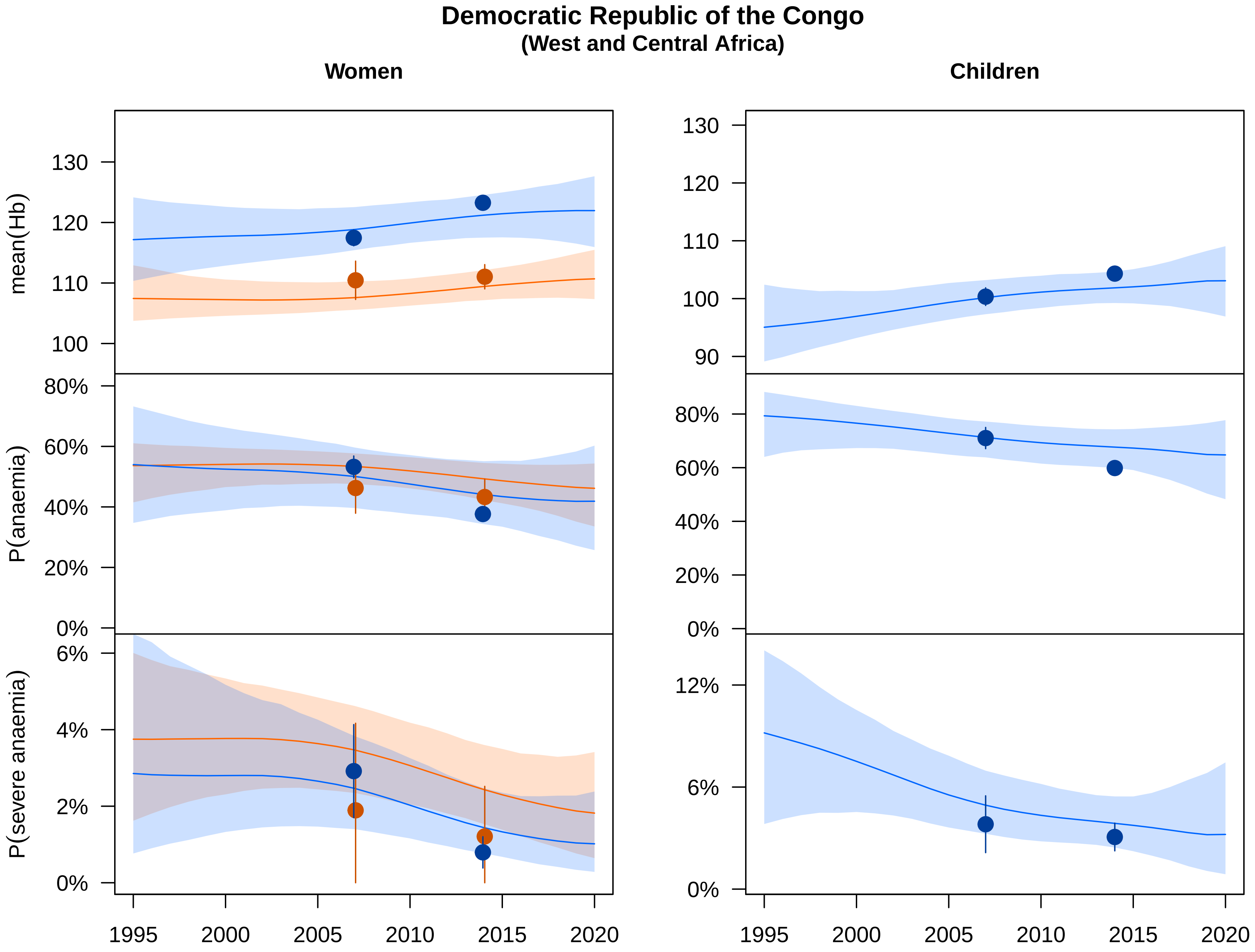

Democratic Republic of the Congo
(West and Central Africa)
Women
Children
mean(Hb)
P(anaemia)
P(severe anaemia)
130
120
110
100
90
80%
60%
40%
20%
0%
6%
4%
2%
12%
1995
2000
2005
2010
2015
2020

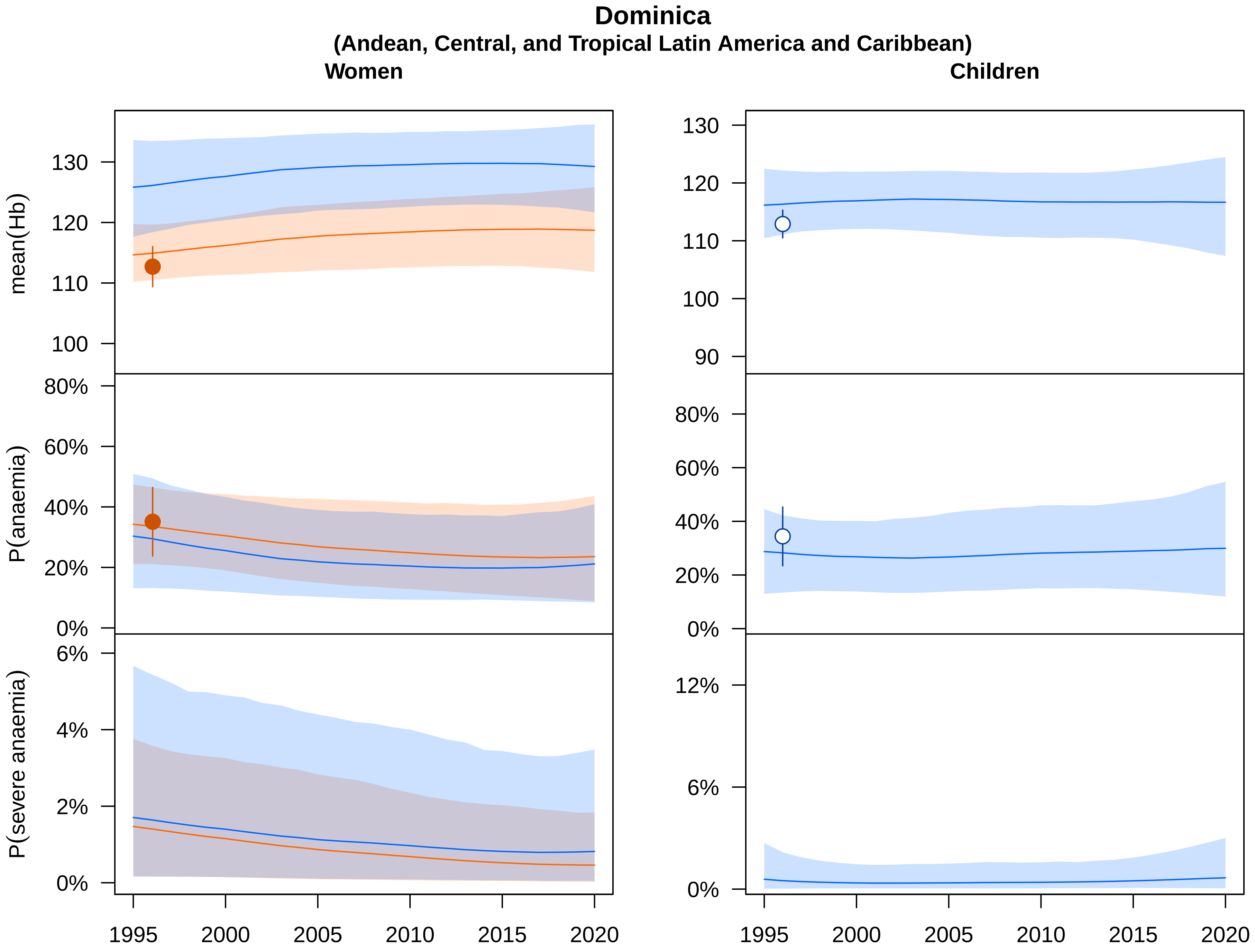

Dominica
(Andean, Central, and Tropical Latin America and Caribbean)
Women
Children
mean(Hb)
P(anaemia)
P(severe anaemia)
130
120
110
100
90
80%
60%
40%
20%
0%
6%
4%
2%
12%
1995
2000
2005
2010
2015
2020

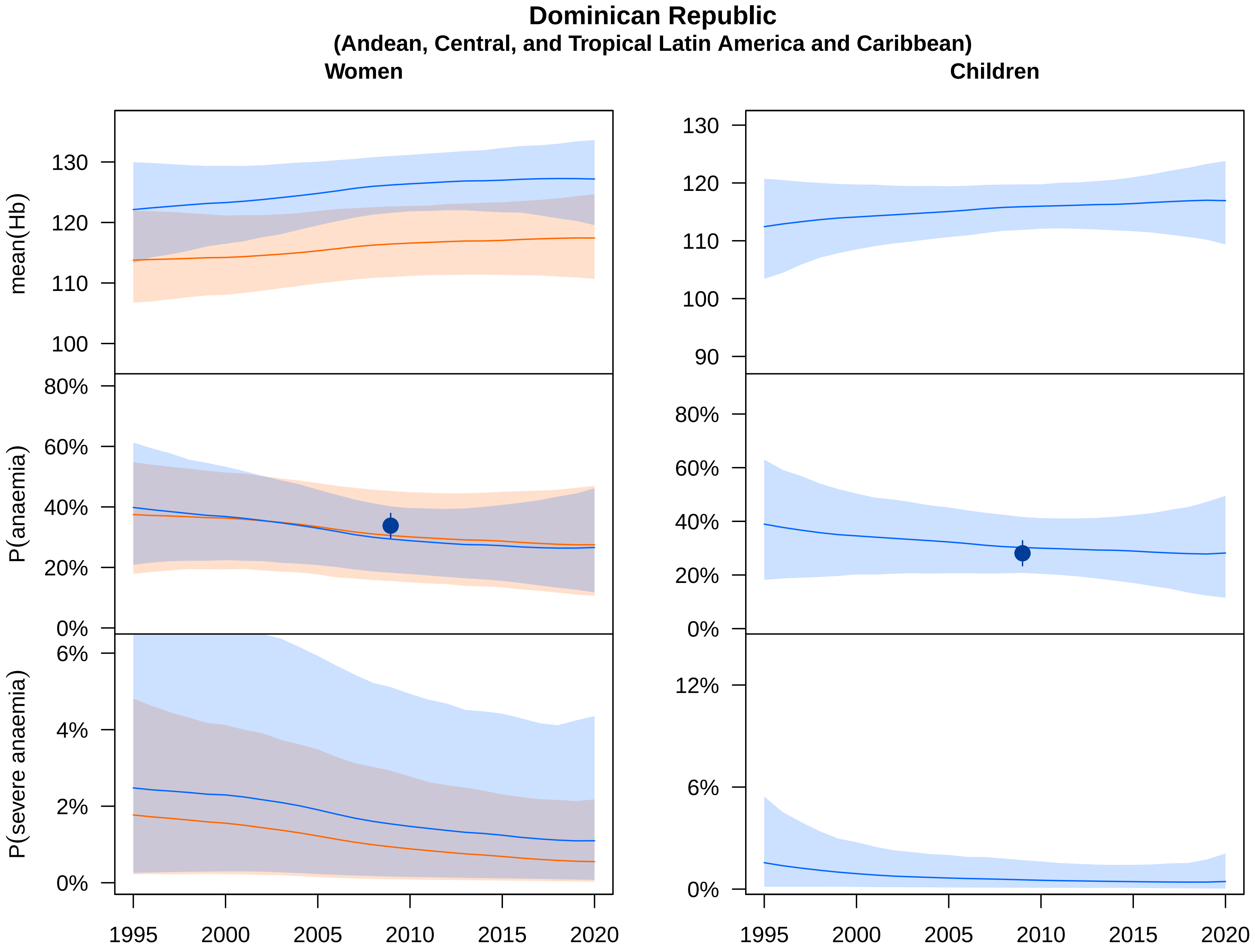
Dominican Republic
(Andean, Central, and Tropical Latin America and Caribbean)
Women
Children
mean(Hb)
P(anaemia)
P(severe anaemia)
130
120
110
100
90
80%
60%
40%
20%
0%
6%
4%
2%
12%
1995
2000
2005
2010
2015
2020

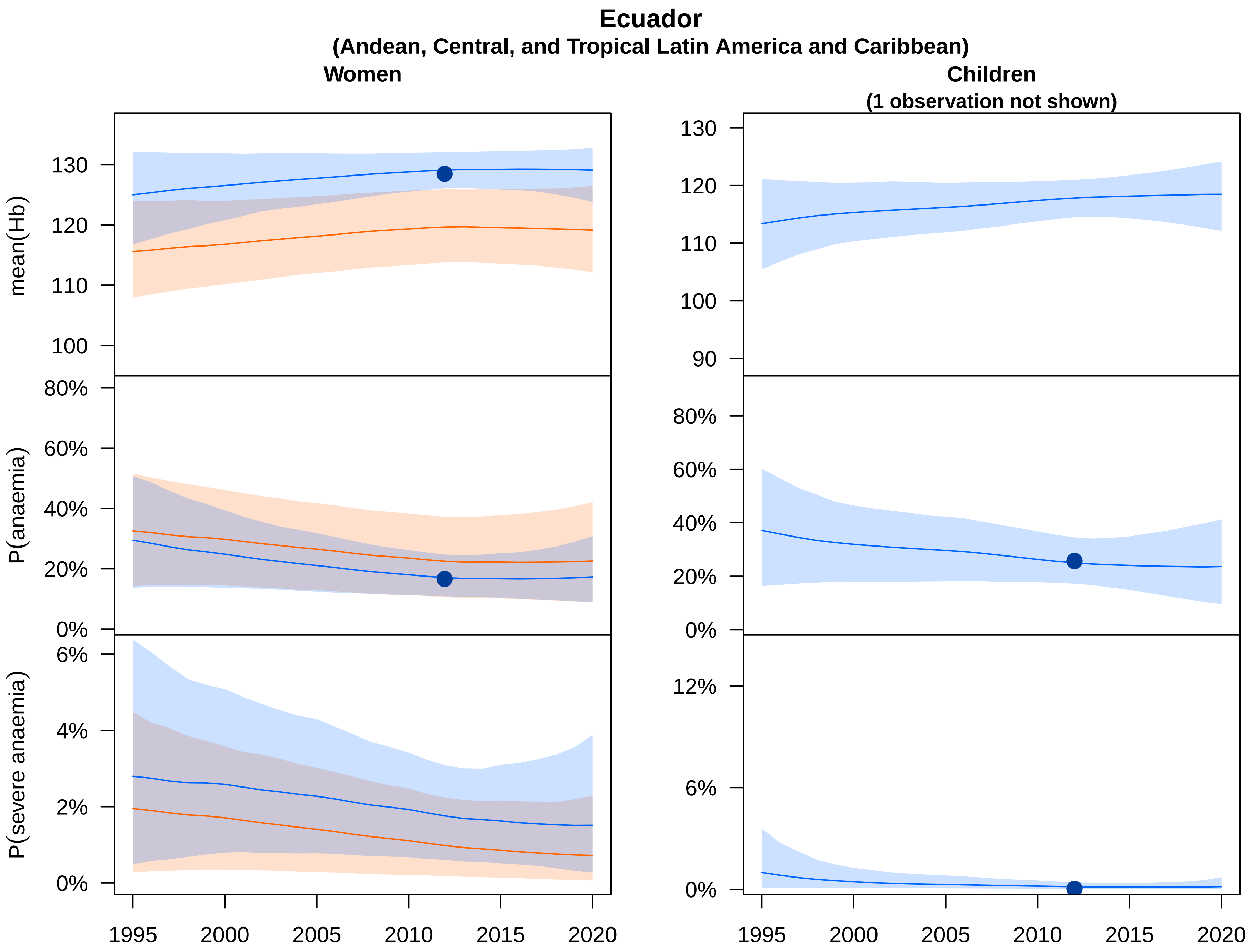
Ecuador
(Andean, Central, and Tropical Latin America and Caribbean)
Women
Children
(1 observation not shown)
mean(Hb)
P(anaemia)
P(severe anaemia)
130
120
110
100
90
80%
60%
40%
20%
0%
6%
4%
2%
12%
1995
2000
2005
2010
2015
2020

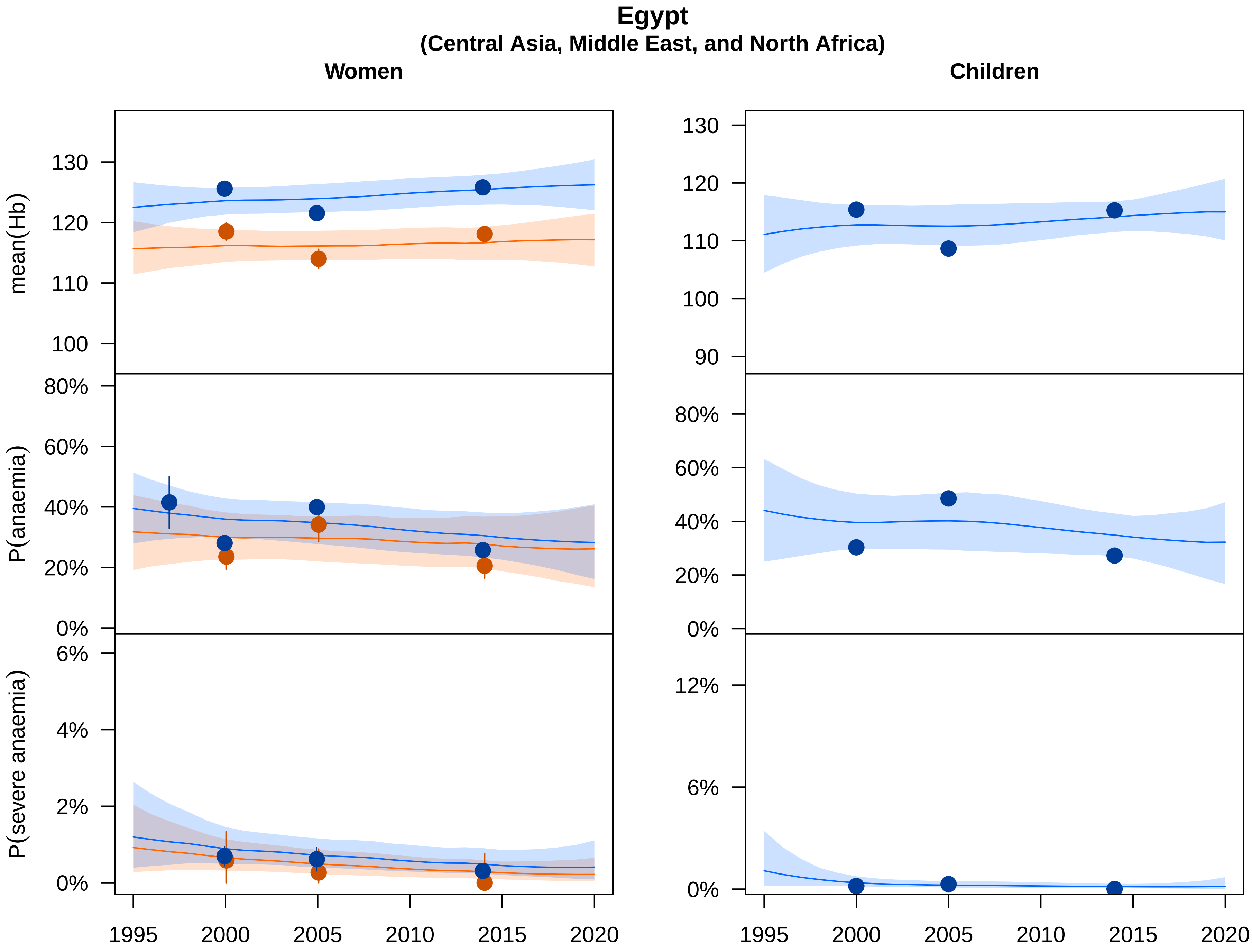

Egypt
(Central Asia, Middle East, and North Africa)
Women
Children
mean(Hb)
P(anaemia)
P(severe anaemia)
130
120
110
100
90
80%
60%
40%
20%
0%
6%
4%
2%
12%
1995
2000
2005
2010
2015
2020

El Salvador
(Andean, Central, and Tropical Latin America and Caribbean)

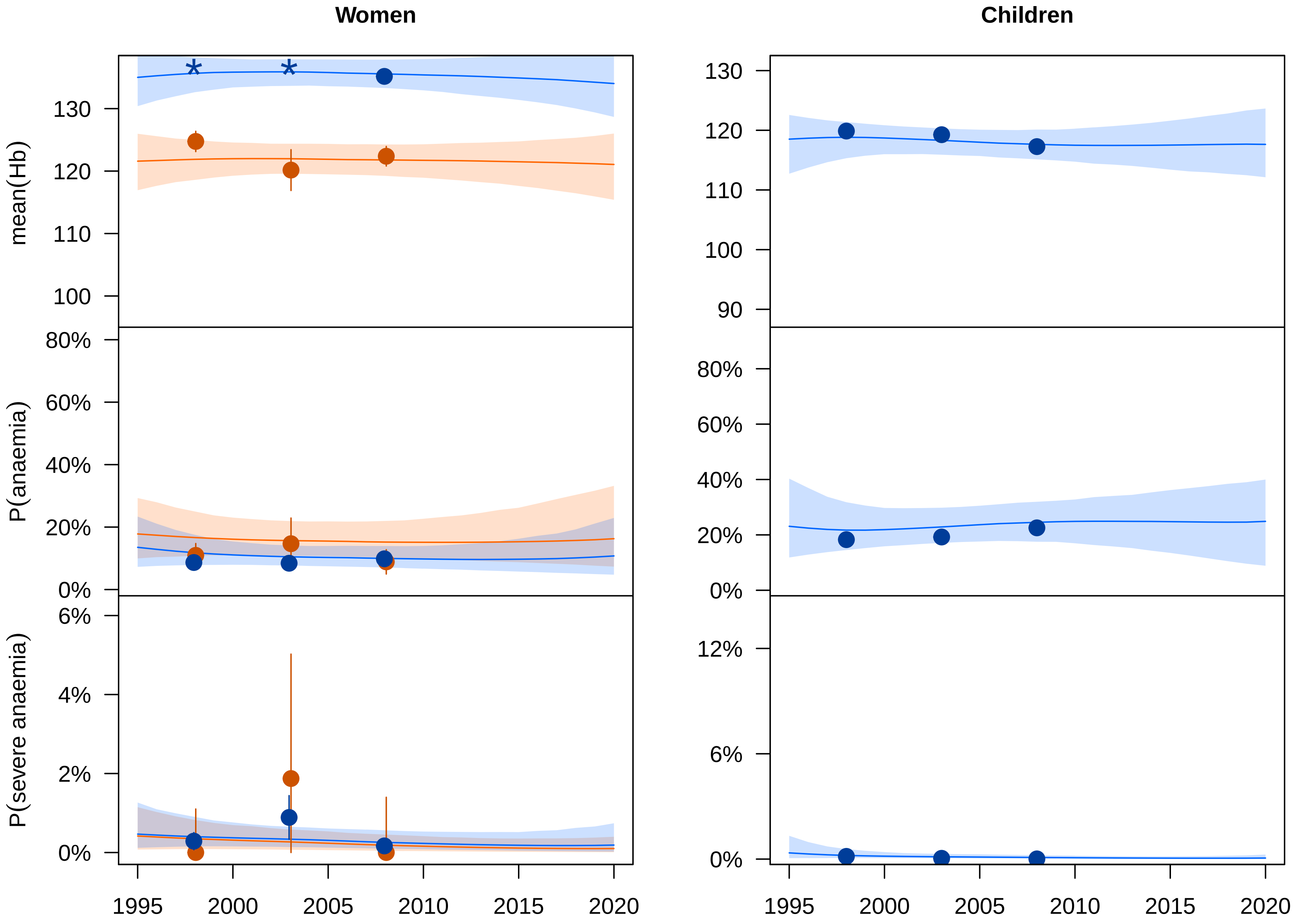

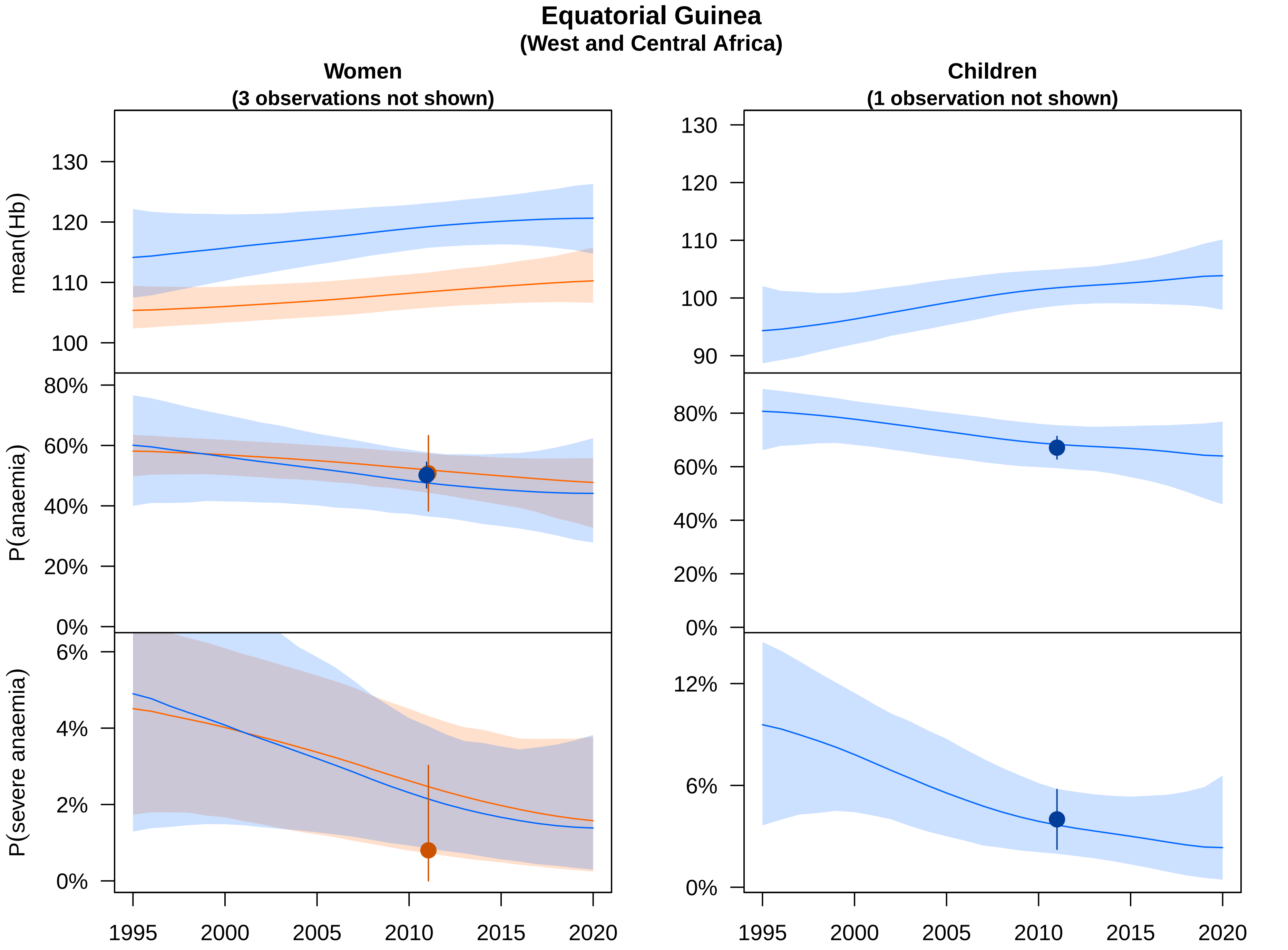
Equatorial Guinea
(West and Central Africa)
Women
(3 observations not shown)
Children
(1 observation not shown)
mean(Hb)
P(anaemia)
P(severe anaemia)
130
120
110
100
90
80%
60%
40%
20%
0%
6%
4%
2%
12%
1995
2000
2005
2010
2015
2020

Eswatini
(Southern Africa)

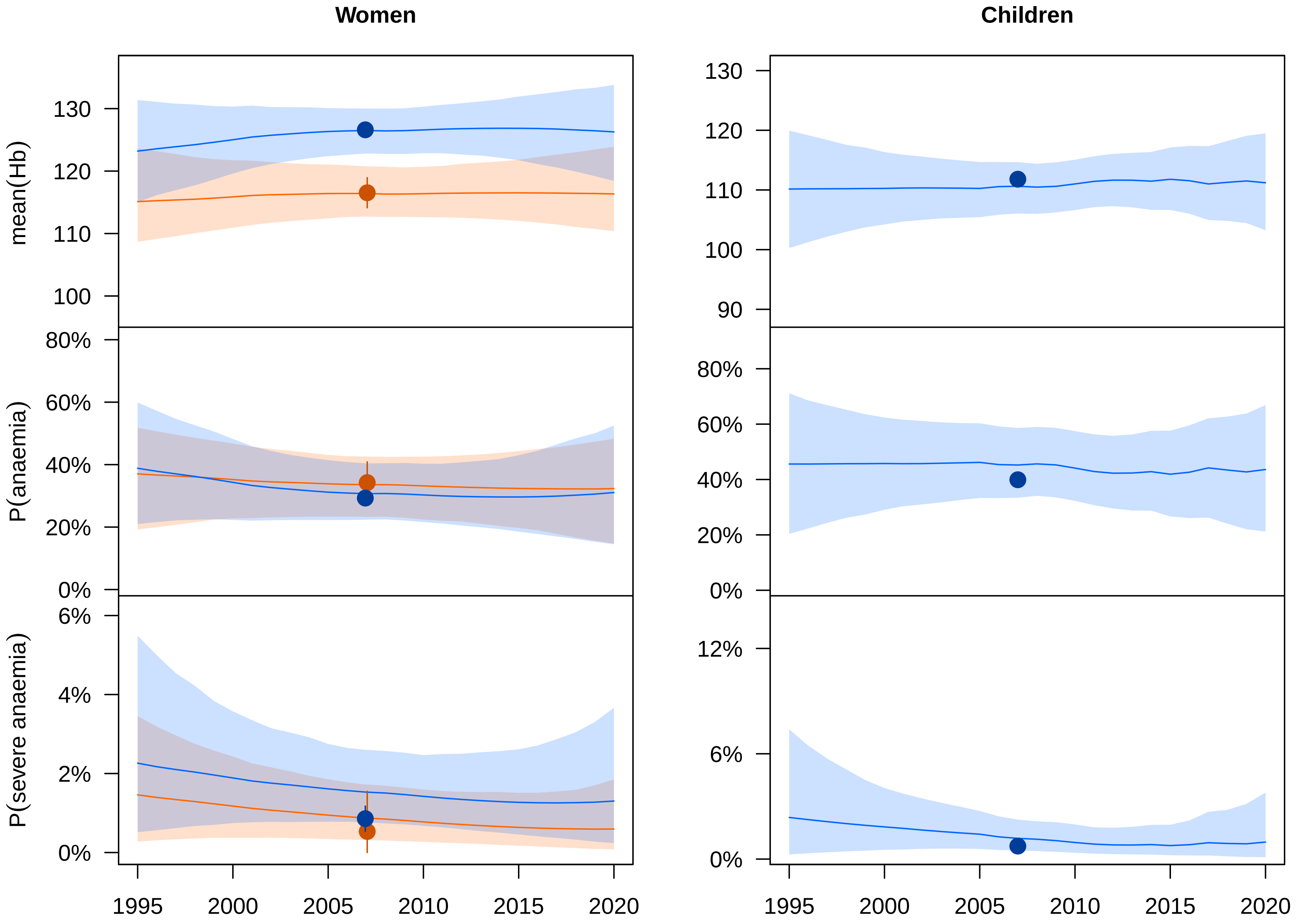

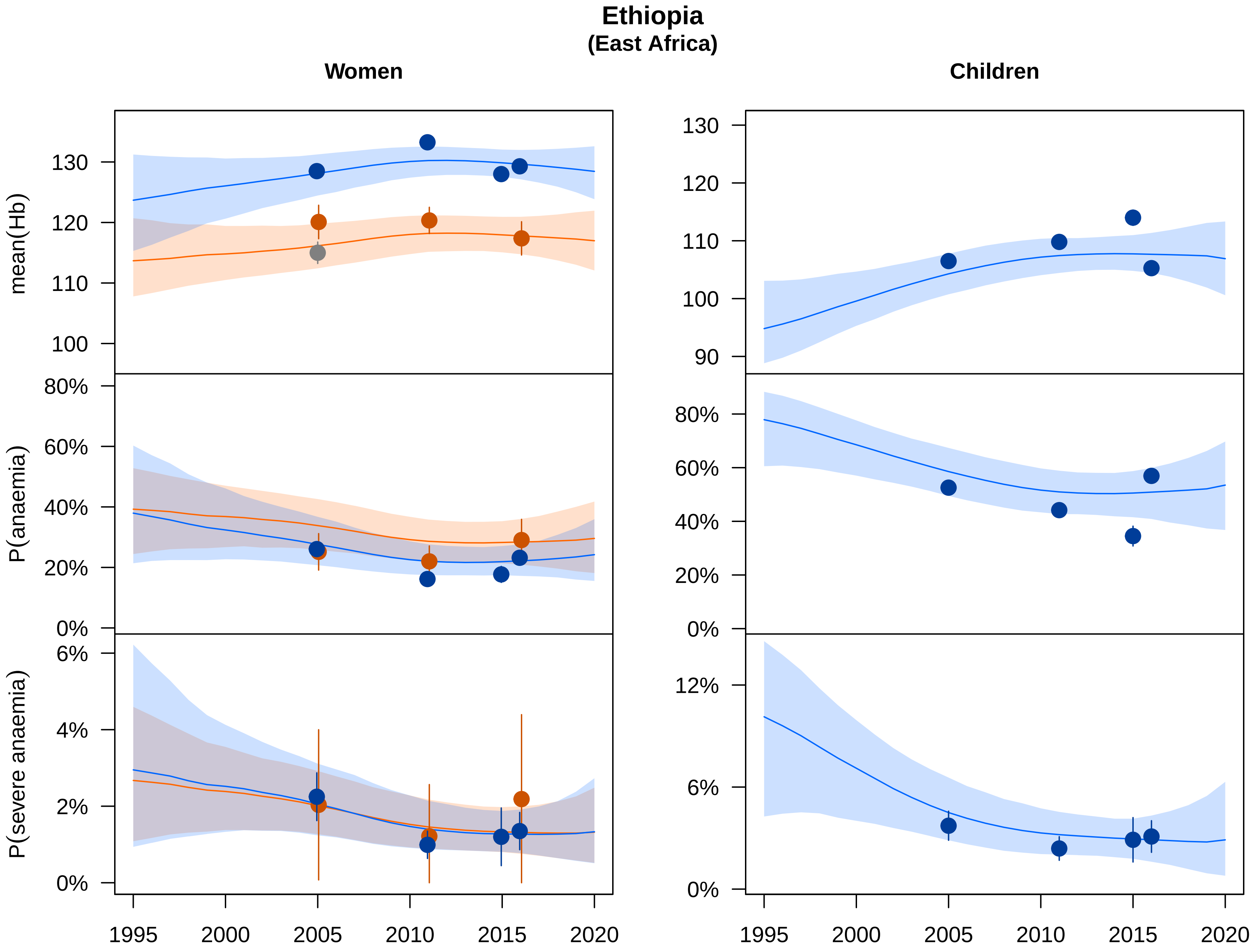

Ethiopia
(East Africa)
Women
Children
mean(Hb)
P(anaemia)
P(severe anaemia)
130
120
110
100
90
80%
60%
40%
20%
0%
6%
4%
2%
12%
1995
2000
2005
2010
2015
2020

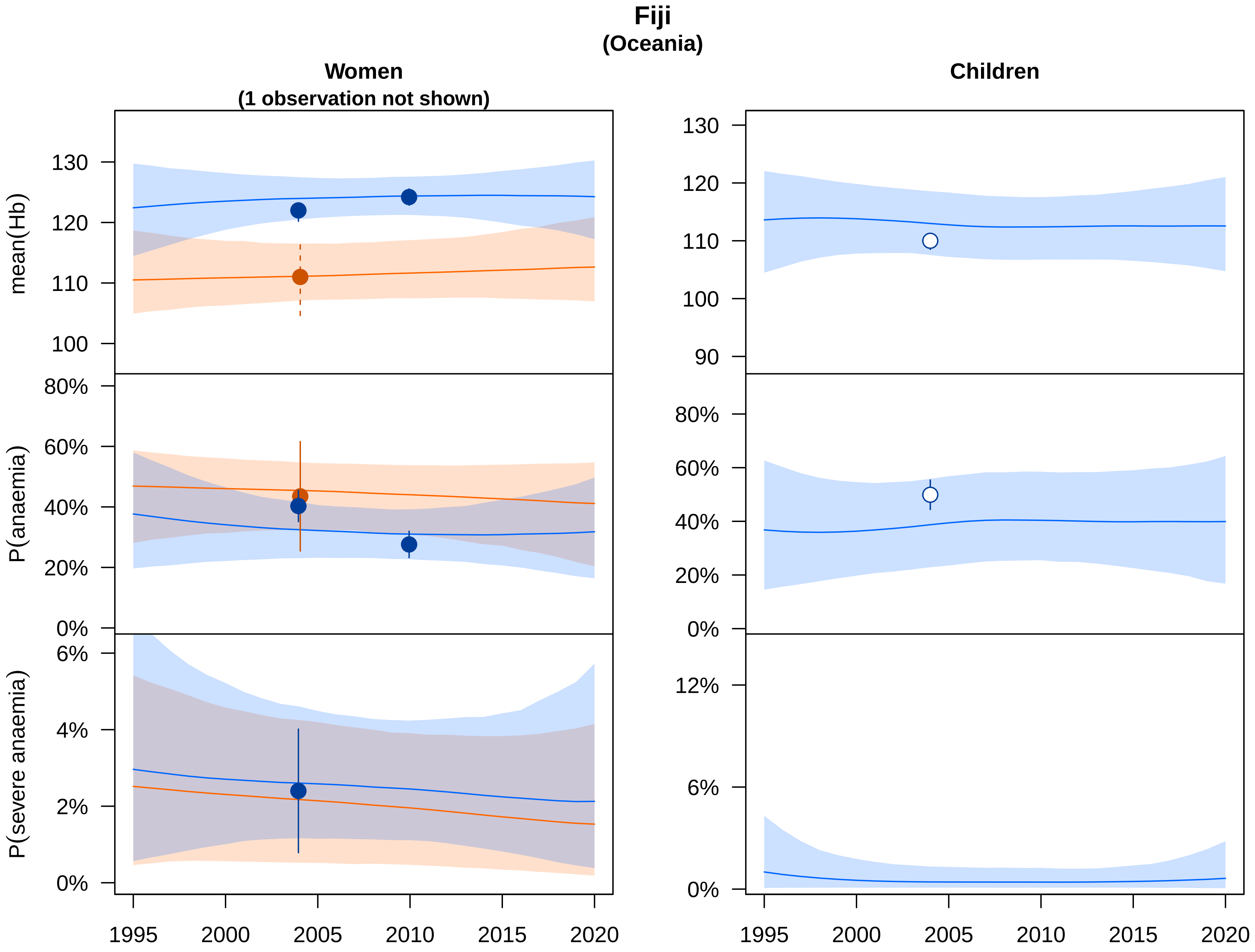

Fiji
(Oceania)
Women
(1 observation not shown)
Children
mean(Hb)
P(anaemia)
P(severe anaemia)
130
120
110
100
90
80%
60%
40%
20%
0%
6%
4%
2%
12%
1995
2000
2005
2010
2015
2020

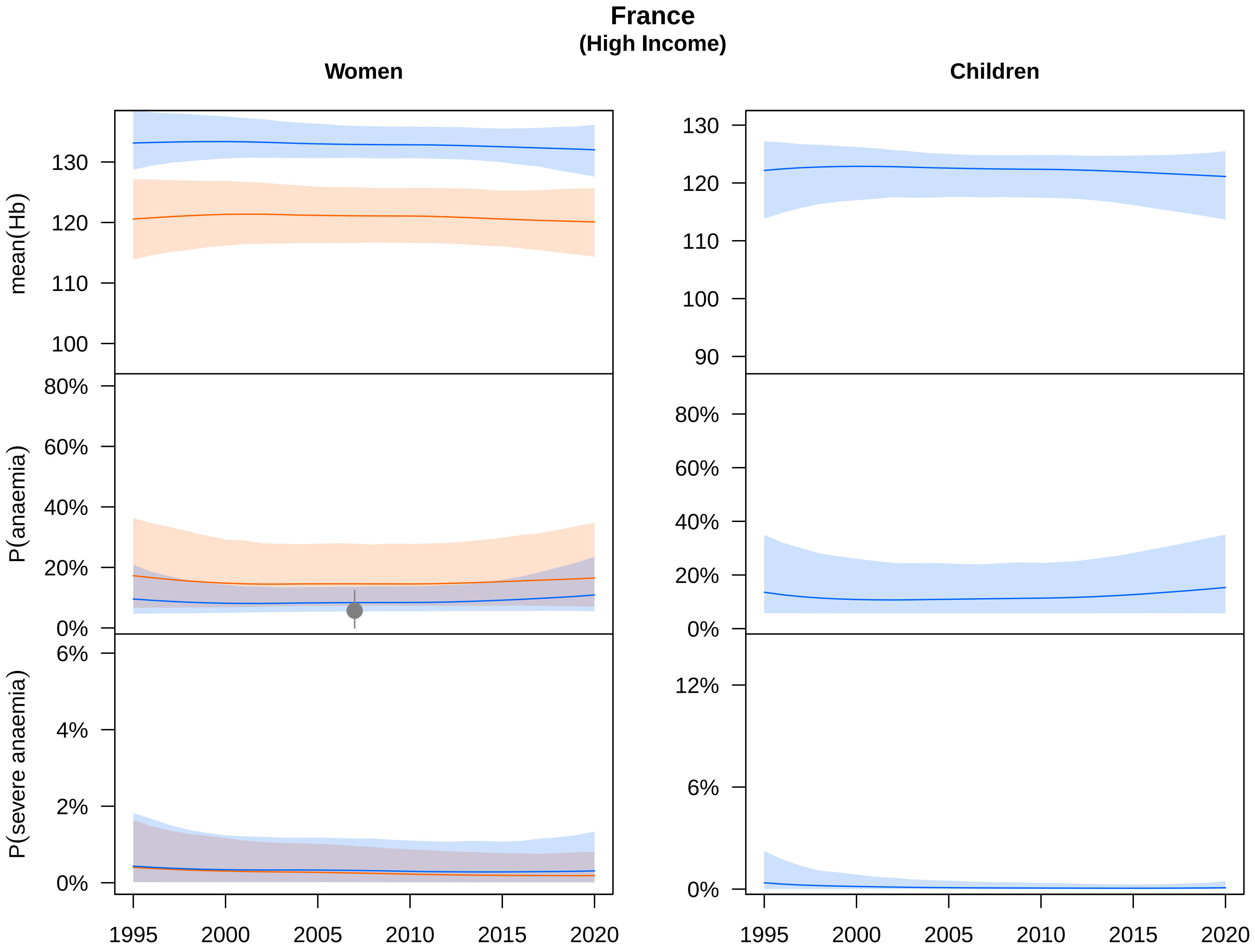

France
(High Income)
Women
Children
mean(Hb)
P(anaemia)
P(severe anaemia)
130
120
110
100
90
80%
60%
40%
20%
0%
6%
4%
2%
12%
1995
2000
2005
2010
2015
2020

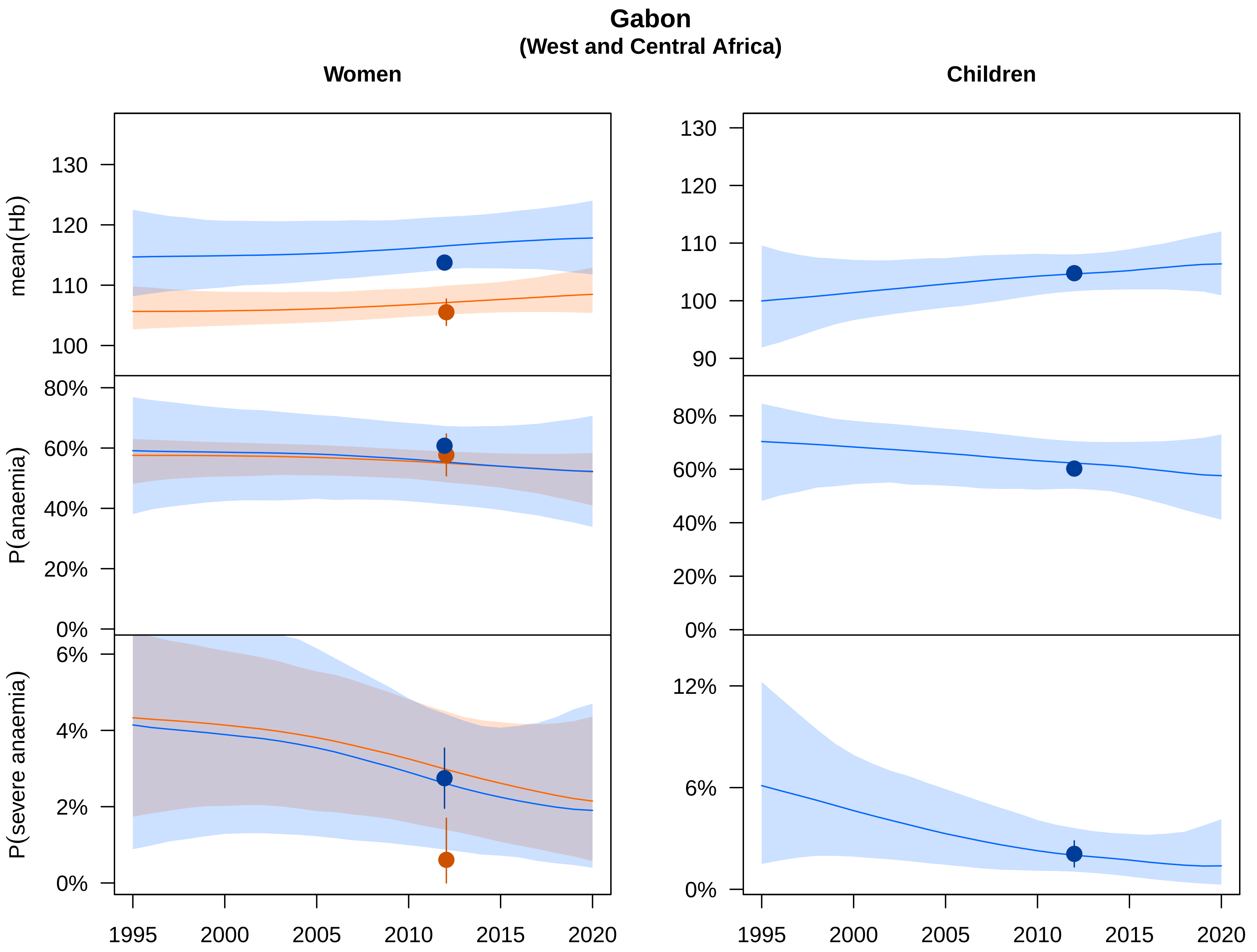
Gabon
(West and Central Africa)
Women
Children
mean(Hb)
P(anaemia)
P(severe anaemia)
130
120
110
100
90
80%
60%
40%
20%
0%
6%
4%
2%
12%
1995
2000
2005
2010
2015
2020

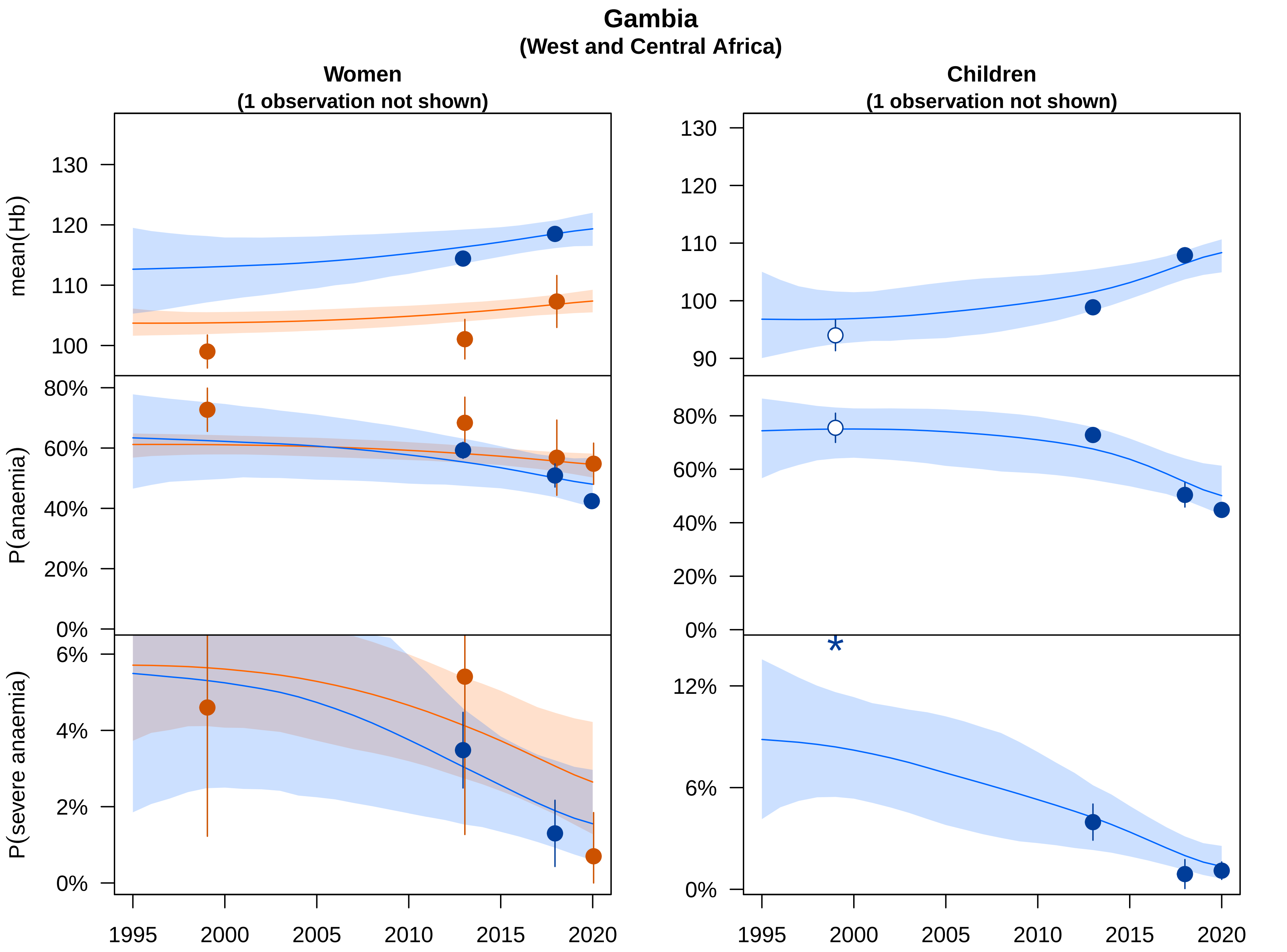

Gambia
(West and Central Africa)
Women
(1 observation not shown)
Children
(1 observation not shown)
mean(Hb)
P(anaemia)
P(severe anaemia)
130
120
110
100
90
80%
60%
40%
20%
0%
6%
4%
2%
12%
1995
2000
2005
2010
2015
2020

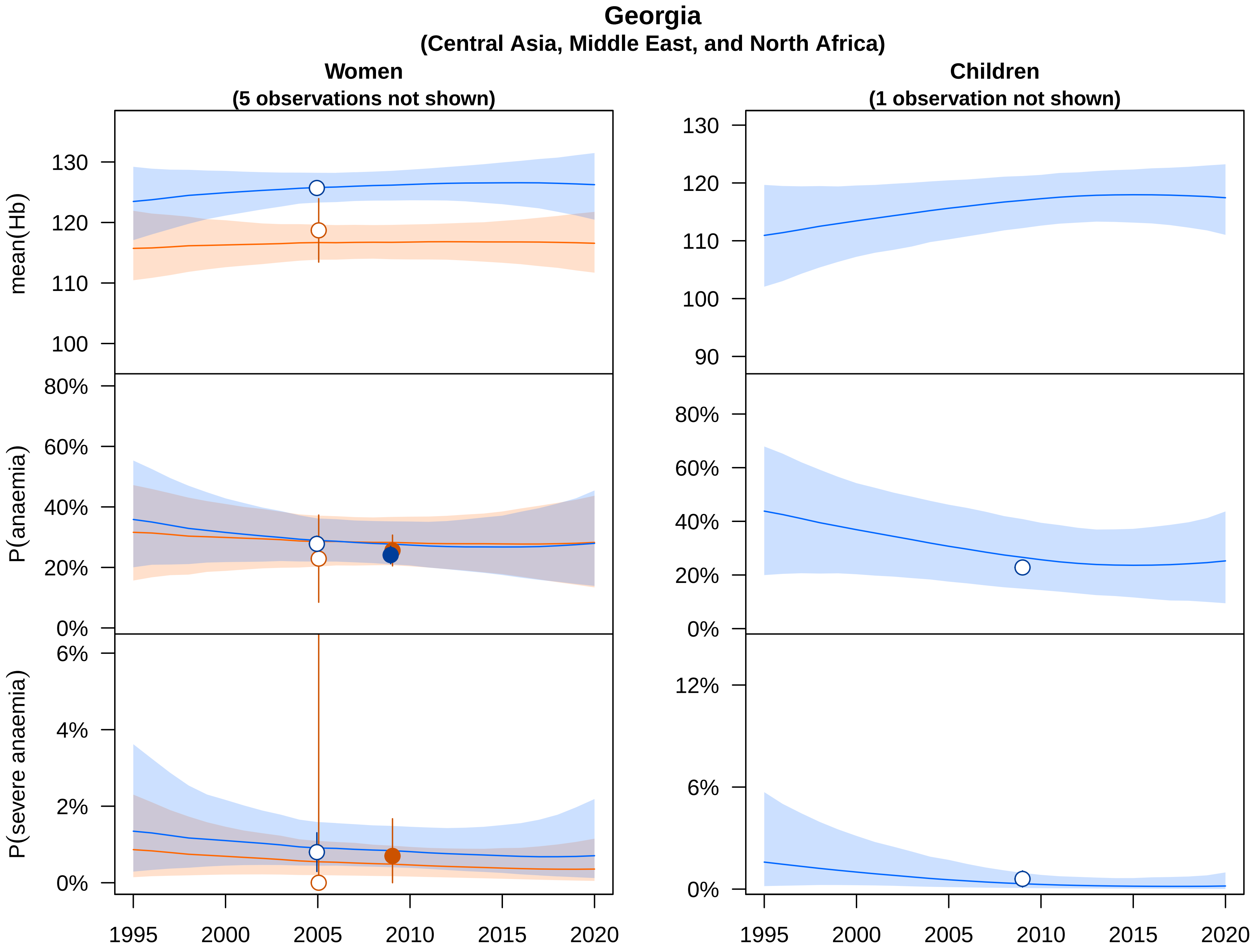

Georgia
(Central Asia, Middle East, and North Africa)
Women
(5 observations not shown)
Children
(1 observation not shown)
mean(Hb)
P(anaemia)
P(severe anaemia)
130
120
110
100
90
80%
60%
40%
20%
0%
6%
4%
2%
12%
1995
2000
2005
2010
2015
2020

Germany
(High Income)

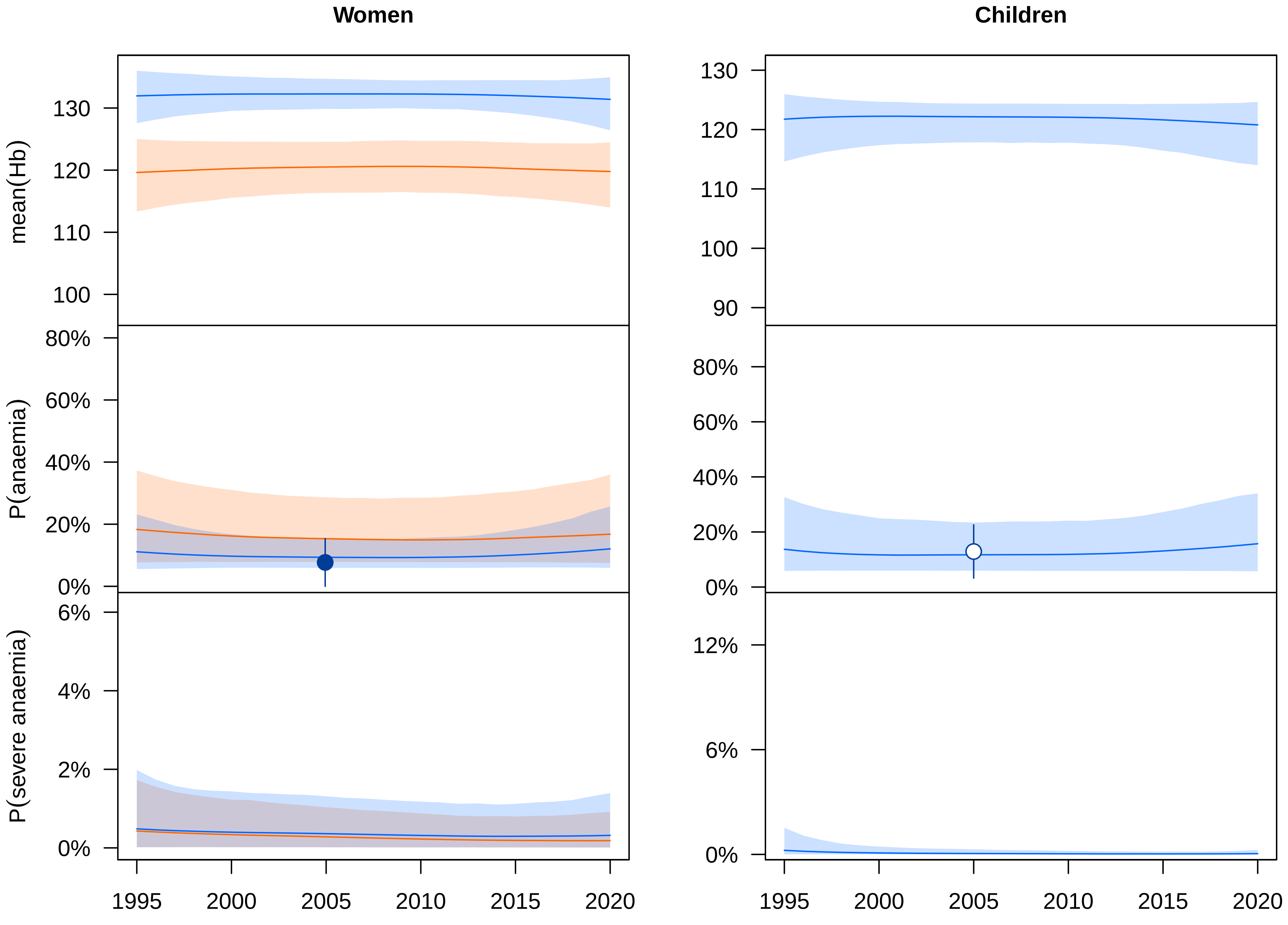

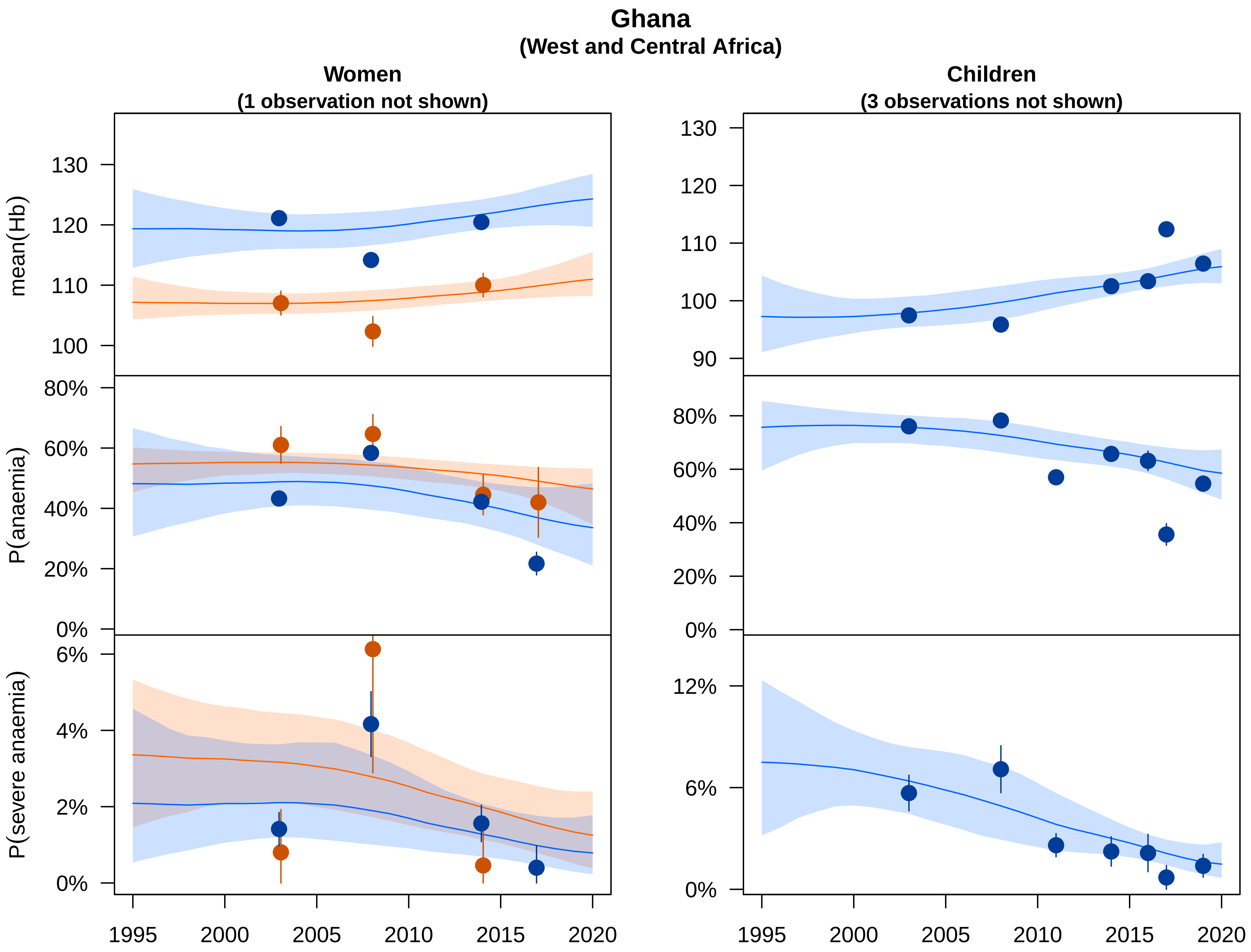
Ghana
(West and Central Africa)
Women
(1 observation not shown)
Children
(3 observations not shown)
mean(Hb)
P(anaemia)
P(severe anaemia)
130
120
110
100
90
80%
60%
40%
20%
0%
12%
6%
4%
2%
1995
2000
2005
2010
2015
2020

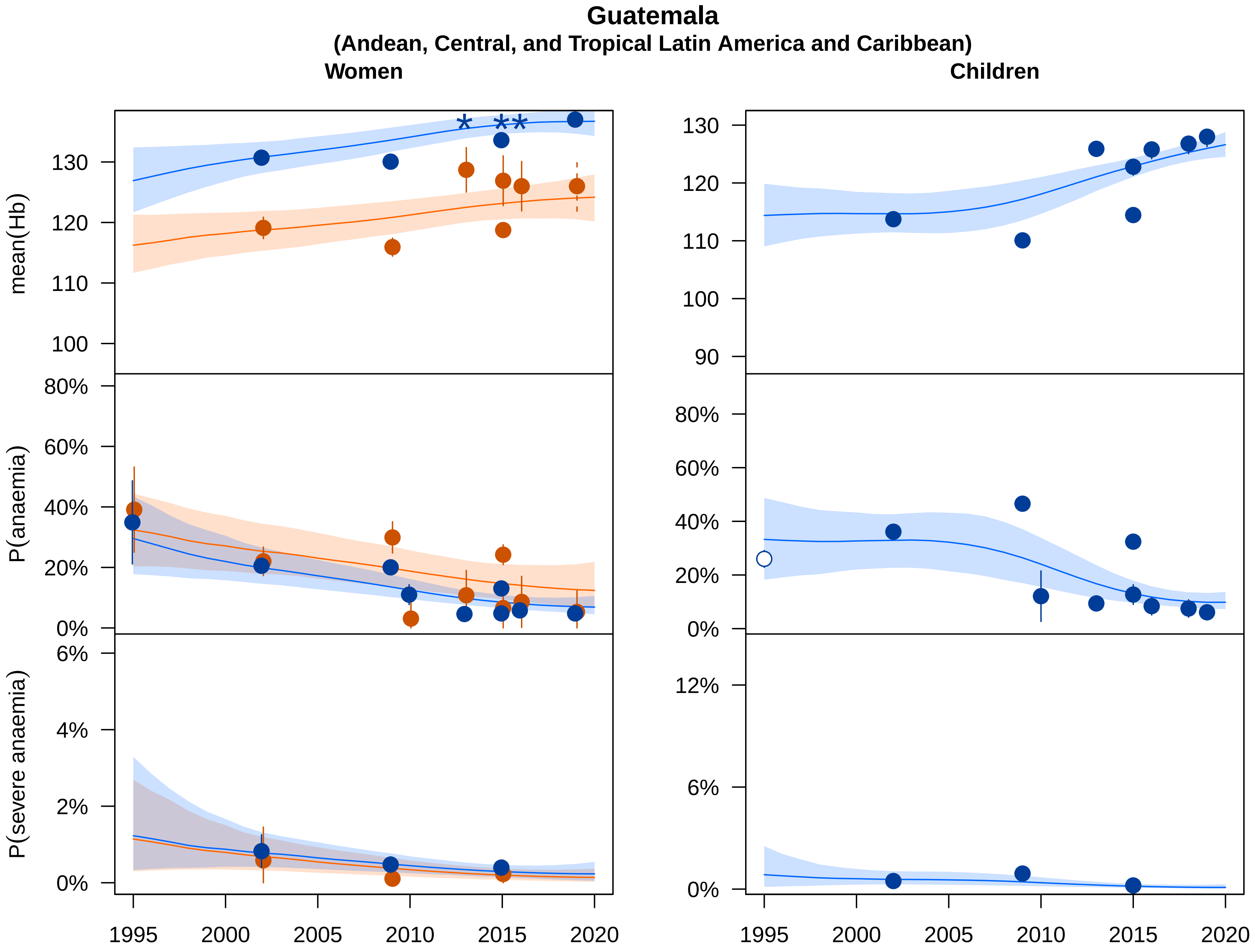
Guatemala
(Andean, Central, and Tropical Latin America and Caribbean)
Women
Children
mean(Hb)
P(anaemia)
P(severe anaemia)
130
120
110
100
90
80%
60%
40%
20%
0%
6%
4%
2%
12%
1995
2000
2005
2010
2015
2020

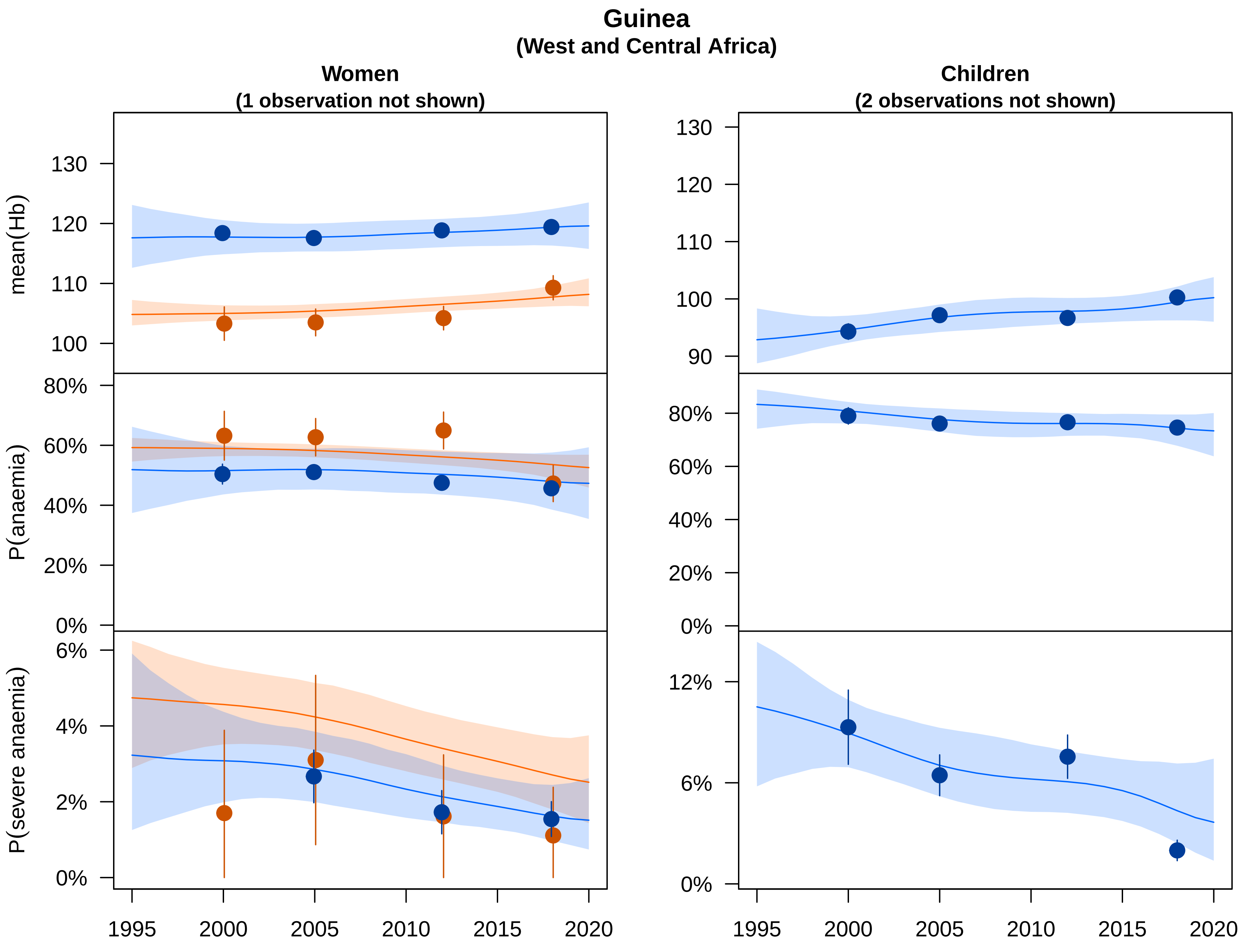

Guinea
(West and Central Africa)
Women
(1 observation not shown)
Children
(2 observations not shown)
mean(Hb)
P(anaemia)
P(severe anaemia)
130
120
110
100
90
80%
60%
40%
20%
0%
6%
4%
2%
12%
1995
2000
2005
2010
2015
2020

Guyana

(Andean, Central, and Tropical Latin America and Caribbean)

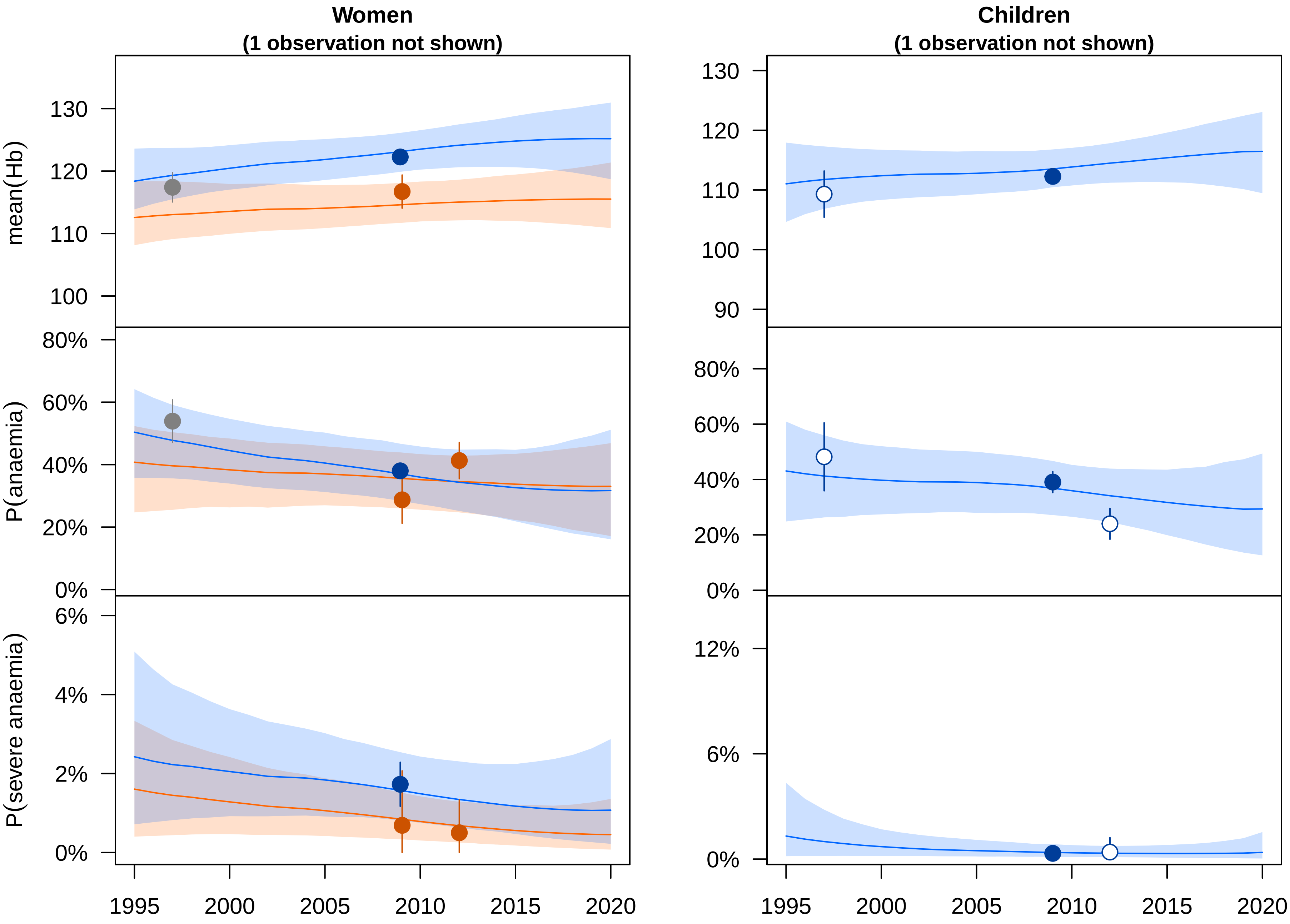

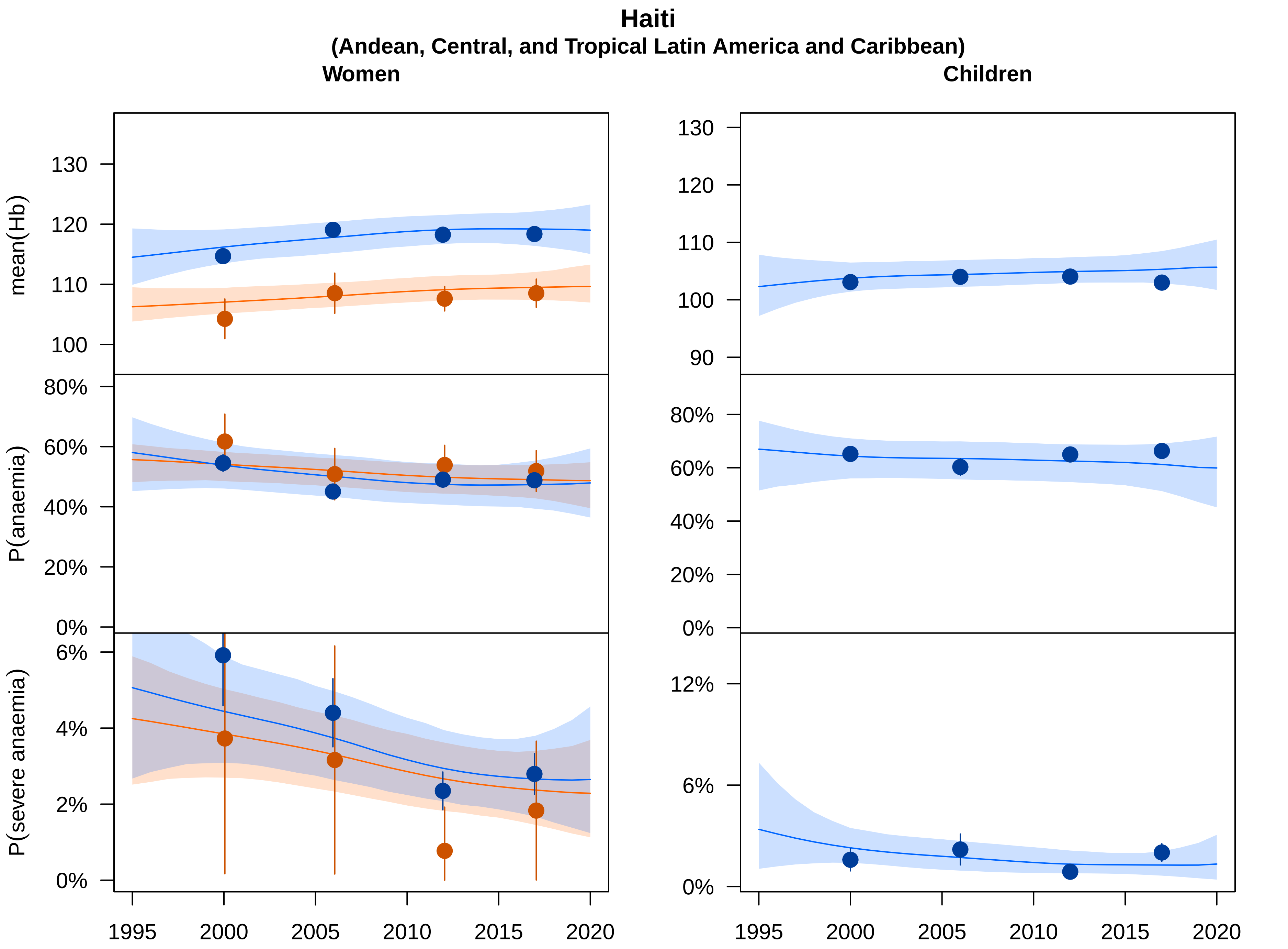

Haiti
(Andean, Central, and Tropical Latin America and Caribbean)
Women
Children
mean(Hb)
P(anaemia)
P(severe anaemia)
130
120
110
100
90
80%
60%
40%
20%
0%
6%
4%
2%
12%
1995
2000
2005
2010
2015
2020

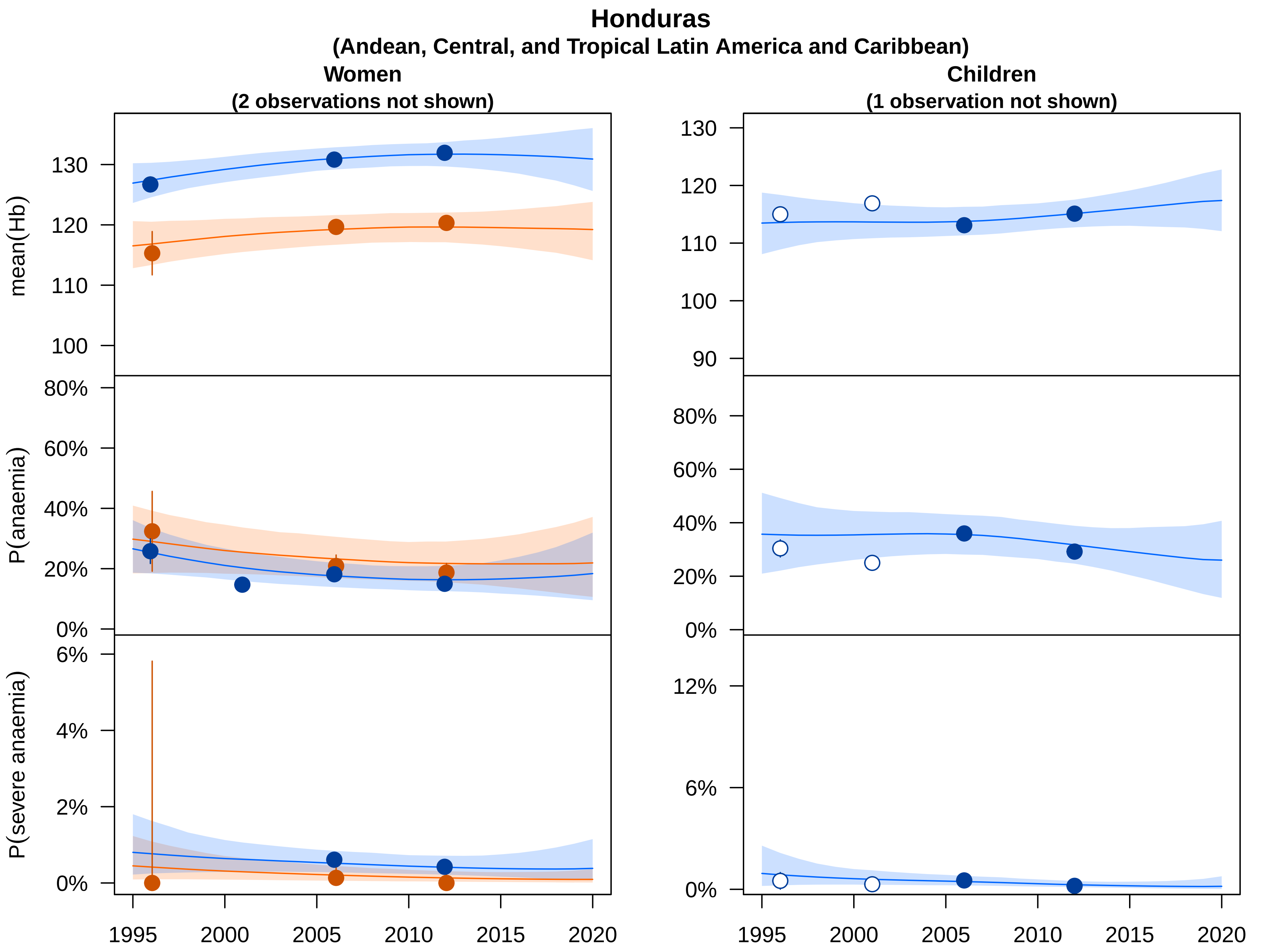
Honduras
(Andean, Central, and Tropical Latin America and Caribbean)
Women
(2 observations not shown)
Children
(1 observation not shown)
mean(Hb)
P(anaemia)
P(severe anaemia)
130
120
110
100
90
80%
60%
40%
20%
0%
6%
4%
2%
12%
1995
2000
2005
2010
2015
2020

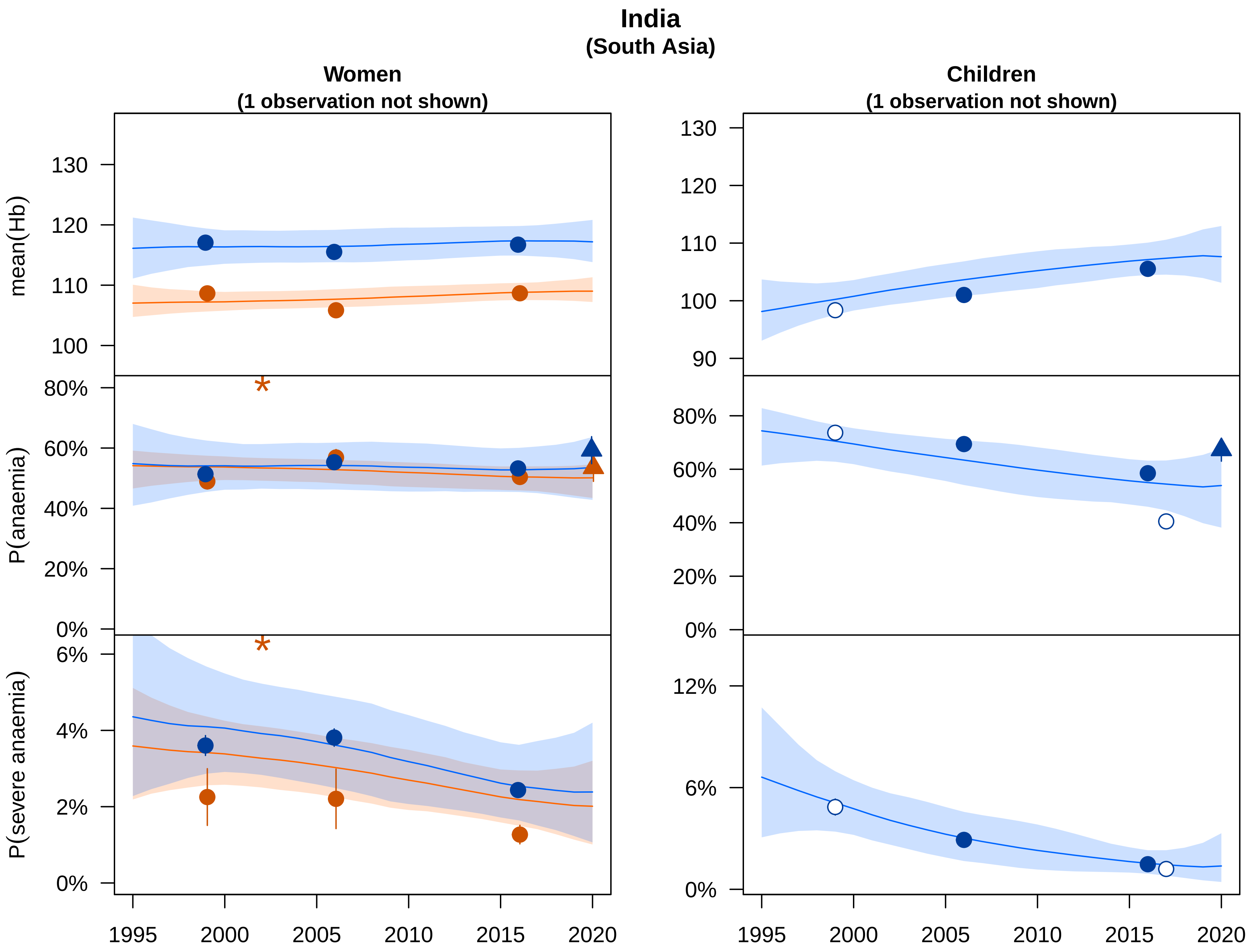

India
(South Asia)
Women
(1 observation not shown)
Children
(1 observation not shown)
mean(Hb)
P(anaemia)
P(severe anaemia)
130
120
110
100
90
80%
60%
40%
20%
0%
6%
4%
2%
12%
1995
2000
2005
2010
2015
2020

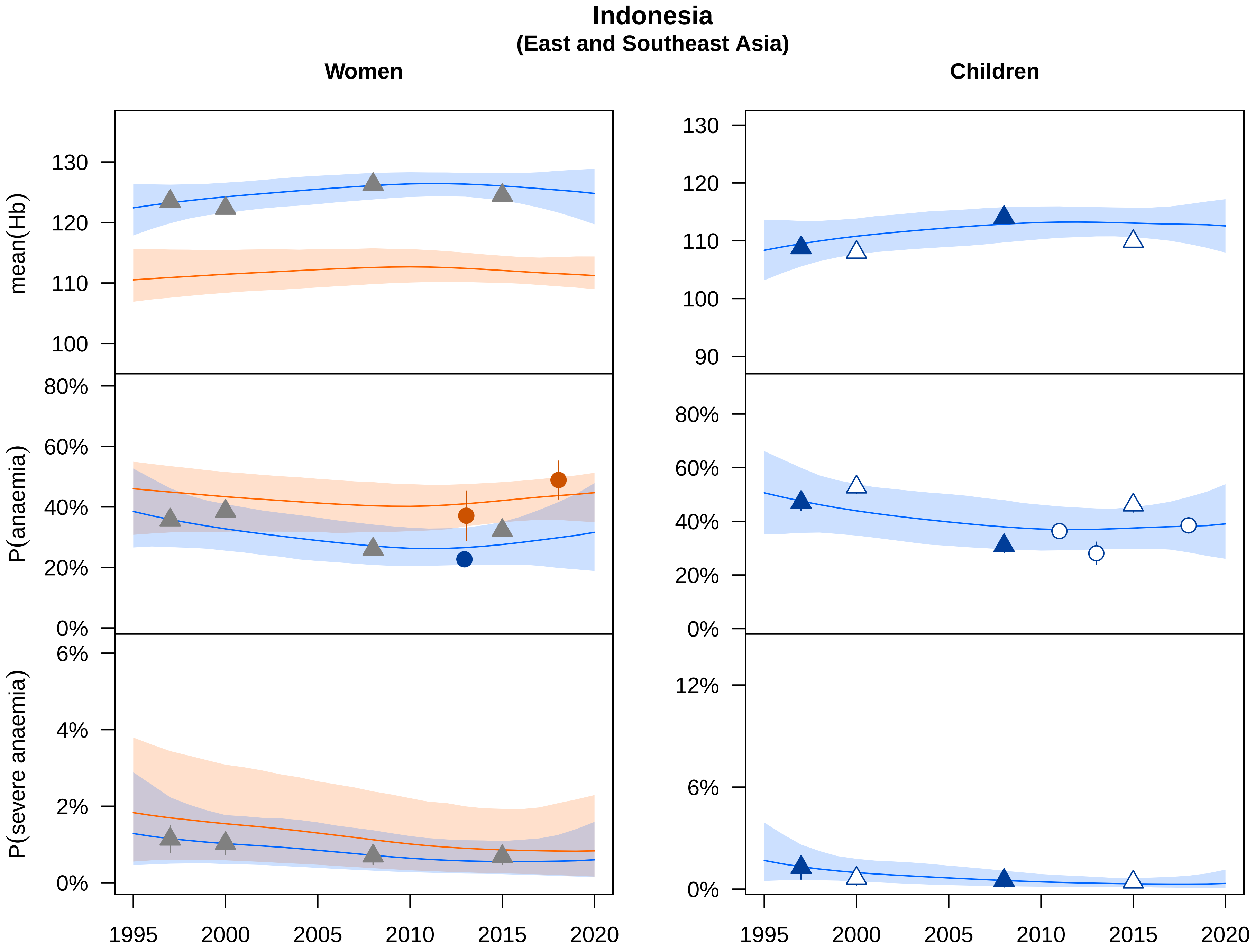

Indonesia
(East and Southeast Asia)
Women
Children
mean(Hb)
P(anaemia)
P(severe anaemia)
130
120
110
100
90
80%
60%
40%
20%
0%
6%
4%
2%
12%
1995
2000
2005
2010
2015
2020

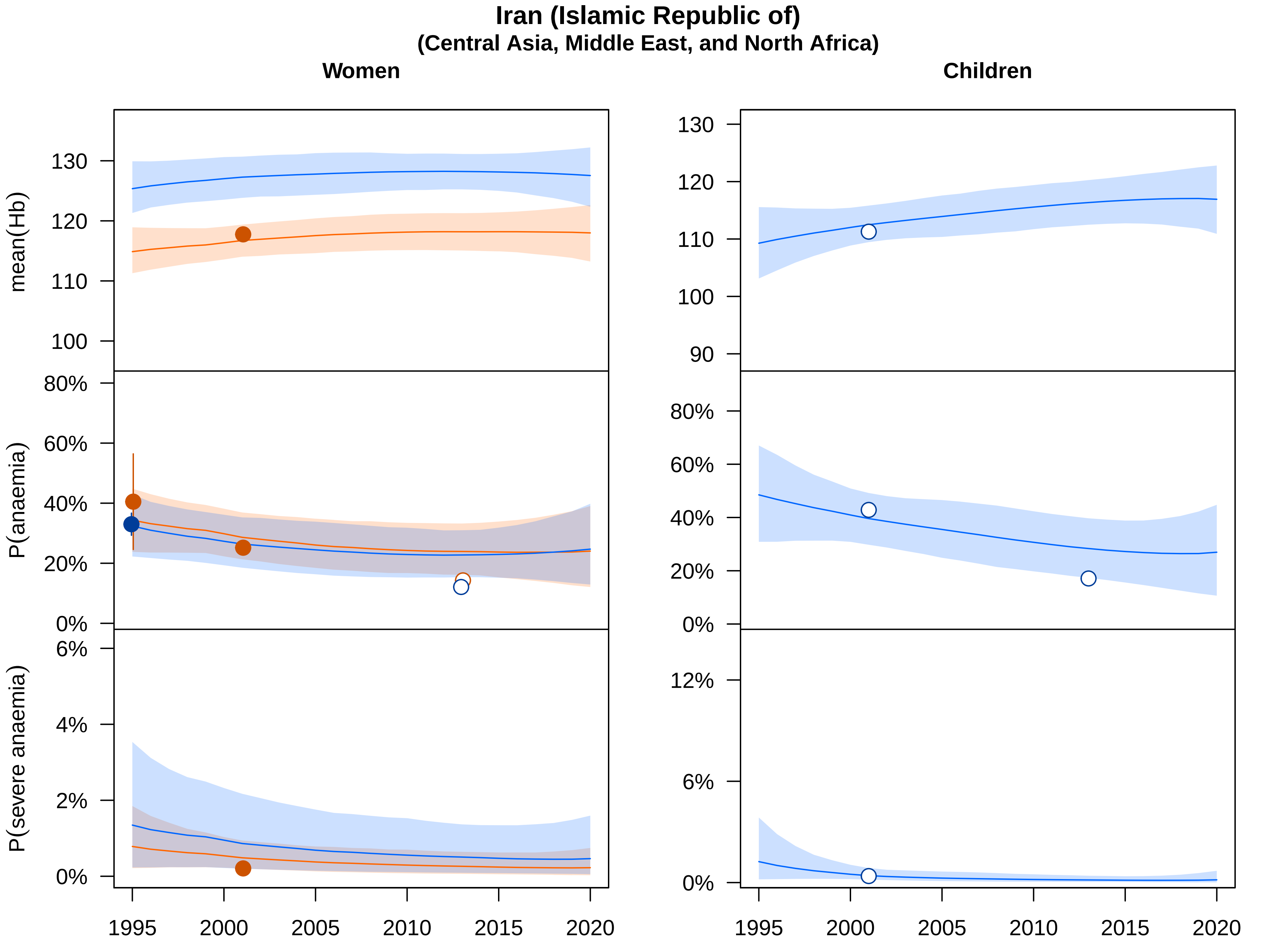
Iran (Islamic Republic of)
(Central Asia, Middle East, and North Africa)
Women
Children
mean(Hb)
P(anaemia)
P(severe anaemia)
130
120
110
100
90
80%
60%
40%
20%
0%
6%
4%
2%
12%
1995
2000
2005
2010
2015
2020

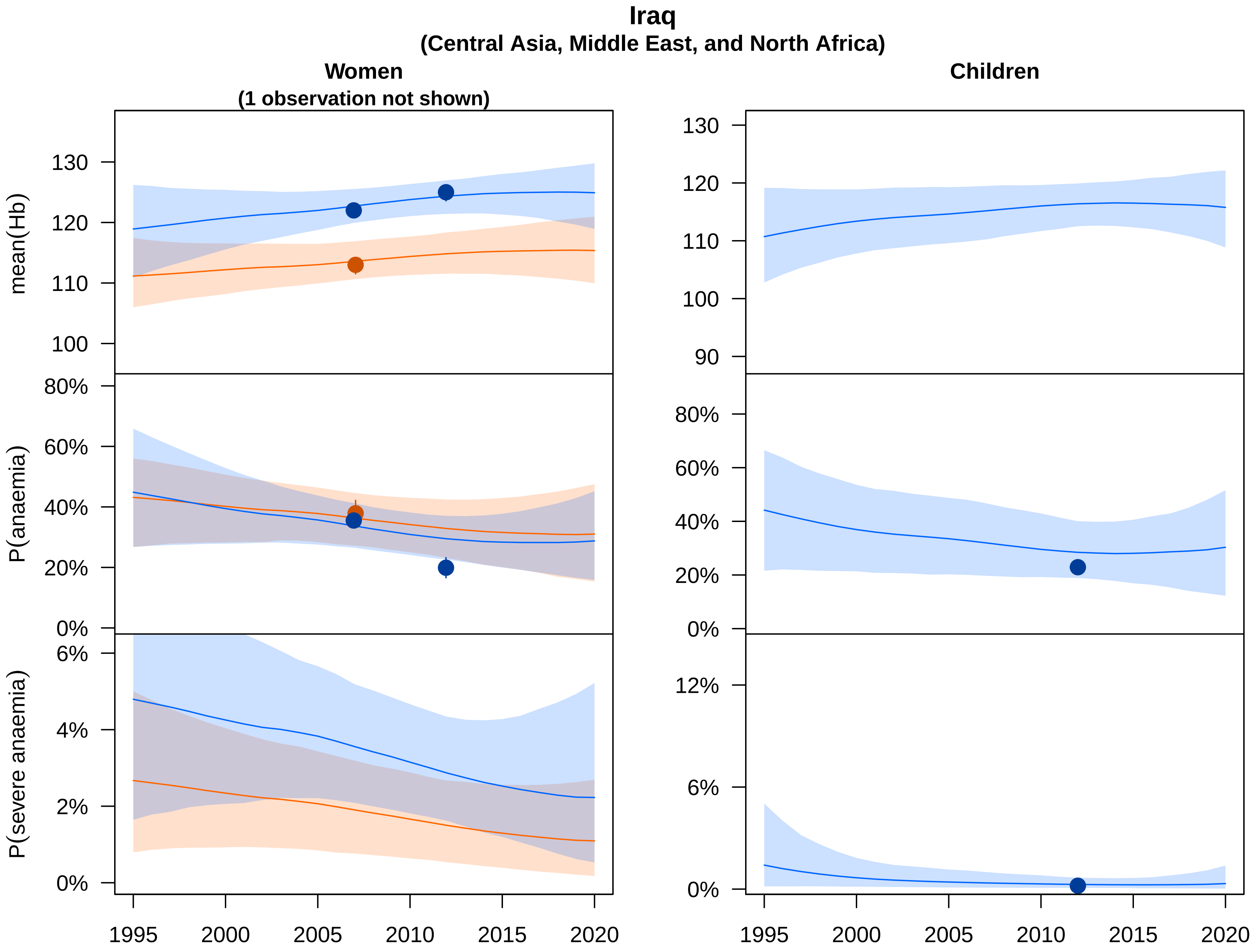

Iraq
(Central Asia, Middle East, and North Africa)
Women
(1 observation not shown)
Children
mean(Hb)
P(anaemia)
P(severe anaemia)
130
120
110
100
90
80%
60%
40%
20%
0%
6%
4%
2%
12%
1995
2000
2005
2010
2015
2020

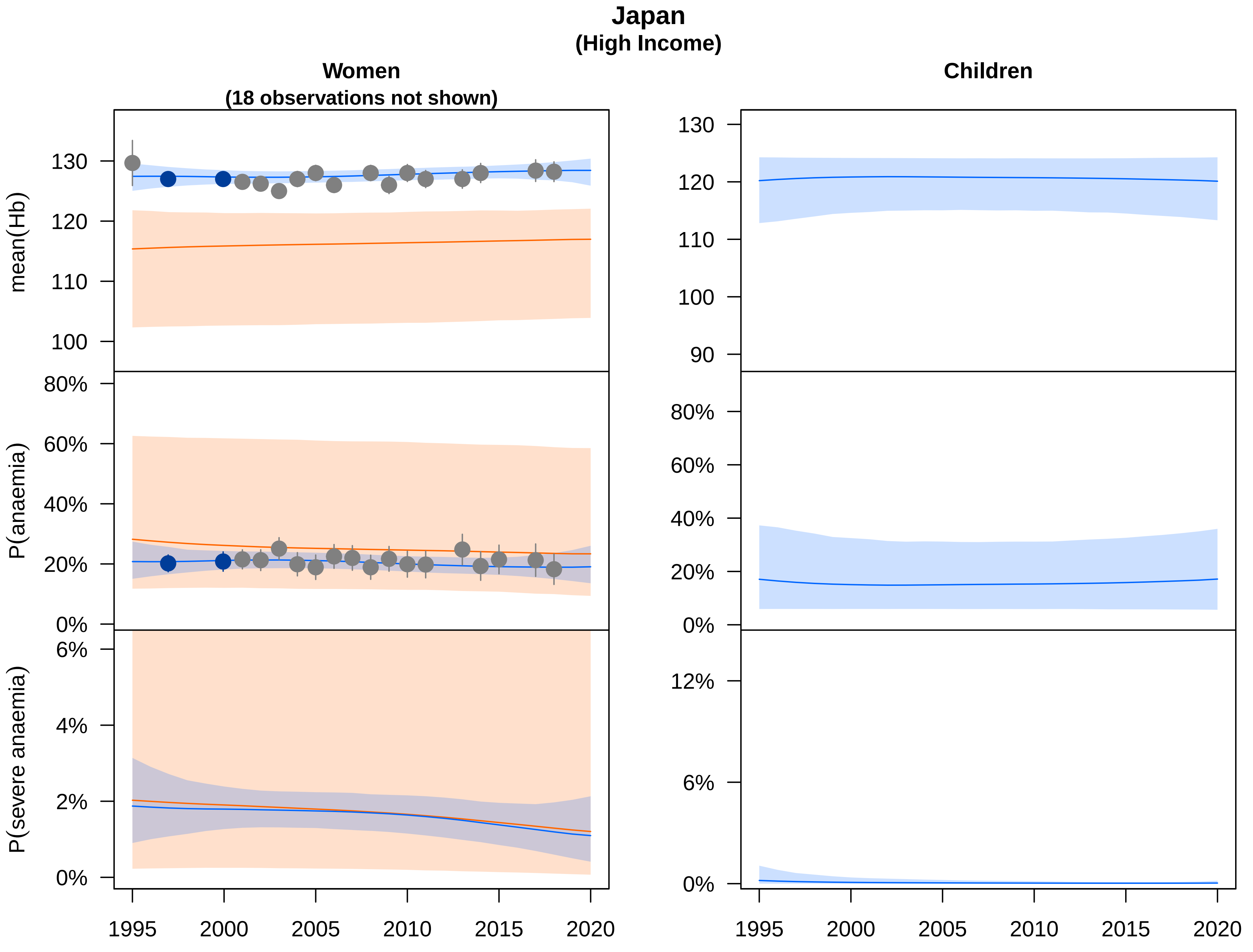

Japan
(High Income)
Women
(18 observations not shown)
Children
mean(Hb)
P(anaemia)
P(severe anaemia)
130
120
110
100
90
80%
60%
40%
20%
0%
6%
4%
2%
12%
1995
2000
2005
2010
2015
2020

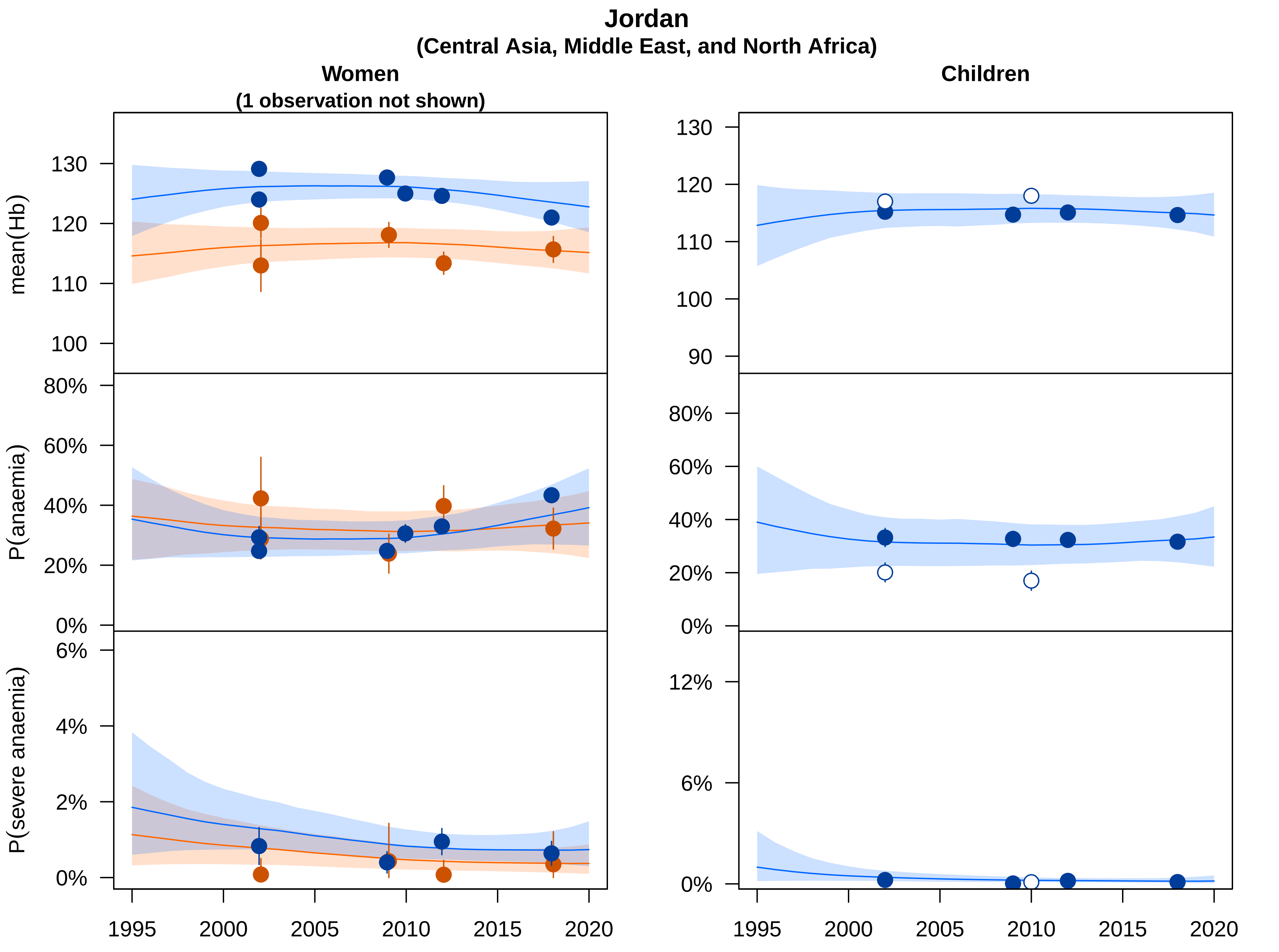
Jordan
(Central Asia, Middle East, and North Africa)
Women
(1 observation not shown)
Children
mean(Hb)
P(anaemia)
P(severe anaemia)
130
120
110
100
90
80%
60%
40%
20%
0%
6%
4%
2%
12%
1995
2000
2005
2010
2015
2020

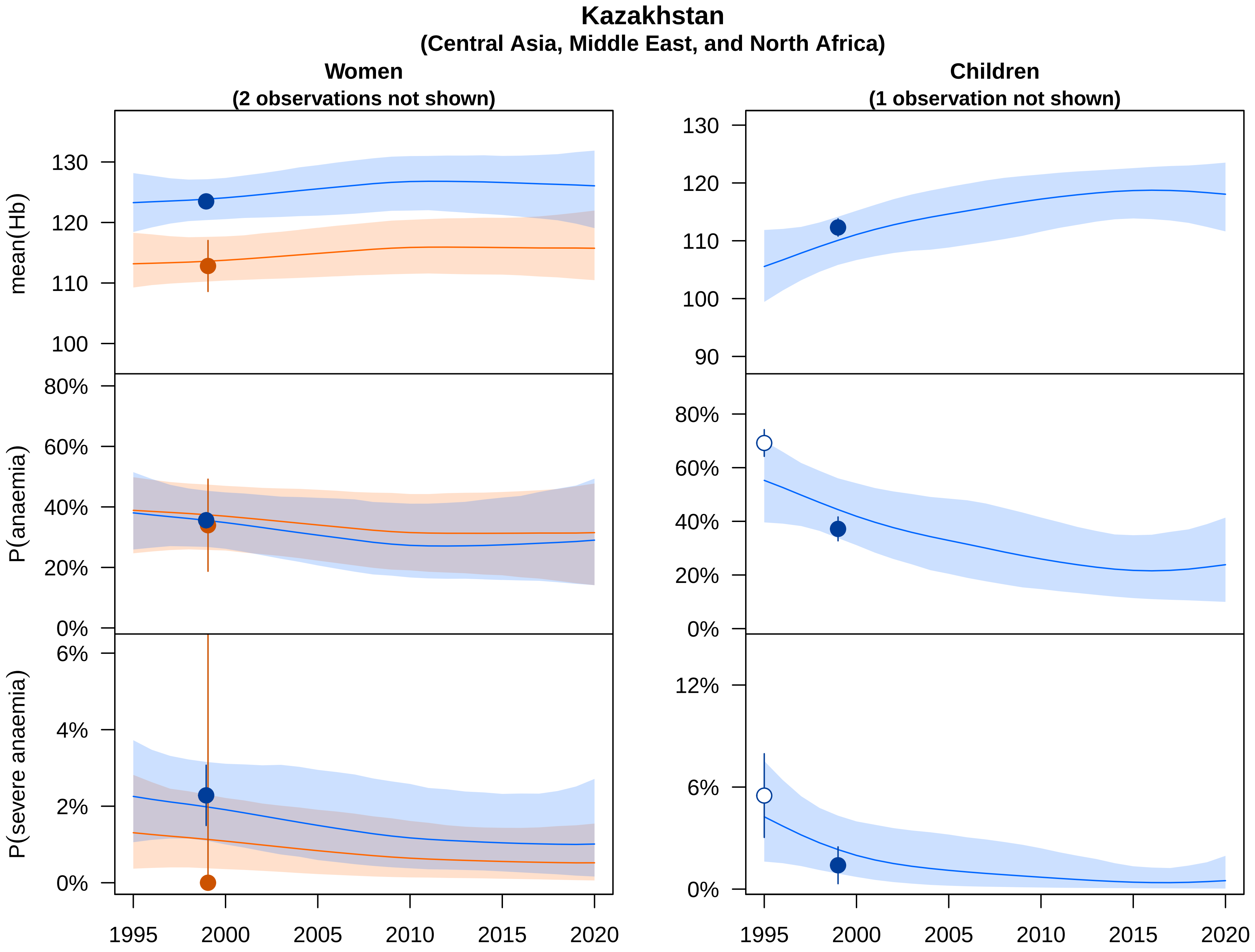
Kazakhstan
(Central Asia, Middle East, and North Africa)
Women
(2 observations not shown)
Children
(1 observation not shown)
mean(Hb)
P(anaemia)
P(severe anaemia)
130
120
110
100
90
80%
60%
40%
20%
0%
6%
4%
2%
12%
1995
2000
2005
2010
2015
2020

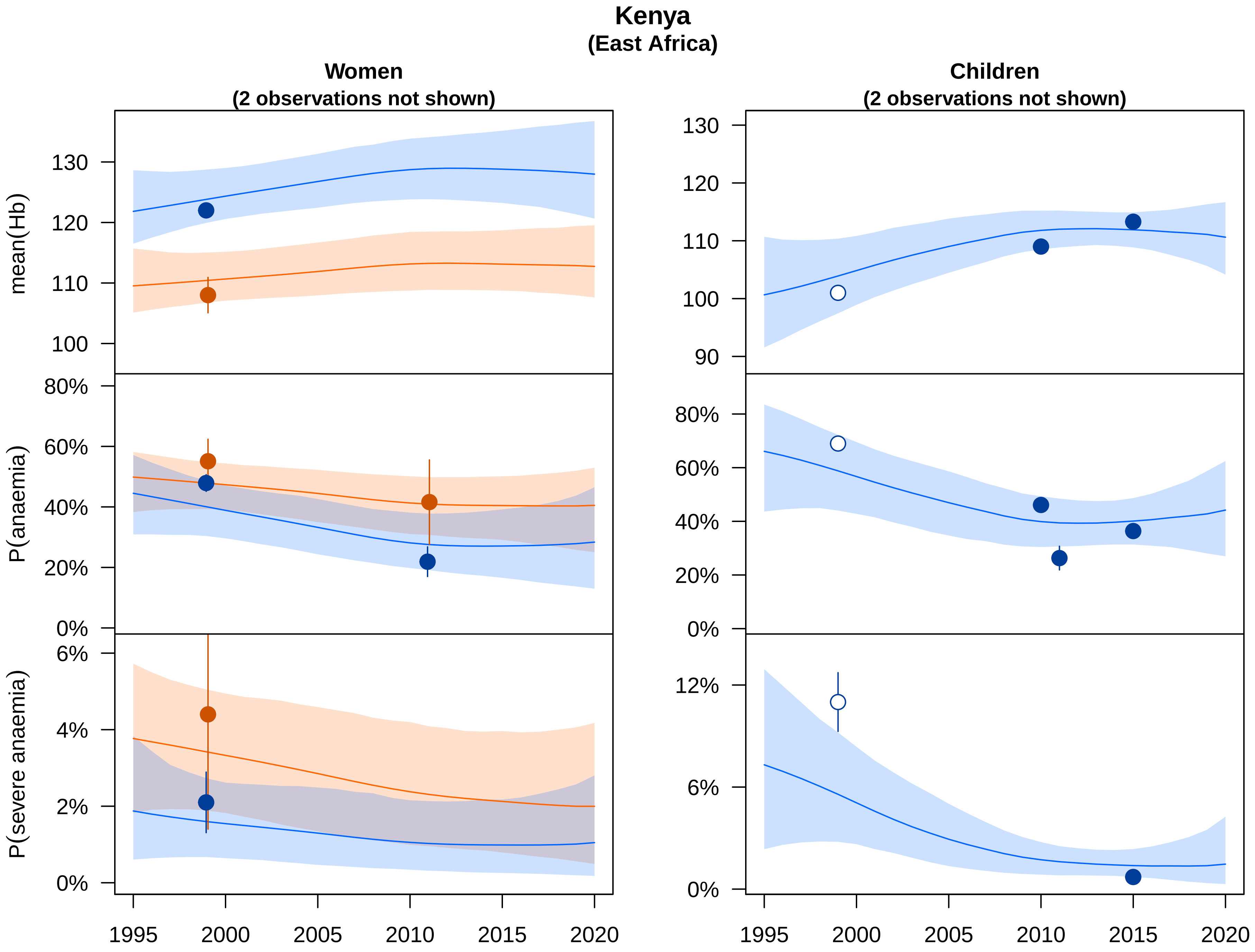
Kenya
(East Africa)
Women
(2 observations not shown)
Children
(2 observations not shown)
mean(Hb)
P(anaemia)
P(severe anaemia)
130
120
110
100
90
80%
60%
40%
20%
0%
6%
4%
2%
12%
1995
2000
2005
2010
2015
2020

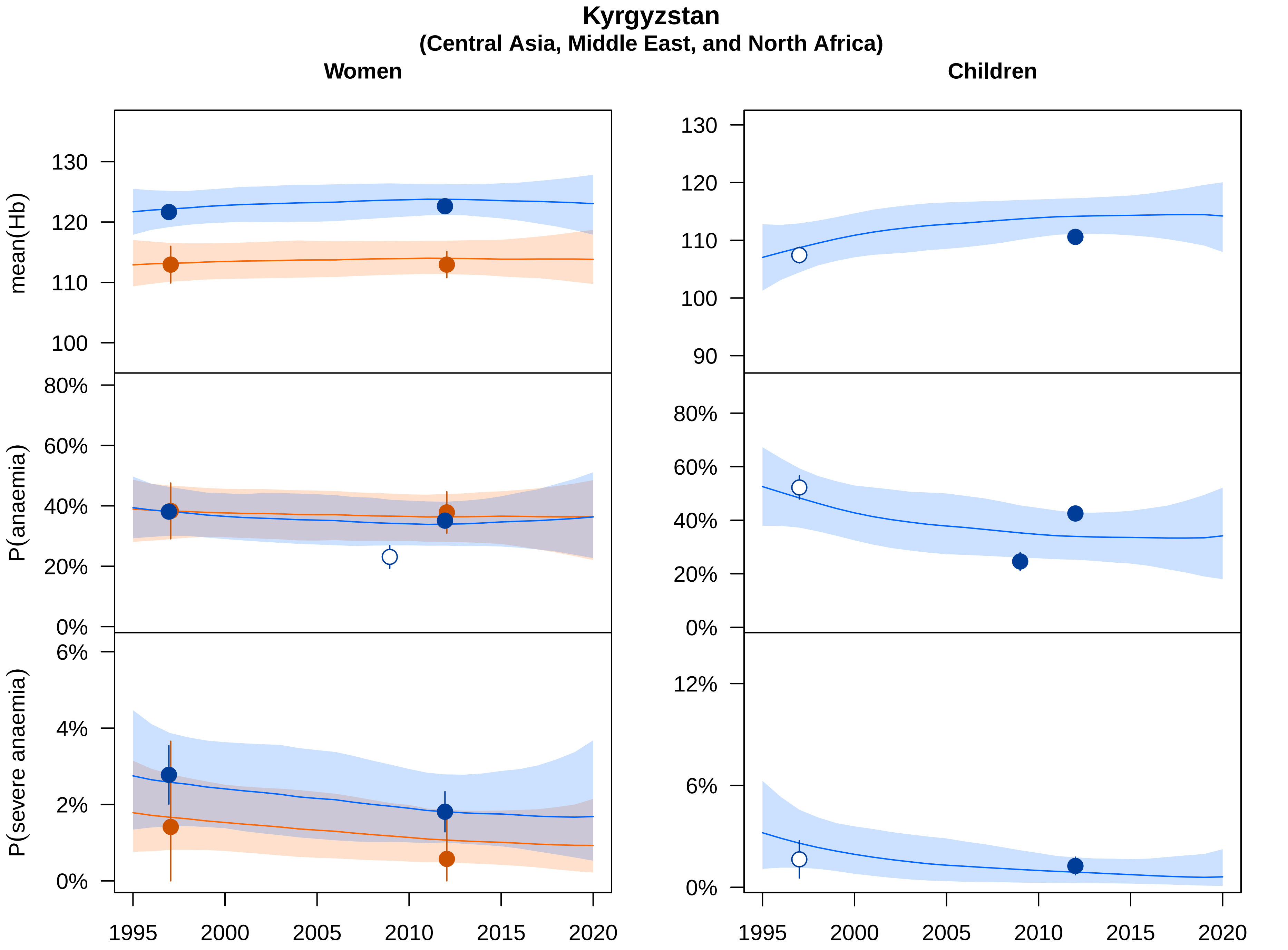
Kyrgyzstan
(Central Asia, Middle East, and North Africa)
Women
Children
mean(Hb)
P(anaemia)
P(severe anaemia)
130
120
110
100
90
80%
60%
40%
20%
0%
6%
4%
2%
12%
1995
2000
2005
2010
2015
2020

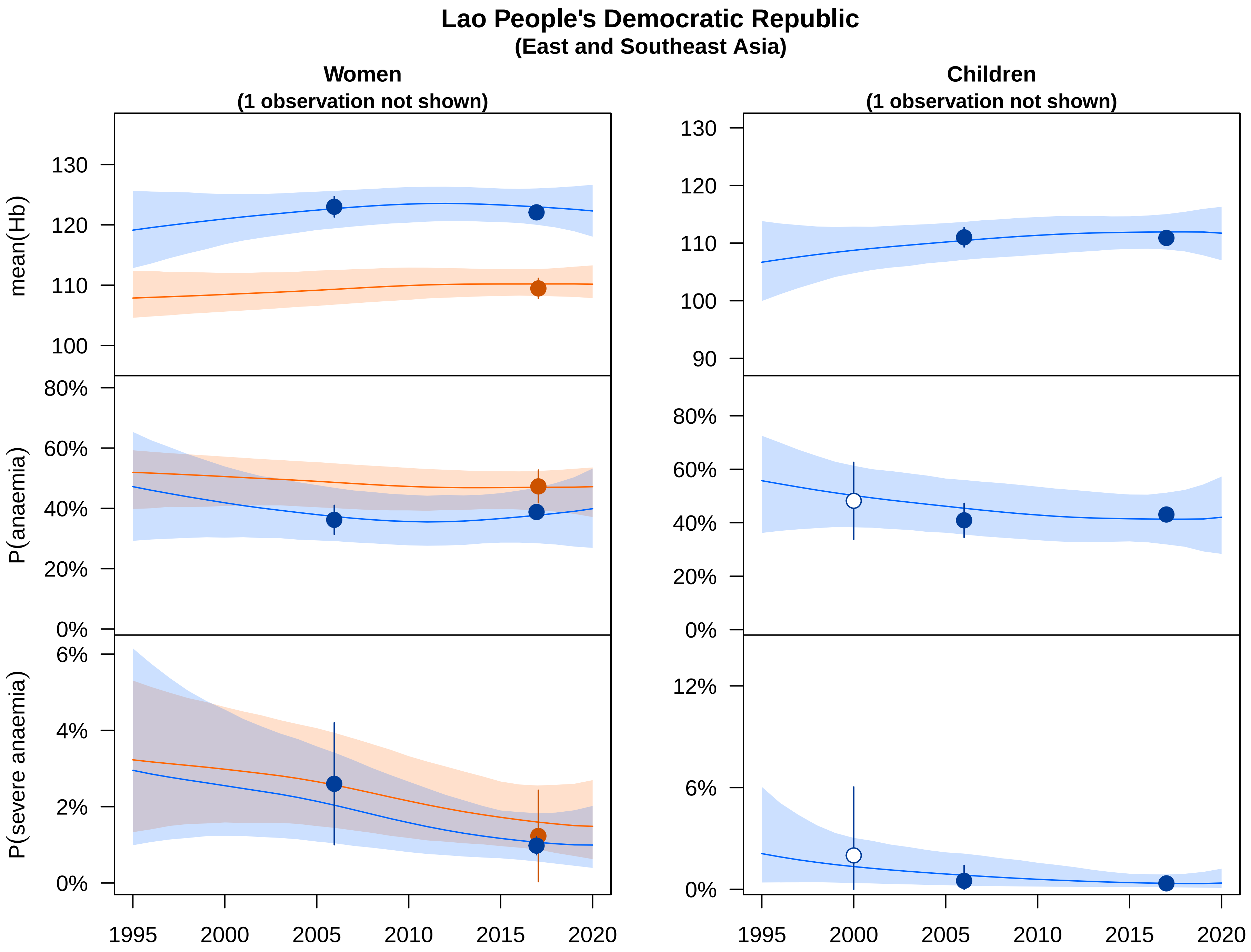
Lao People's Democratic Republic
(East and Southeast Asia)
Women
(1 observation not shown)
Children
(1 observation not shown)
mean(Hb)
P(anaemia)
P(severe anaemia)
130
120
110
100
90
80%
60%
40%
20%
0%
6%
4%
2%
12%
1995
2000
2005
2010
2015
2020

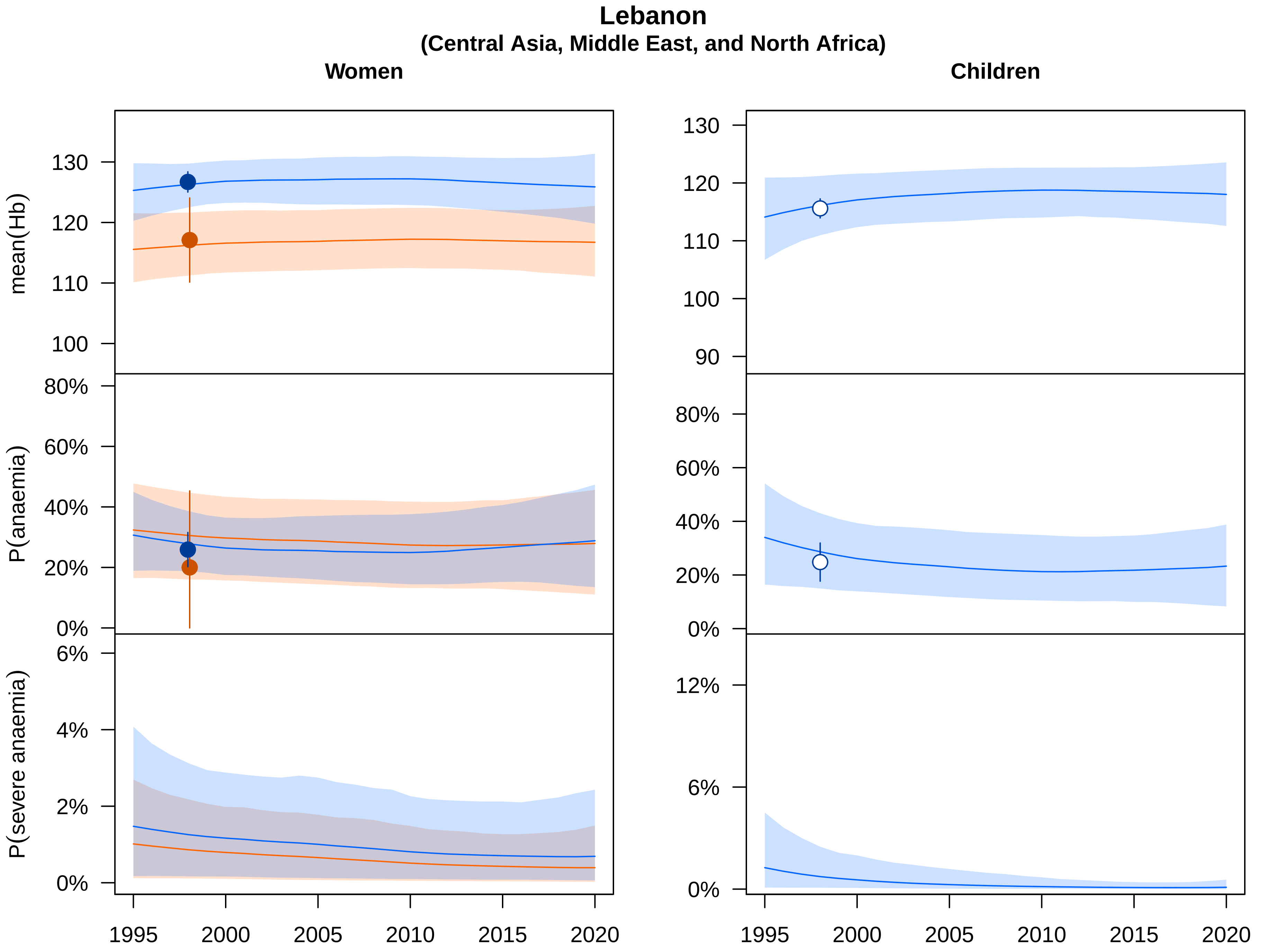

Lebanon
(Central Asia, Middle East, and North Africa)
Women
Children
mean(Hb)
P(anaemia)
P(severe anaemia)
130
120
110
100
90
80%
60%
40%
20%
0%
6%
4%
2%
12%
1995
2000
2005
2010
2015
2020

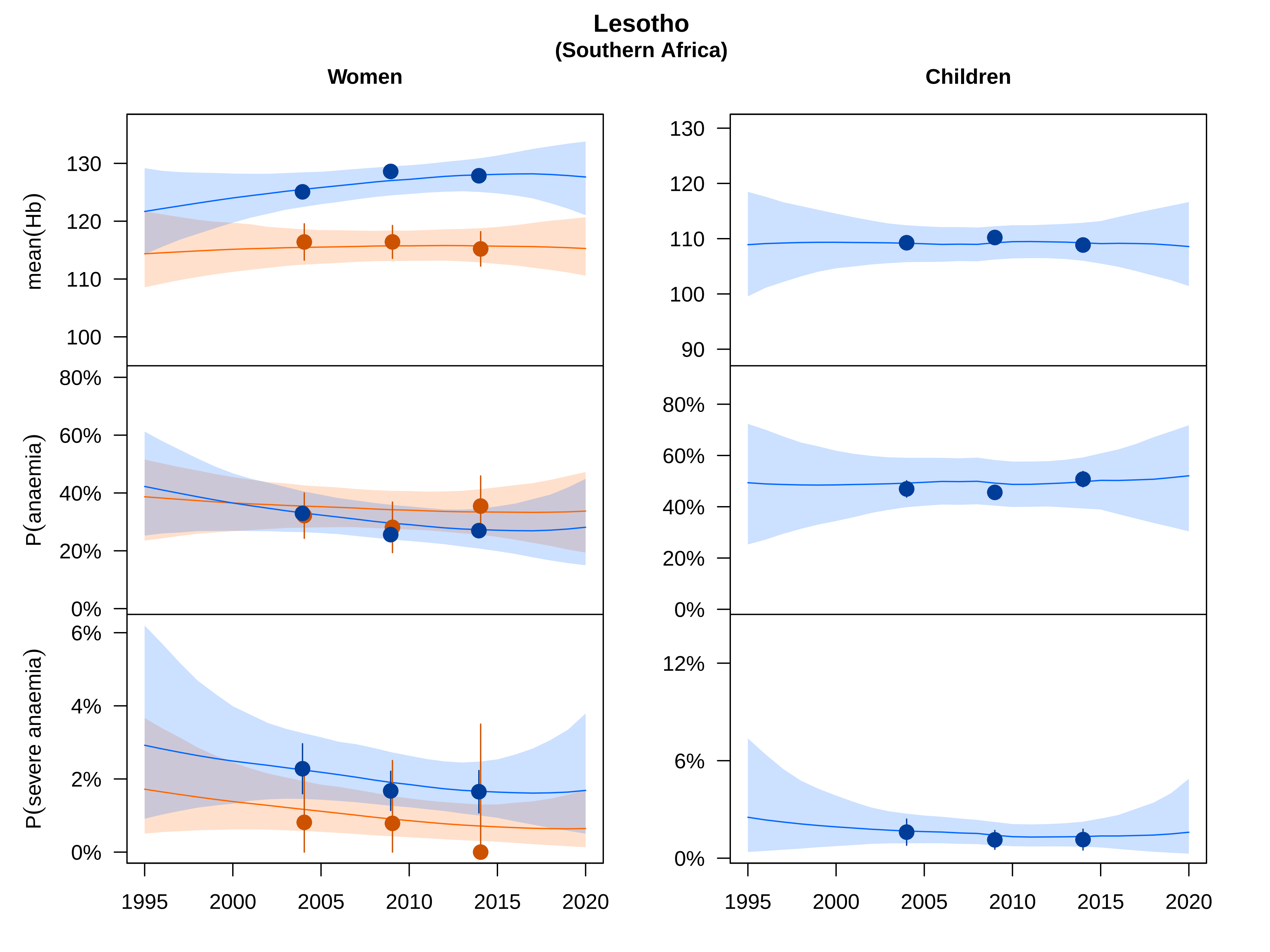

Lesotho
(Southern Africa)
Women
Children
mean(Hb)
P(anaemia)
P(severe anaemia)
130
120
110
100
90
80%
60%
40%
20%
0%
6%
4%
2%
12%
1995
2000
2005
2010
2015
2020

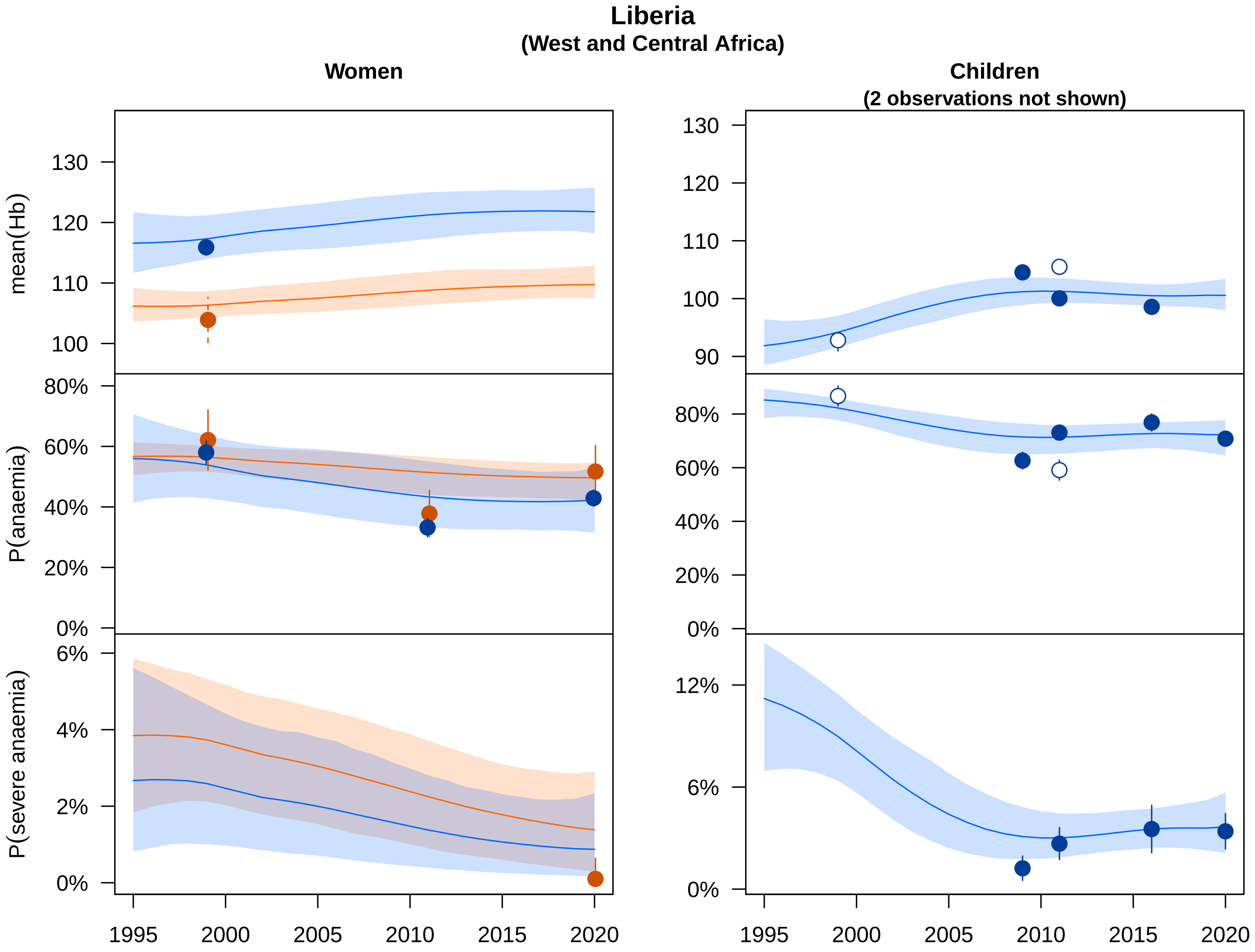
Liberia
(West and Central Africa)
Women
Children
(2 observations not shown)
mean(Hb)
P(anaemia)
P(severe anaemia)
130
120
110
100
90
80%
60%
40%
20%
0%
6%
4%
2%
12%
1995
2000
2005
2010
2015
2020

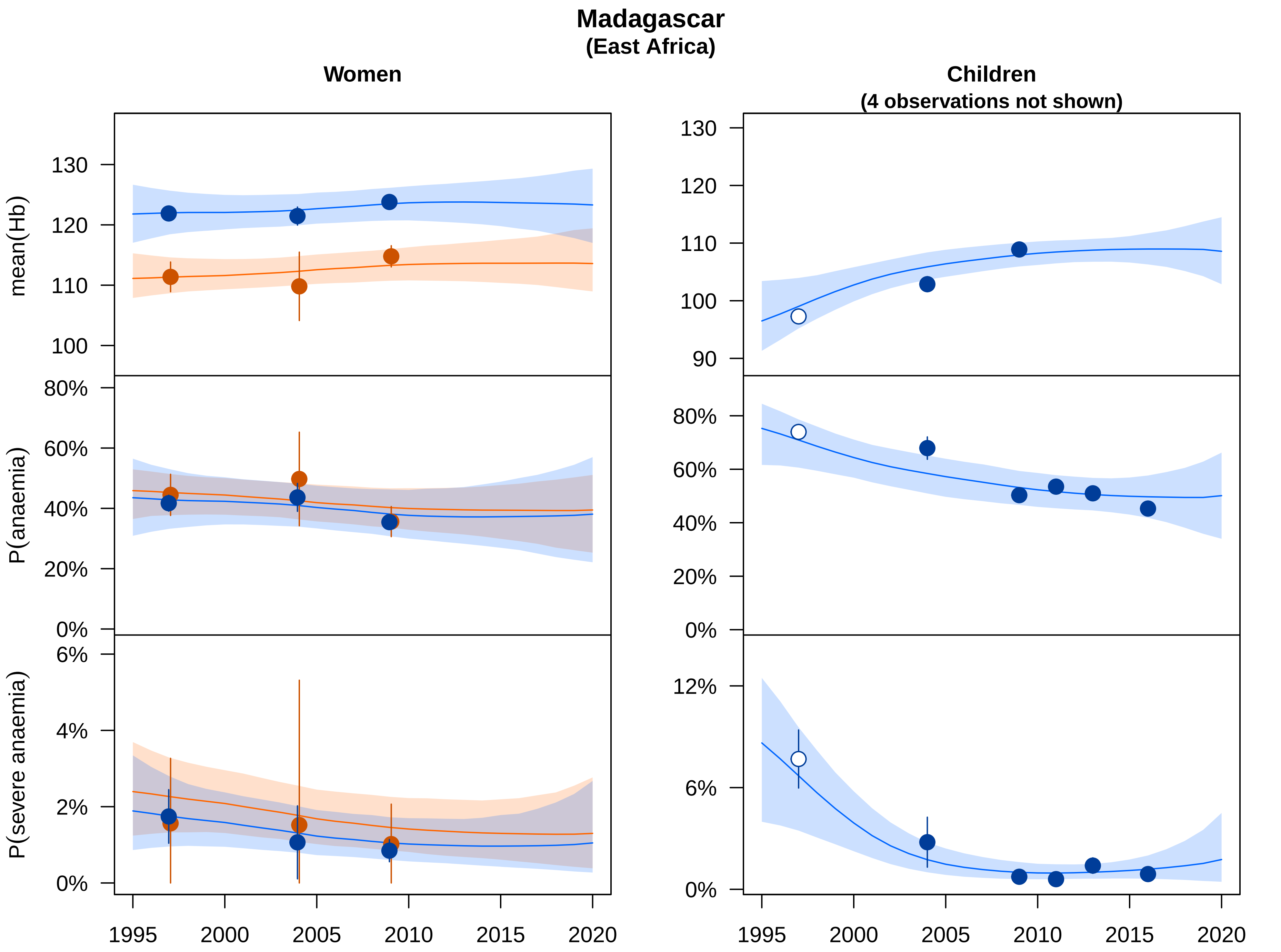
Madagascar
(East Africa)
Women
Children
(4 observations not shown)
mean(Hb)
P(anaemia)
P(severe anaemia)
130
120
110
100
90
80%
60%
40%
20%
0%
6%
4%
2%
12%
1995
2000
2005
2010
2015
2020

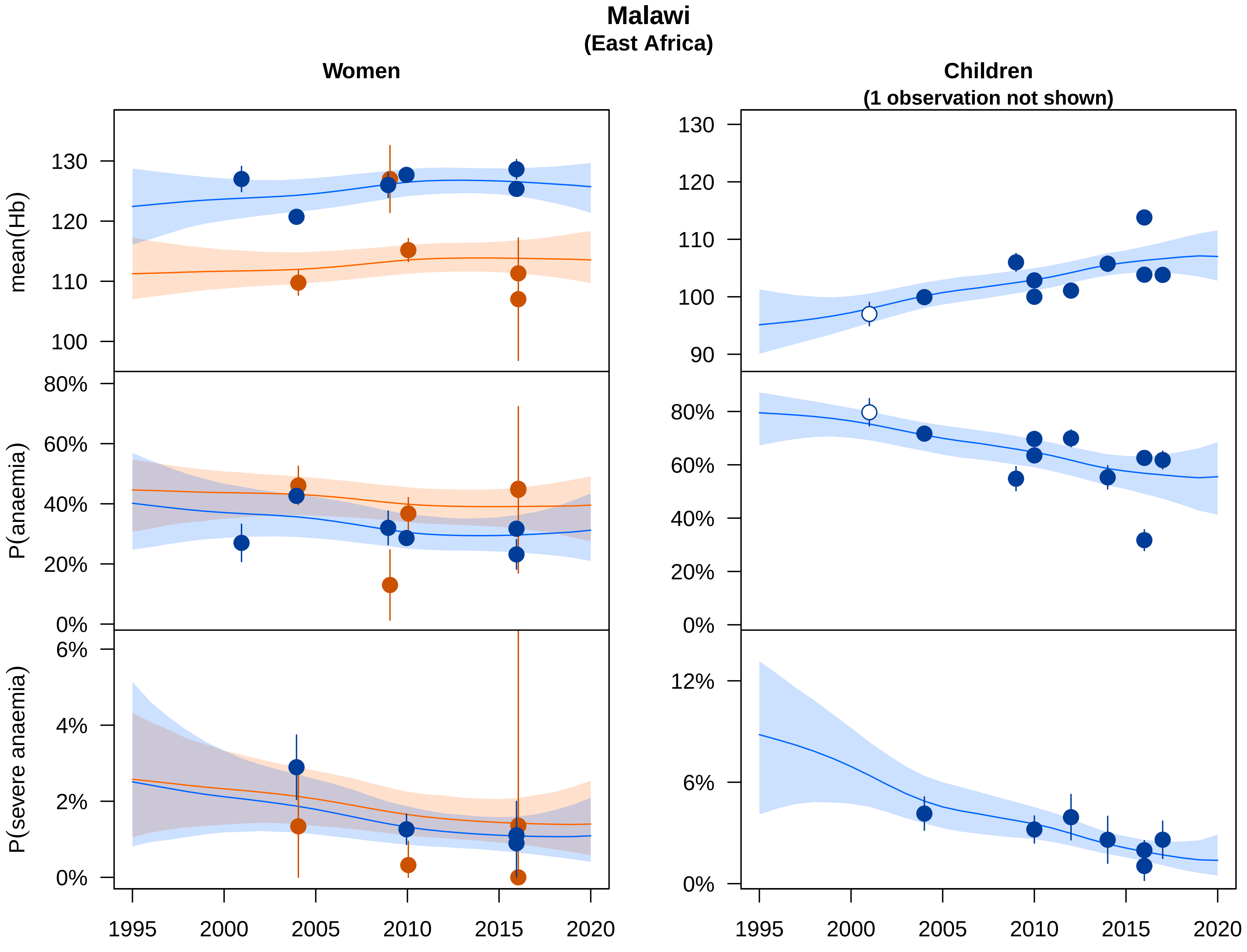
Malawi
(East Africa)
Women
Children
(1 observation not shown)
mean(Hb)
P(anaemia)
P(severe anaemia)
130
120
110
100
90
80%
60%
40%
20%
0%
6%
4%
2%
12%
1995
2000
2005
2010
2015
2020

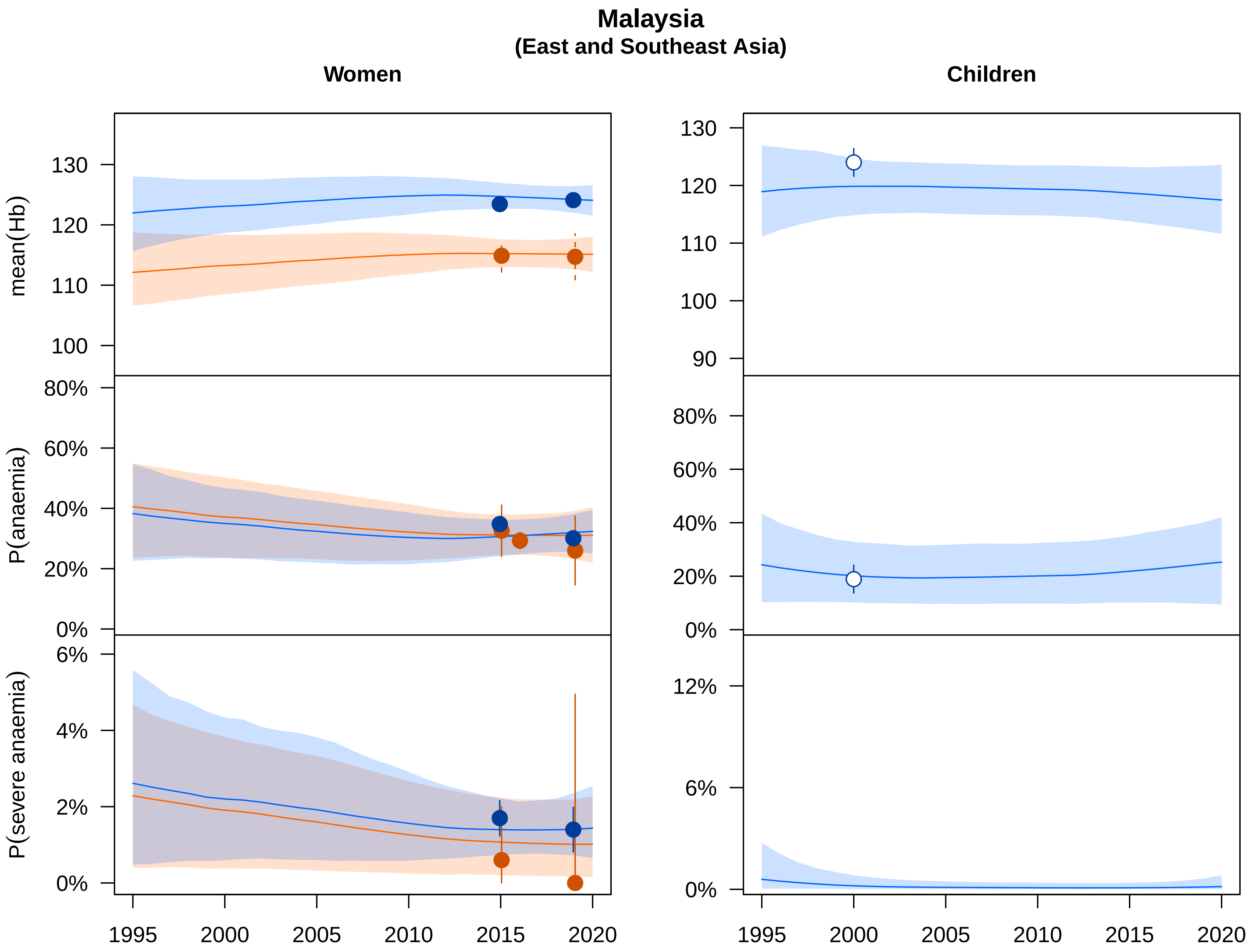
Malaysia
(East and Southeast Asia)
Women
Children
mean(Hb)
P(anaemia)
P(severe anaemia)
130
120
110
100
90
80%
60%
40%
20%
0%
6%
4%
2%
12%
1995
2000
2005
2010
2015
2020

Maldives
(East and Southeast Asia)

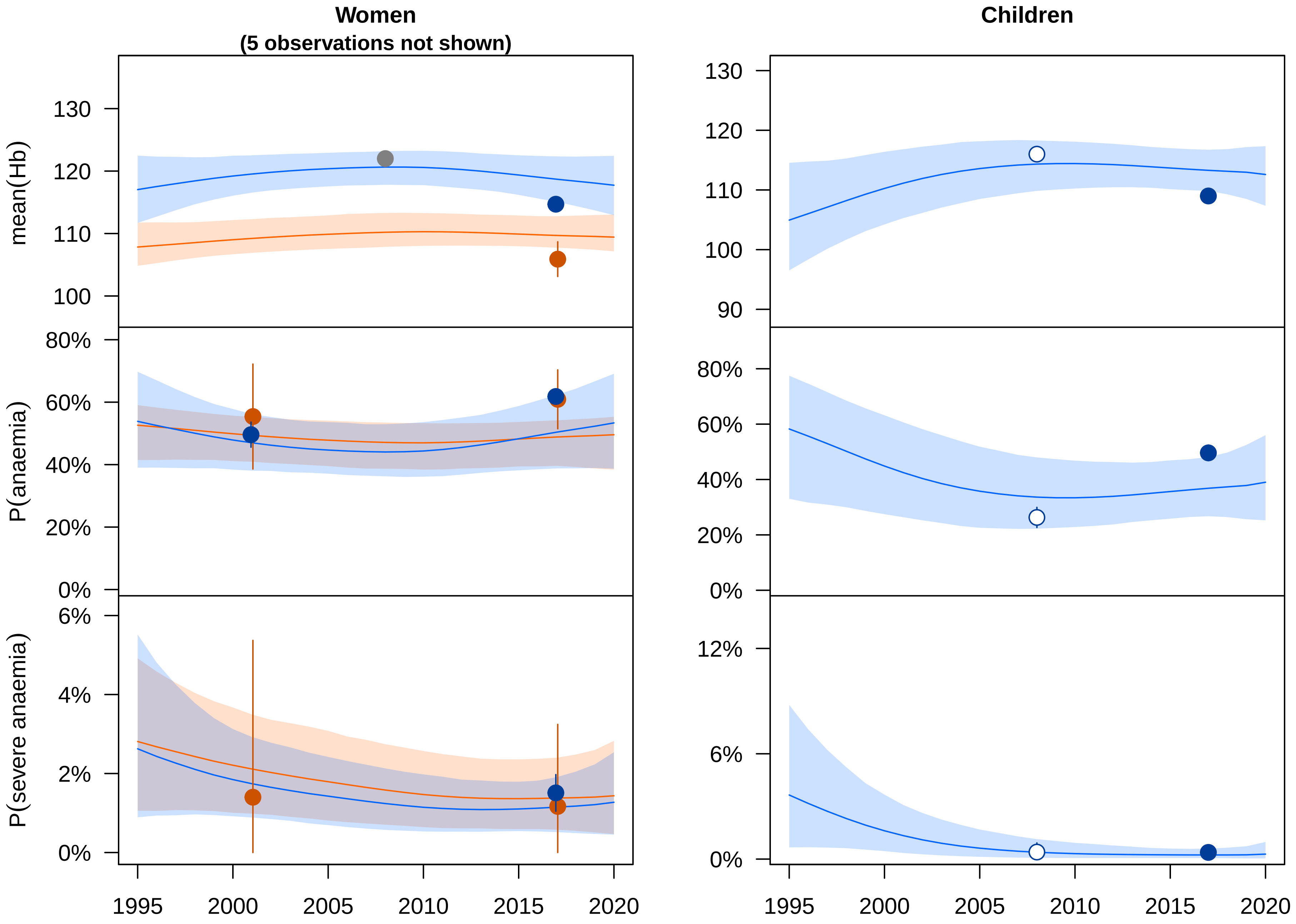

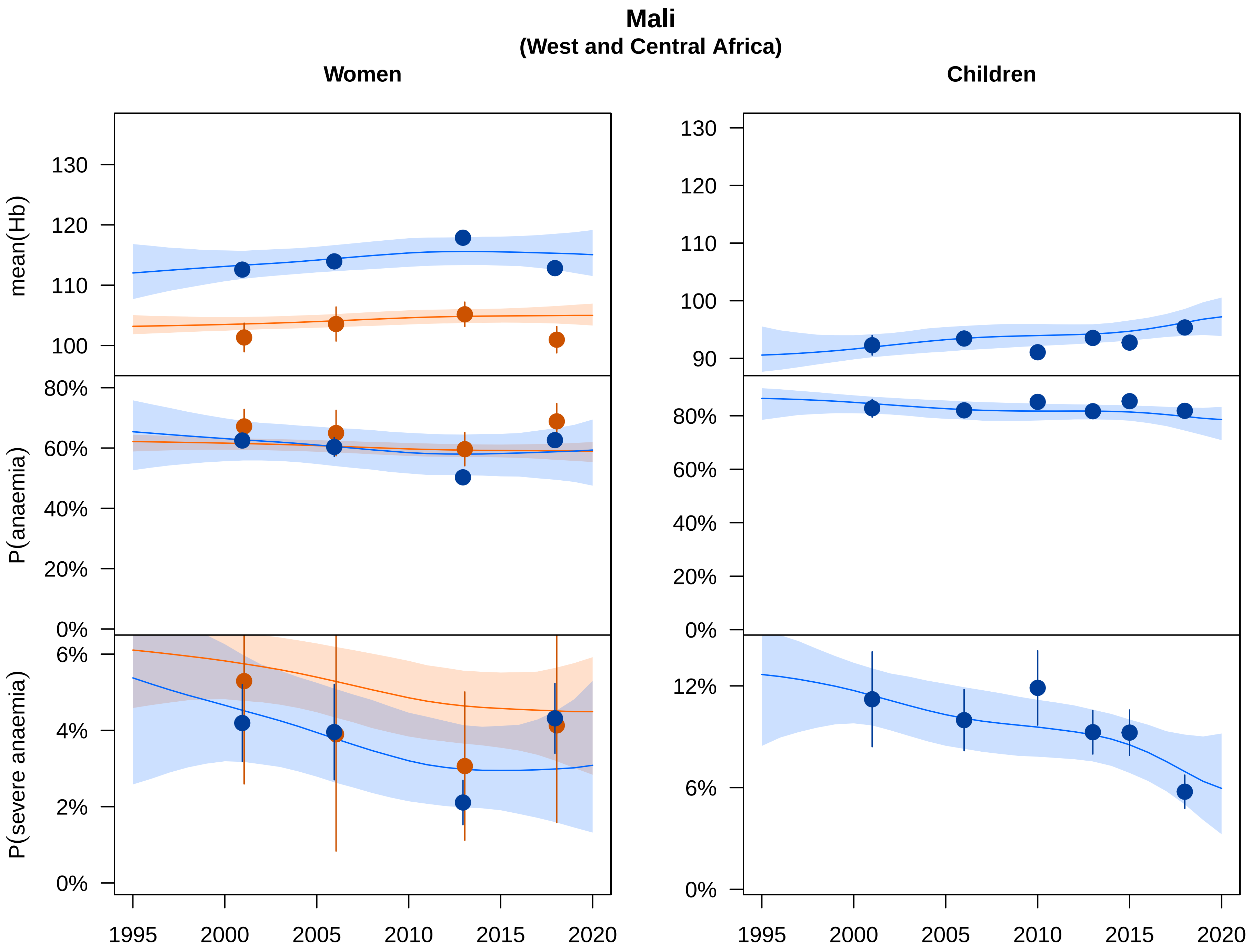

Mali
(West and Central Africa)
Women
Children
mean(Hb)
P(anaemia)
P(severe anaemia)
130
120
110
100
90
80%
60%
40%
20%
0%
6%
4%
2%
12%
1995
2000
2005
2010
2015
2020

Marshall Islands
(Oceania)

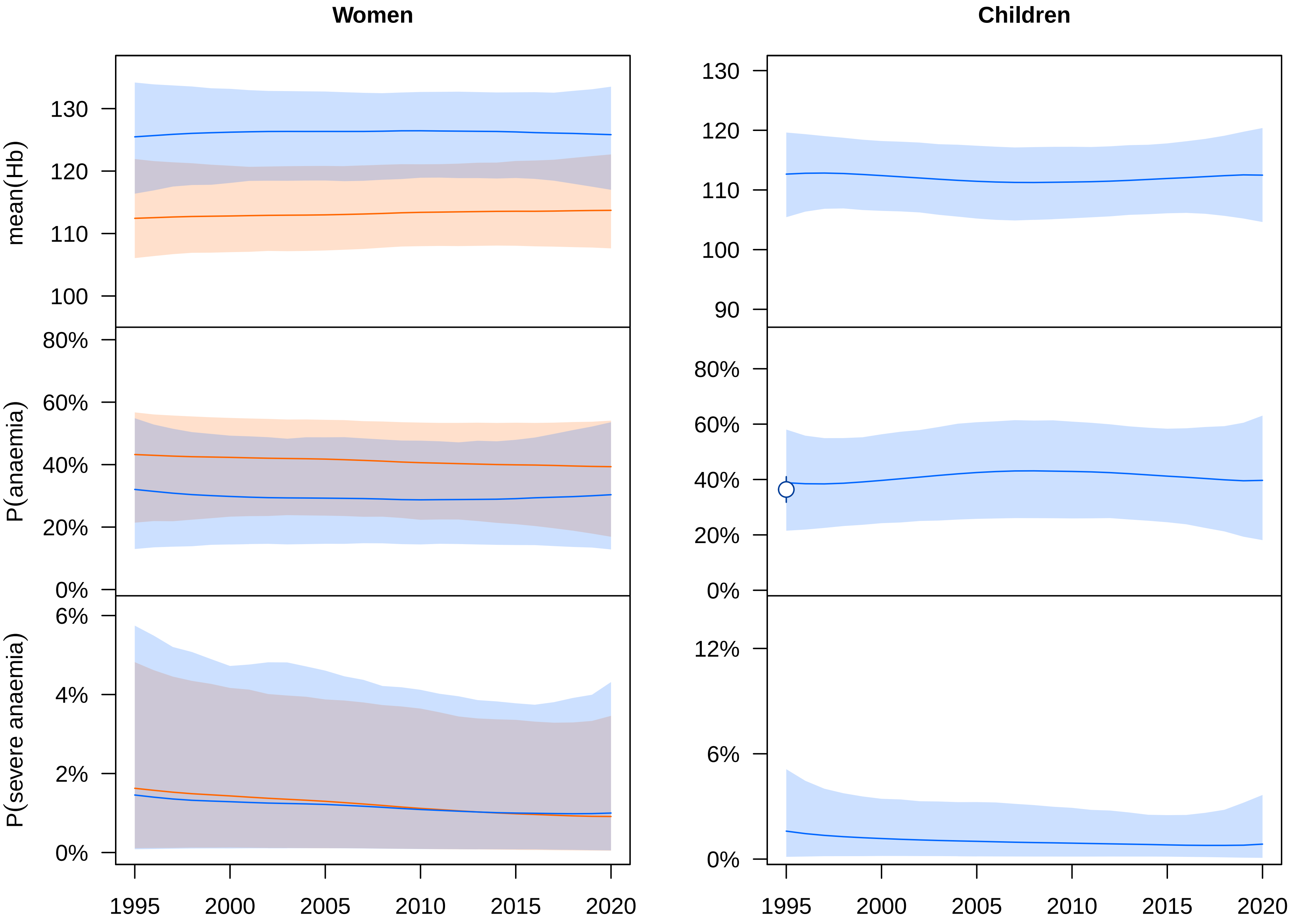

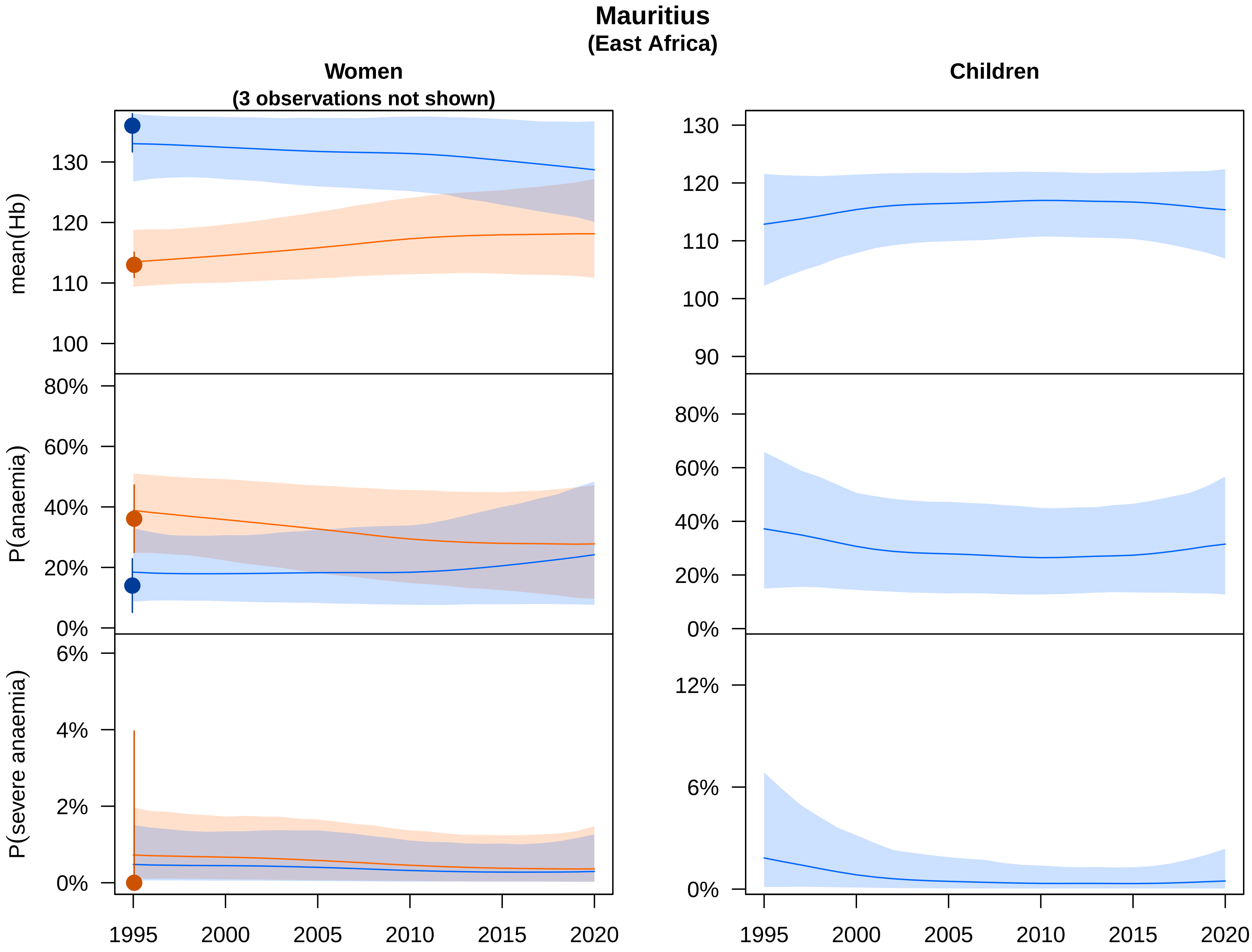
Mauritius
(East Africa)
Women
(3 observations not shown)
Children
mean(Hb)
P(anaemia)
P(severe anaemia)
130
120
110
100
90
80%
60%
40%
20%
0%
6%
4%
2%
12%
1995
2000
2005
2010
2015
2020

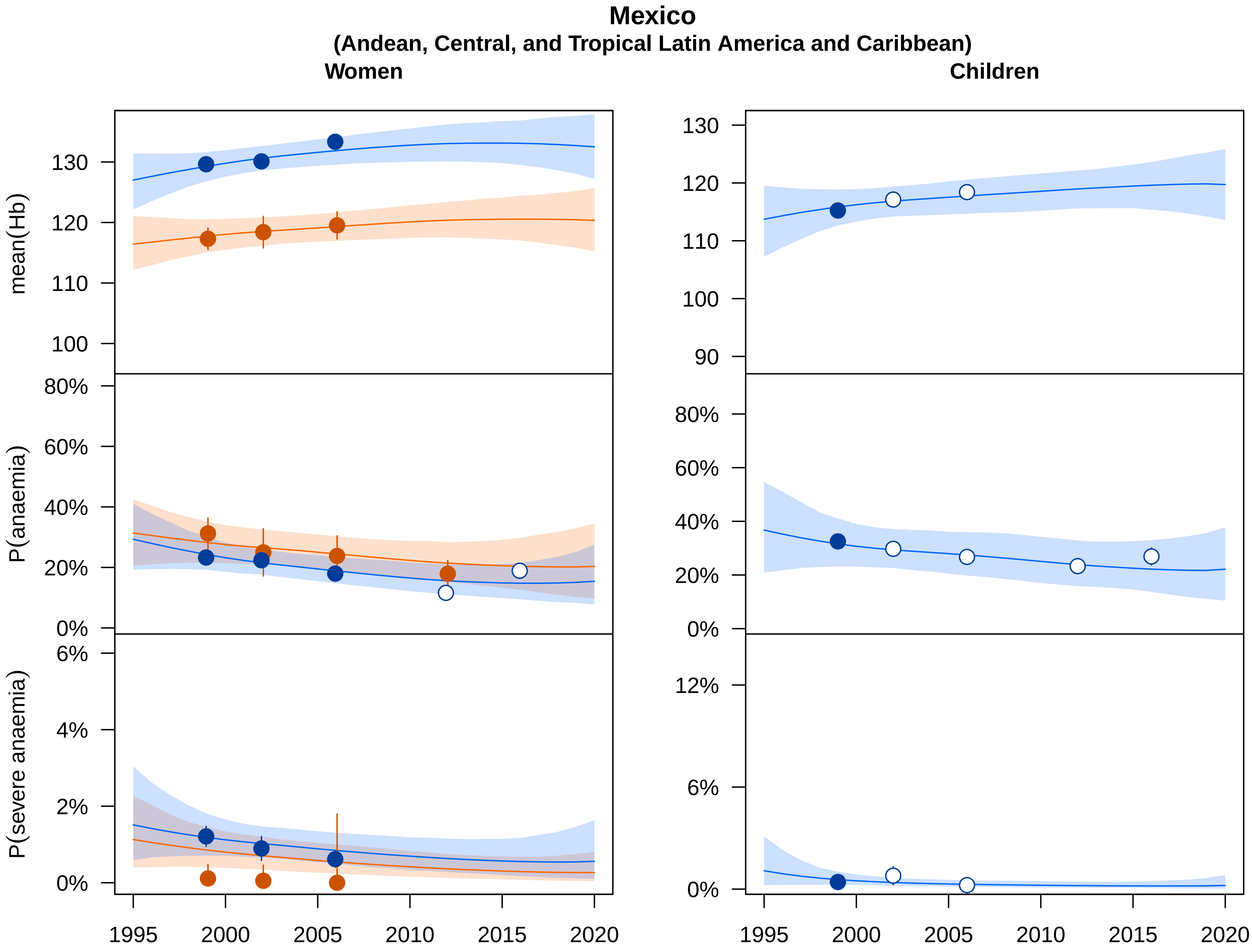

Mexico
(Andean, Central, and Tropical Latin America and Caribbean)
Women
Children
mean(Hb)
P(anaemia)
P(severe anaemia)
100
110
120
130
90
0%
20%
40%
60%
80%
2%
4%
6%
12%
1995
2000
2005
2010
2015
2020

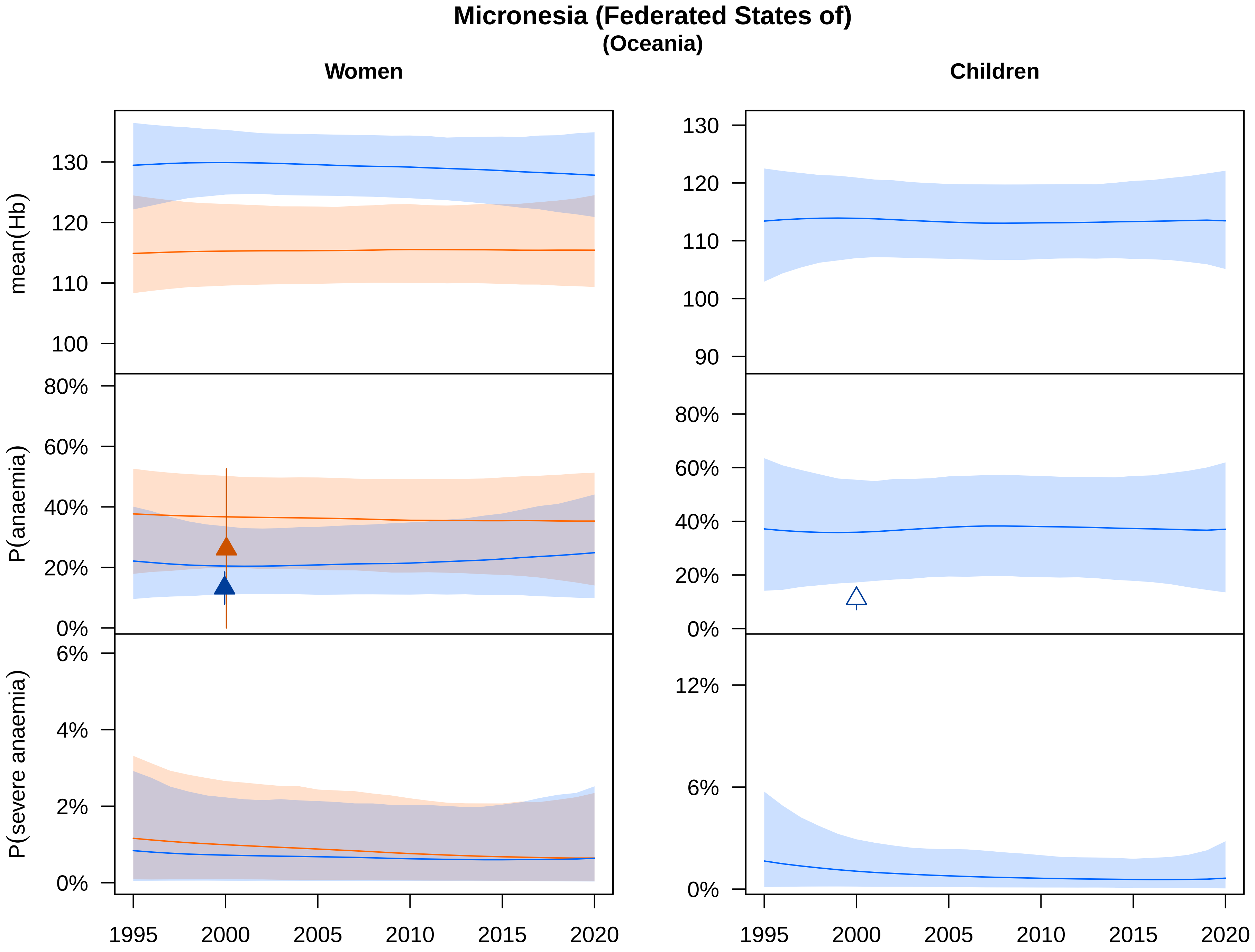
Micronesia (Federated States of)
(Oceania)
Women
Children
mean(Hb)
P(anaemia)
P(severe anaemia)
130
120
110
100
90
80%
60%
40%
20%
0%
6%
4%
2%
12%
1995
2000
2005
2010
2015
2020

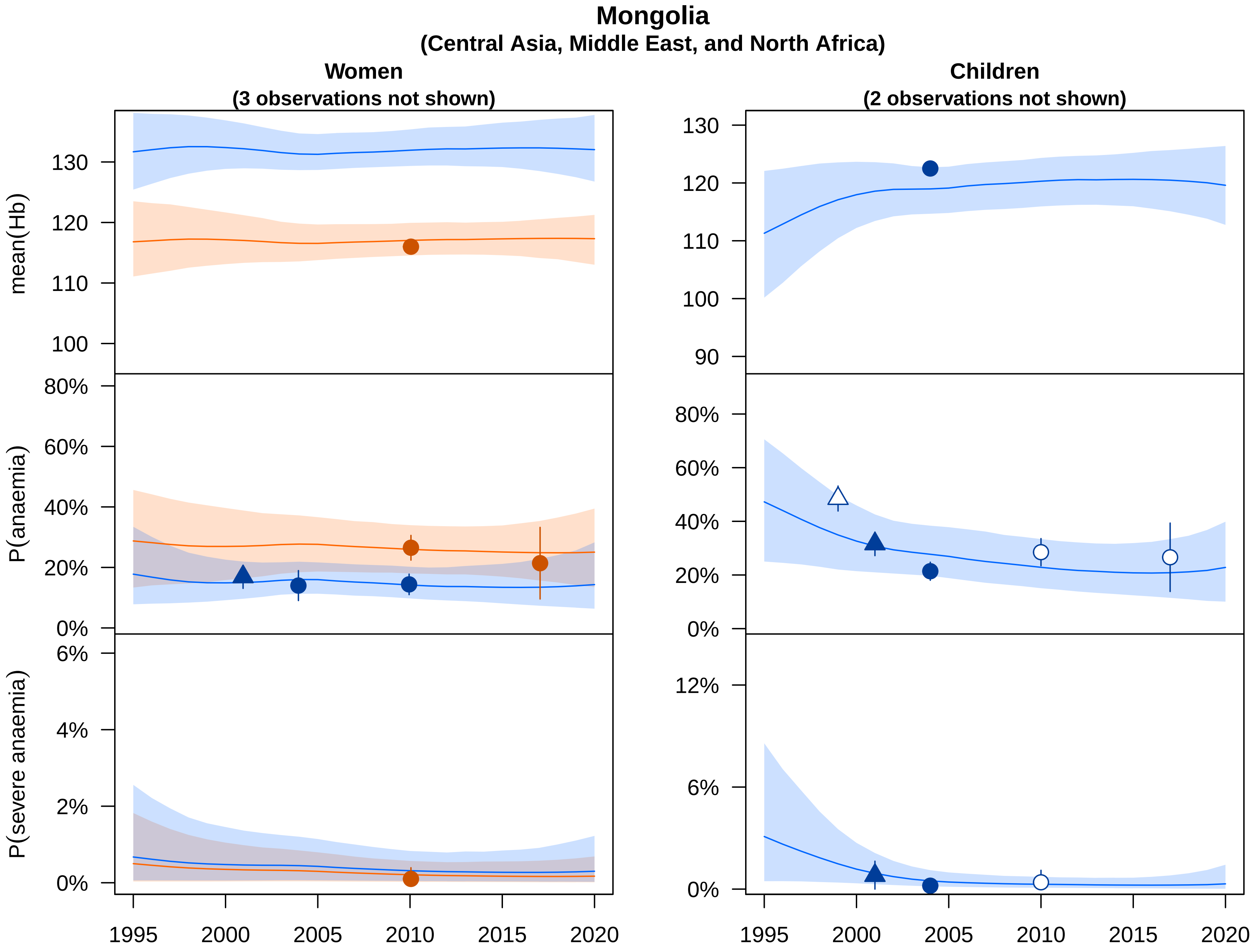

Mongolia
(Central Asia, Middle East, and North Africa)
Women
(3 observations not shown)
Children
(2 observations not shown)
mean(Hb)
P(anaemia)
P(severe anaemia)
130
120
110
100
90
80%
60%
40%
20%
0%
6%
4%
2%
12%
1995
2000
2005
2010
2015
2020

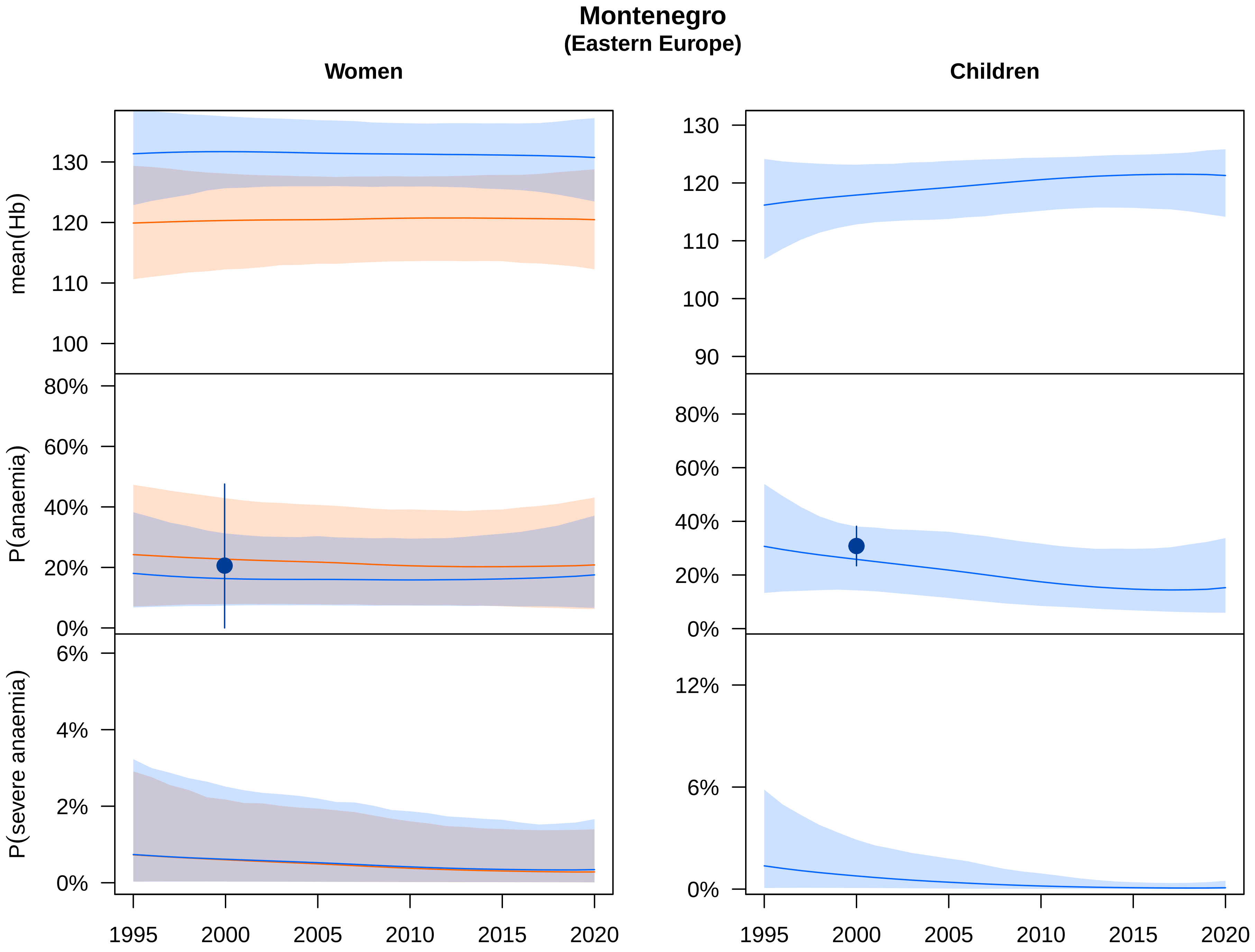

Montenegro
(Eastern Europe)
Women
Children
mean(Hb)
P(anaemia)
P(severe anaemia)
130
120
110
100
90
80%
60%
40%
20%
0%
6%
4%
2%
12%
1995
2000
2005
2010
2015
2020

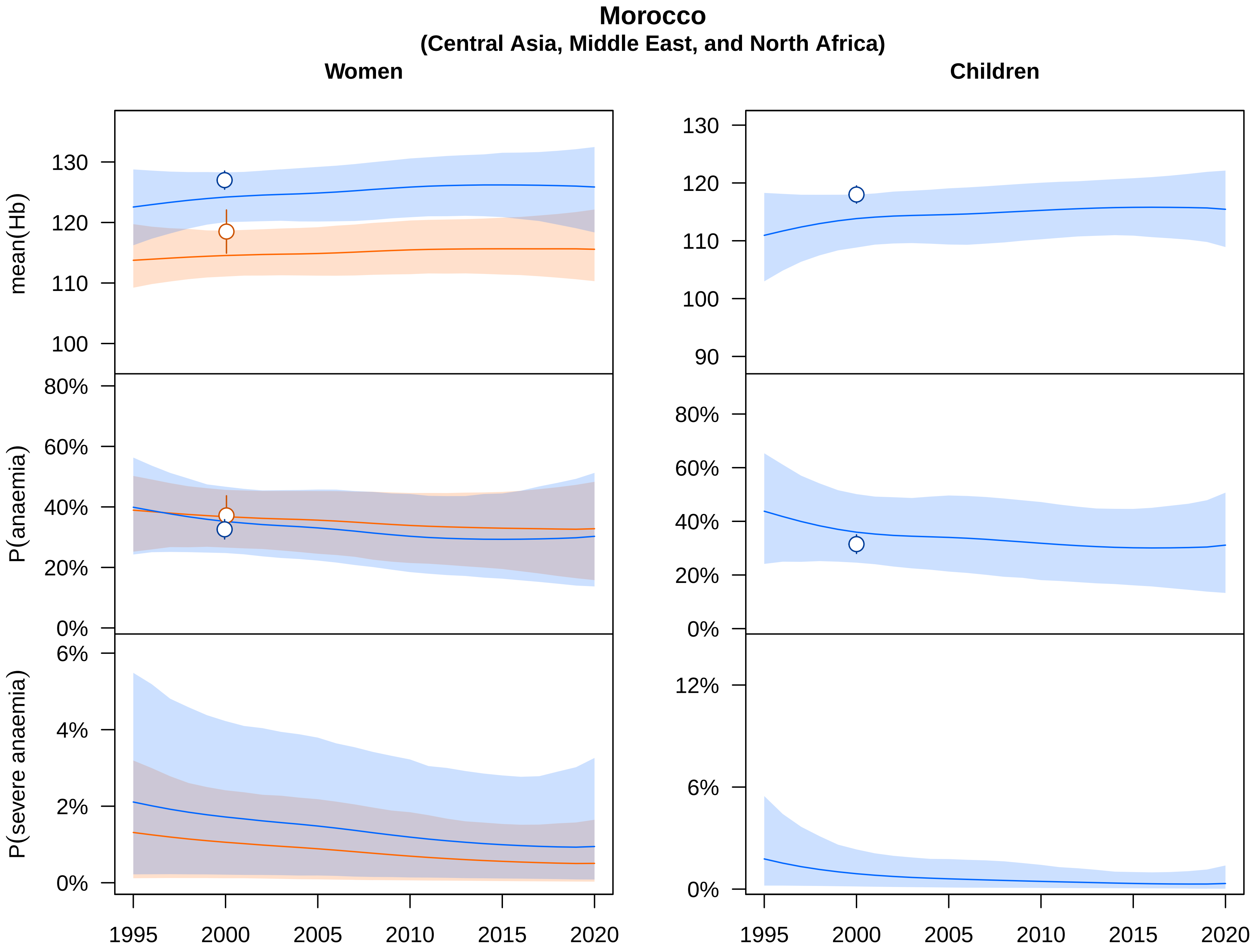
Morocco
(Central Asia, Middle East, and North Africa)
Women
Children
mean(Hb)
P(anaemia)
P(severe anaemia)
130
120
110
100
90
80%
60%
40%
20%
0%
6%
4%
2%
12%
1995
2000
2005
2010
2015
2020

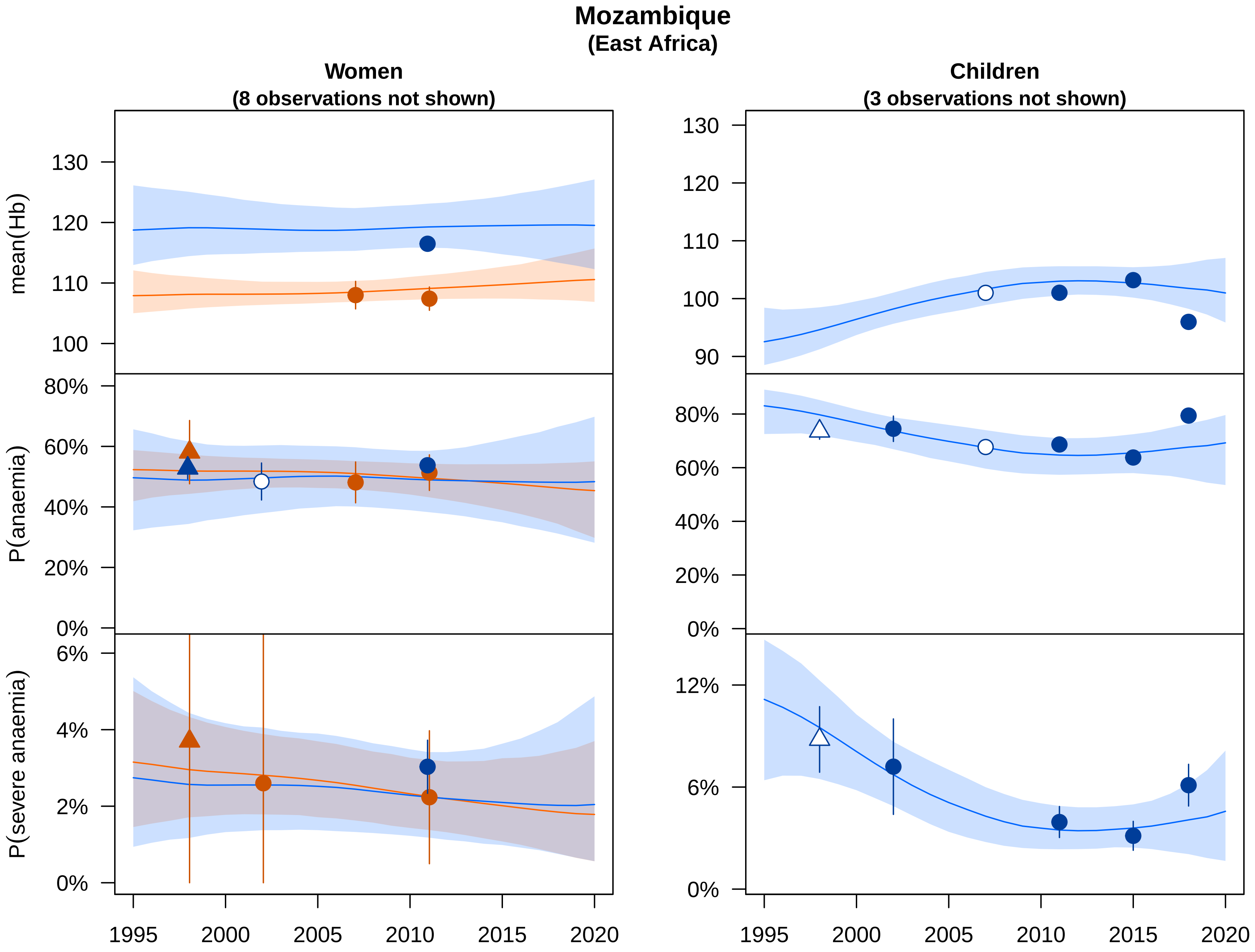
Mozambique
(East Africa)
Women
(8 observations not shown)
Children
(3 observations not shown)
mean(Hb)
P(anaemia)
P(severe anaemia)
130
120
110
100
90
80%
60%
40%
20%
0%
6%
4%
2%
12%
1995
2000
2005
2010
2015
2020

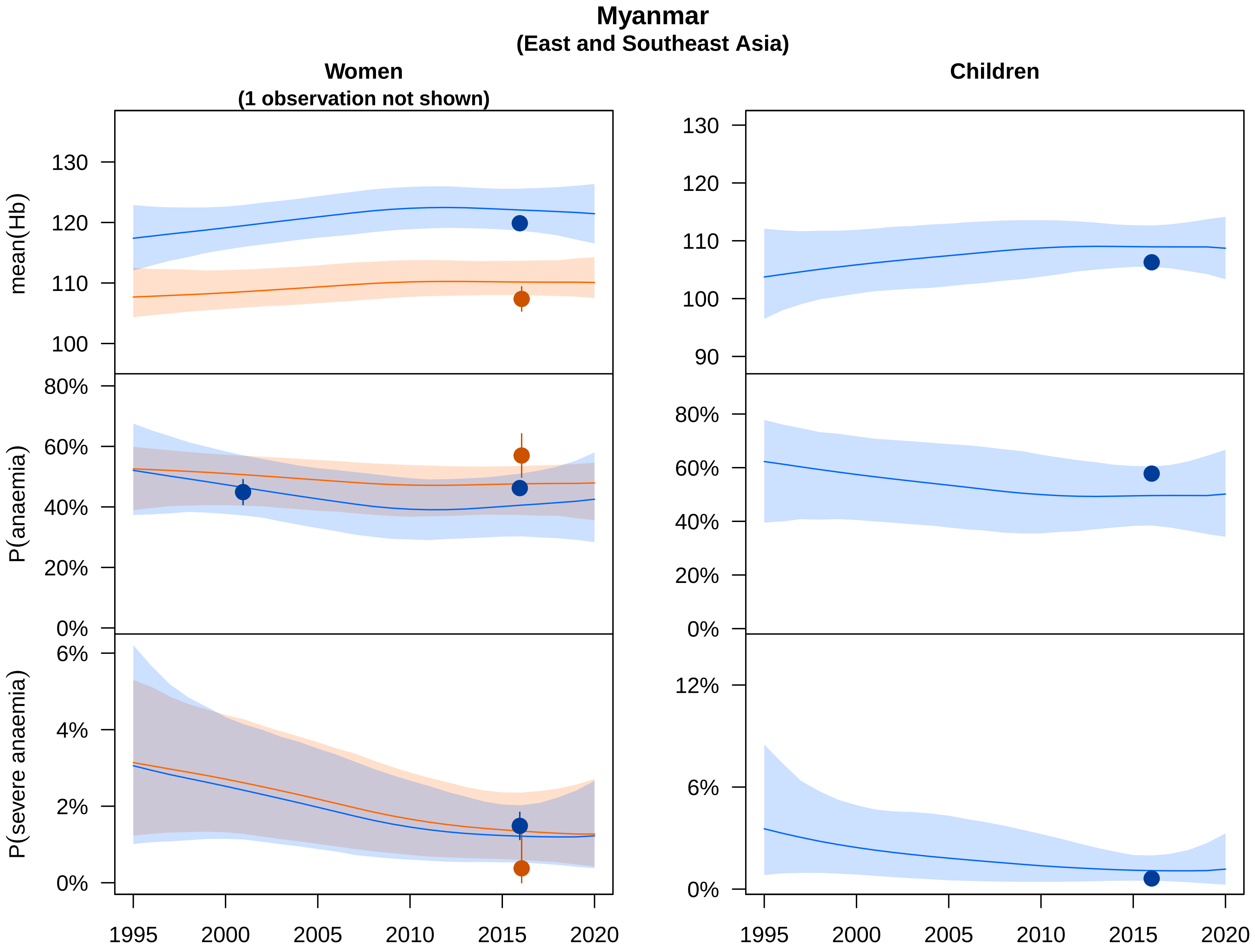
Myanmar
(East and Southeast Asia)
Women
(1 observation not shown)
Children
mean(Hb)
P(anaemia)
P(severe anaemia)
130
120
110
100
90
80%
60%
40%
20%
0%
6%
4%
2%
12%
1995
2000
2005
2010
2015
2020

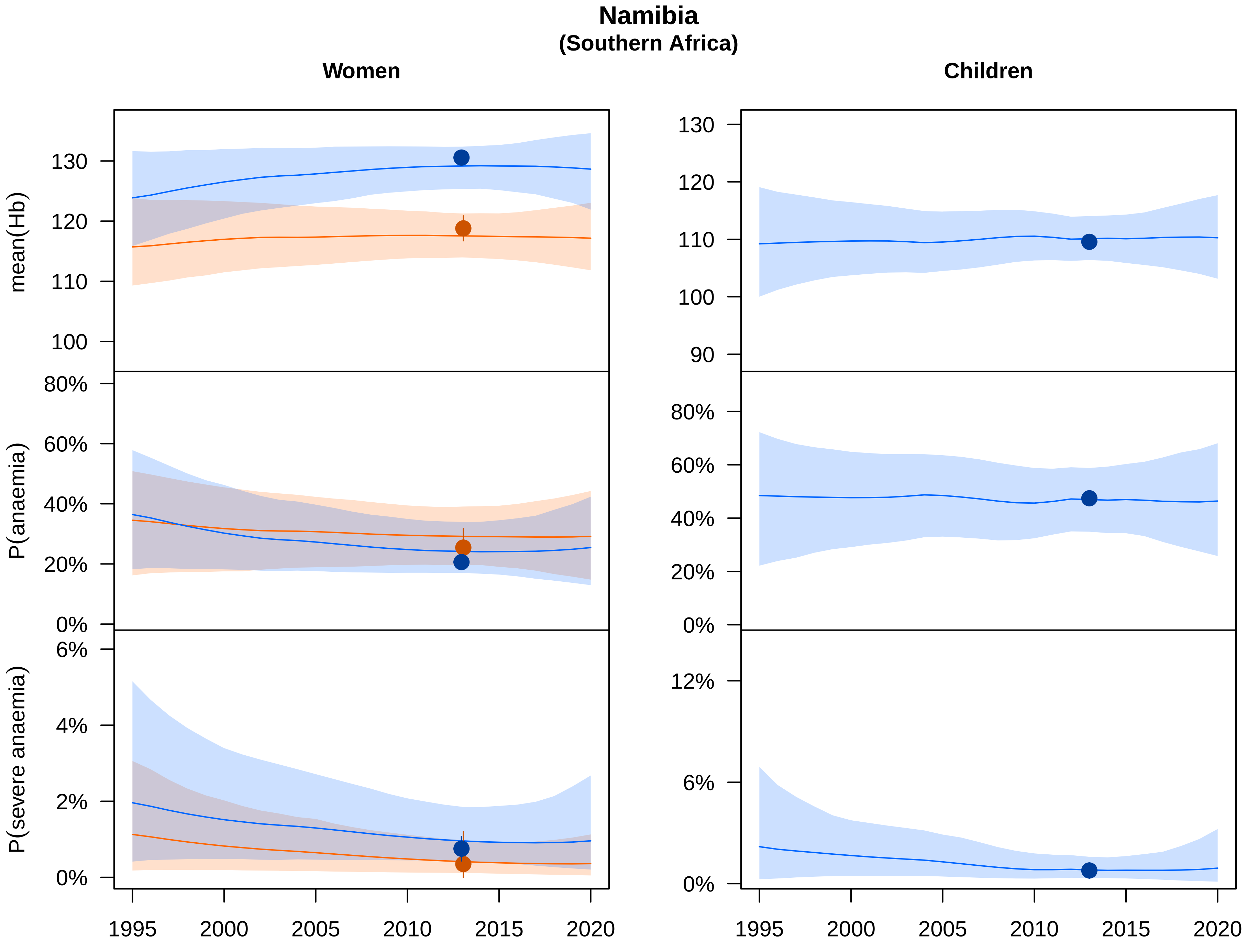
Namibia
(Southern Africa)
Women
Children
mean(Hb)
P(anaemia)
P(severe anaemia)
130
120
110
100
90
80%
60%
40%
20%
0%
6%
4%
2%
12%
1995
2000
2005
2010
2015
2020

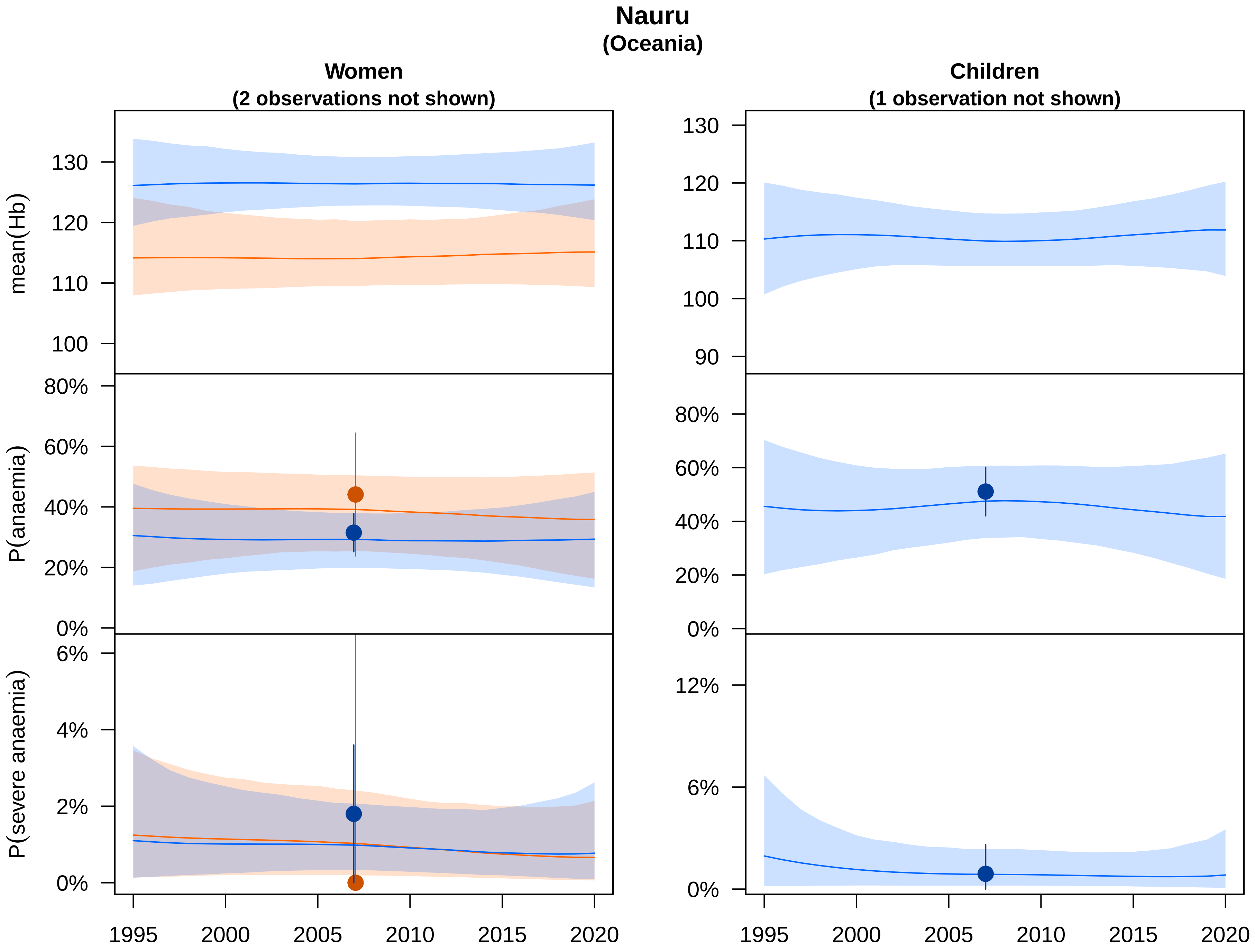

Nauru
(Oceania)
Women
(2 observations not shown)
Children
(1 observation not shown)
mean(Hb)
P(anaemia)
P(severe anaemia)
130
120
110
100
90
80%
60%
40%
20%
0%
6%
4%
2%
12%
1995
2000
2005
2010
2015
2020

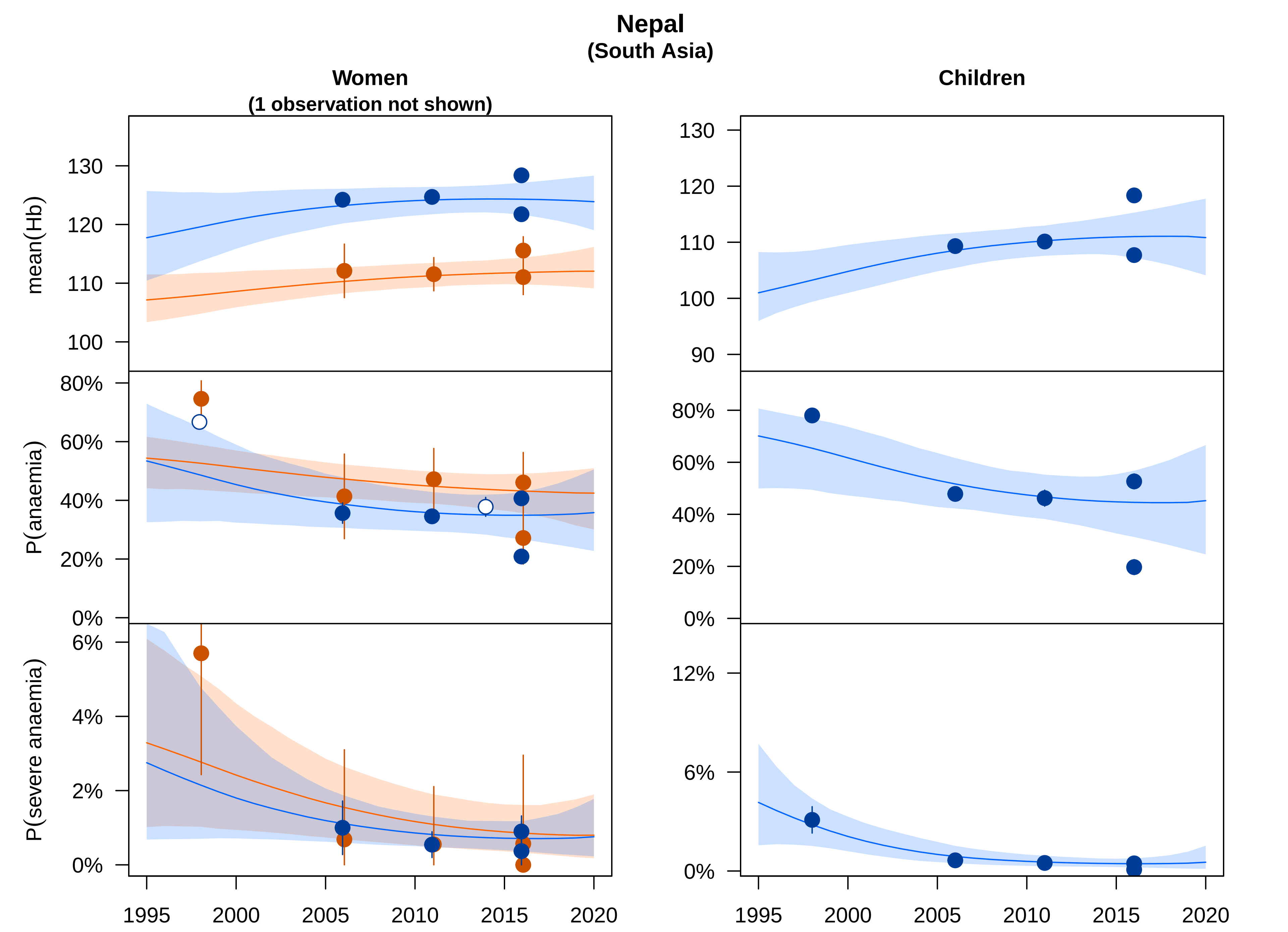

Nepal
(South Asia)
Women
(1 observation not shown)
Children
mean(Hb)
130
120
110
100
90
P(anaemia)
80%
60%
40%
20%
0%
P(severe anaemia)
6%
4%
2%
12%
1995
2000
2005
2010
2015
2020

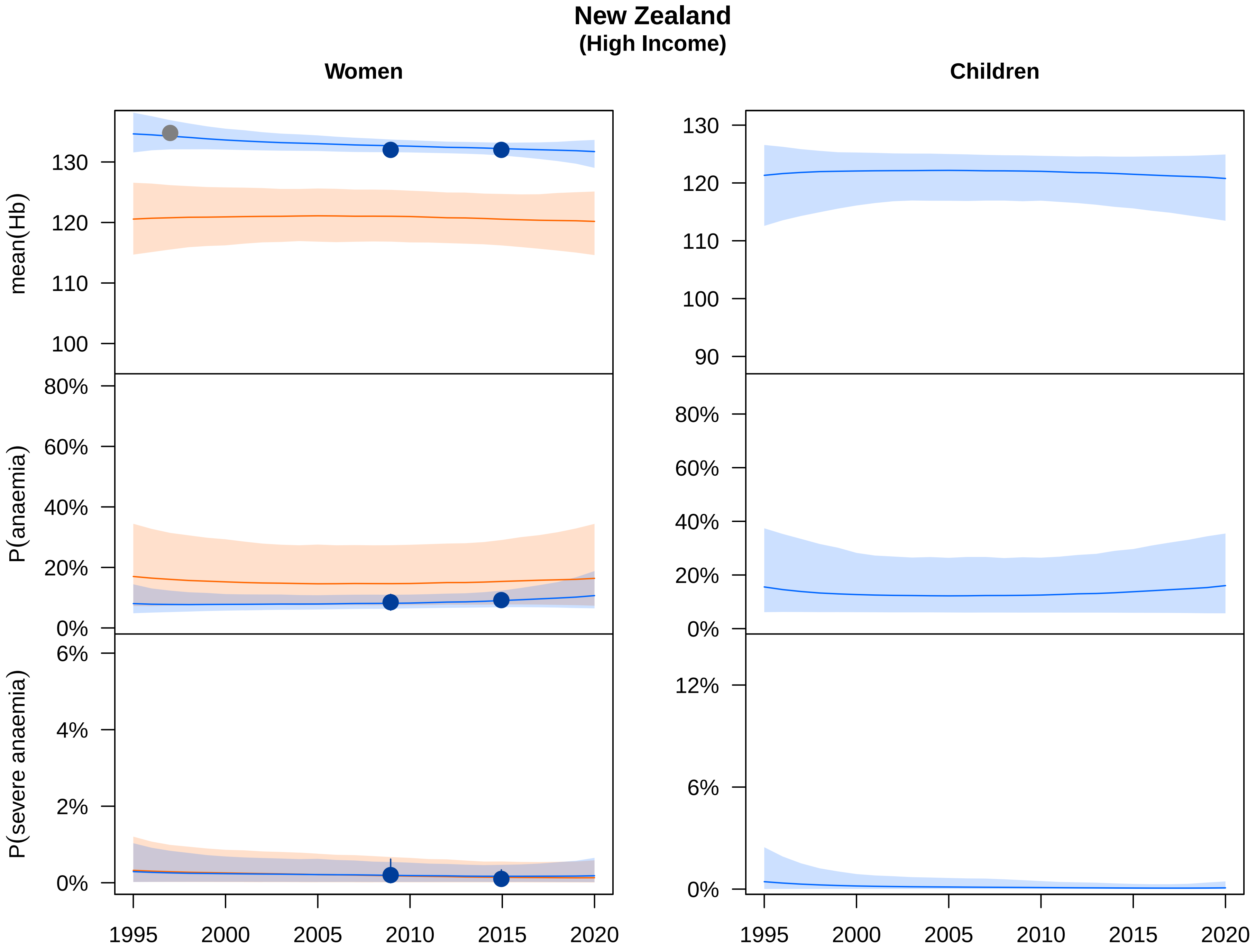
New Zealand
(High Income)
Women
Children
mean(Hb)
P(anaemia)
P(severe anaemia)
130
120
110
100
90
80%
60%
40%
20%
0%
6%
4%
2%
12%
1995
2000
2005
2010
2015
2020

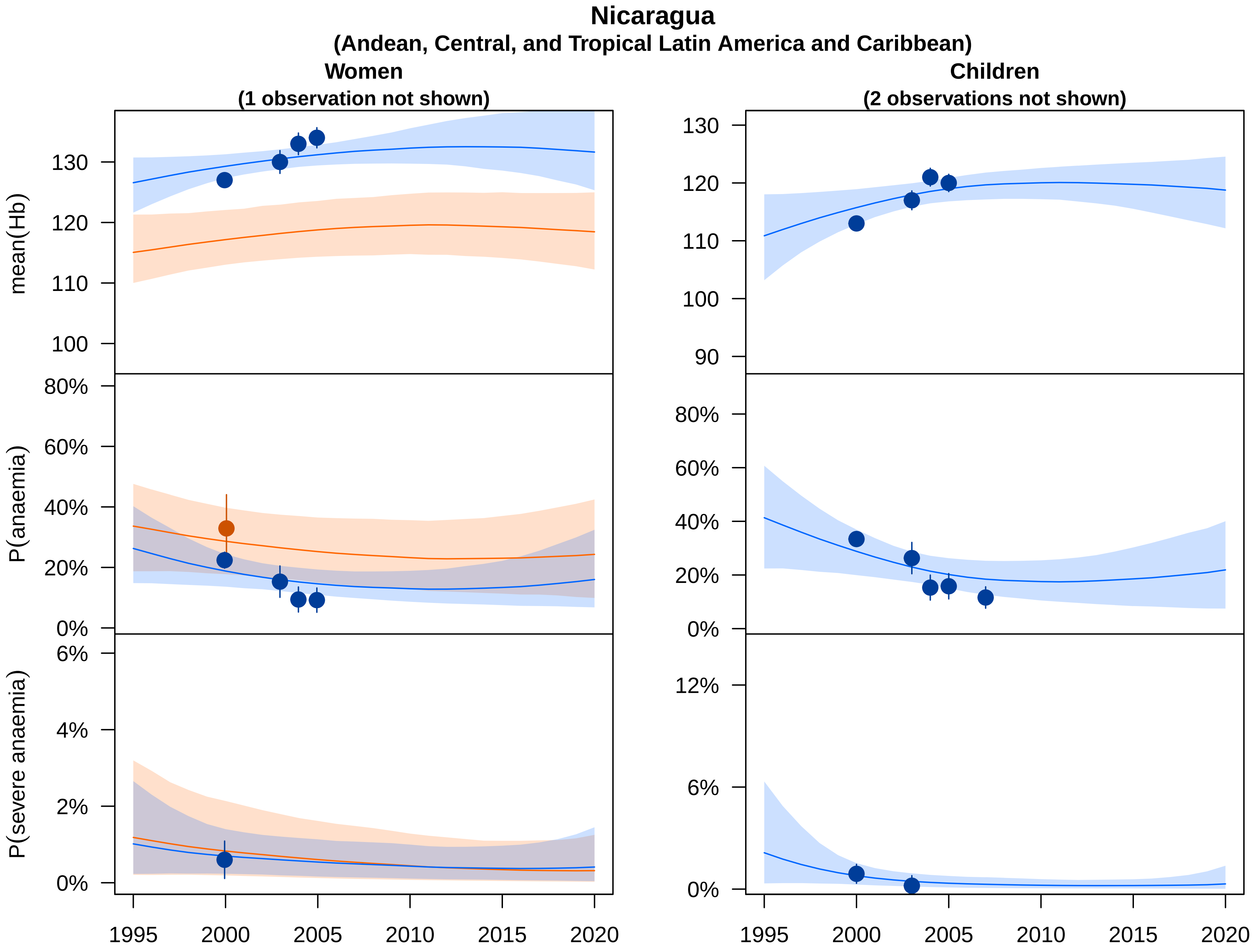
Nicaragua
(Andean, Central, and Tropical Latin America and Caribbean)
Women
(1 observation not shown)
Children
(2 observations not shown)
mean(Hb)
P(anaemia)
P(severe anaemia)
130
120
110
100
90
80%
60%
40%
20%
0%
6%
4%
2%
12%
1995
2000
2005
2010
2015
2020

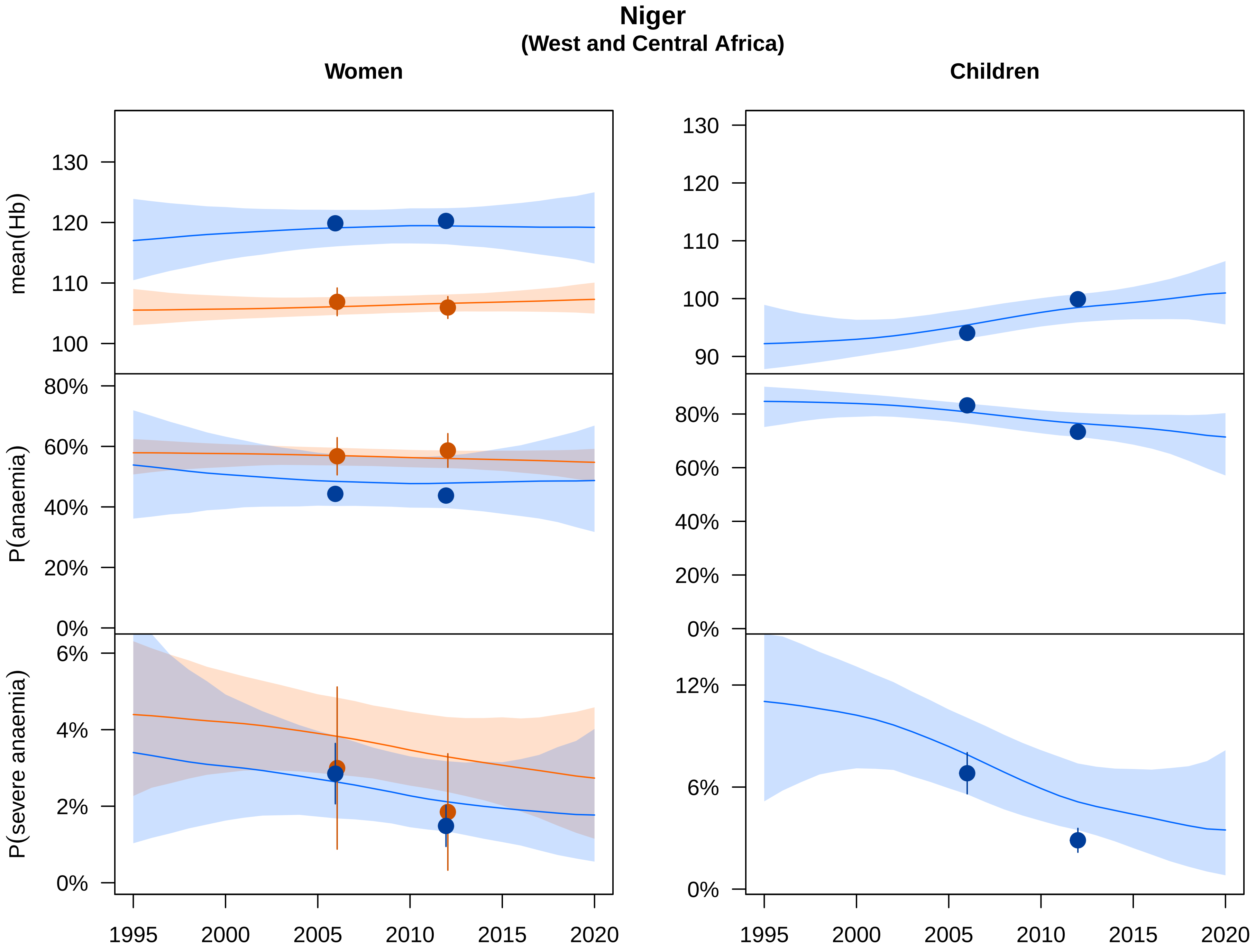

Niger
(West and Central Africa)
Women
Children
mean(Hb)
P(anaemia)
P(severe anaemia)
130
120
110
100
90
80%
60%
40%
20%
0%
6%
4%
2%
12%
1995
2000
2005
2010
2015
2020

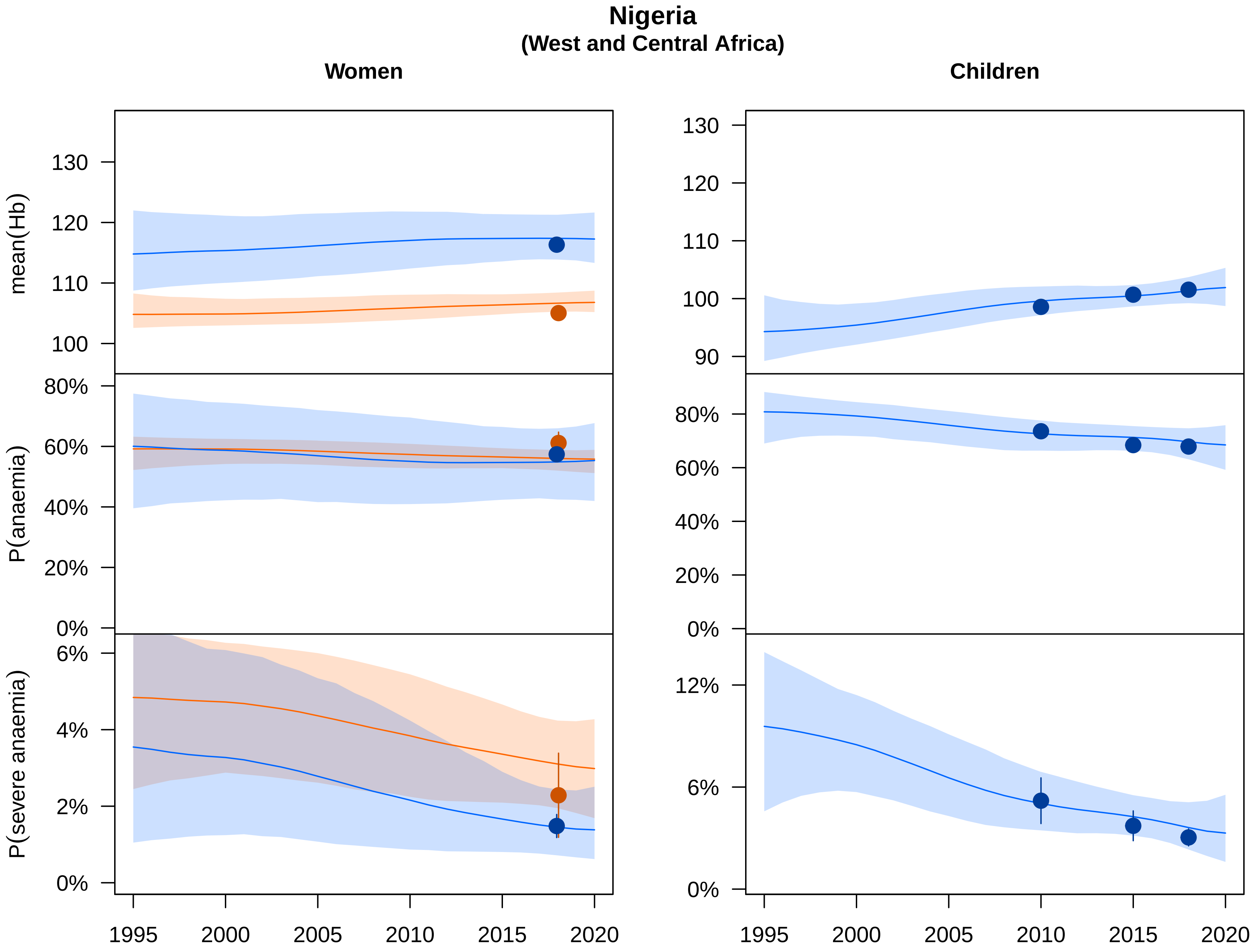
Nigeria
(West and Central Africa)
Women
Children
mean(Hb)
P(anaemia)
P(severe anaemia)
130
120
110
100
90
80%
60%
40%
20%
0%
6%
4%
2%
12%
1995
2000
2005
2010
2015
2020

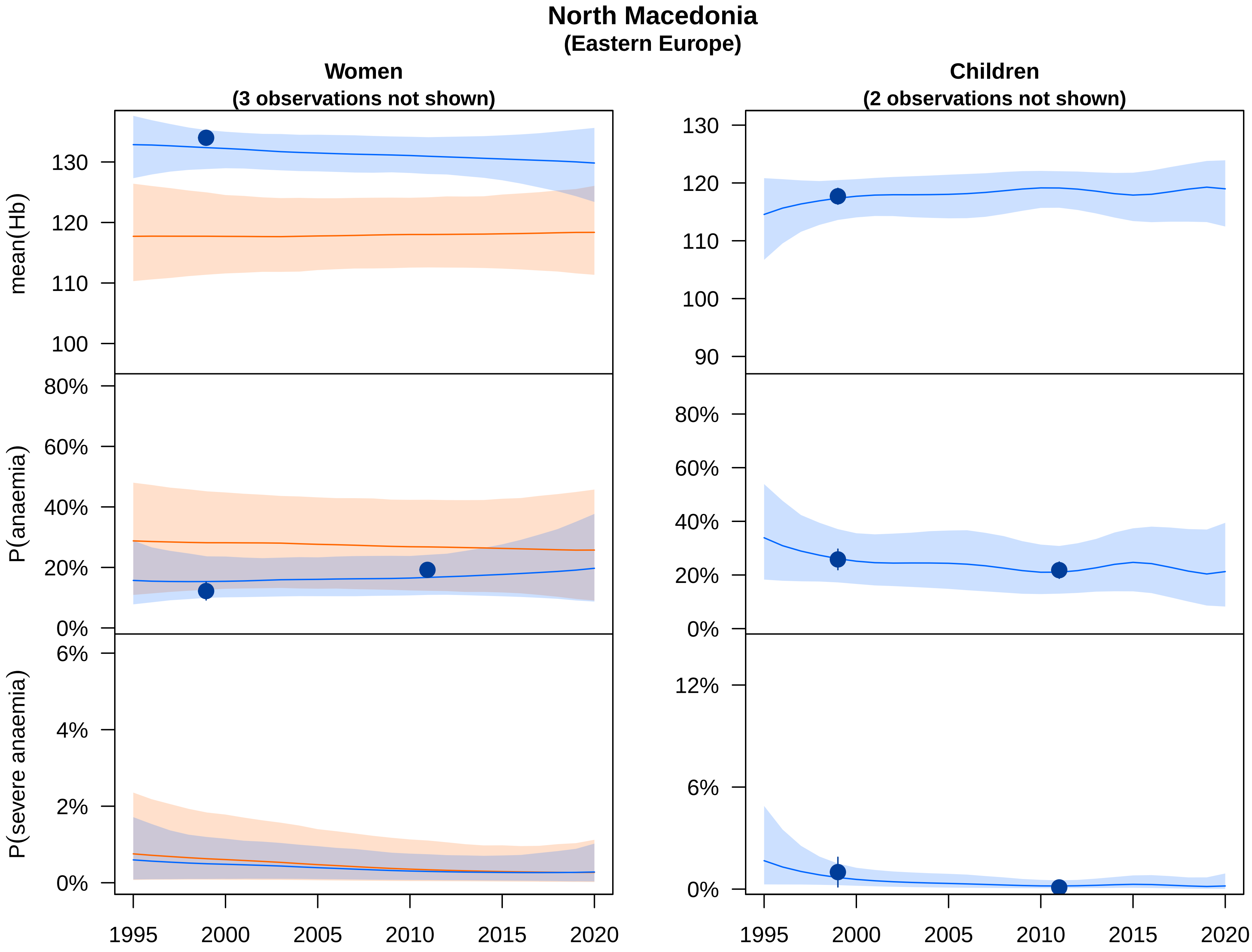

North Macedonia
(Eastern Europe)
Women
(3 observations not shown)
Children
(2 observations not shown)
mean(Hb)
P(anaemia)
P(severe anaemia)
130
120
110
100
90
80%
60%
40%
20%
0%
6%
4%
2%
12%
1995
2000
2005
2010
2015
2020

Occupied Palestinian Territory

(Central Asia, Middle East, and North Africa)

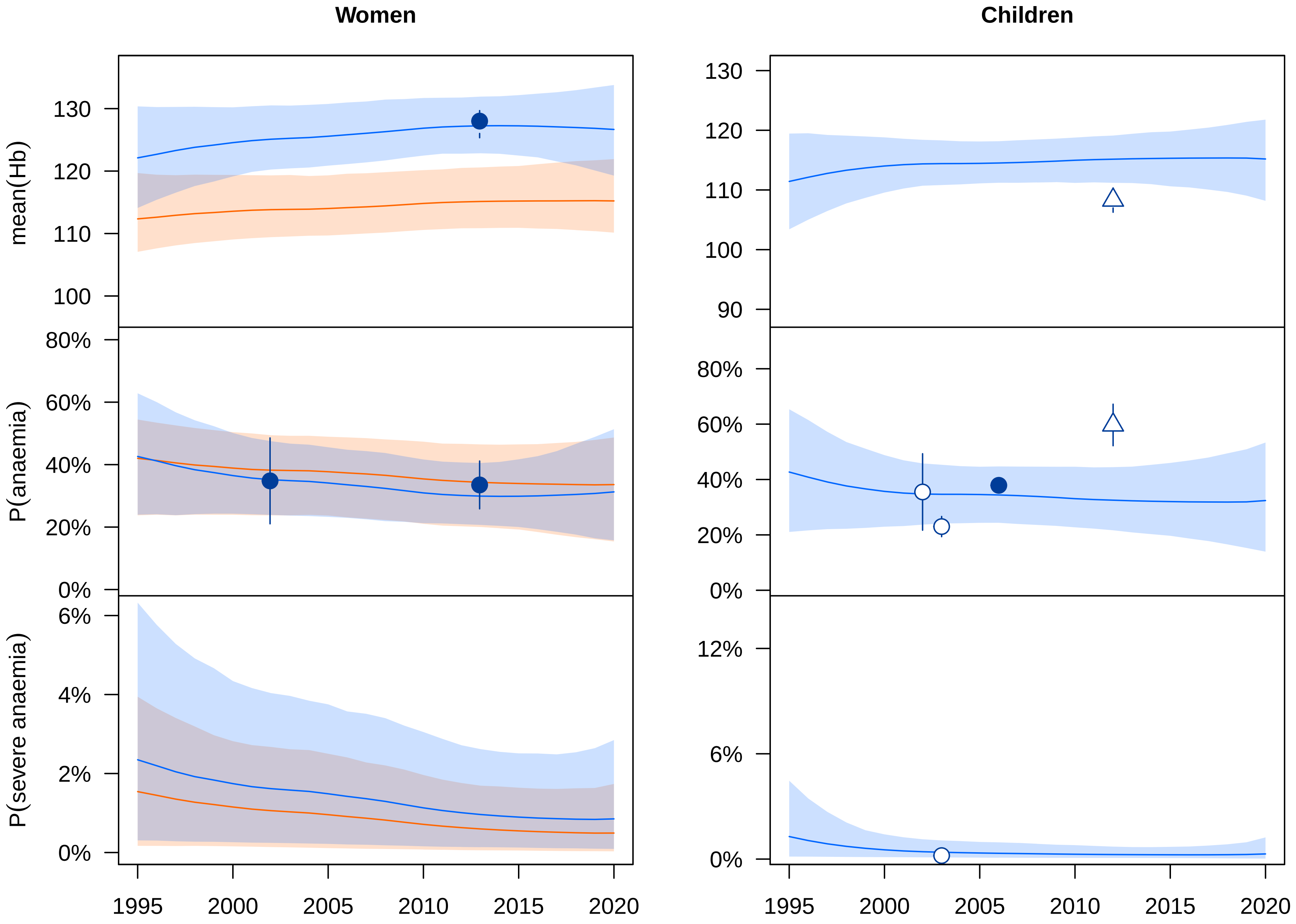

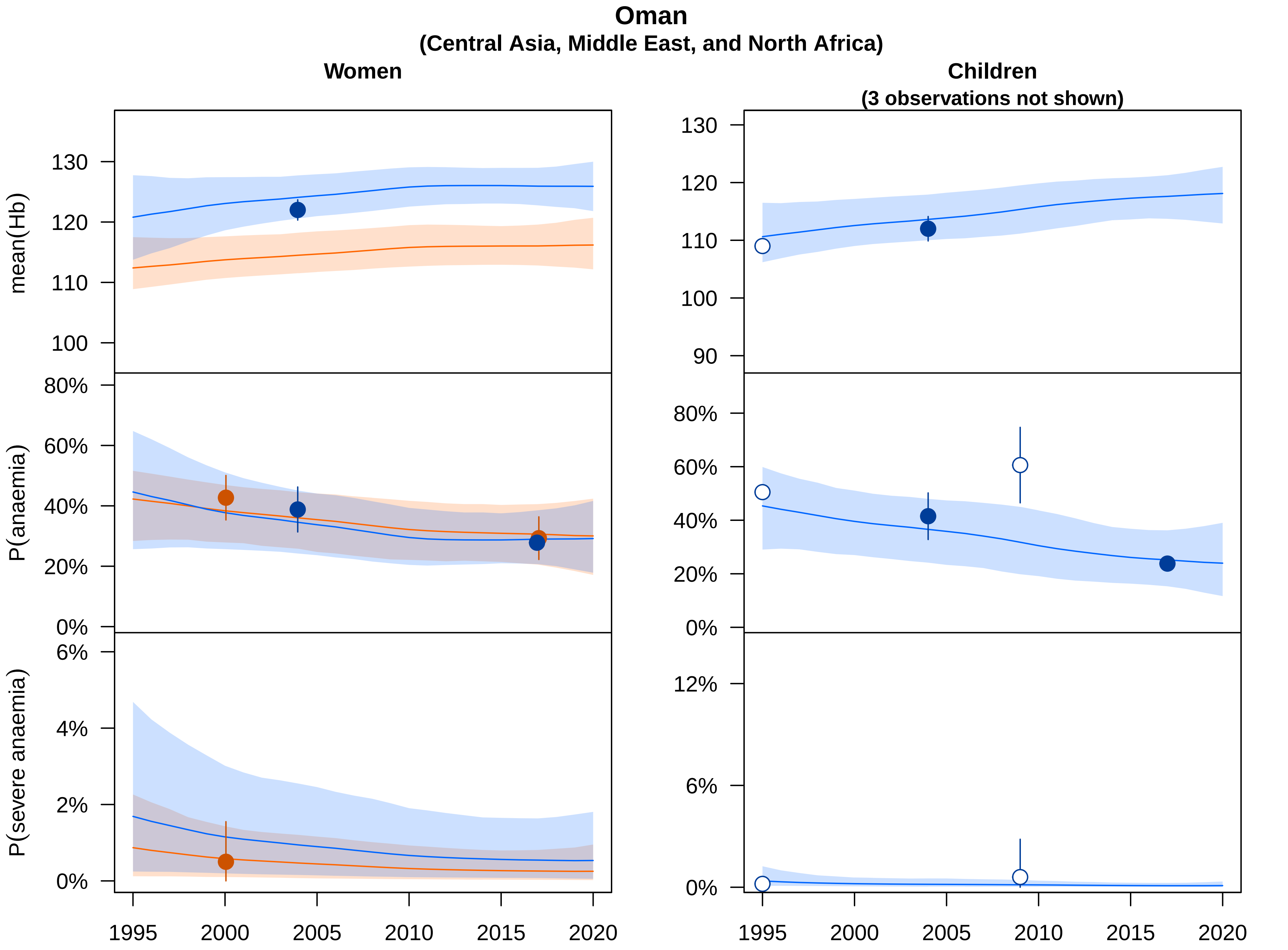
Oman
(Central Asia, Middle East, and North Africa)
Women
Children
(3 observations not shown)
mean(Hb)
P(anaemia)
P(severe anaemia)
130
120
110
100
90
80%
60%
40%
20%
0%
6%
4%
2%
12%
1995
2000
2005
2010
2015
2020

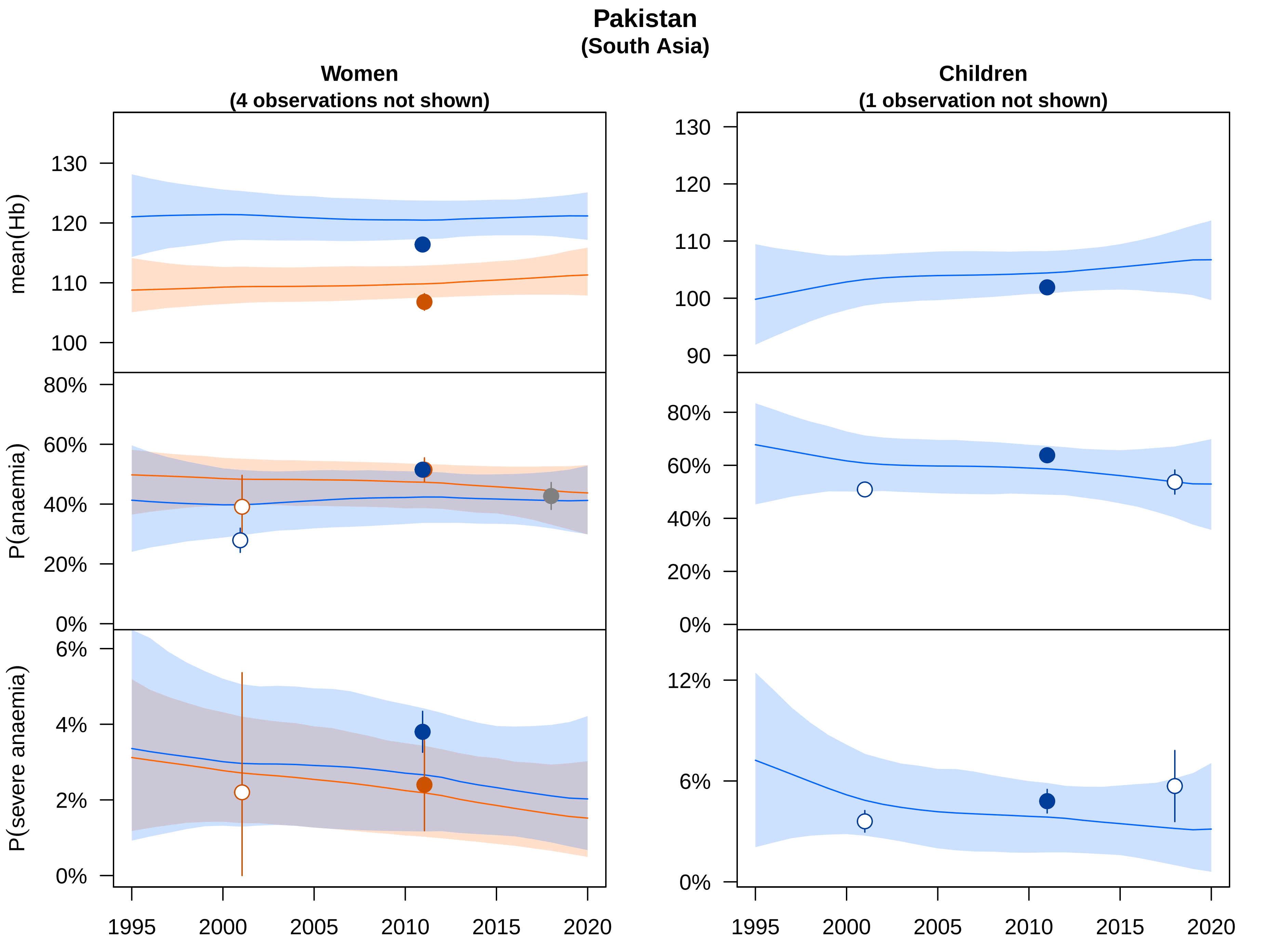

Pakistan
(South Asia)
Women
(4 observations not shown)
Children
(1 observation not shown)
mean(Hb)
P(anaemia)
P(severe anaemia)
130
120
110
100
90
80%
60%
40%
20%
0%
6%
4%
2%
12%
1995
2000
2005
2010
2015
2020

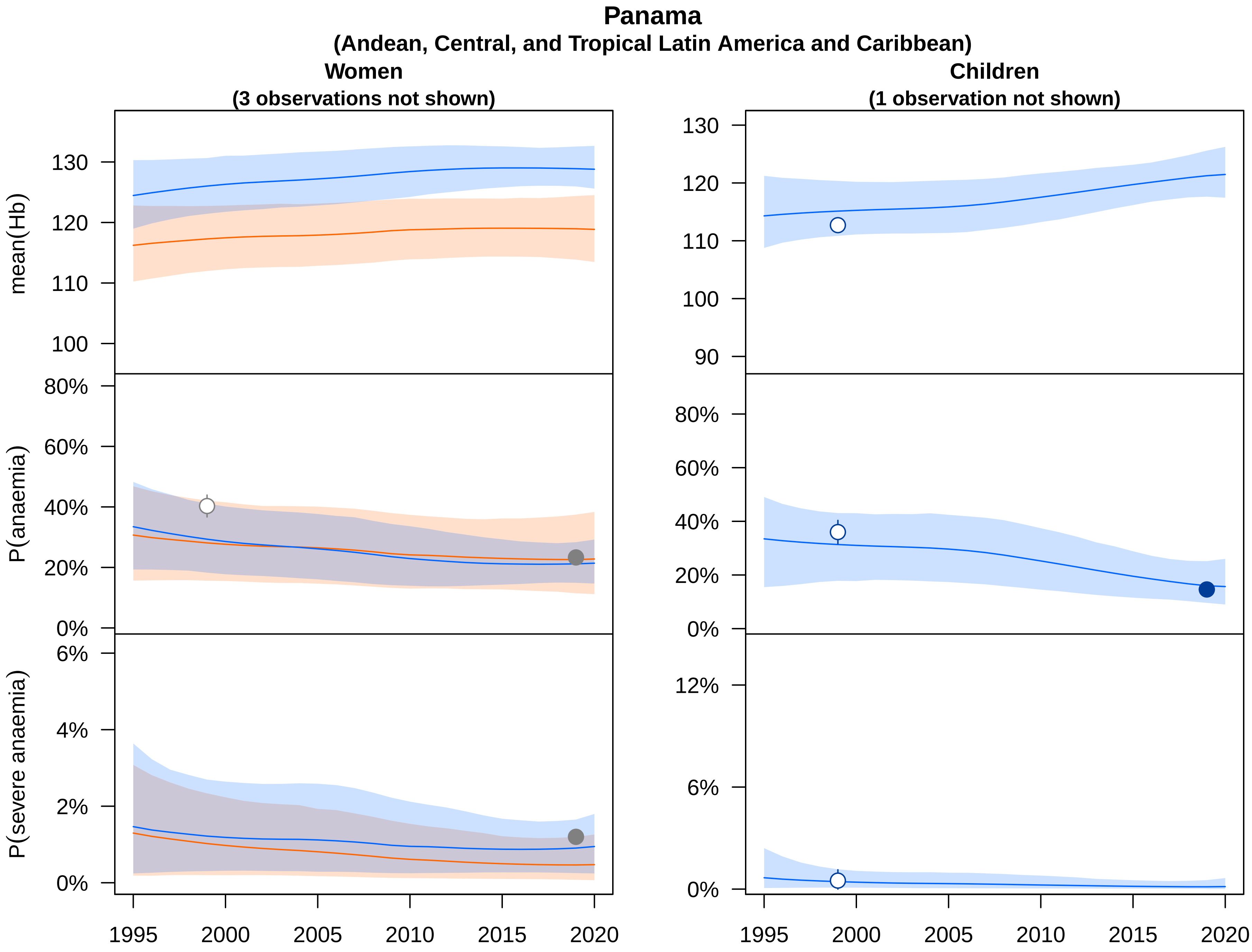
Panama
(Andean, Central, and Tropical Latin America and Caribbean)
Women
(3 observations not shown)
Children
(1 observation not shown)
mean(Hb)
P(anaemia)
P(severe anaemia)
130
120
110
100
90
80%
60%
40%
20%
0%
6%
4%
2%
12%
1995
2000
2005
2010
2015
2020

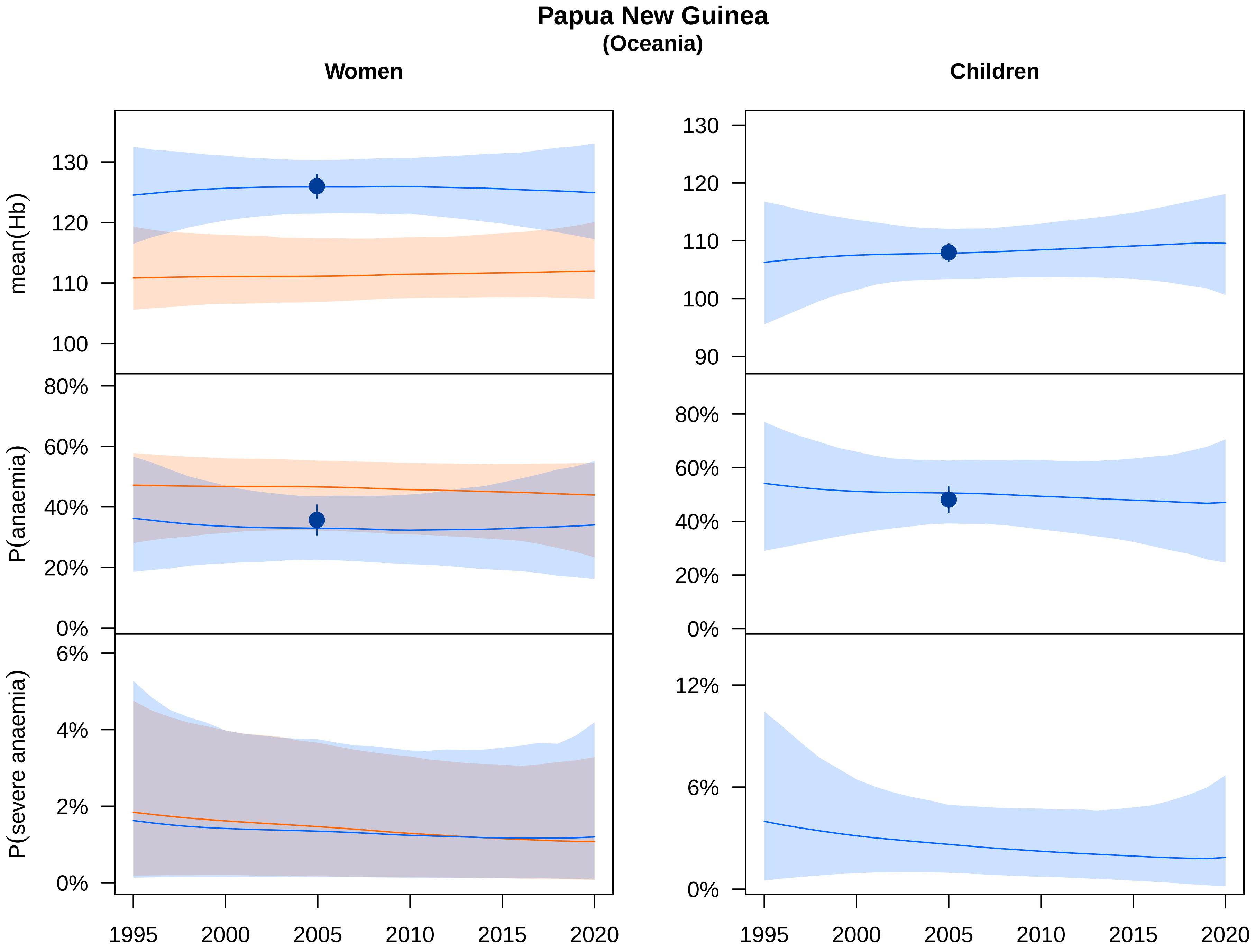
Papua New Guinea
(Oceania)
Women
Children
mean(Hb)
P(anaemia)
P(severe anaemia)
130
120
110
100
90
80%
60%
40%
20%
0%
6%
4%
2%
12%
1995
2000
2005
2010
2015
2020

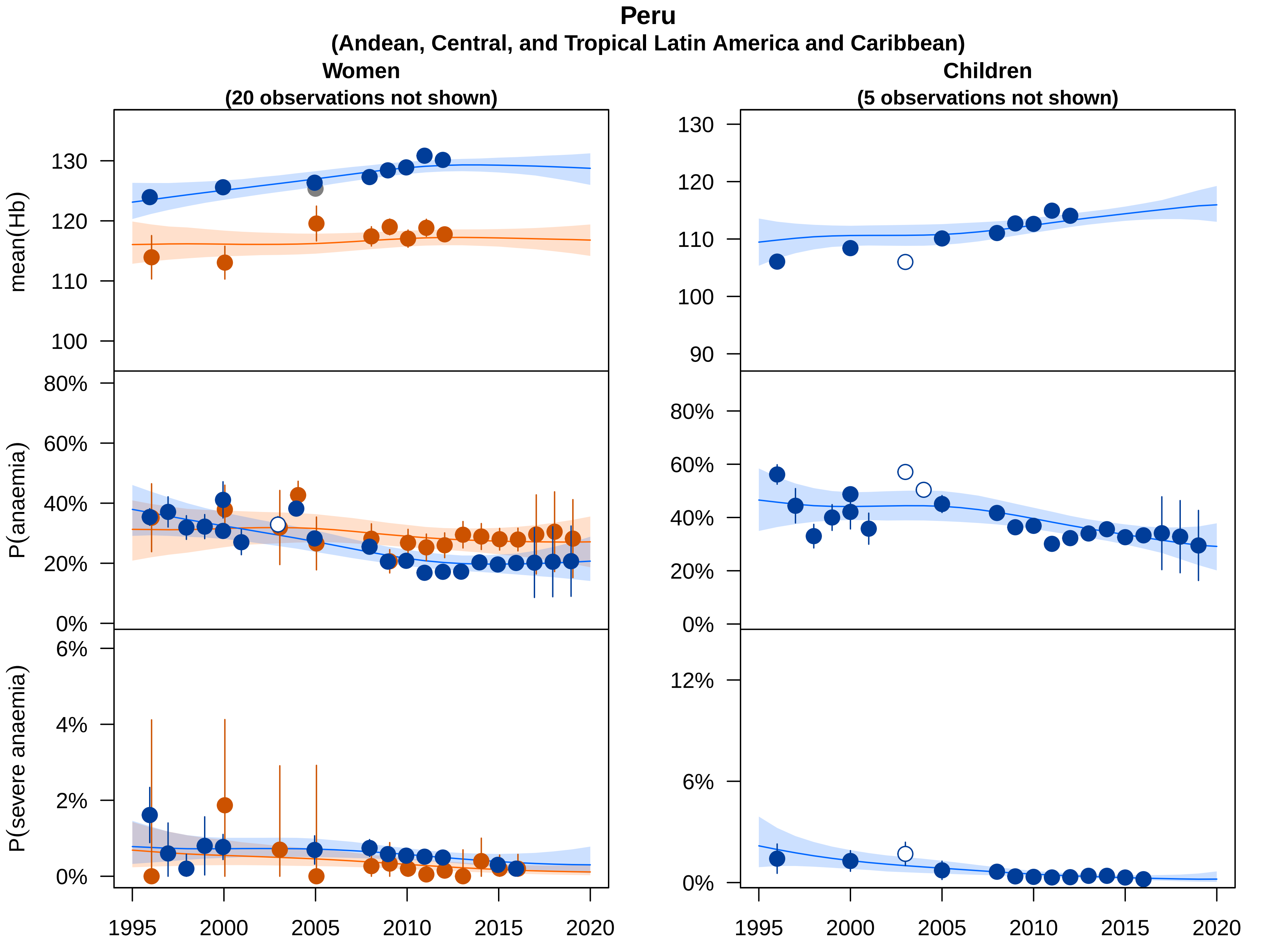

Peru
(Andean, Central, and Tropical Latin America and Caribbean)
Women
(20 observations not shown)
Children
(5 observations not shown)
mean(Hb)
P(anaemia)
P(severe anaemia)
130
120
110
100
90
80%
60%
40%
20%
0%
6%
4%
2%
12%
1995
2000
2005
2010
2015
2020

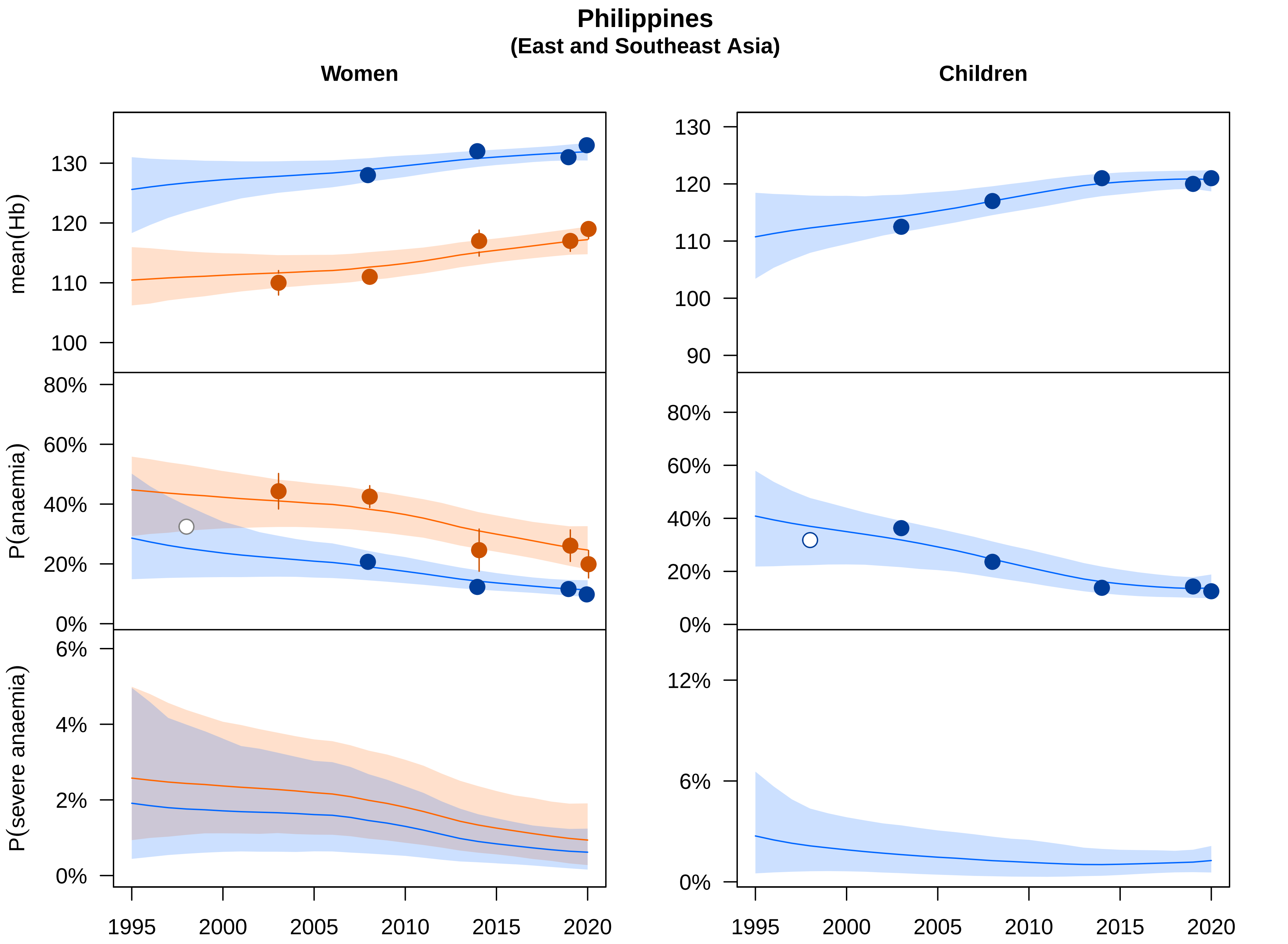

Philippines
(East and Southeast Asia)
Women
Children
mean(Hb)
P(anaemia)
P(severe anaemia)
130
120
110
100
90
80%
60%
40%
20%
0%
6%
4%
2%
12%
1995
2000
2005
2010
2015
2020

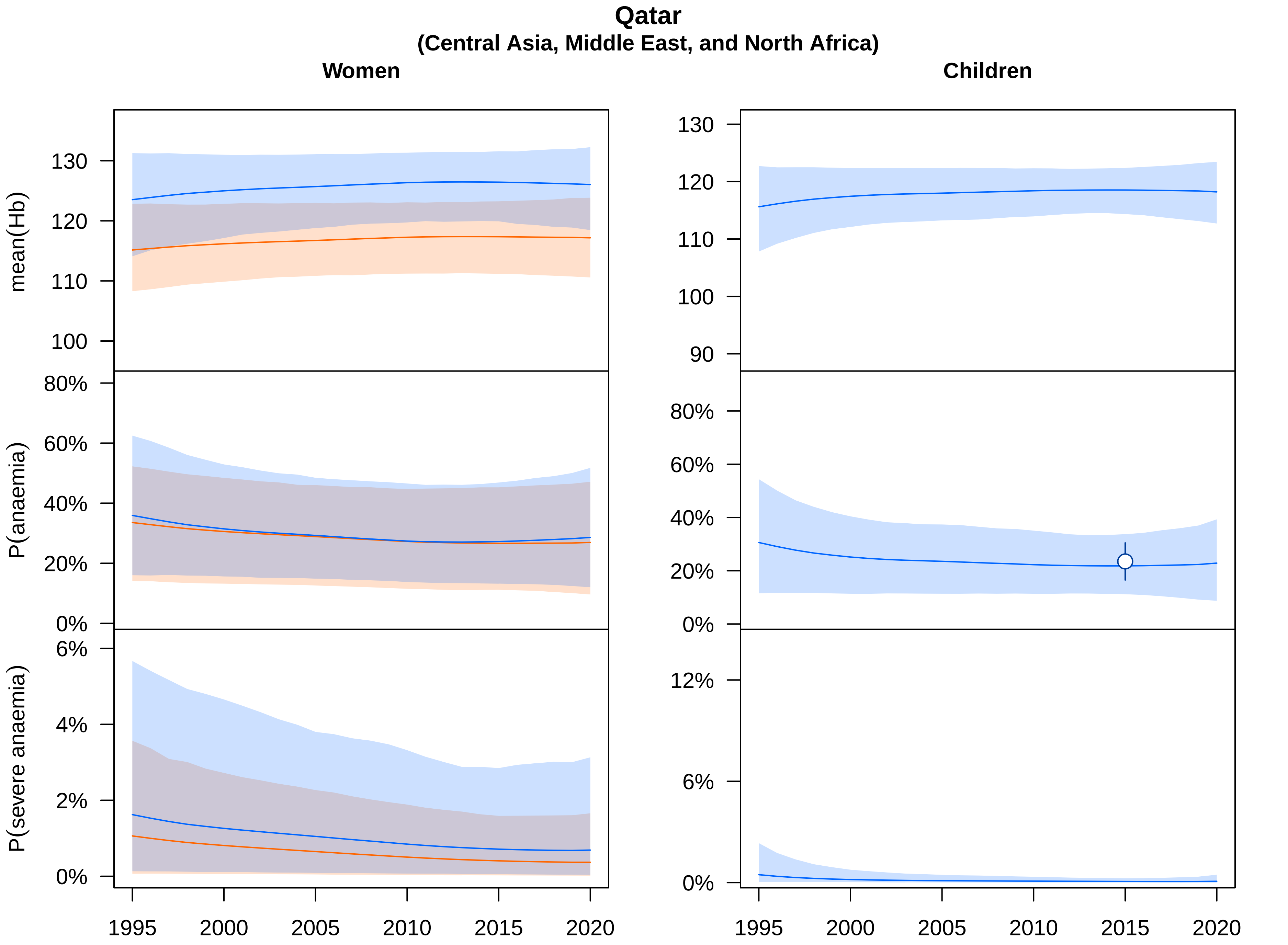
Qatar
(Central Asia, Middle East, and North Africa)
Women
Children
mean(Hb)
P(anaemia)
P(severe anaemia)
130
120
110
100
90
80%
60%
40%
20%
0%
6%
4%
2%
12%
1995
2000
2005
2010
2015
2020

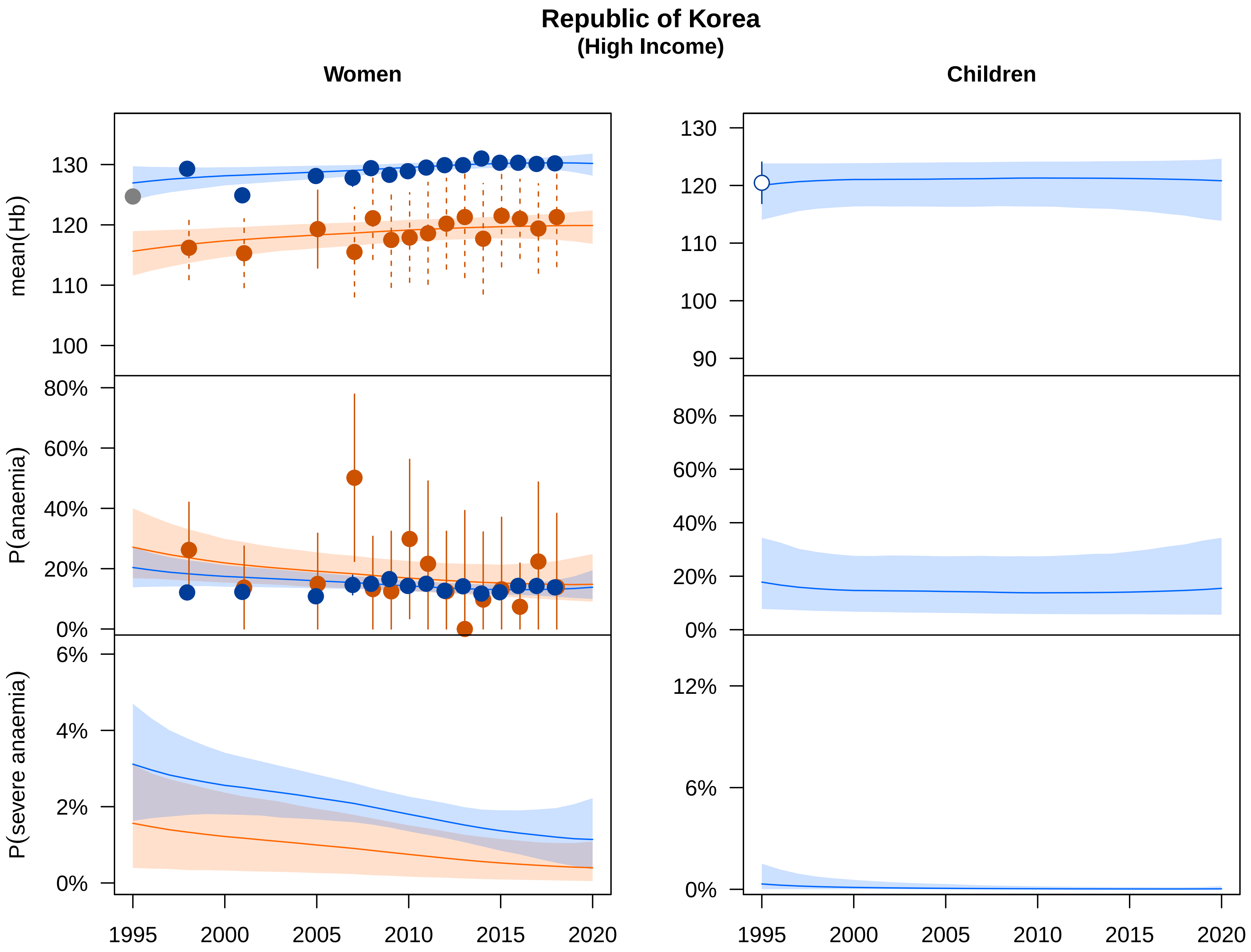
Republic of Korea
(High Income)
Women
Children
mean(Hb)
P(anaemia)
P(severe anaemia)
130
120
110
100
90
80%
60%
40%
20%
0%
6%
4%
2%
12%
1995
2000
2005
2010
2015
2020

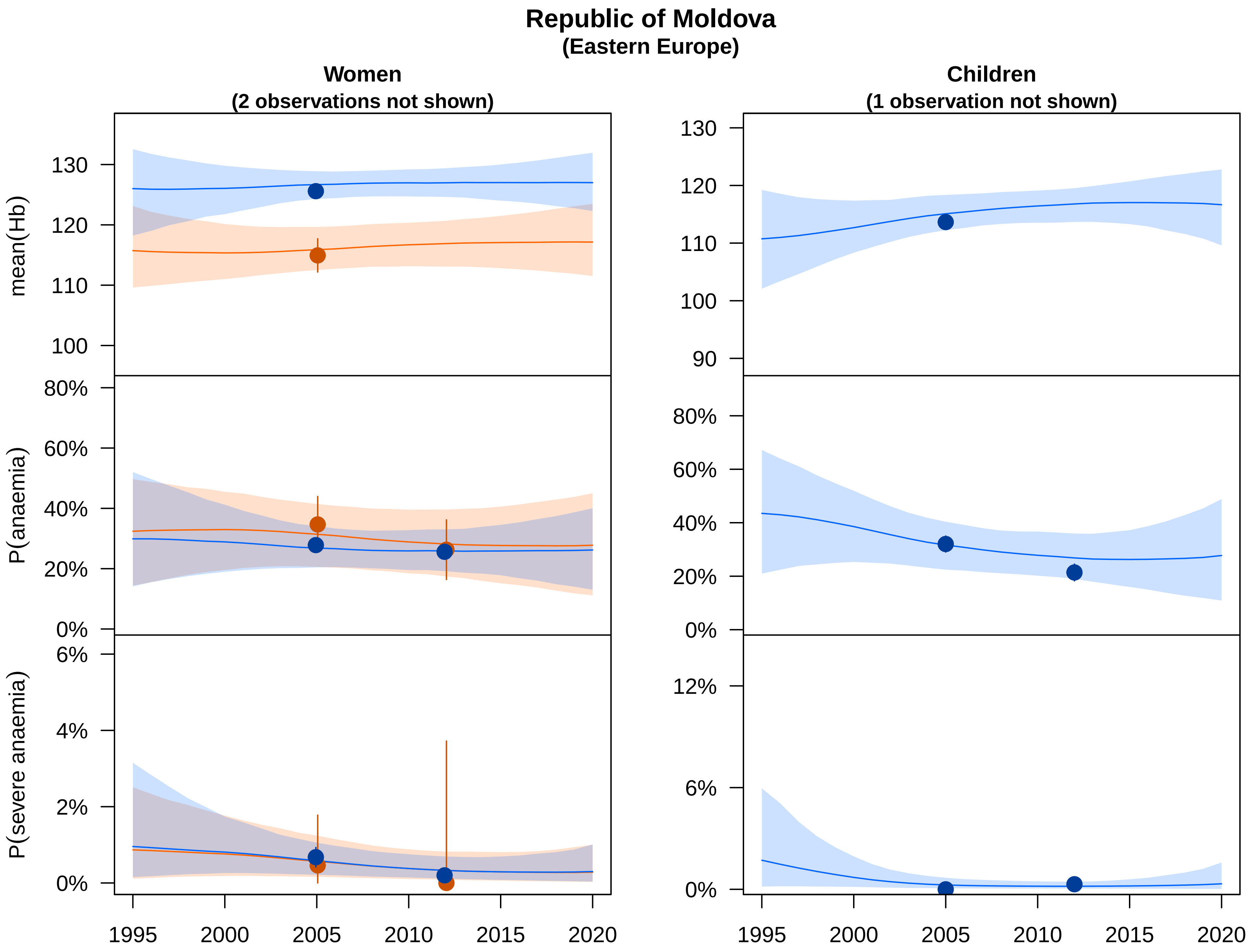

Republic of Moldova
(Eastern Europe)
Women
(2 observations not shown)
Children
(1 observation not shown)
mean(Hb)
P(anaemia)
P(severe anaemia)
130
120
110
100
90
80%
60%
40%
20%
0%
6%
4%
2%
12%
1995
2000
2005
2010
2015
2020

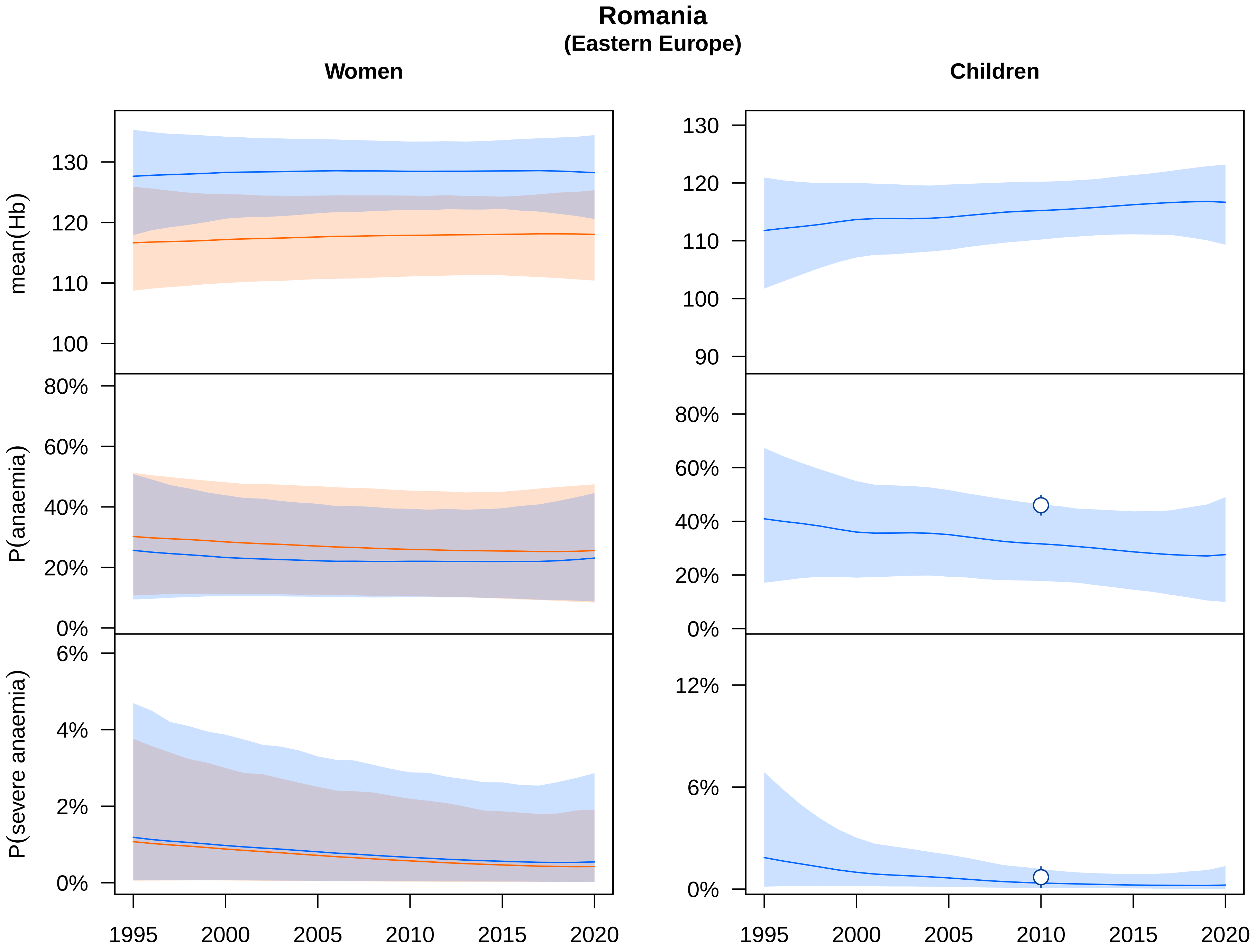

Romania
(Eastern Europe)
Women
Children
mean(Hb)
P(anaemia)
P(severe anaemia)
130
120
110
100
90
80%
60%
40%
20%
0%
6%
4%
2%
12%
1995
2000
2005
2010
2015
2020

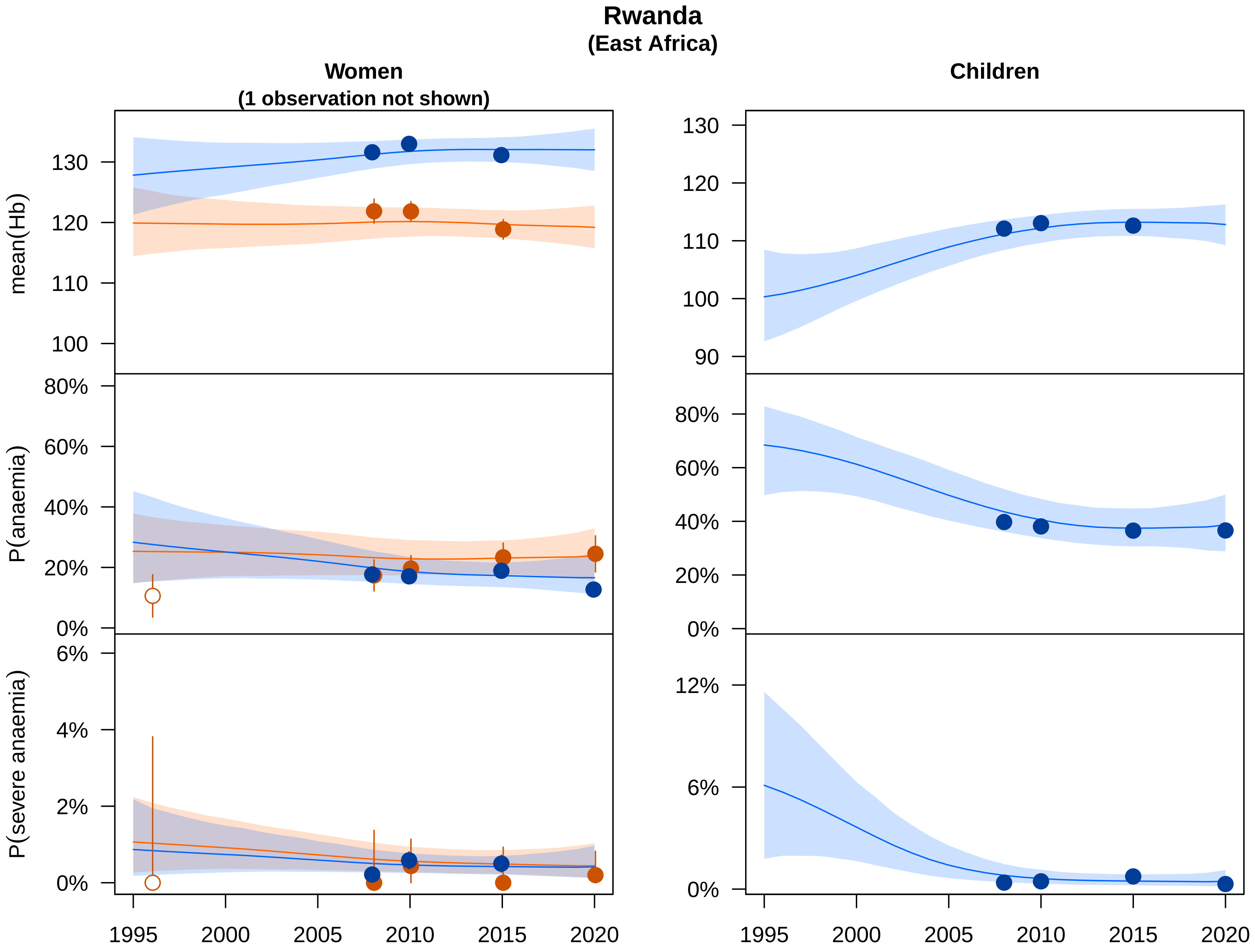

Rwanda
(East Africa)
Women
(1 observation not shown)
Children
mean(Hb)
P(anaemia)
P(severe anaemia)
130
120
110
100
90
80%
60%
40%
20%
0%
6%
4%
2%
12%
1995
2000
2005
2010
2015
2020

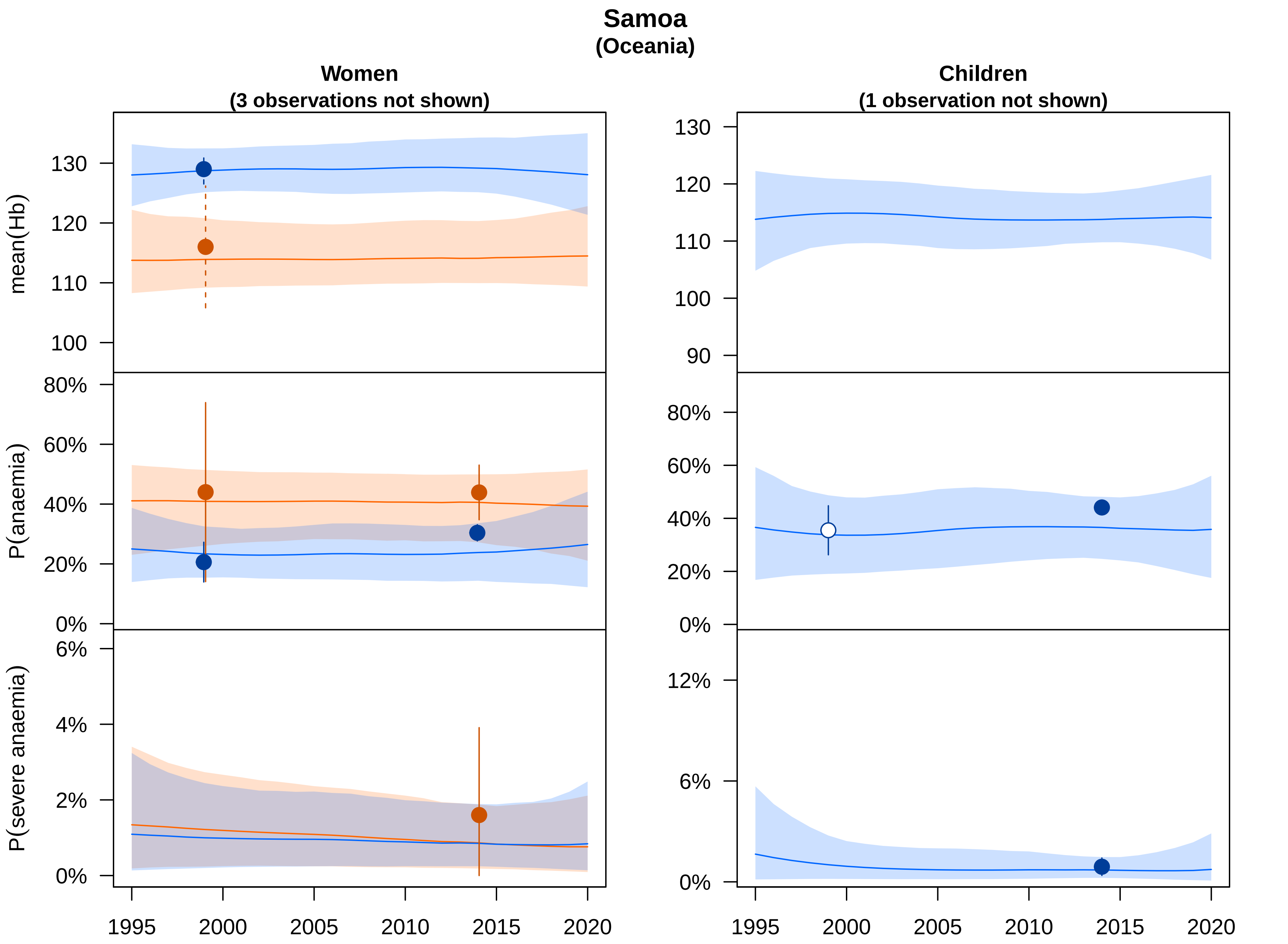
Samoa
(Oceania)
Women
(3 observations not shown)
Children
(1 observation not shown)
mean(Hb)
P(anaemia)
P(severe anaemia)
130
120
110
100
90
80%
60%
40%
20%
0%
6%
4%
2%
12%
1995
2000
2005
2010
2015
2020

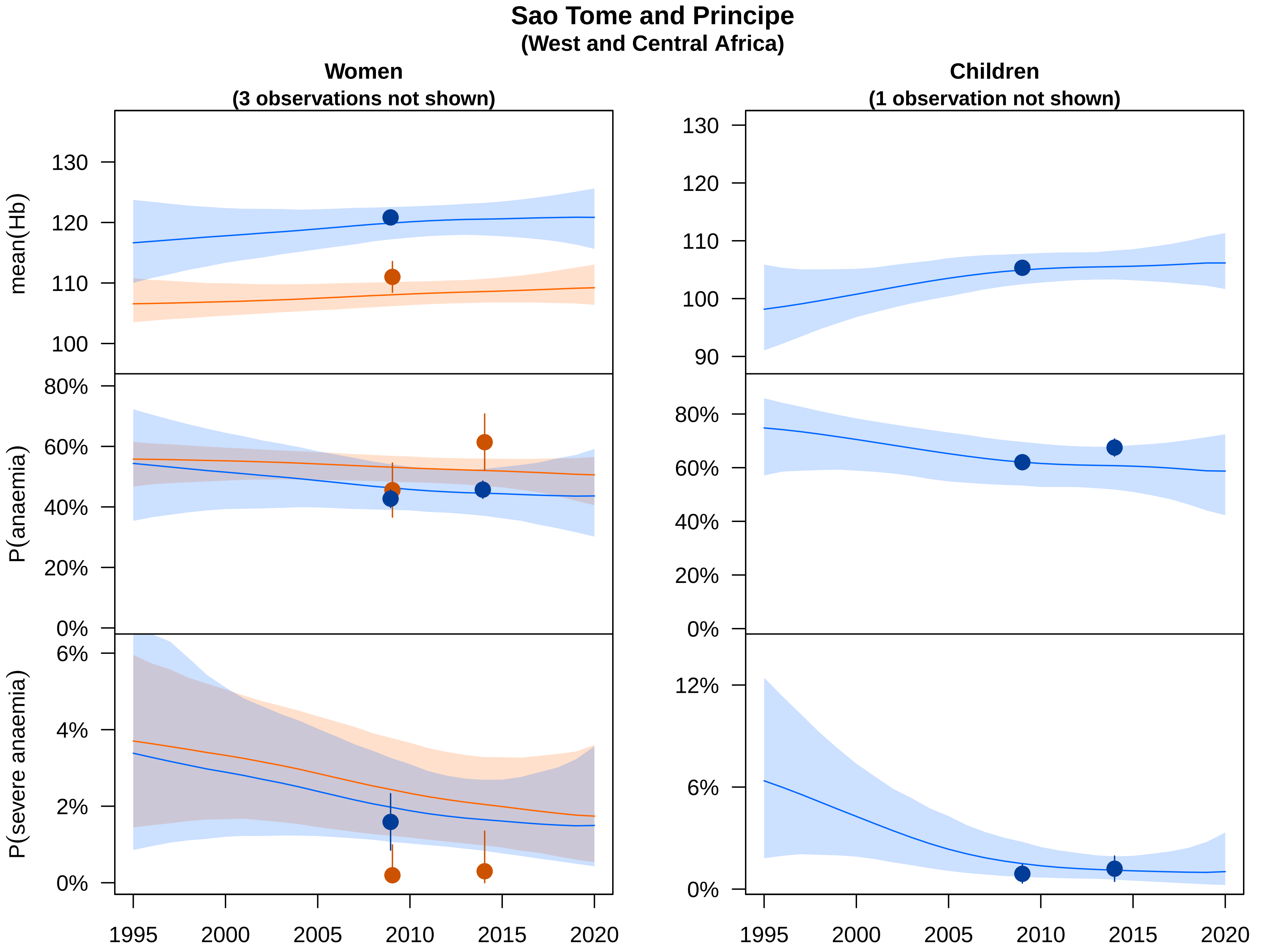

Sao Tome and Principe
(West and Central Africa)
Women
(3 observations not shown)
Children
(1 observation not shown)
mean(Hb)
P(anaemia)
P(severe anaemia)
130
120
110
100
90
80%
60%
40%
20%
0%
6%
4%
2%
12%
1995
2000
2005
2010
2015
2020

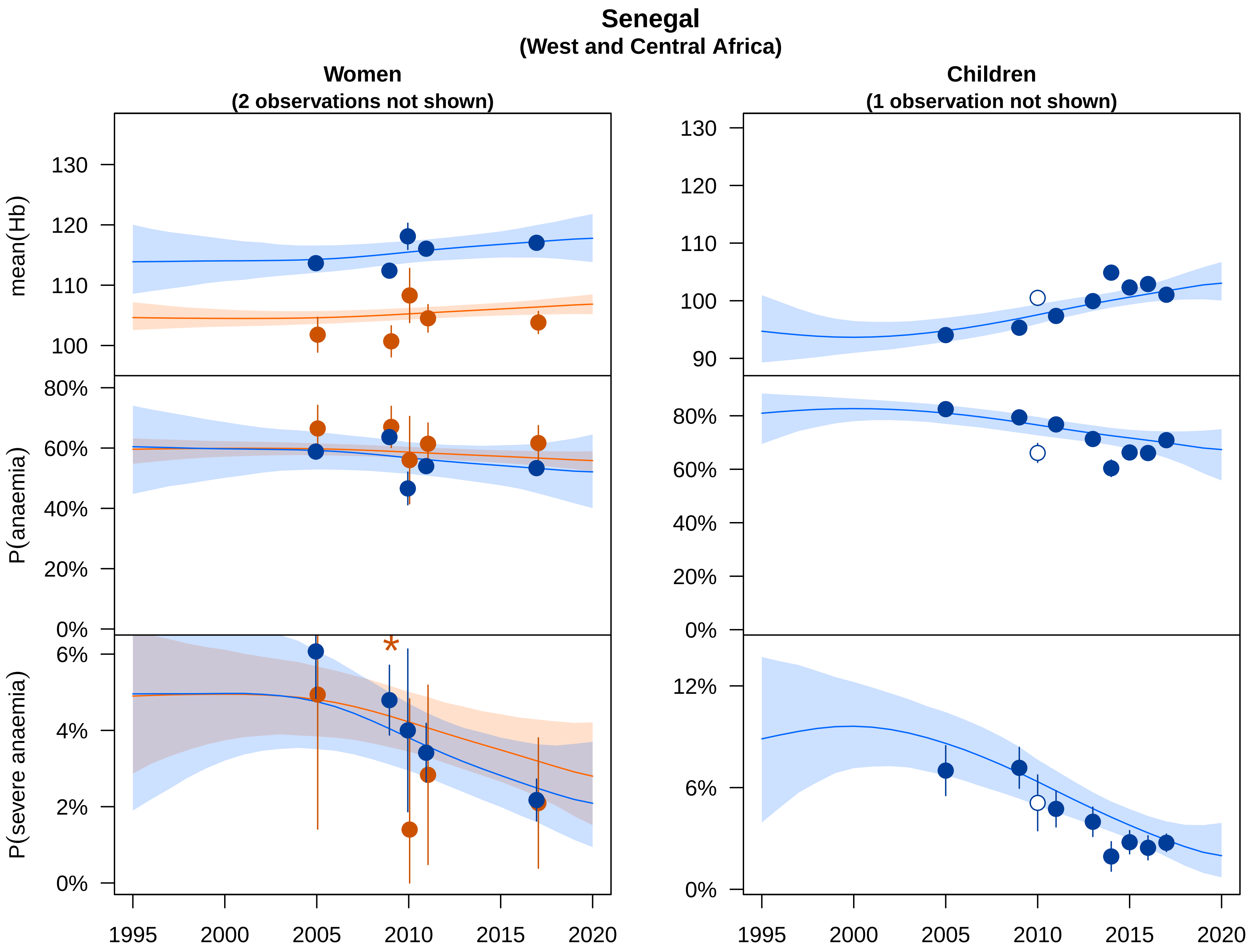
Senegal
(West and Central Africa)
Women
(2 observations not shown)
Children
(1 observation not shown)
mean(Hb)
P(anaemia)
P(severe anaemia)
130
120
110
100
90
80%
60%
40%
20%
0%
12%
6%
4%
2%
1995
2000
2005
2010
2015
2020
*

Serbia
(Eastern Europe)

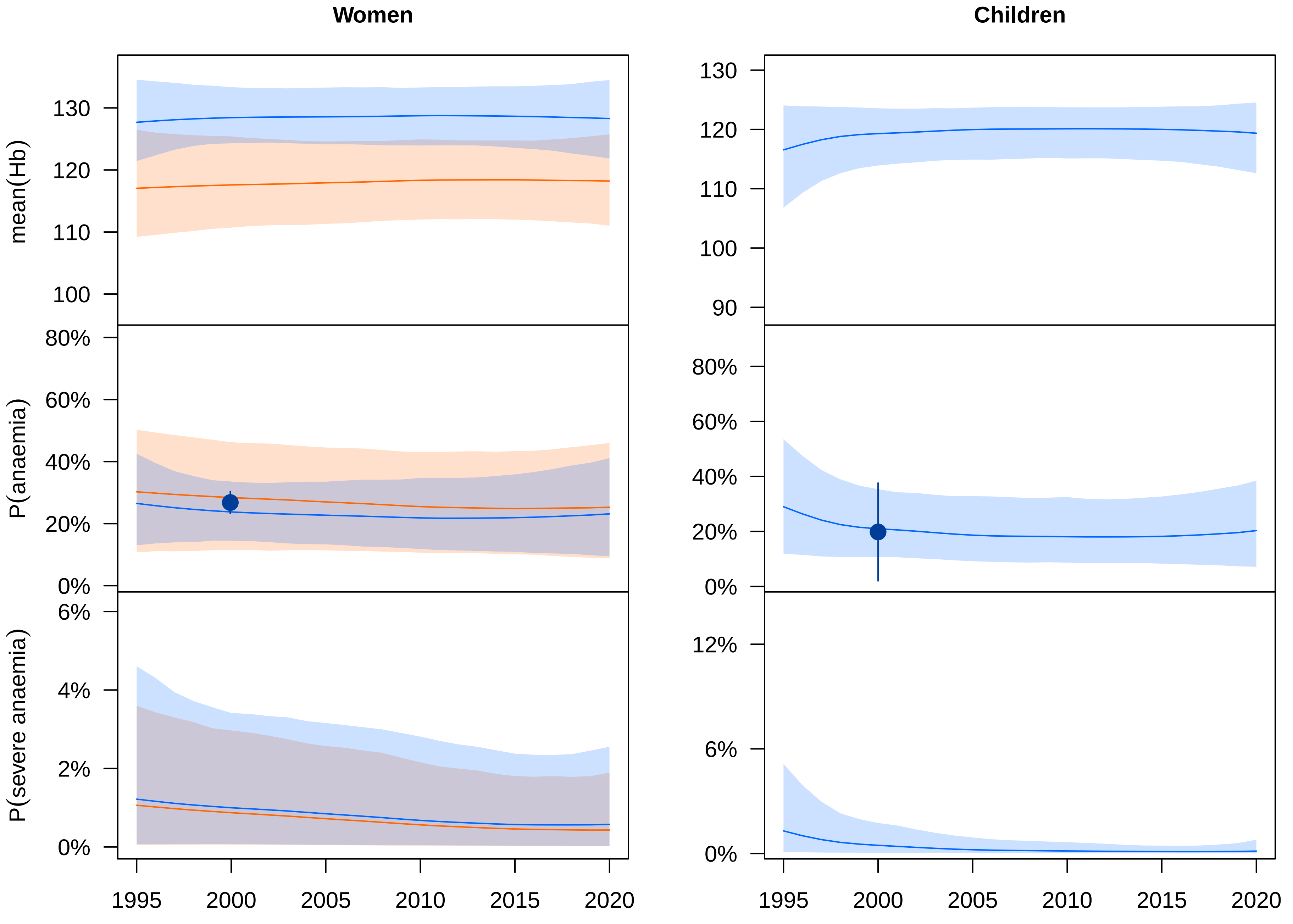

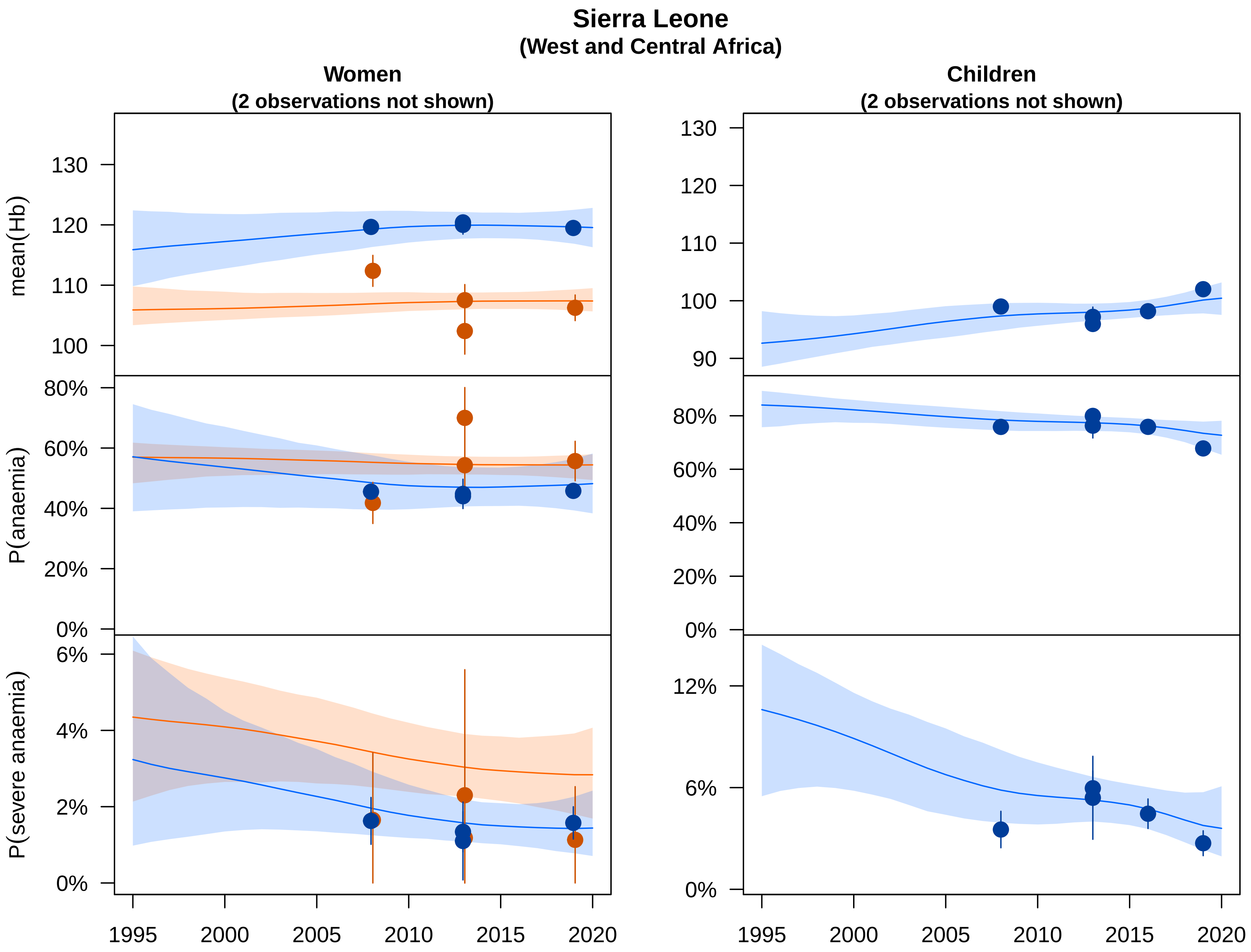

Sierra Leone
(West and Central Africa)
Women
(2 observations not shown)
Children
(2 observations not shown)
mean(Hb)
P(anaemia)
P(severe anaemia)
130
120
110
100
90
80%
60%
40%
20%
0%
6%
4%
2%
12%
1995
2000
2005
2010
2015
2020

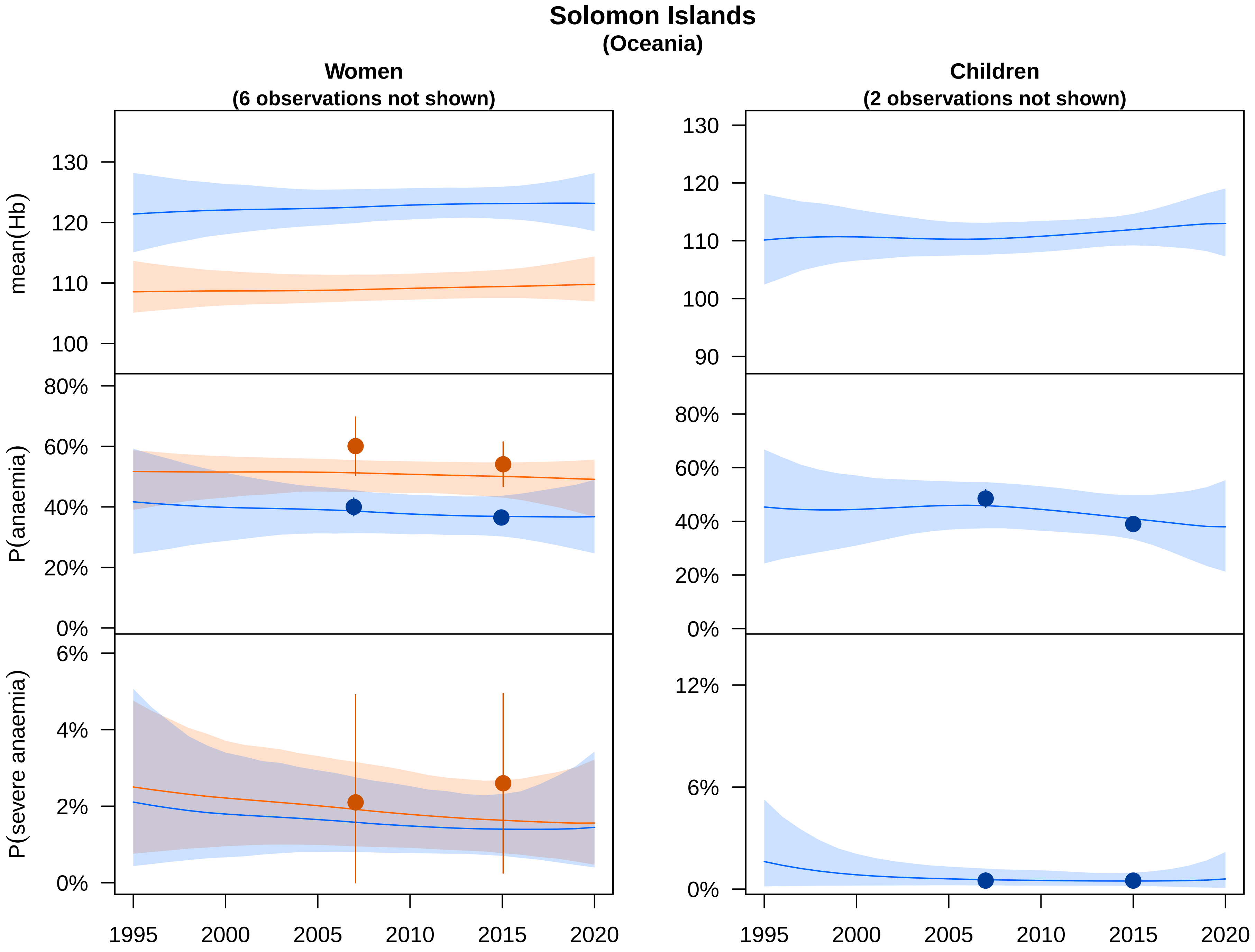

Solomon Islands
(Oceania)
Women
(6 observations not shown)
Children
(2 observations not shown)
mean(Hb)
P(anaemia)
P(severe anaemia)
130
120
110
100
90
80%
60%
40%
20%
0%
6%
4%
2%
12%
1995
2000
2005
2010
2015
2020

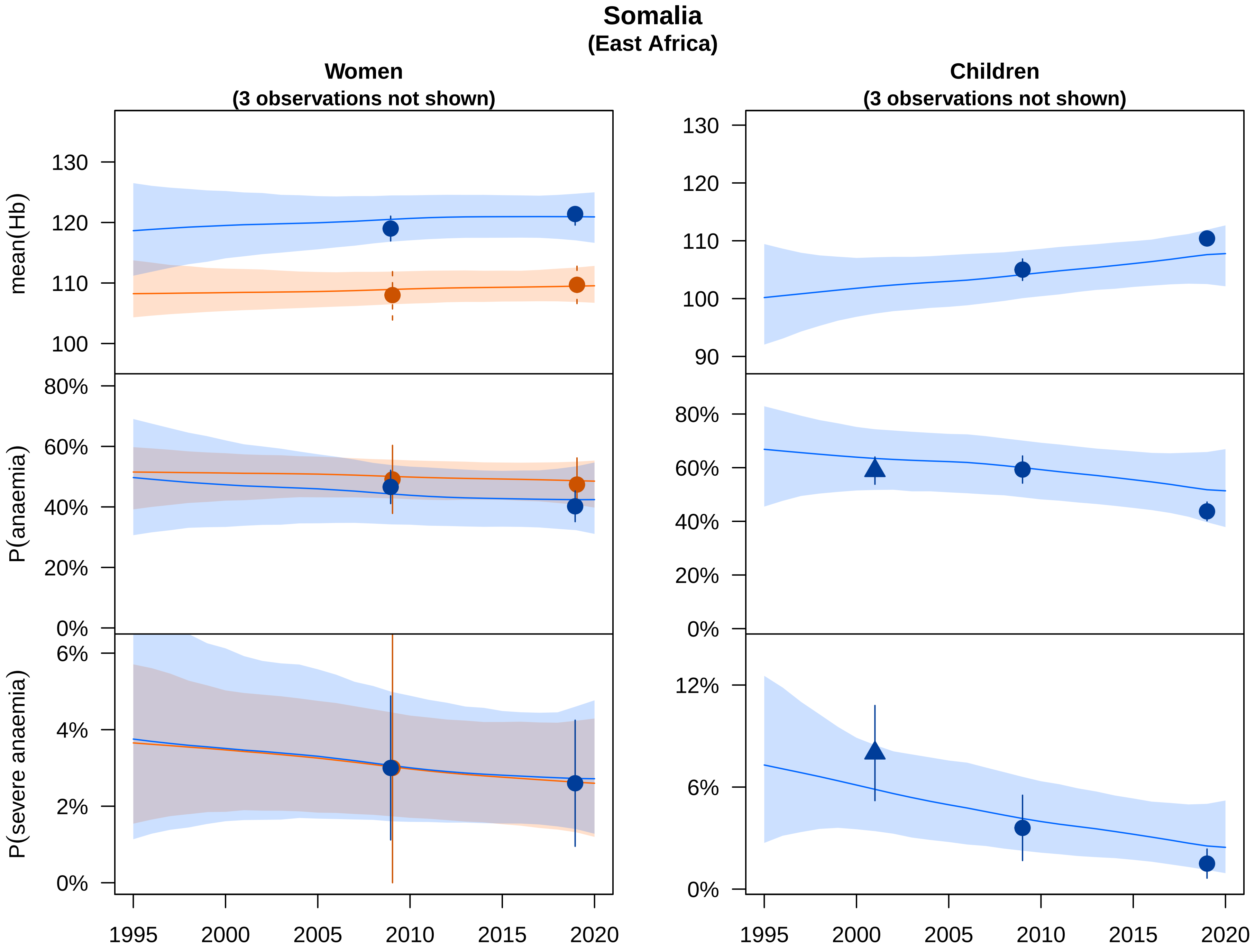

Somalia
(East Africa)
Women
(3 observations not shown)
Children
(3 observations not shown)
mean(Hb)
P(anaemia)
P(severe anaemia)
130
120
110
100
90
80%
60%
40%
20%
0%
6%
4%
2%
12%
1995
2000
2005
2010
2015
2020

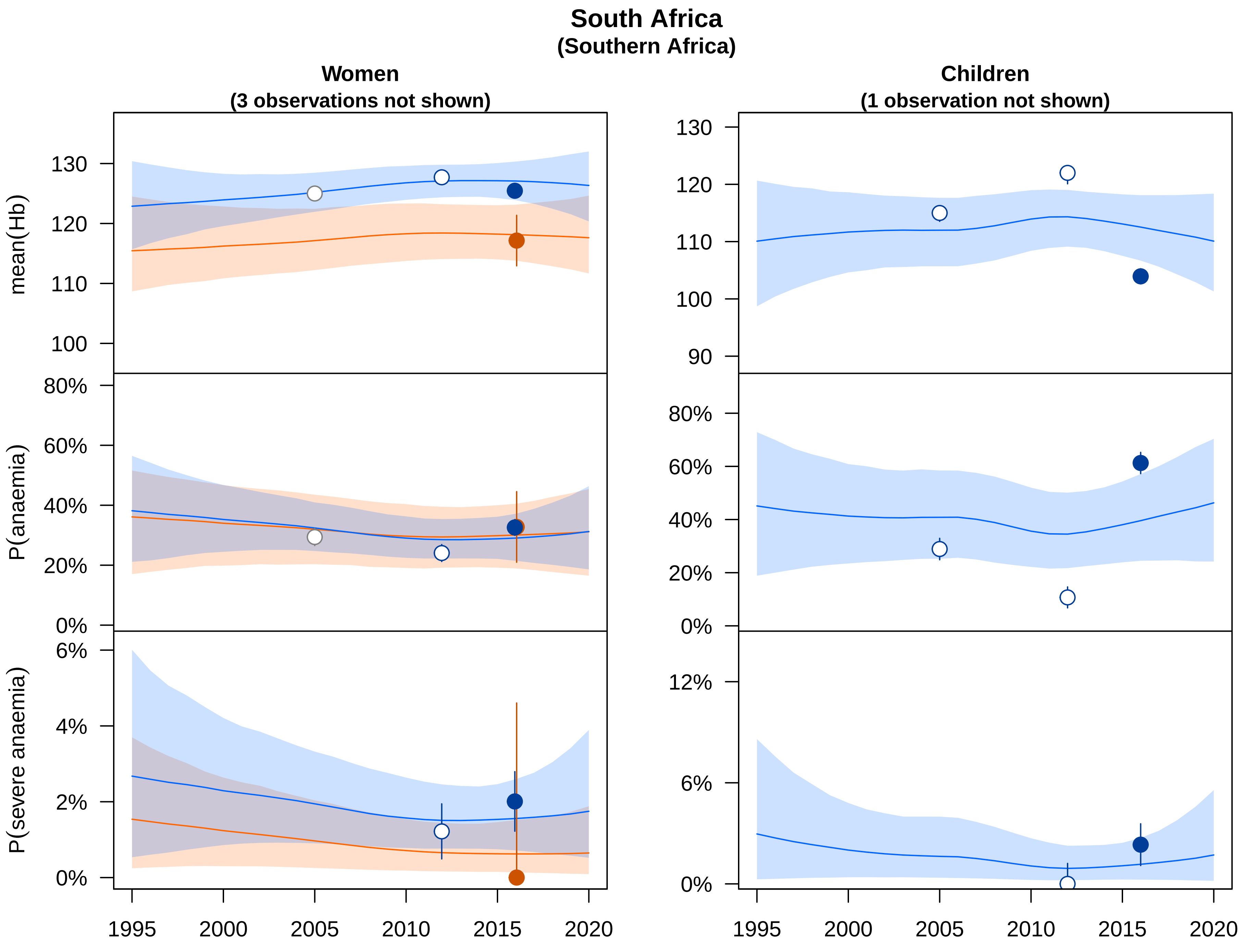
South Africa
(Southern Africa)
Women
(3 observations not shown)
Children
(1 observation not shown)
mean(Hb)
P(anaemia)
P(severe anaemia)
130
120
110
100
90
80%
60%
40%
20%
0%
6%
4%
2%
12%
1995
2000
2005
2010
2015
2020

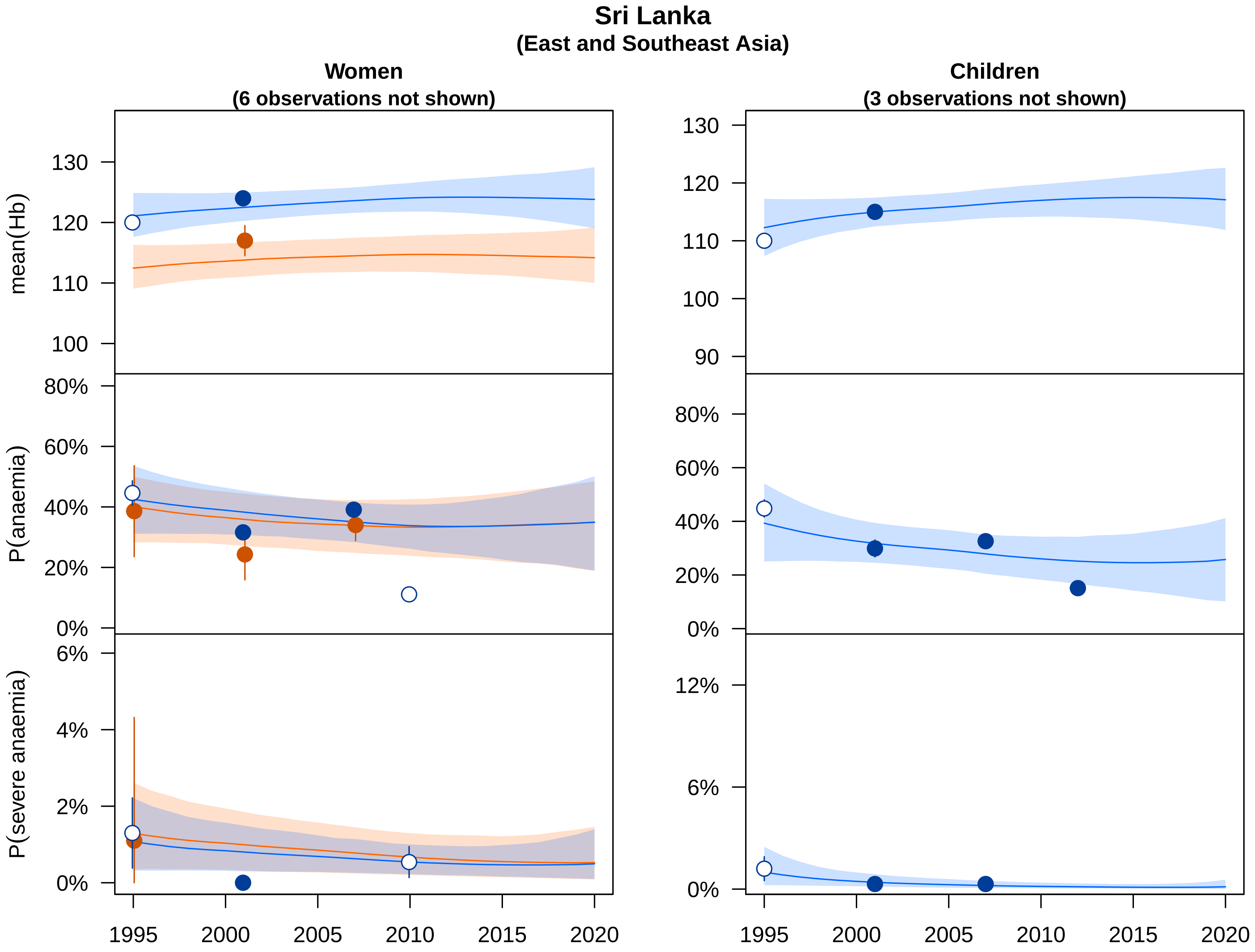
Sri Lanka
(East and Southeast Asia)
Women
(6 observations not shown)
Children
(3 observations not shown)
mean(Hb)
P(anaemia)
P(severe anaemia)
130
120
110
100
90
80%
60%
40%
20%
0%
6%
4%
2%
12%
1995
2000
2005
2010
2015
2020

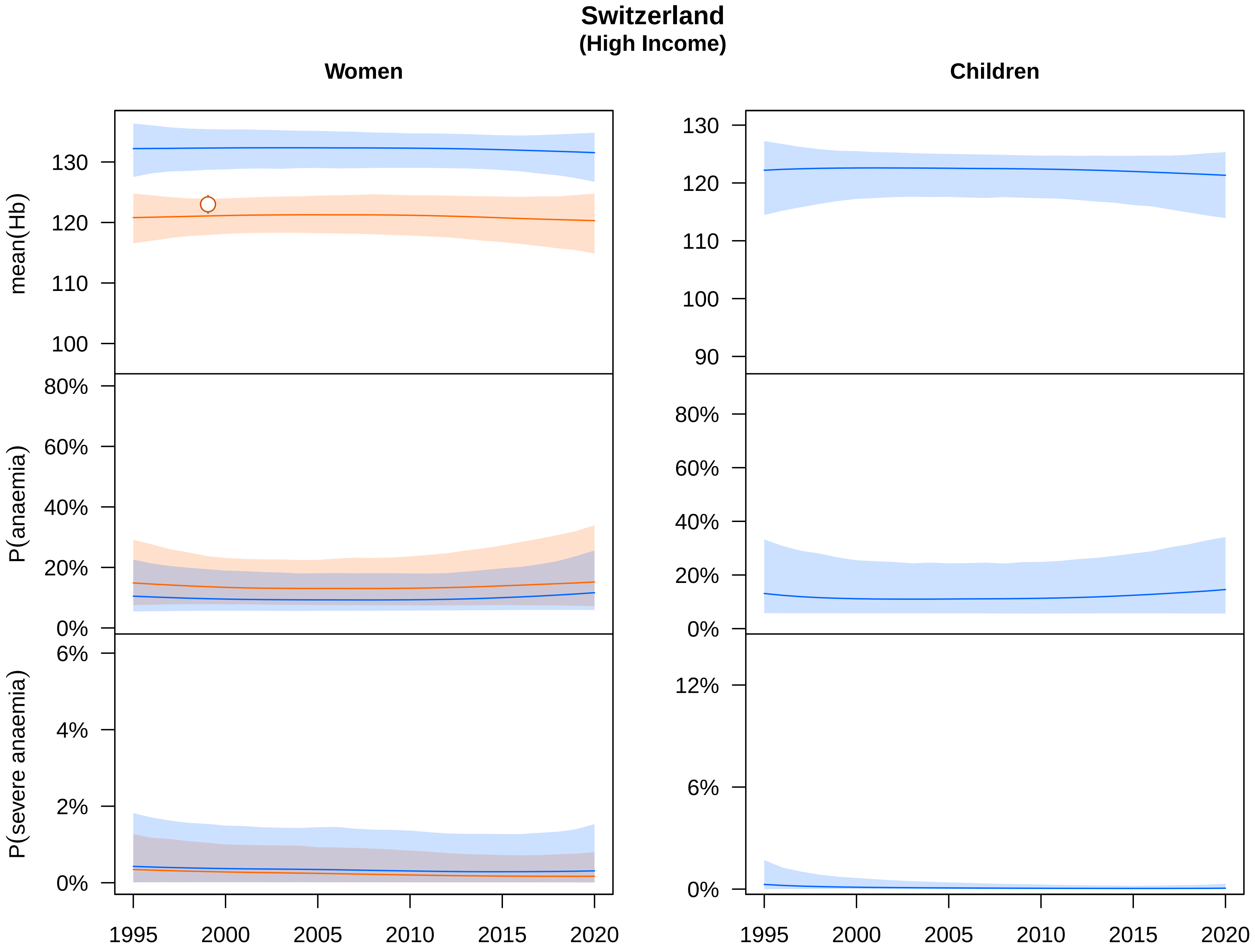

Switzerland
(High Income)
Women
Children
mean(Hb)
P(anaemia)
P(severe anaemia)
130
120
110
100
90
80%
60%
40%
20%
0%
6%
4%
2%
12%
1995
2000
2005
2010
2015
2020

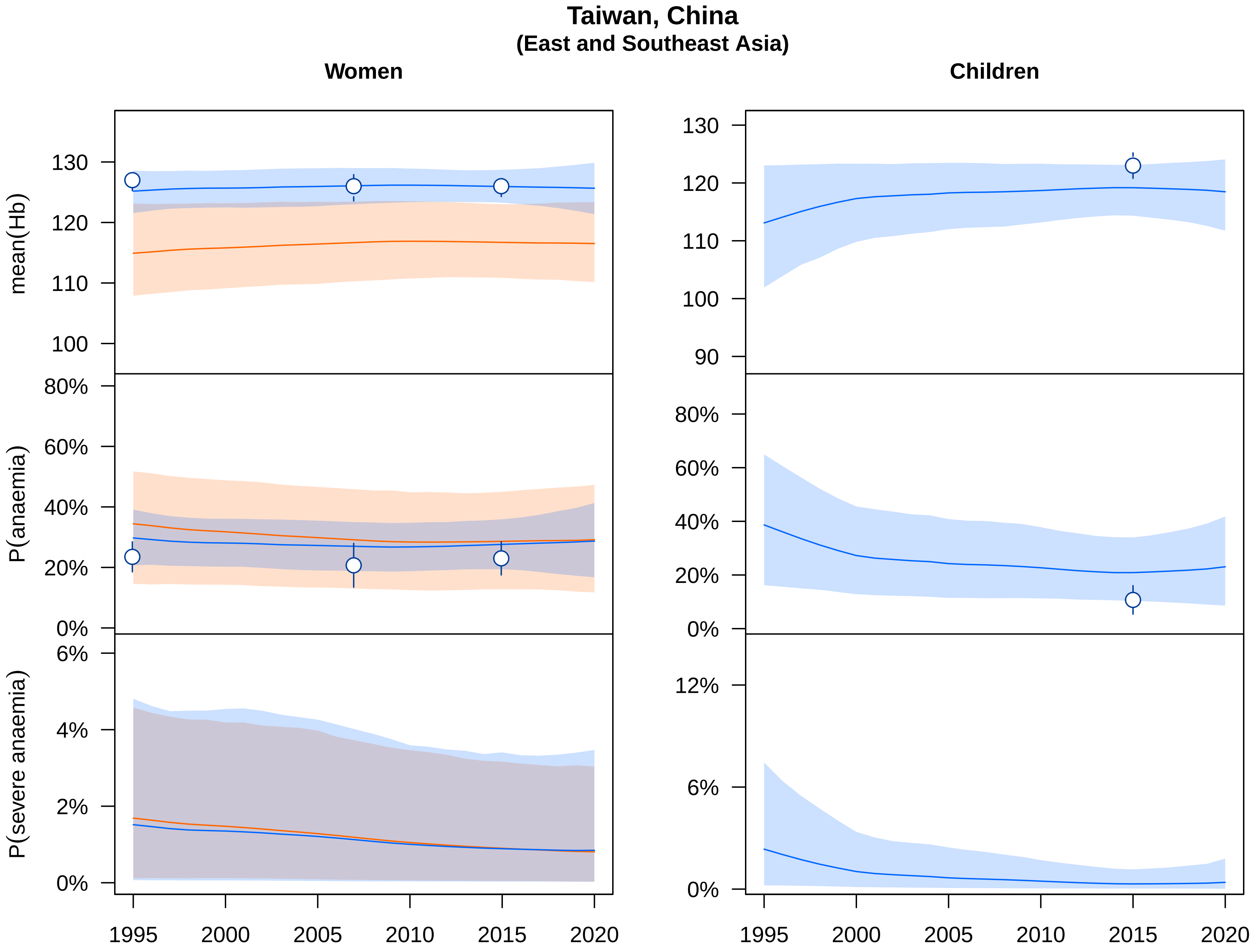

Taiwan, China
(East and Southeast Asia)
Women
Children
mean(Hb)
P(anaemia)
P(severe anaemia)
130
120
110
100
90
80%
60%
40%
20%
0%
6%
4%
2%
12%
1995
2000
2005
2010
2015
2020

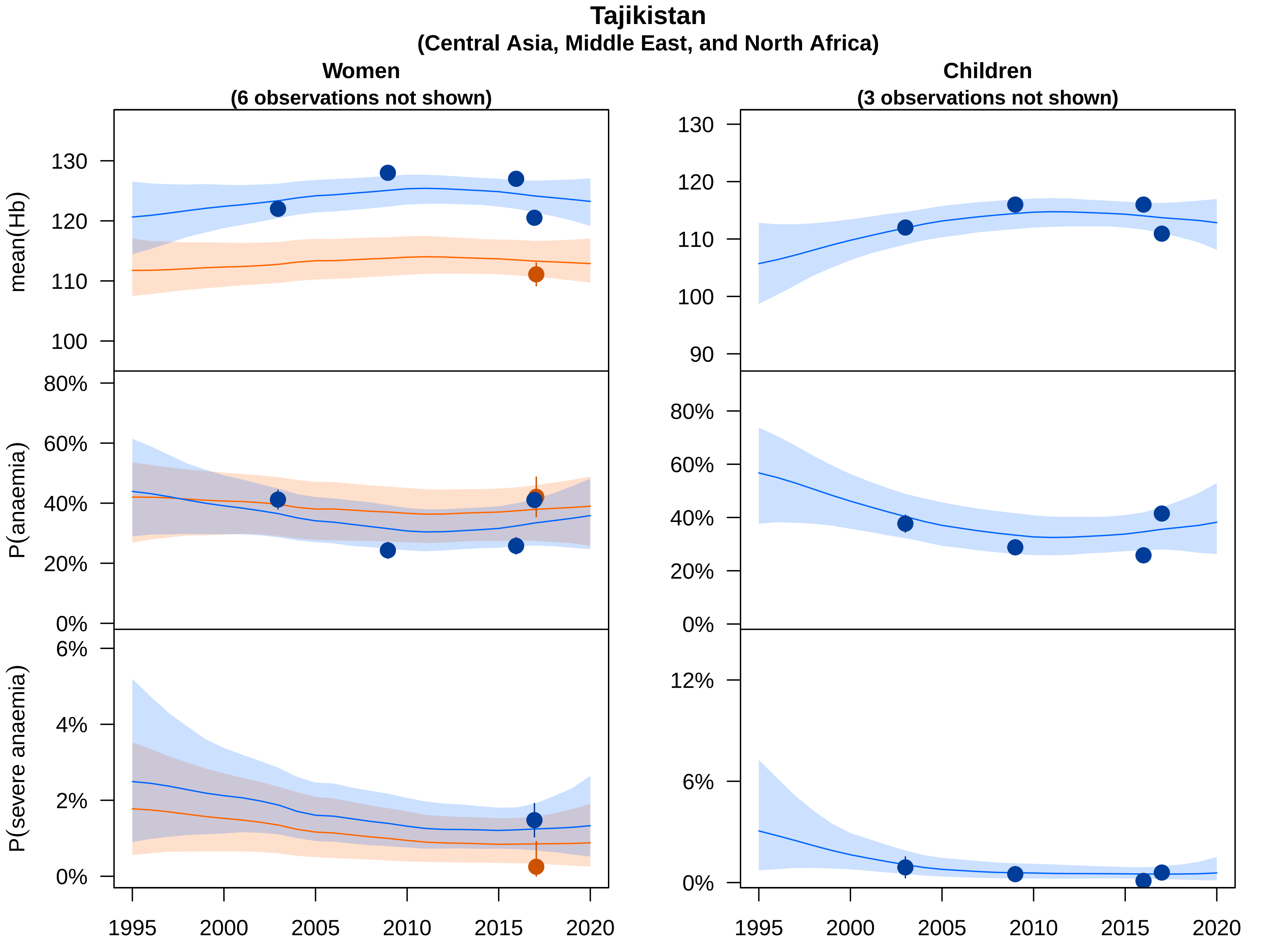

Tajikistan
(Central Asia, Middle East, and North Africa)
Women
(6 observations not shown)
Children
(3 observations not shown)
mean(Hb)
P(anaemia)
P(severe anaemia)
130
120
110
100
90
80%
60%
40%
20%
0%
6%
4%
2%
12%
1995
2000
2005
2010
2015
2020

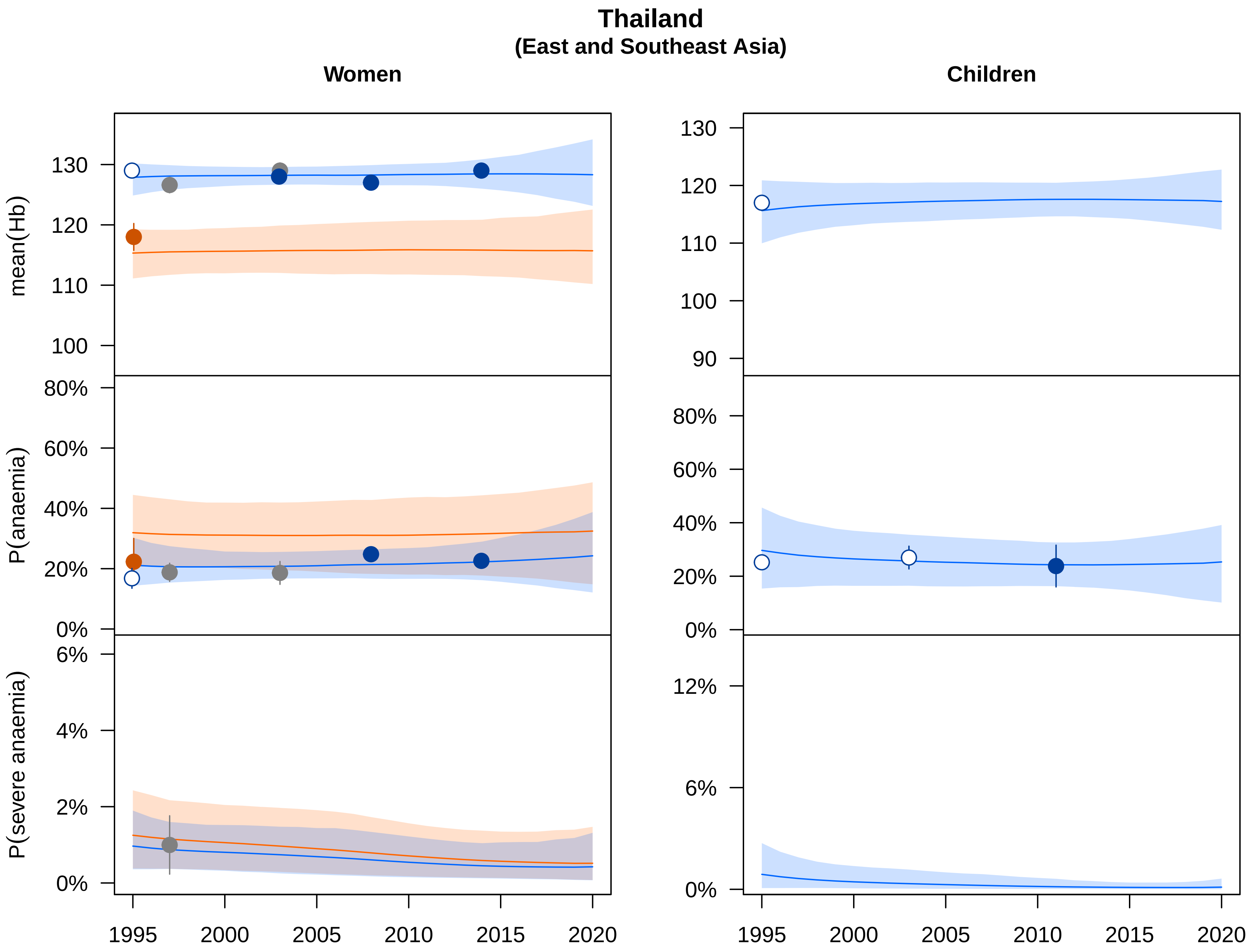
Thailand
(East and Southeast Asia)
Women
Children
mean(Hb)
P(anaemia)
P(severe anaemia)
130
120
110
100
90
80%
60%
40%
20%
0%
6%
4%
2%
12%
1995
2000
2005
2010
2015
2020

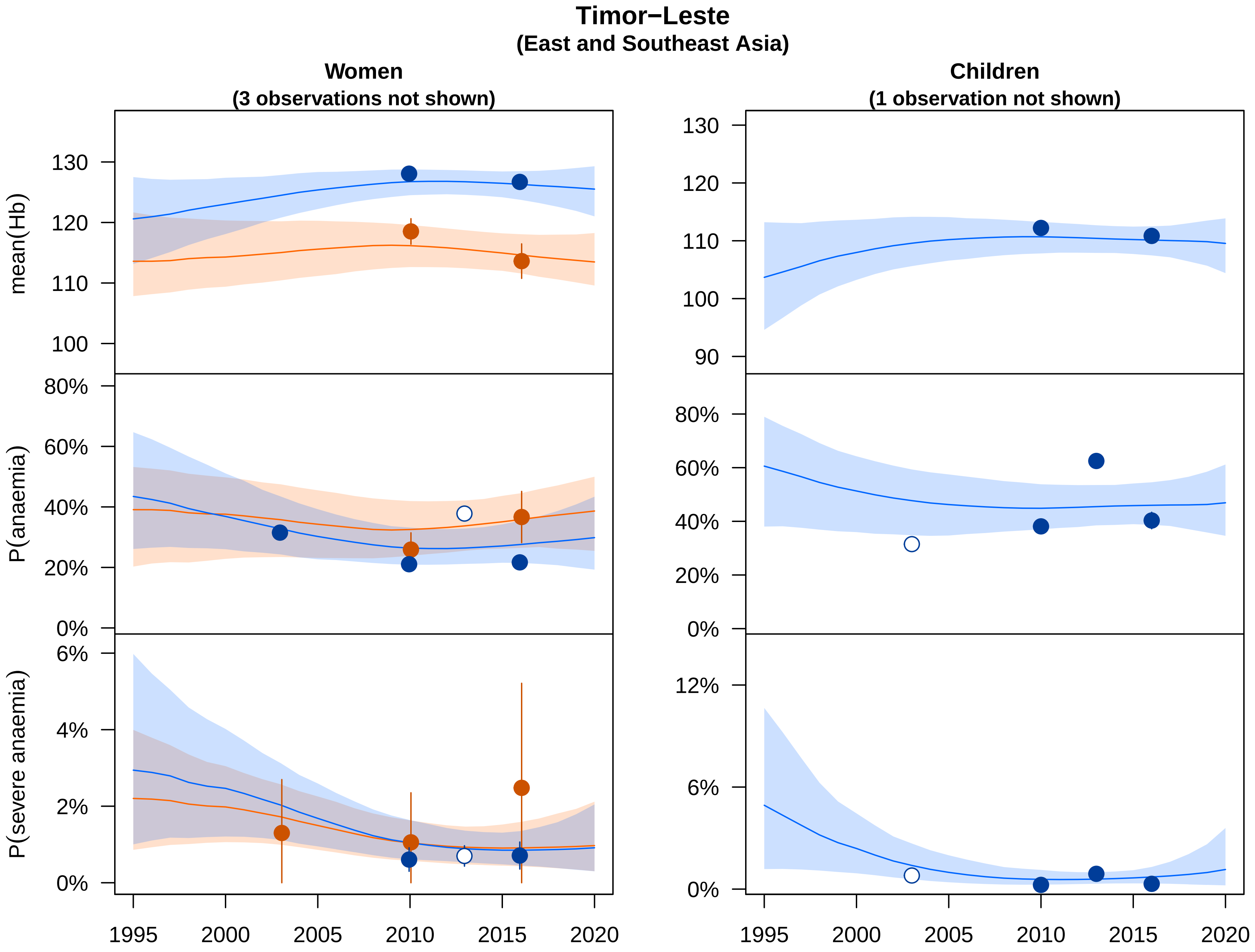

Timor−Leste
(East and Southeast Asia)
Women
(3 observations not shown)
Children
(1 observation not shown)
mean(Hb)
P(anaemia)
P(severe anaemia)
130
120
110
100
90
80%
60%
40%
20%
0%
6%
4%
2%
12%
1995
2000
2005
2010
2015
2020

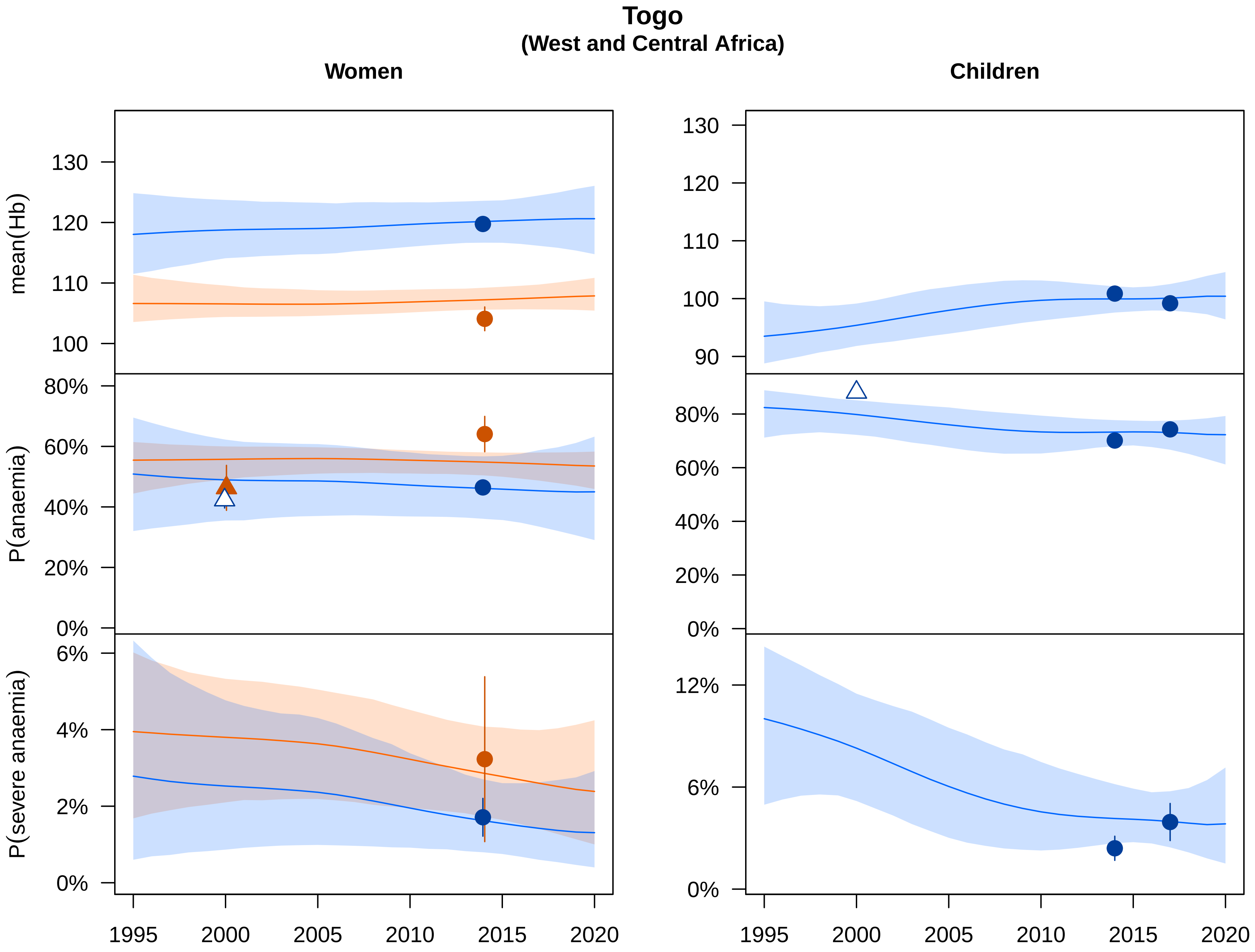

Togo
(West and Central Africa)
Women
Children
mean(Hb)
P(anaemia)
P(severe anaemia)
130
120
110
100
90
80%
60%
40%
20%
0%
6%
4%
2%
12%
1995
2000
2005
2010
2015
2020

Tunisia
(Central Asia, Middle East, and North Africa)

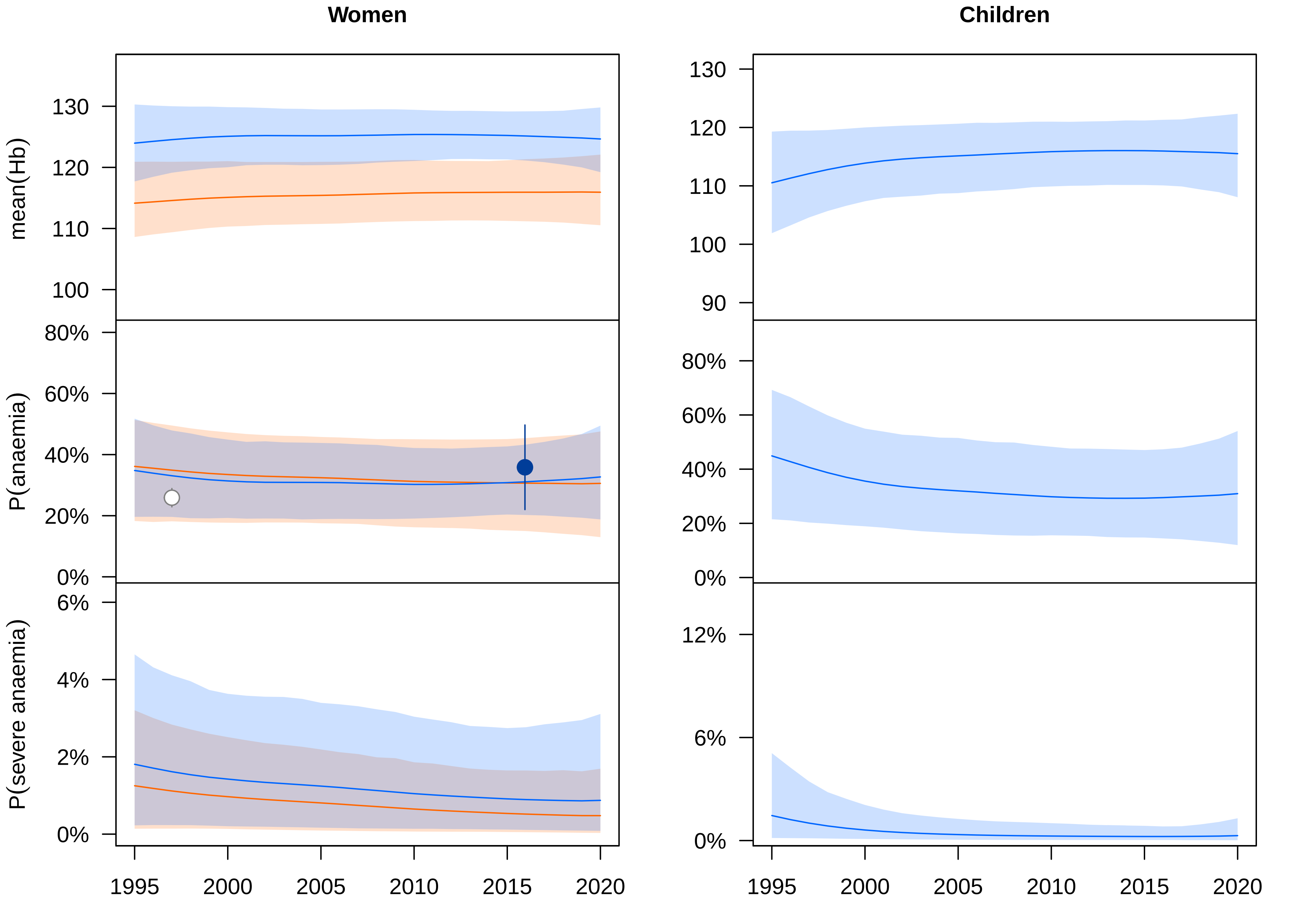

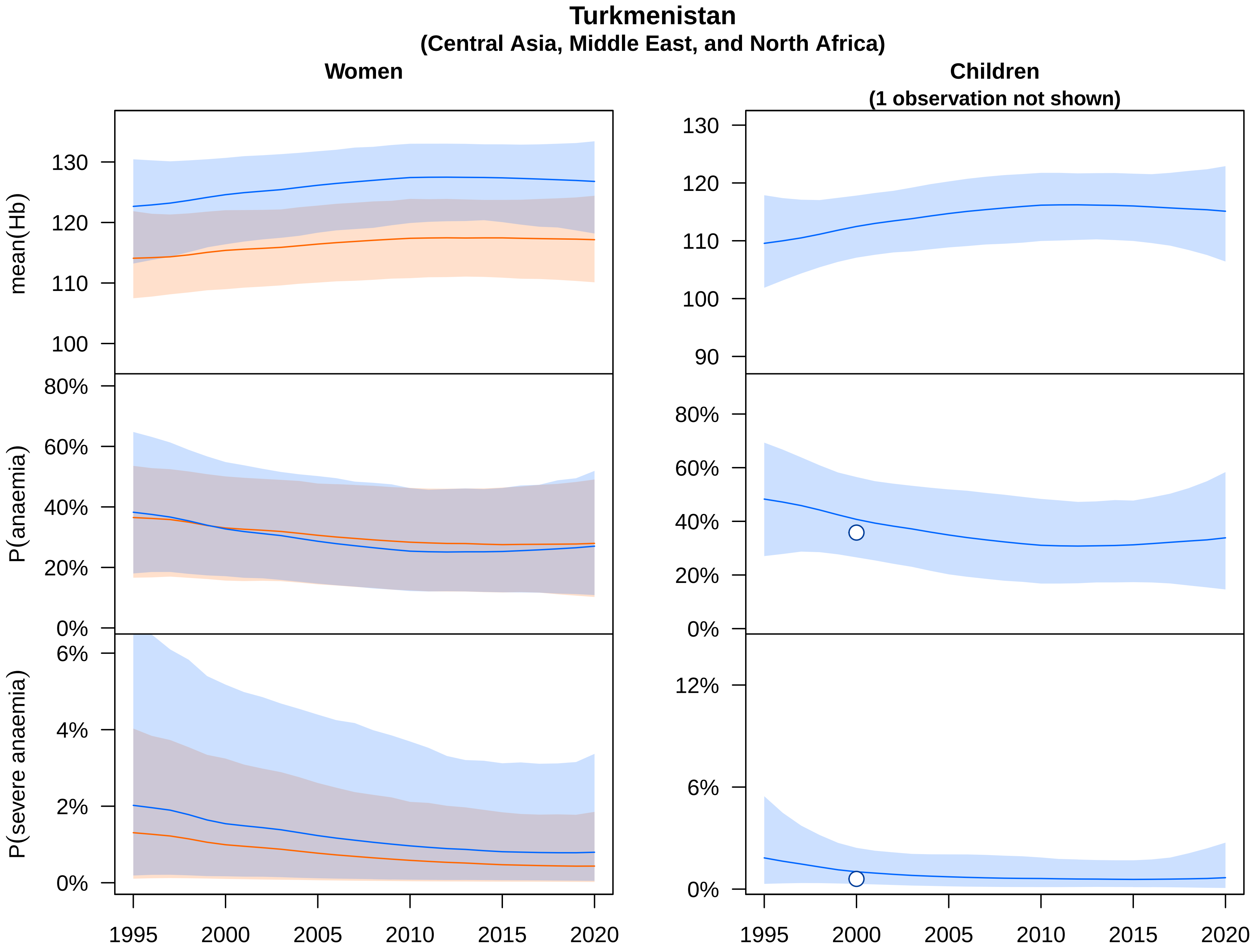

Turkmenistan
(Central Asia, Middle East, and North Africa)
Women
Children
(1 observation not shown)
mean(Hb)
P(anaemia)
P(severe anaemia)
130
120
110
100
90
80%
60%
40%
20%
0%
6%
4%
2%
12%
1995
2000
2005
2010
2015
2020

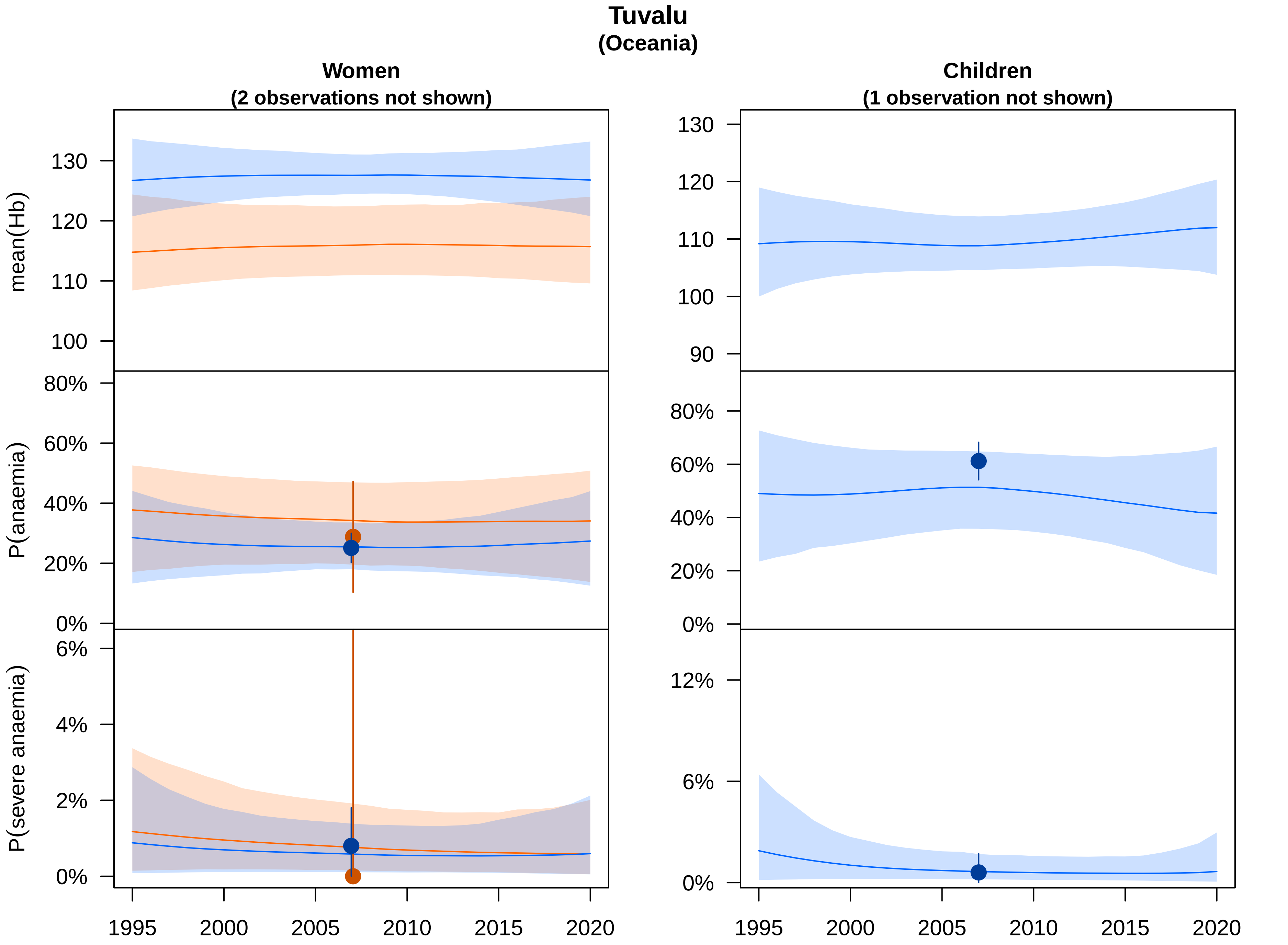

Tuvalu
(Oceania)
Women
(2 observations not shown)
Children
(1 observation not shown)
mean(Hb)
P(anaemia)
P(severe anaemia)
130
120
110
100
90
80%
60%
40%
20%
0%
6%
4%
2%
12%
1995
2000
2005
2010
2015
2020

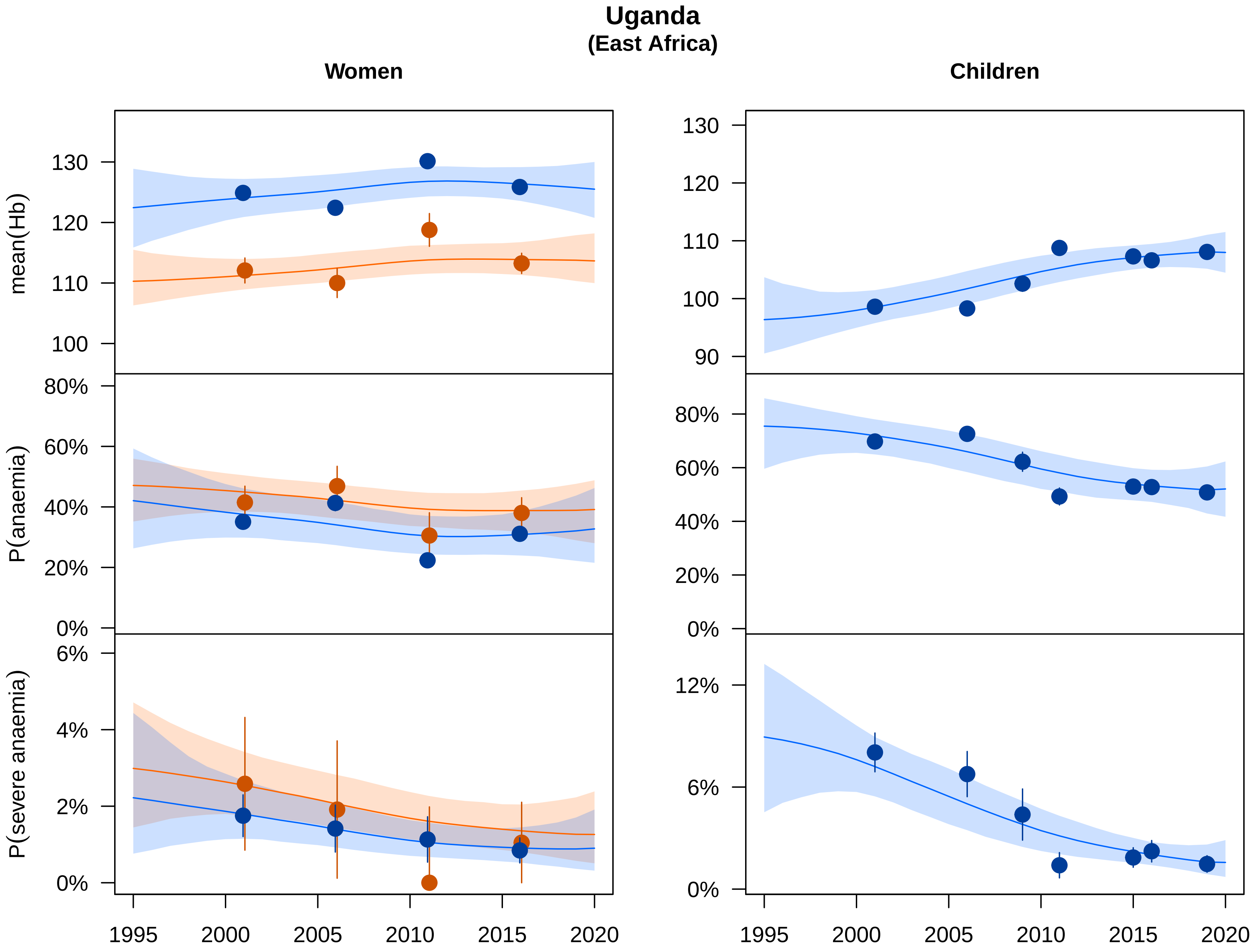

Uganda
(East Africa)
Women
Children
mean(Hb)
P(anaemia)
P(severe anaemia)
130
120
110
100
90
80%
60%
40%
20%
0%
6%
4%
2%
12%
1995
2000
2005
2010
2015
2020

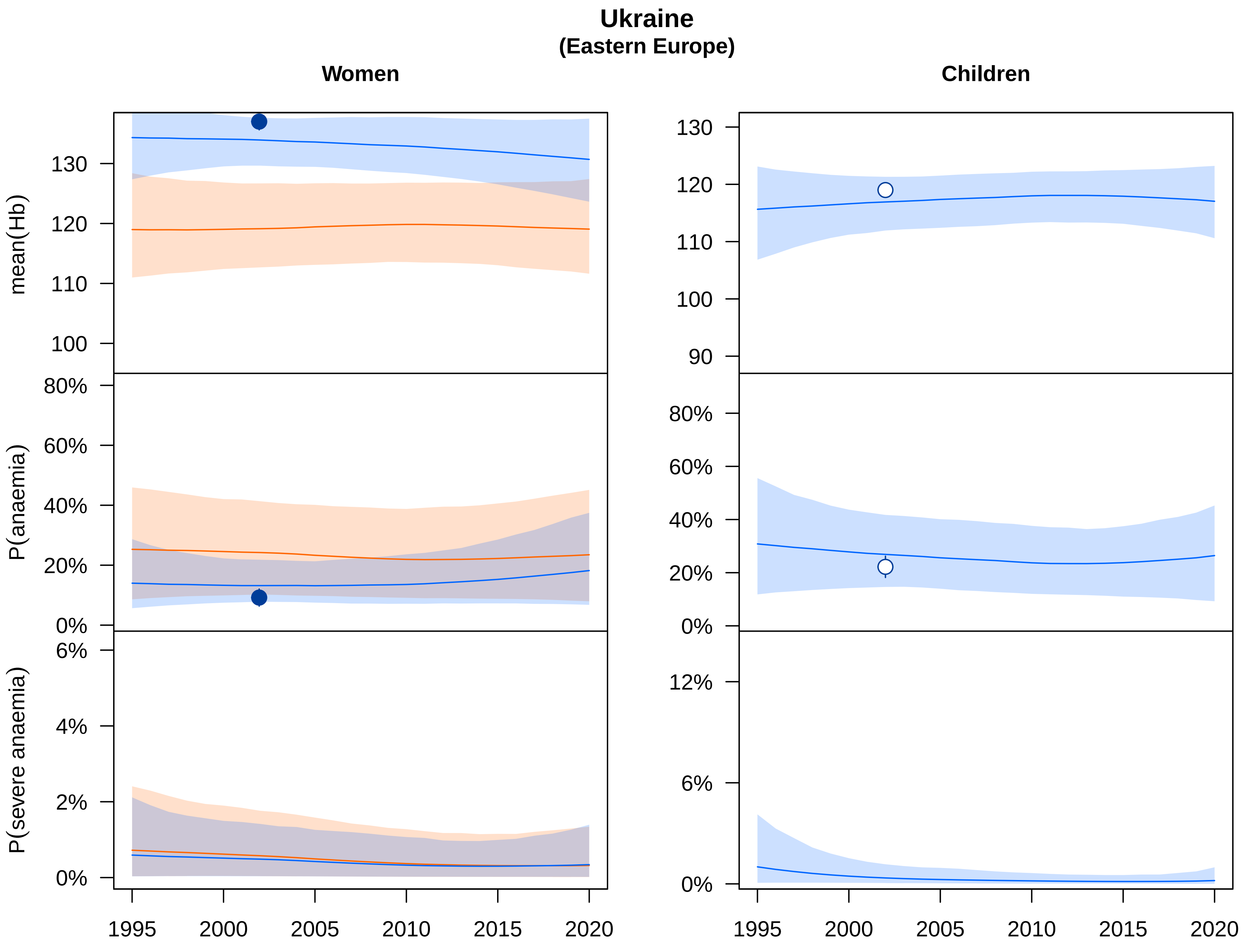
Ukraine
(Eastern Europe)
Women
Children
mean(Hb)
P(anaemia)
P(severe anaemia)
130
120
110
100
90
80%
60%
40%
20%
0%
6%
4%
2%
12%
1995
2000
2005
2010
2015
2020

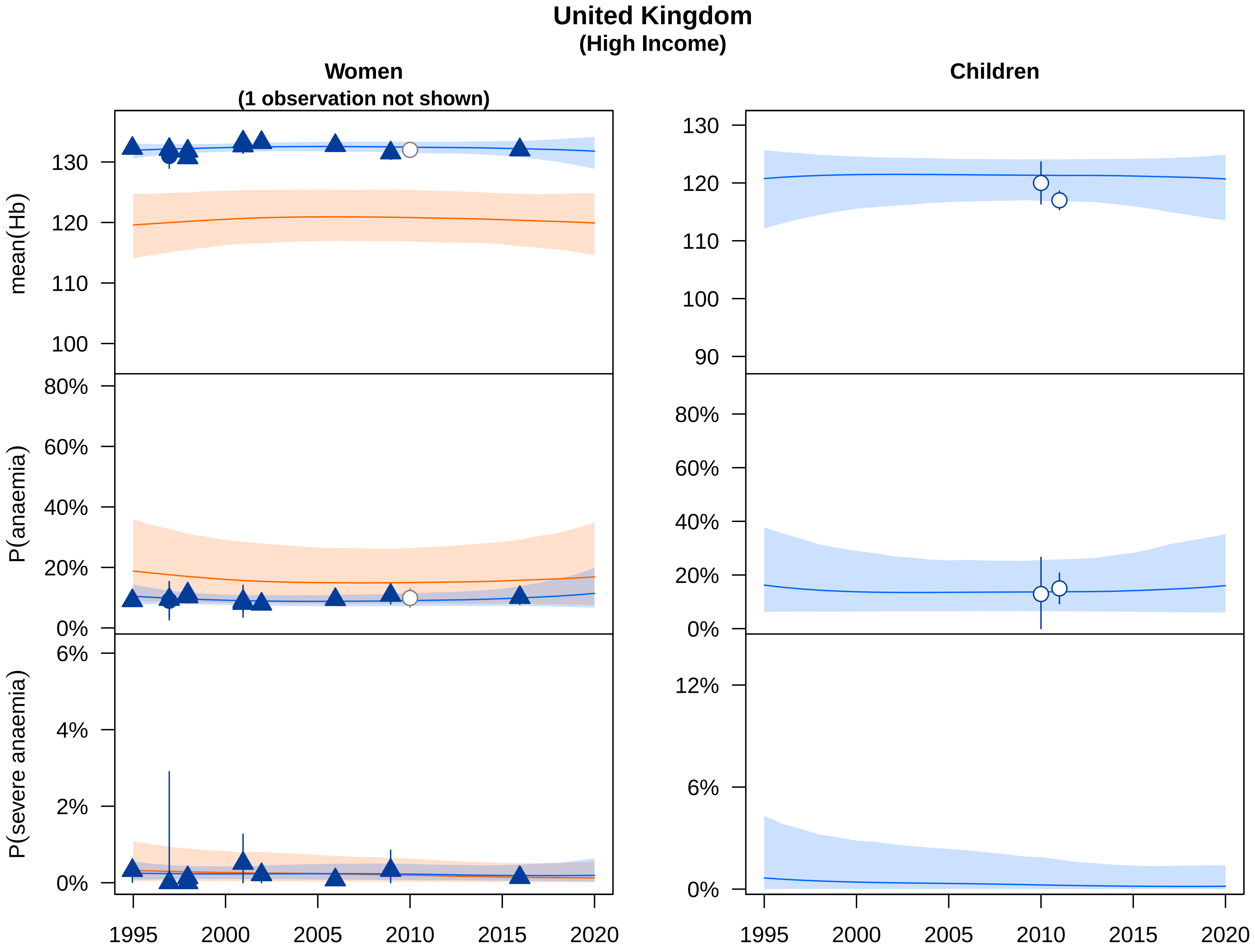
United Kingdom
(High Income)
Women
(1 observation not shown)
Children
mean(Hb)
P(anaemia)
P(severe anaemia)
130
120
110
100
90
80%
60%
40%
20%
0%
6%
4%
2%
12%
1995
2000
2005
2010
2015
2020

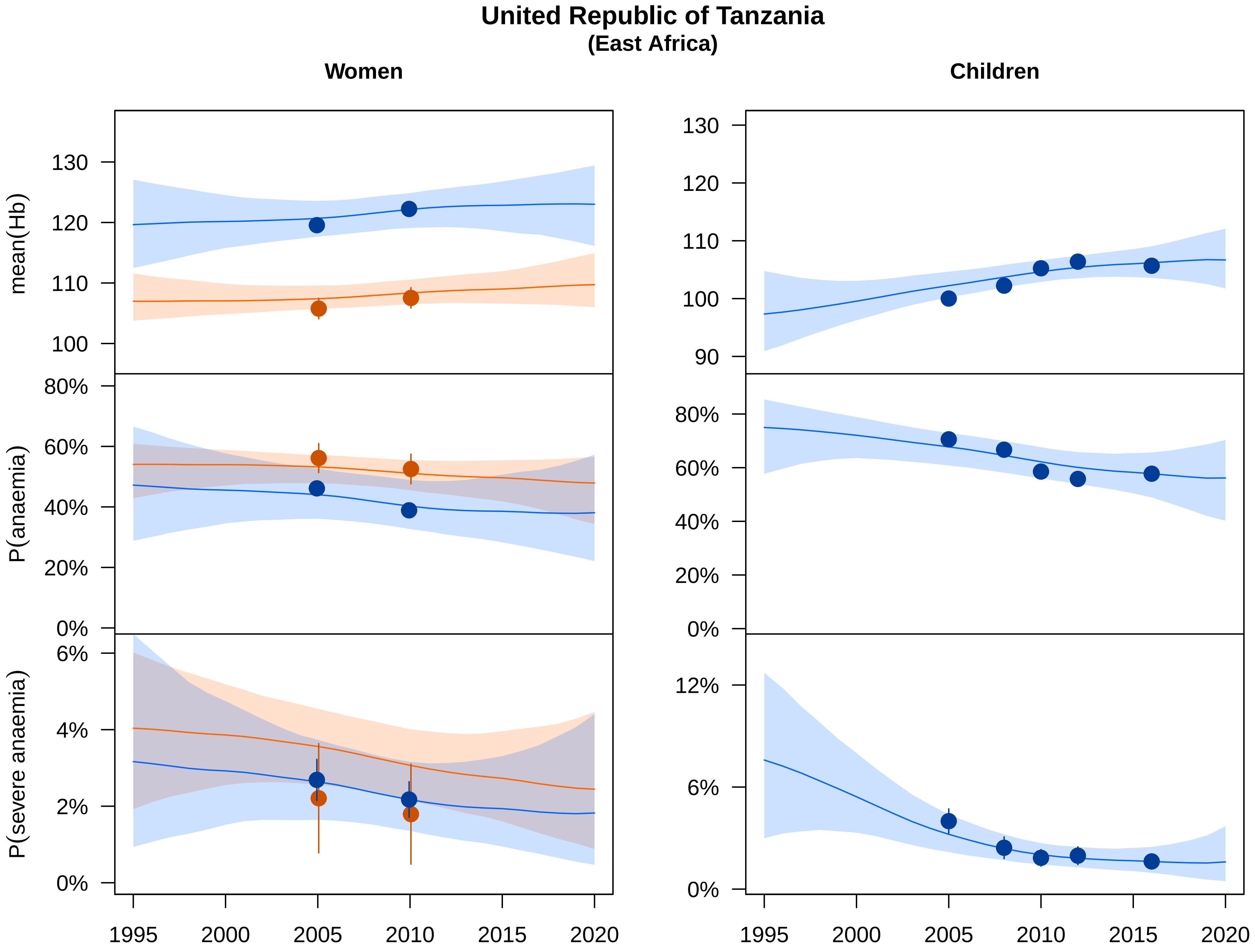
United Republic of Tanzania
(East Africa)
Women
Children
mean(Hb)
P(anaemia)
P(severe anaemia)
130
120
110
100
90
80%
60%
40%
20%
0%
6%
4%
2%
12%
1995
2000
2005
2010
2015
2020

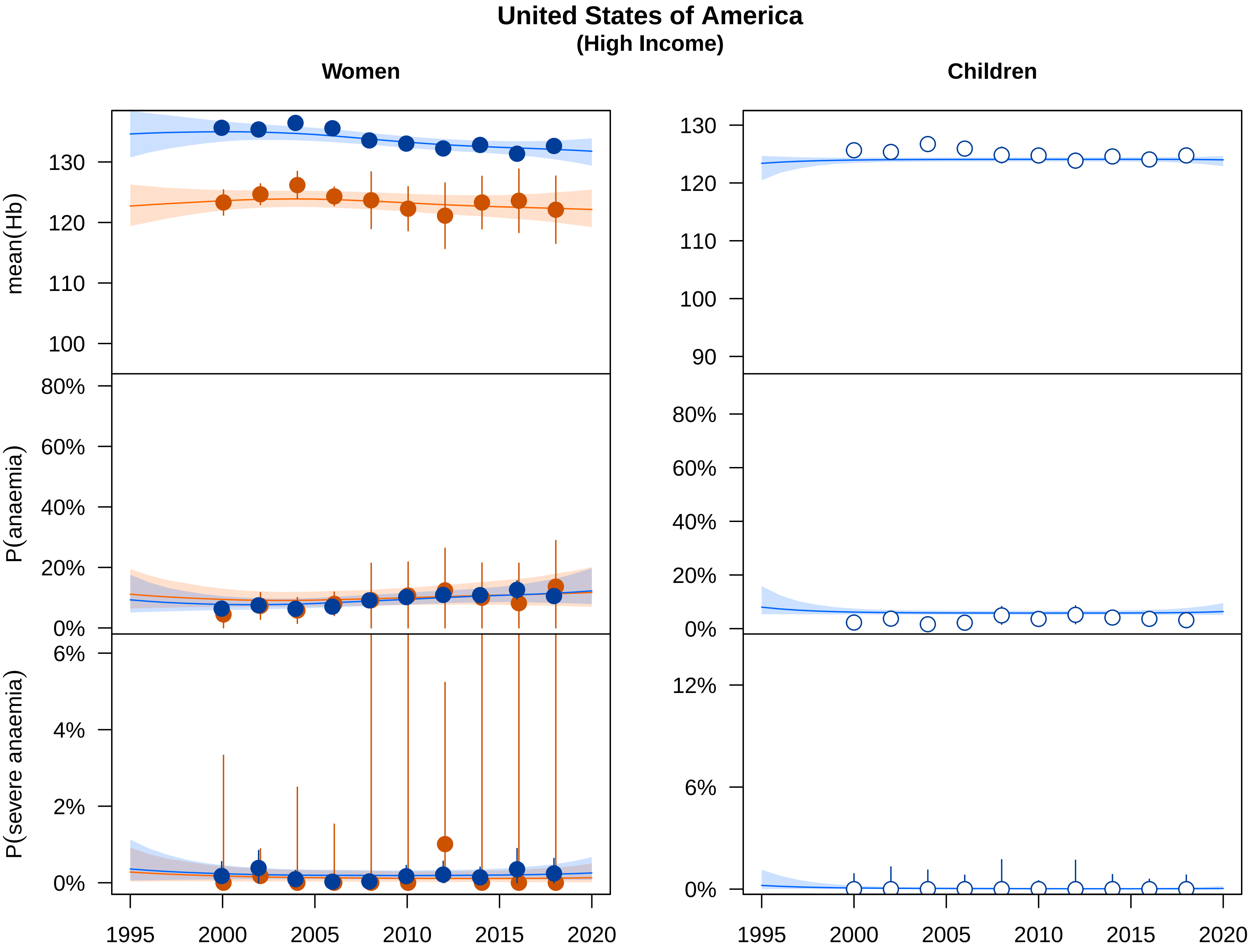
United States of America
(High Income)
Women
Children
mean(Hb)
P(anaemia)
P(severe anaemia)
130
120
110
100
90
80%
60%
40%
20%
0%
6%
4%
2%
12%
1995
2000
2005
2010
2015
2020

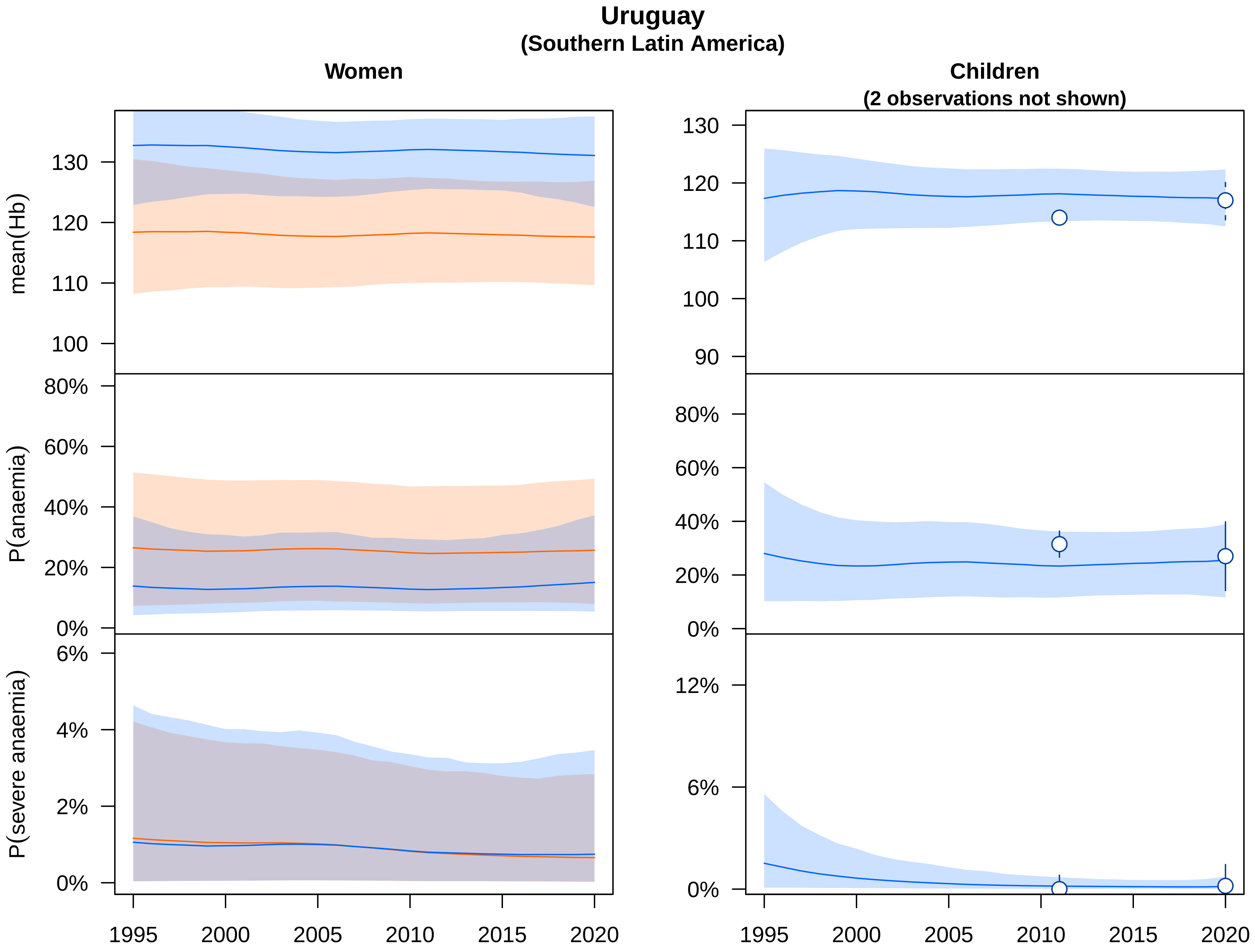

Uruguay
(Southern Latin America)
Women
Children
(2 observations not shown)
mean(Hb)
P(anaemia)
P(severe anaemia)
130
120
110
100
90
80%
60%
40%
20%
0%
6%
4%
2%
12%
1995
2000
2005
2010
2015
2020

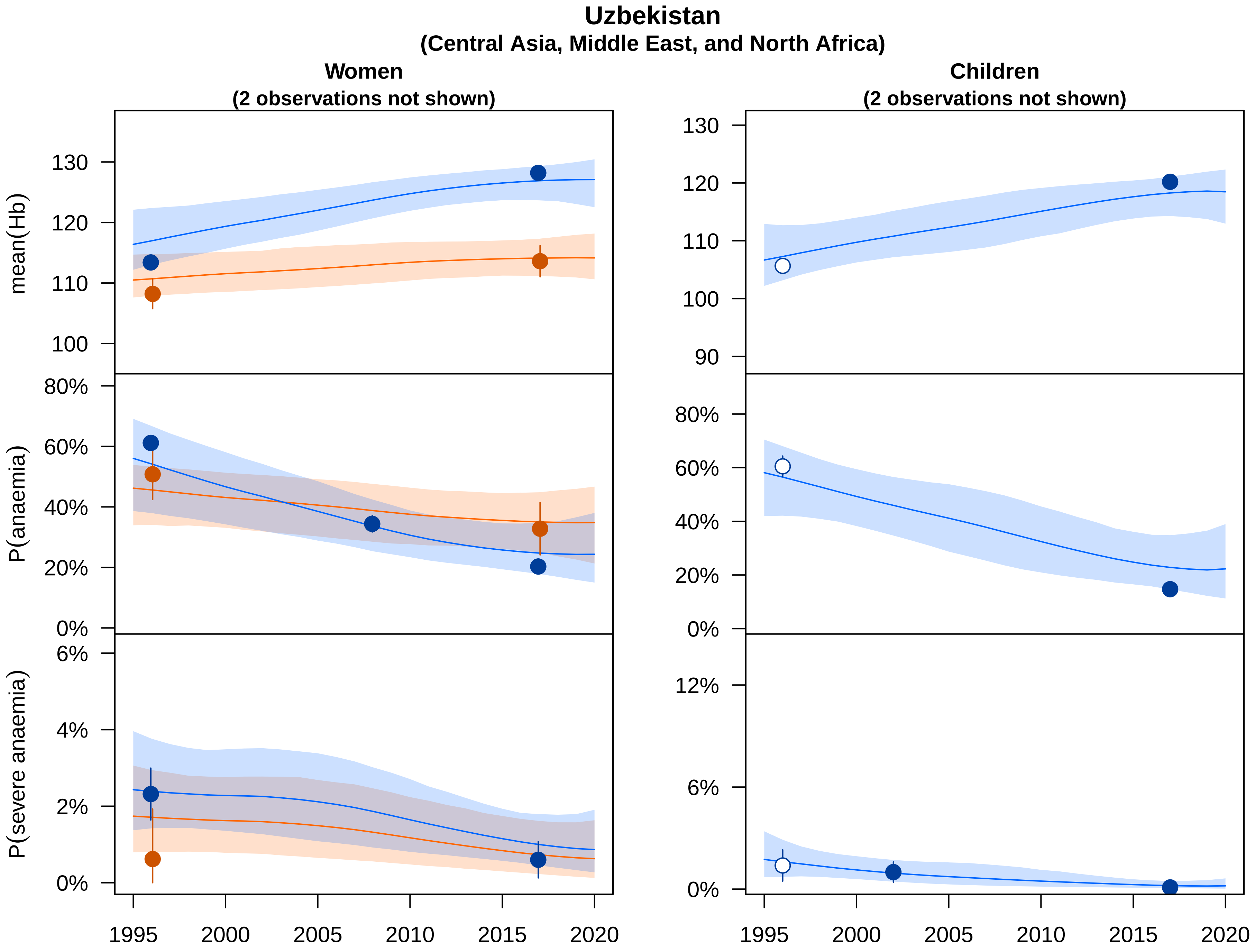
Uzbekistan
(Central Asia, Middle East, and North Africa)
Women
(2 observations not shown)
Children
(2 observations not shown)
mean(Hb)
P(anaemia)
P(severe anaemia)
130
120
110
100
90
80%
60%
40%
20%
0%
6%
4%
2%
12%
1995
2000
2005
2010
2015
2020

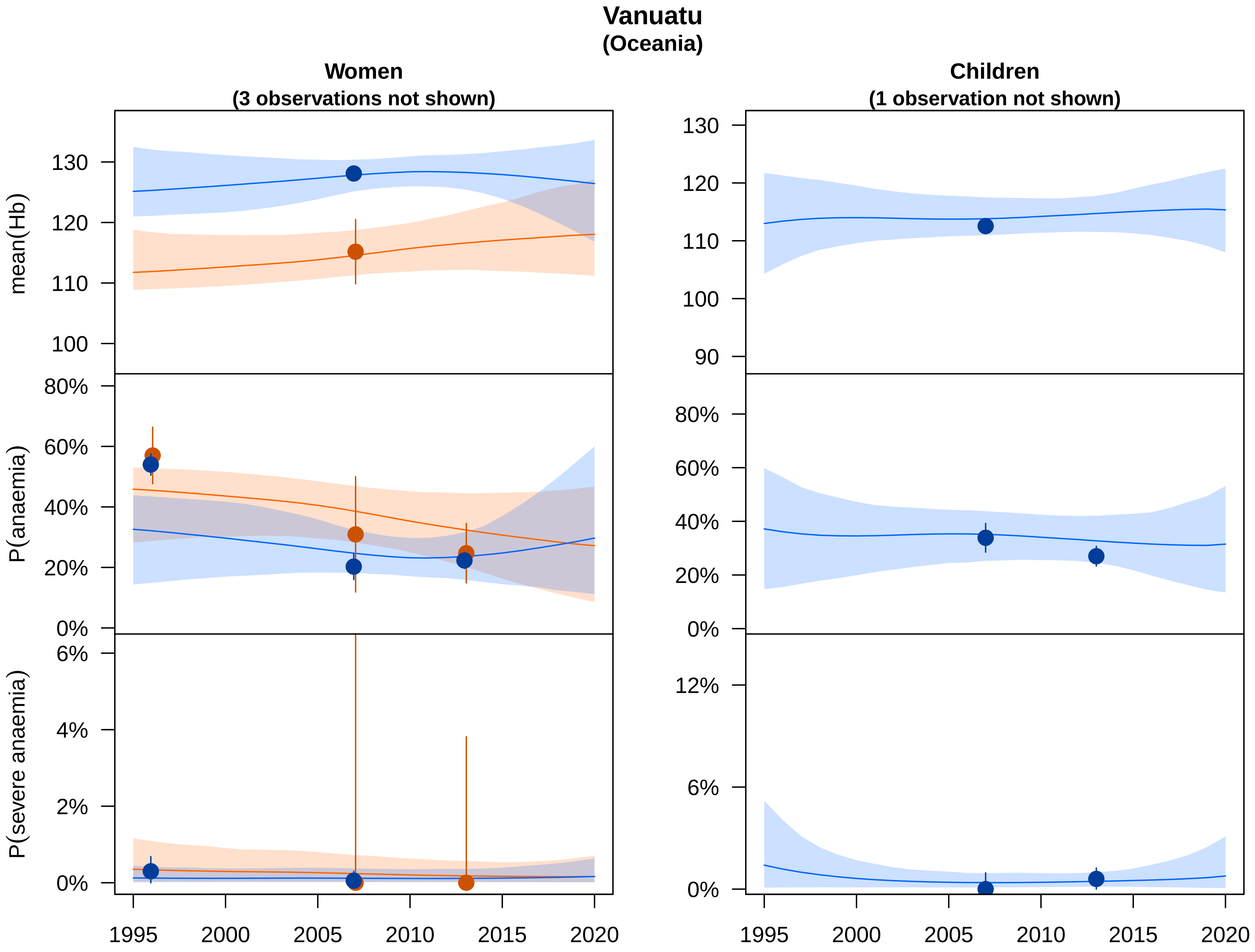
Vanuatu
(Oceania)
Women
(3 observations not shown)
Children
(1 observation not shown)
mean(Hb)
P(anaemia)
P(severe anaemia)
130
120
110
100
90
80%
60%
40%
20%
0%
6%
4%
2%
12%
1995
2000
2005
2010
2015
2020

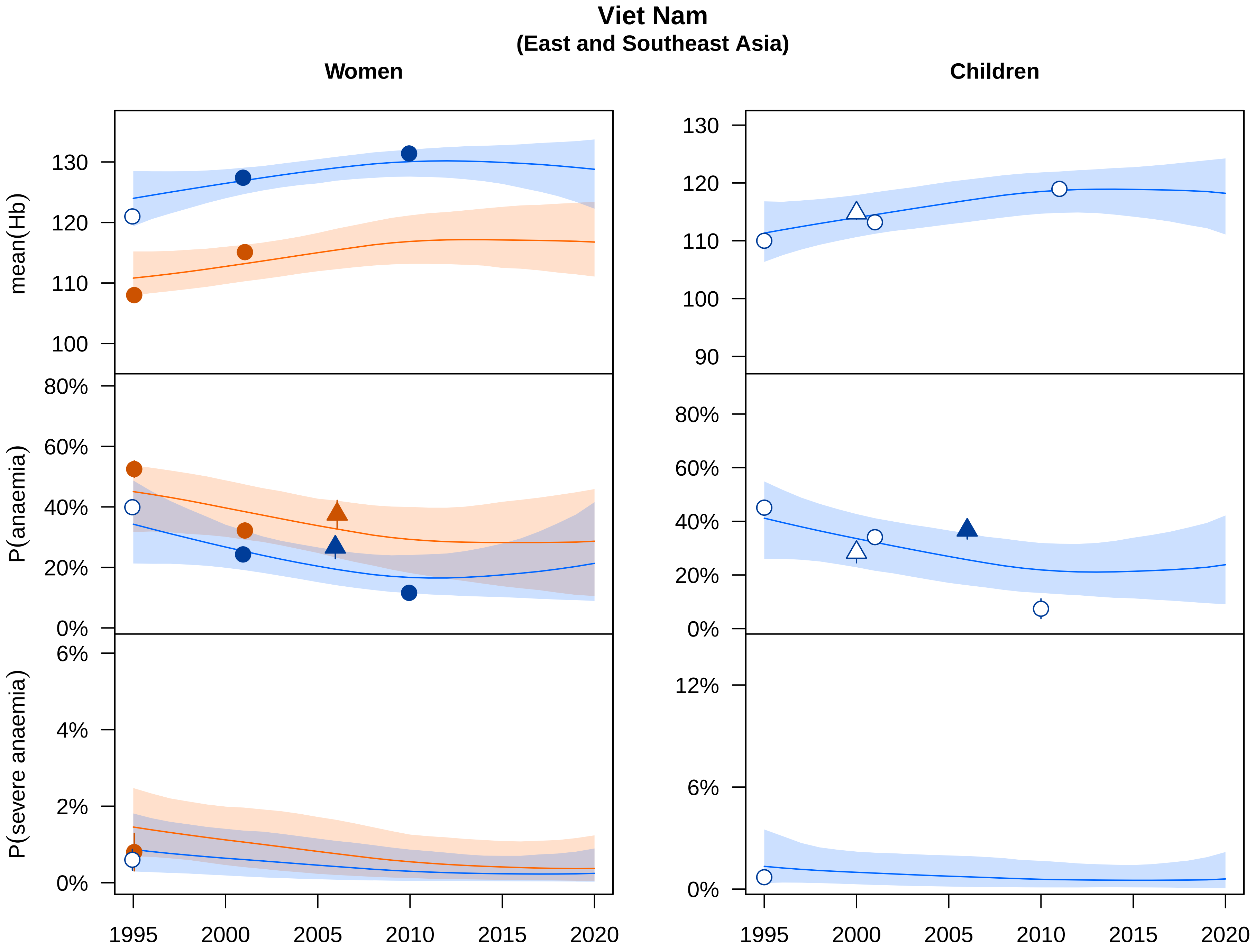

Viet Nam
(East and Southeast Asia)
Women
Children
mean(Hb)
P(anaemia)
P(severe anaemia)
100
110
120
130
90
0%
20%
40%
60%
80%
2%
4%
6%
6%
12%
1995
2000
2005
2010
2015
2020

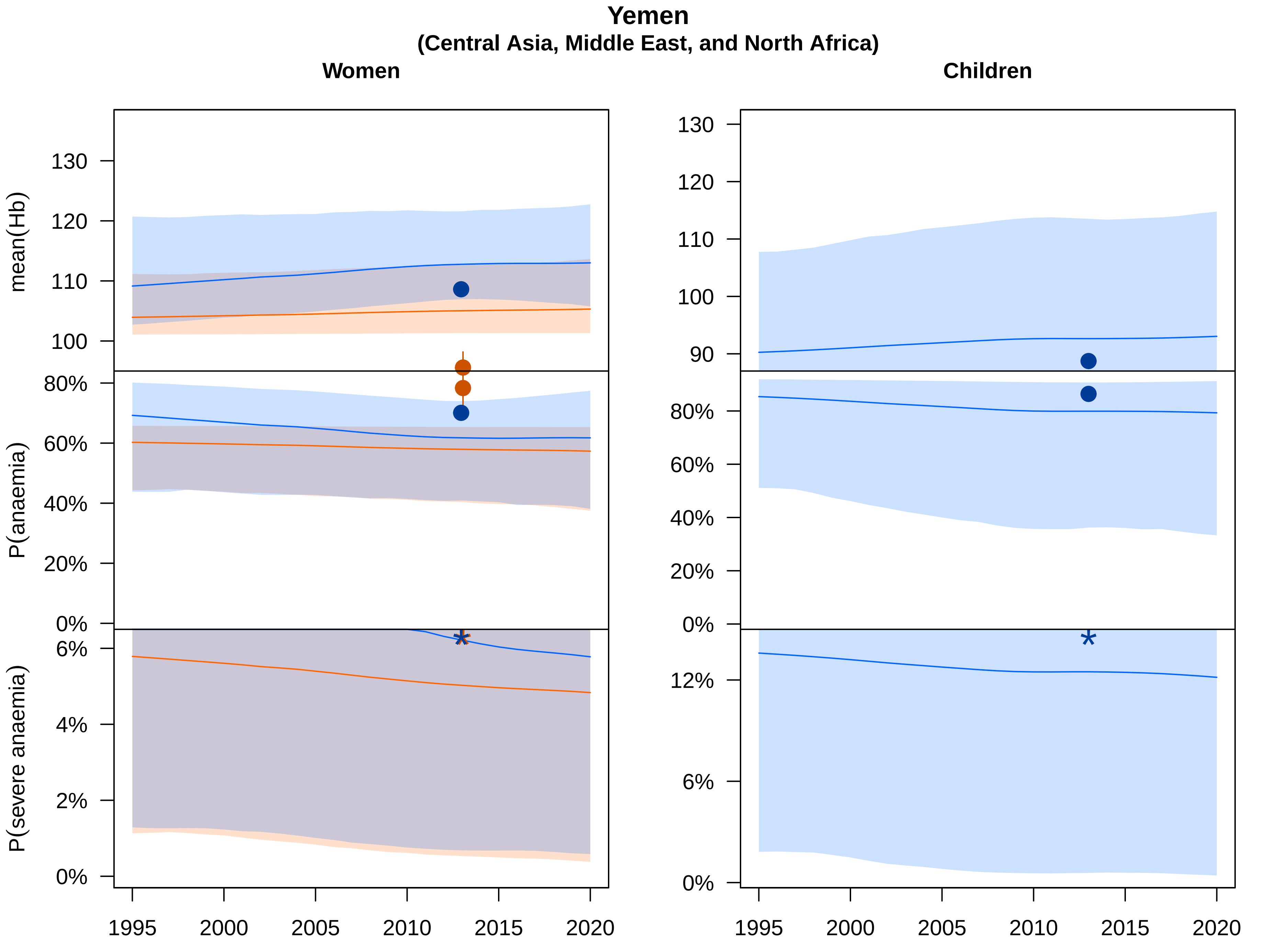

Yemen
(Central Asia, Middle East, and North Africa)
Women
Children
mean(Hb)
P(anaemia)
P(severe anaemia)
130
120
110
100
90
80%
60%
40%
20%
0%
6%
4%
2%
12%
1995
2000
2005
2010
2015
2020

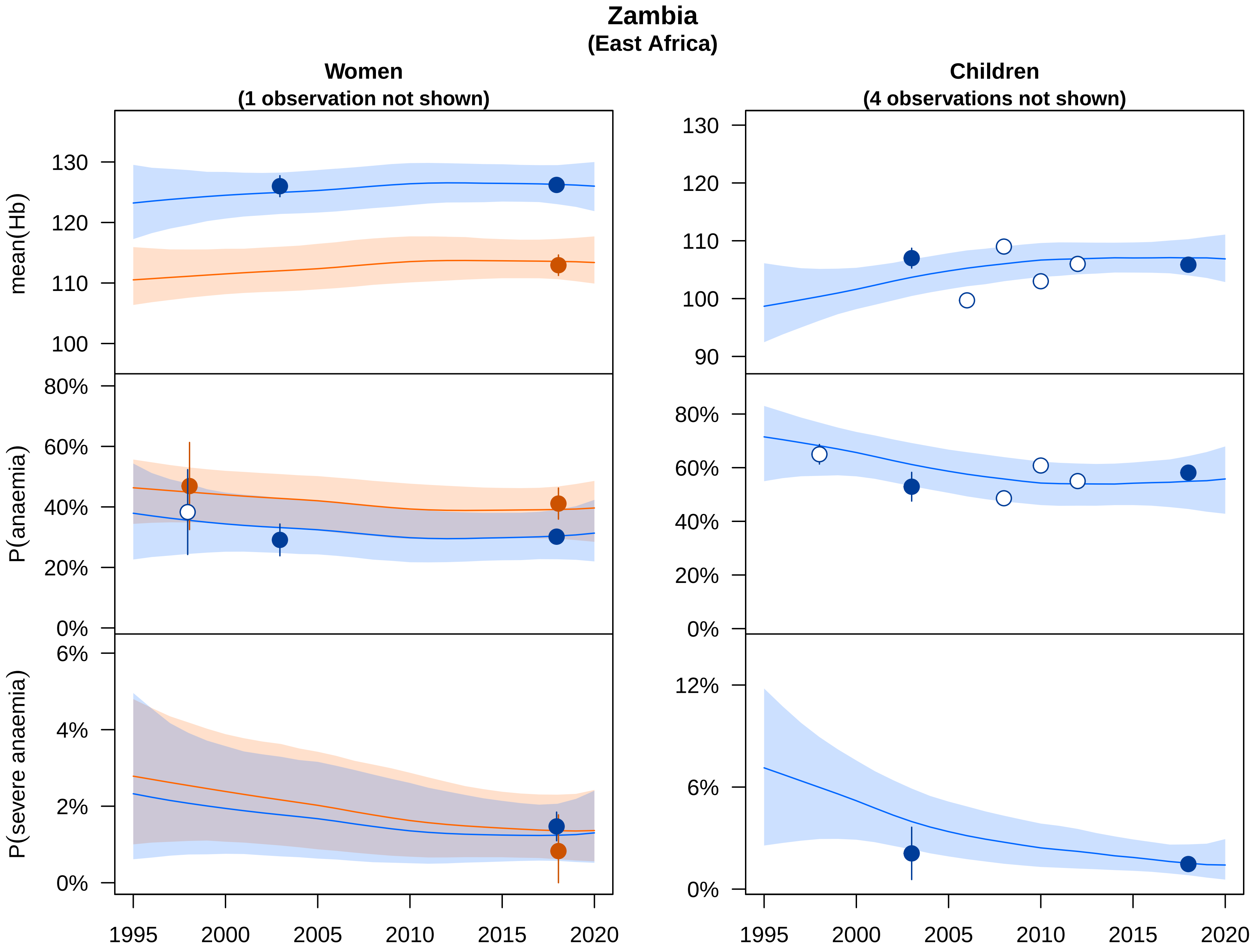

Zambia
(East Africa)
Women
(1 observation not shown)
Children
(4 observations not shown)
mean(Hb)
P(anaemia)
P(severe anaemia)
130
120
110
100
90
80%
60%
40%
20%
0%
6%
4%
2%
12%
1995
2000
2005
2010
2015
2020

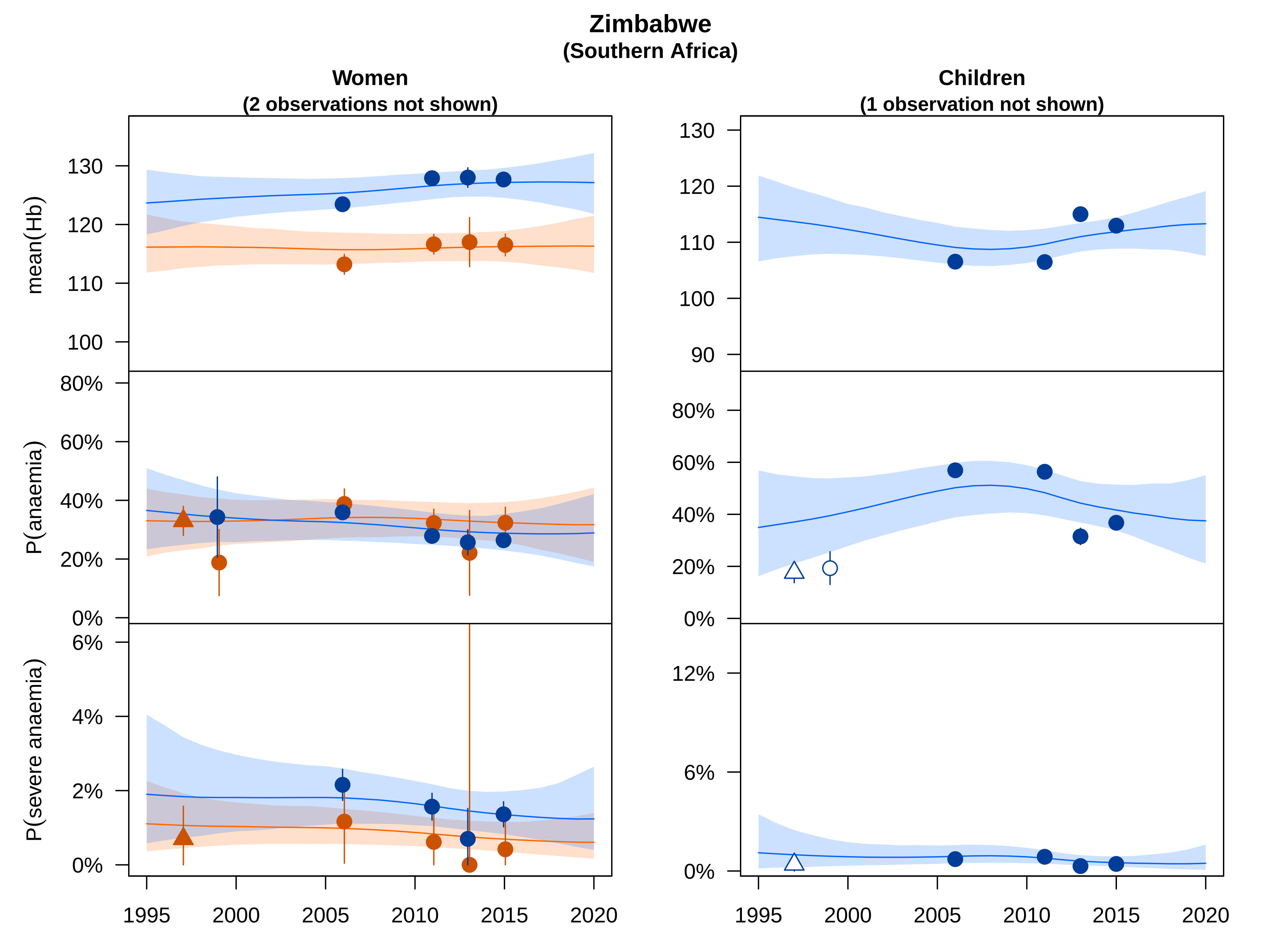

Zimbabwe
(Southern Africa)
Women
(2 observations not shown)
Children
(1 observation not shown)
mean(Hb)
P(anaemia)
P(severe anaemia)
130
120
110
100
90
80%
60%
40%
20%
0%
6%
4%
2%
12%
1995
2000
2005
2010
2015
2020
